# N-Protonated Acridinium Catalyst Enables Anti-Markovnikov Hydration of Unconjugated Tri- and Disubstituted Olefins

**DOI:** 10.1021/jacs.4c18185

**Published:** 2025-01-31

**Authors:** Boris Alexander van der Worp, Tobias Ritter

**Affiliations:** † Max-Planck-Institut für Kohlenforschung, Kaiser-Wilhelm-Platz 1, 45470 Mülheim an der Ruhr, Germany; ‡ Institute of Organic Chemistry, RWTH Aachen University, Landoltweg 1, 52074 Aachen, Germany

## Abstract

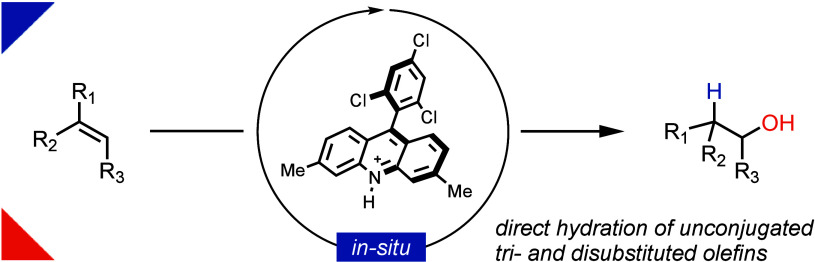

The preparation of alcohols with anti-Markovnikov selectivity directly from olefins and water is a sought-after reaction due to its atom-economy and potential cost-effectiveness. Herein, we present the first general method for direct, catalytic anti-Markovnikov hydration of unconjugated tri- and disubstituted olefins. The key advancement is made possible by an oxidative (E*_red_ = 2.15 V) *N*-H acridinium catalyst, which allowed for the functionalization of alkenes that were previously unreactive in such transformations due to their high oxidation potential. The developed protocol is not limited to hydration but also enables other hydrofunctionalizations, such as hydroetherifications, following the same mechanistic pathway.

Alcohols^1^ are used as solvents, fuels, detergents, plasticizers, and other specialty chemicals.^2,3^ The synthesis of alcohols often relies on the use of olefins as a readily available feedstock.^3^ Hydration of olefins, typically catalyzed by acid, yields alcohols with Markovnikov selectivity.^4,5^ In contrast, the preparation of alcohols with anti-Markovnikov selectivity is based on indirect methods and proceeds through the stoichiometric formation of other intermediates, resulting in the generation of an equivalent amount of waste (Scheme 1A).^6−11^ Progress in visible-light photoredox catalysis^12^ has enabled the direct anti-Markovnikov hydration and other hydrofunctionalizations of olefins via an alkene radical cation pathway (Scheme 1B).^13−16^ However, the developed protocols are limited to styrenes and olefins with oxidation potentials lower than 2.0–2.1 V, which leaves the majority of unconjugated alkenes outside the scope of the transformation. Therefore, the realization of a direct and catalytic anti-Markovnikov hydration across a broad range of olefins remains a significant challenge.^17^ In this paper, we report the first method for direct anti-Markovnikov hydration of unconjugated tri- and disubstituted alkenes, a process catalytic in all components except olefin and water (Scheme 1C). The reaction is facilitated by a novel, rationally designed *N*-protonated acridinium catalyst, characterized by an elevated excited-state reduction potential and sufficient stability. The combination of these two features embedded in the catalyst allowed us to target alkenes that were previously inaccessible for such transformations due to the high oxidation potential of these substrates (Scheme 1D).

**Scheme 1:**
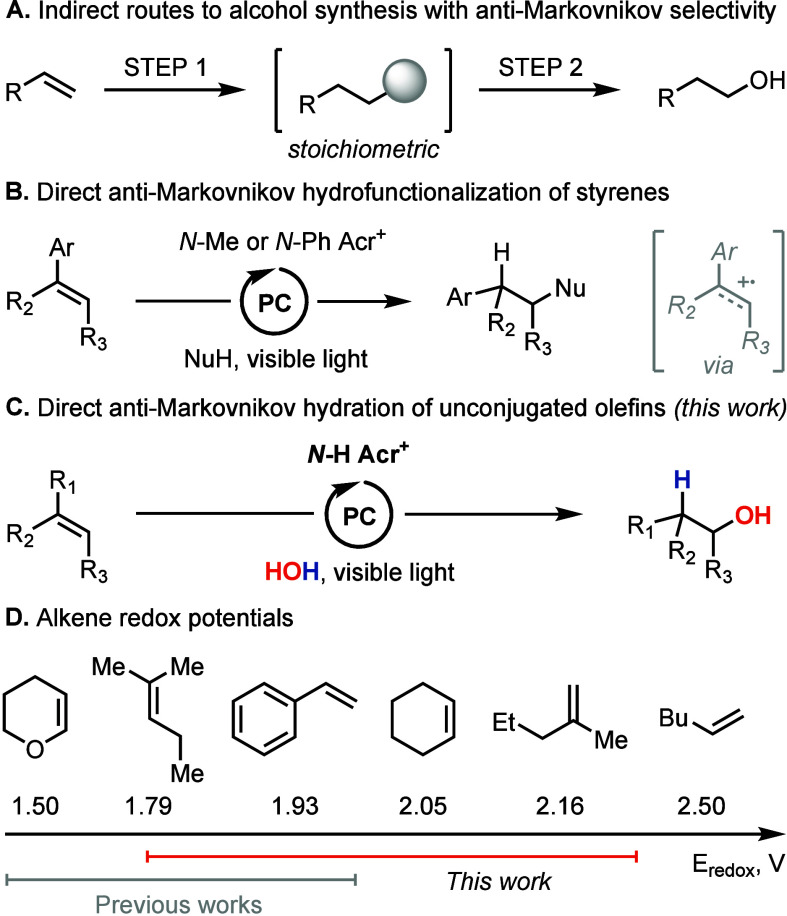
Approaches to the Synthesis of Alcohols with Anti-Markovnikov Selectivity

Today, industrial methods for the production of anti-Markovnikov alcohols rely on the Ziegler process, which delivers primary alcohols with an even number of carbon atoms via aluminum-mediated ethylene oligomerization followed by oxidation and hydrolysis,^6^ and reductive hydroformylation, which converts alkenes into alcohols with a one-carbon-atom extended chain.^7,8^ For smaller-scale production, the two-step hydroboration-oxidation protocol is commonly used due to its reliability and efficiency.^5,9^ Recent advances, such as Grubbs’ triple relay catalysis^10^ and regioselective epoxide hydrogenations,^11^ offer viable alternatives. Yet, all of these methods require stoichiometric reagents other than water to achieve formal anti-Markovnikov hydration of olefins and, except for Grubbs’ system, involve multiple steps. The rapid development of photochemical techniques in the last years has introduced new possibilities in this area.^12^ By harnessing the reactivity of free radical addition to olefins that often complements the traditional approaches of polar chemistry, visible-light photoredox catalysis has enabled diverse anti-Markovnikov hydrofunctionalizations of unactivated alkenes under mild conditions.^18−23^ Still, the direct addition of water remains unexplored, as both water’s high oxidation potential (*E*° > 3 V vs NHE)^24^ and the significant bond dissociation energy of the oxygen–hydrogen bond (BDE = 118.2 kcal/mol)^25^ hinder the facile generation of open-shell species from H_2_O. In this context, Nicewicz and coauthors pioneered a complementary strategy based on the one-electron oxidation of the olefins, followed by nucleophilic attack on the resulting radical cation.^13^ Utilizing a dual catalytic system of a strongly oxidative acridinium photoredox catalyst (E*_red_ = 2.04 V vs SCE) and a hydrogen atom transfer catalyst, they demonstrated hydroetherifications,^14a^ hydroaminations,^14b^ hydrohalogenations,^14c^ and other transformations of various alkenes,^13^ primarily styrenes. The Lei group later extended this concept to anti-Markovnikov hydration,^15^ while the Shu group applied it to the synthesis of primary amines.^16^ These seminal contributions have largely focused on the functionalization of privileged substrates, such as styrenes or enol ethers, owing to their accessible oxidation potentials. In contrast, functionalization of less electronically predisposed substrates, namely unconjugated tri-, di-, and monosubstituted alkenes, has been limited. Although sporadic examples exist for trisubstituted olefins, these have been restricted to reactions involving unfunctionalized hydrocarbons or favorable intramolecular cyclizations. Anti-Markovnikov hydrofunctionalization of disubstututed or primary olefins through the one-electron oxidation pathway is currently unknown. With these limitations in mind, we sought to develop a method for anti-Markovnikov hydration of alkenes with oxidation potentials higher than those of styrenes,^26^ thereby expanding the scope of olefins amenable to this and related transformations.

The general mechanism for anti-Markovnikov olefin hydrofunctionalization through the intermediate formation of alkene radical cations is shown in Scheme 2A.^13,27^ The key step that initiates the catalytic cycle is the one-electron oxidation of the olefin by a photooxidant. To empirically assess different photooxidants, we selected the conversion of disubstituted alkene **5a** to alcohol **6a** in an acetone/water solvent mixture as our model reaction (Scheme 2B). The initial catalyst choices—9-mesityl-10-methylacridinium perchlorate (Fukuzumi catalyst, Acr_*Fuk*_) and 9-mesityl-3,6-di-*tert*-butyl-10-phenylacridinium tetrafluoroborate (Nicewicz catalyst, Acr_*Nic*_)—proved ineffective to catalyze the transformation, with a maximum yield of 12%, as detected by ^1^H NMR spectroscopy. Although these widely used acridinium dyes are among the most powerful photooxidants known to function under visible-light irradiation,^28^ their reduction potentials remain insufficient for the oxidation of disubstituted olefins and most other unconjugated alkenes, except for certain trisubstituted ones. From our previous work,^23^ we knew that acridine with a 9-(2-chlorophenyl) group, when protonated, possessed a greater excited-state reduction potential (E*_red_(**1**H^+^BF_4_^–^) = 2.19 V) than the conventional acridinium dyes. Applying the acridine **1** together with the strong mineral acid HBF_4_ to generate the catalytically active acridinium species (**1**H^+^BF_4_^–^) in situ, we observed formation of the desired product **6a** in 40% yield. Seeking to further increase the ground-state reduction potential of **1** we replaced the 2-chlorophenyl group at the 9-position with a more electron-deficient 2,4,6-trichlorophenyl moiety. The new catalyst **2**, upon protonation, demonstrated a higher reduction potential, but did not raise the reaction yield substantially. A common observation with catalysts **1** and **2** was incomplete conversion of the starting material **5a**. This fact, along with our failure to detect residual quantities of **1** and **2** in the crude mixture after reaction, indicated potential catalyst degradation. To address this issue, we aimed to enhance stability of the used dye and prepared 10-phenylated and 3,6-dimethylated versions of **2**, catalysts **3**^**+**^ and **4**, respectively. Catalyst **3**^**+**^ did not show improved performance; however, its redox properties illuminated differences between analogous *N*-H and *N*-Ph acridiniums (Supporting Information, pages S62–S75). Compared to **2**H^+^, the replacement of the acridine nitrogen proton with a phenyl group increased the **3**^+^ ground-state reduction potential but, at the same time, shifted both absorption and emission spectra to longer wavelengths, leading to a lower excitation energy (*E*_0,0_). As the result, the decrease in *E*_0,0_ balanced the gain in ground-state reduction potential, and the excited-state reduction potential of catalyst **3**^**+**^ remained identical to that of **2**H^+^ (E*_red_ = 2.28 V). Dimethylated catalyst **4** demonstrated higher stability and, despite a slight loss in reduction potential, afforded full conversion in the reaction. Based on collected observations, we posit that the combination of two factors, namely, the high excited-state reduction potential and the stability toward side-reactivity at the 3,6 positions of the acridine core make **4**H^+^ an efficient catalyst for anti-Markovnikov hydration of **5a**. As for the choice between *N*-H and *N*-Ph catalysts, our further development focused on *N*-H acridiniums. We envision that the facile Lewis^29^ or Brønsted^23,30^ acid-mediated interconversion between neutral acridines and positively charged acridiniums has a potential to provide versatile opportunities in catalysis, allowing access to both PCET^31−34^ and SET^35^ reactivity modes with a single catalyst. The acridine **4** can be easily prepared in two steps from commercially available reagents and activated in situ by the addition of a strong acid. A slight excess of acid was beneficial to ensure complete protonation of the acridine species in solution (Supporting Information, pages S30–S32). Optimization of the reaction conditions identified the use of 5 mol % of both acridine and HAT catalysts, 10 mol % aqueous HBF_4_, and an acetone/H_2_O (5/1, v/v) solvent mixture at a concentration of 0.33 M, with 18 h of blue light (450 nm) irradiation, as the preferred conditions (Supporting Information, pages S28–S30).

**Scheme 2:**
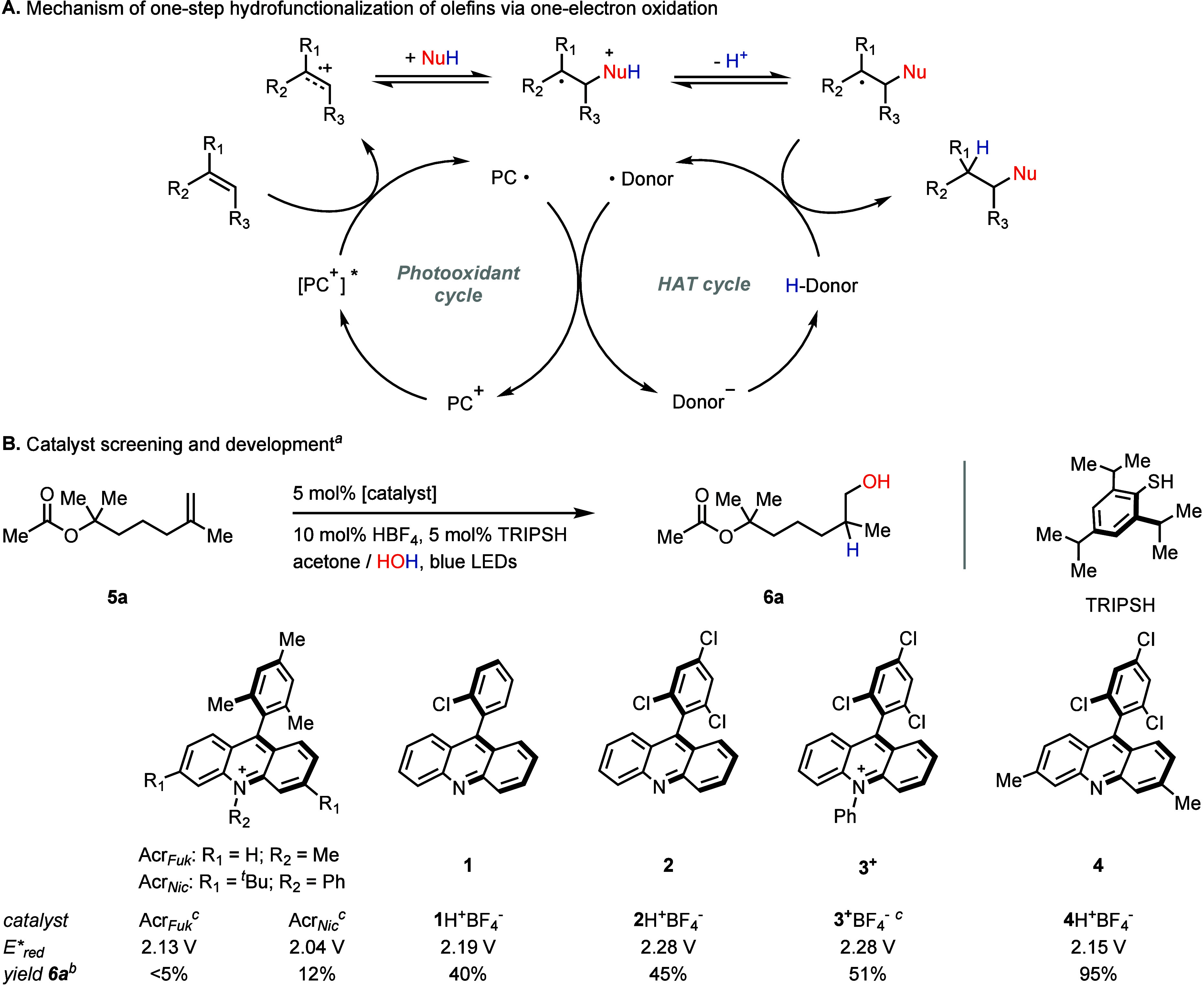
Reaction Mechanism and Catalyst Development Reactions were conducted on a 0.1 mmol scale. Exact reaction conditions included 5 mol % catalyst, 5 mol % TRIPSH, 10 mol % aq. HBF_4_ solution, acetone/H_2_O solvent mixture (5/1, 0.33 M), 18 h of irradiation at 450 nm. Nu, nucleophile; PC, photocatalyst; Acr, acridinium; HAT, hydrogen atom transfer. Yields were determined by ^1^H NMR spectroscopy of the reaction’s crude material. HBF_4_ was not used.

The scope of the developed reaction is shown in Scheme 3. We first focused on 1,1-disubstituted alkenes, a class of substrates previously inaccessible to functionalization via one-electron oxidation. The hydration worked smoothly across a broad range of olefins and produced anti-Markovnikov products with consistently high regioselectivity (>25:1). Ketones, esters, ethers, alcohols, and carboxylic acids were tolerated under the reaction conditions. Aldehydes, as exemplified by entry **6f**, also remained unaffected. The reaction left triple bonds intact (**6m**), and other potentially labile compounds, such as tertiary esters (**6a**) and alcohols (**6o**), yielded the desired products without any signs of decomposition or isomerization. Electron-deficient and electron-neutral aromatic compounds (e.g., **6c**, **h**, **j, k**) posed no challenges either. The developed acridine catalyst **4**, in its protonated form, did not possess a reduction potential high enough to oxidize α-olefins or electron-deficient alkenes. This fact allowed us to selectively functionalize disubstituted double bonds in the presence of primary alkenes, as well as *α,β*-unsaturated carbonyl and carboxyl compounds (entries **6n**, **f**, **g**, **i**).

**Scheme 3:**
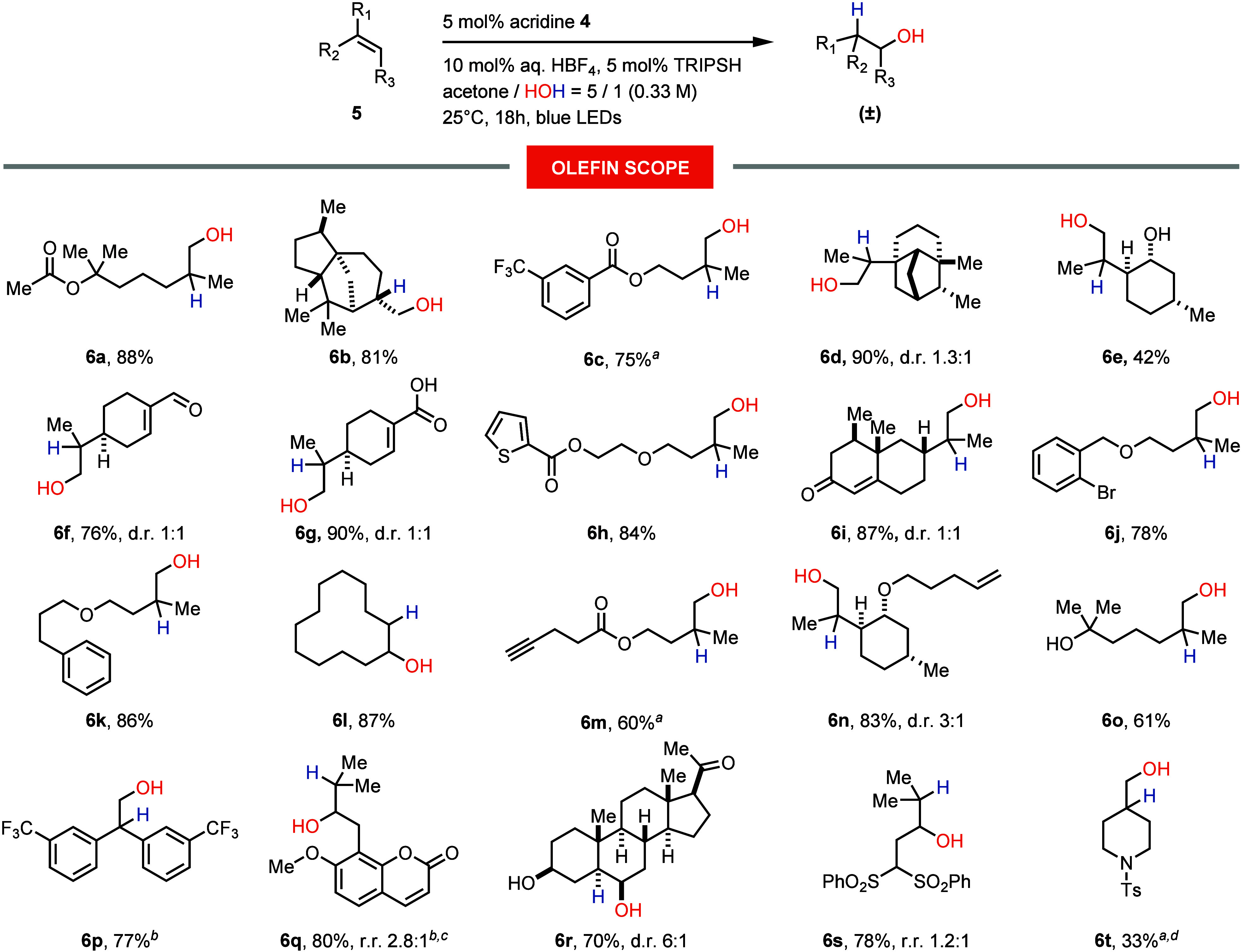
Hydration Reaction Scope Reaction time is 24 h. 10 mol % TRIPSH was used. Reaction time is 36 h. 10 mol % acridine **4**, 15 mol % HBF_4_ were used. Reactions were conducted on 0.5 mmol scale. Regiosiomeric (r.r.) and diastereoisomeric (d.r.) ratios were determined by ^1^H NMR of the reaction’s crude material. r.r. > 25:1 in all cases unless otherwise noted.

Besides the 1,1-disubstituted alkenes, the developed methodology proved effective for other, more readily oxidizable olefins. Alcohol **6p** was prepared from the electron-deficient styrene **5p**, whose hydration is challenging for both the Fukuzumi and Nicewicz catalysts (Supporting Information, pages S33–S35). Similarly, alcohol **6s** was successfully synthesized from trisubstituted olefin **5s**, which featured two strong electron-withdrawing groups (PhSO_2_−) in the β-position to the double bond. Other trisubstituted olefins, such as **5r** and **5q**, also yielded the respective alcohols, with **6q** representing the only example where the developed system tolerated an electron-rich aromatic substrate (Supporting Information, pages S78–S81). Compared to the 1,1-disubstituted alkenes, hydration of trisubstituted olefins proceeded with a diminished regioselectivity. With the exception of **6r**, which was detected as the only constitutional isomer, the anti-Markovnikov to Markovnikov product ratio for other trisubstituted substrates ranged from 3:1 to 1:1, depending on the substrate’s structure. This ratio was independent of the photocatalyst, HAT catalyst, or solvent used in the reaction (Supporting Information, pages S32–S33). We assume that the change in regioselectivity is of fundamental nature and reflects the difference in the stabilities of the radical intermediates formed after nucleophilic attack on the distonic alkene radical cation (primary vs tertiary in the case of 1,1-disubstituted alkenes and secondary vs tertiary in the case of trisubstituted alkenes).

To gain a better understanding of the operational limits of the developed catalytic system, the oxidation potentials of a series of alkenes were estimated semiempirically (Supporting Information, pages S75–S78). The results are shown in Figure 1. The model 1,1-disubstituted alkene, 2-methyl-1-pentene, exhibited a predicted oxidation potential (*E*_PRED_) of 2.16 V, which aligned closely with the reduction potential of **4**H^+^ (E*_red_ = 2.15 V) and substantiated the observed efficient reactivity of various of 1,1-disubstituted alkenes under the standard reaction conditions. In contrast, β-acetoxy-1,1-disubstituted alkene showed a significantly higher oxidation potential of 2.33 V. This finding corresponded with the extended irradiation times (24 h) required to achieve high conversion in the synthesis of alcohols **6c** and **6m**. Together with the data on nonreactive substrates (Supporting Information, Figure S2), these results delineate the functional range of **4**H^+^ to alkenes with oxidation potentials up to 2.33–2.43 V, beyond which the oxidation potency of **4**H^+^ becomes insufficient to sustain catalytic turnover.

**Figure 1:**
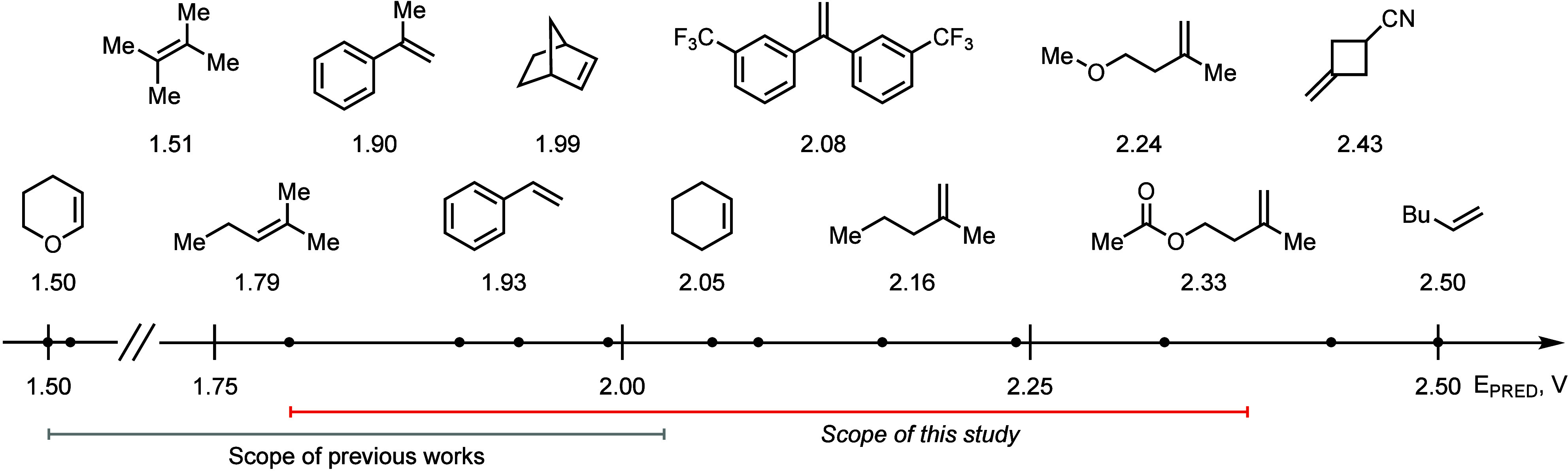
Alkene oxidation potential series.

Finally, the developed protocol could be applied to other hydrofunctionalizations beyond hydration. Varying the nucleophile, we carried out the addition of alcohols such as methanol, ethanol, and isopropanol across the double bond of olefin **5k**, producing ethers **7**–**9** (Scheme 4).

**Scheme 4:**
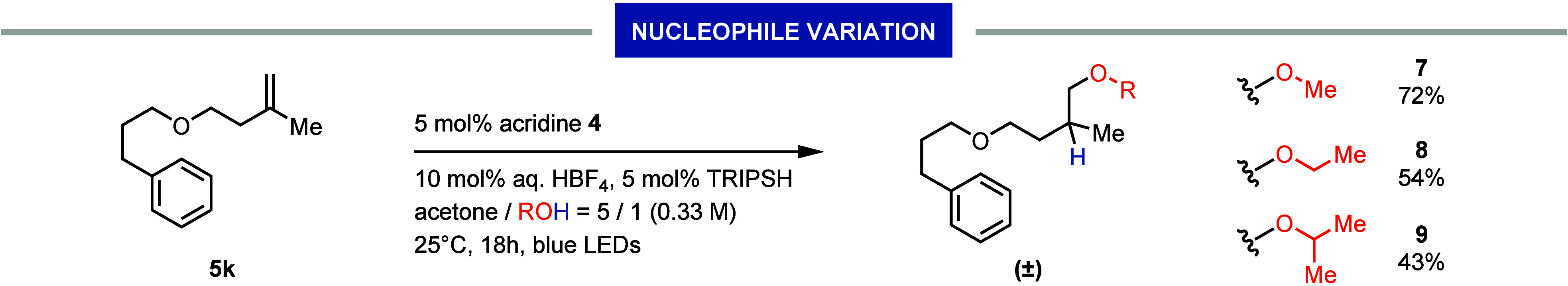
Reactivity with Other Nucleophiles Reactions were conducted on 0.5 mmol scale. r.r. > 25:1 in all cases.

We realized the hydration of a broad range of unconjugated tri- and disubstituted alkenes. Central to this success was the rational design of a novel *N*-protonated acridinium catalyst, which features an elevated excited-state reduction potential and sufficient stability. We hope that our contribution will bring the chemistry community one step closer to solving the fundamental problem of direct, catalytic anti-Markovnikov hydration of α-olefins.

## Supporting Information Available

Experimental procedures, analytical methods, and spectroscopic data for the synthesized compounds (PDF)
