# Supplementary material for: N-Protonated Acridinium Catalyst Enables Anti-Markovnikov Hydration of Unconjugated Tri- and Disubstituted Olefins
Source: J Am Chem Soc. 2025 Jan 31;147(6):4736–42. doi: 10.1021/jacs.4c18185 (PMC11826999; doi:10.1021/jacs.4c18185)
Supplement: Supplementary file 1 — ja4c18185_si_001.pdf [file ja4c18185_si_001.pdf]

SUPPORTING INFORMATION

N-Protonated Acridinium Catalyst Enables Anti-Markovnikov Hydration of Unconjugated Tri- and Disubstituted Olefins

Boris Alexander van der Worp,[1,2] Tobias Ritter[1]*

[1]Max-Planck-Institut für Kohlenforschung, Kaiser-Wilhelm Platz 1, D-45470 Mülheim an der Ruhr, Germany

[2]Institute of Organic Chemistry, RWTH Aachen University, Landoltweg 1, 52074 Aachen, Germany

*E-mail: ritter@kofo.mpg.de

TABLE OF CONTENTS

TABLE OF CONTENTS 1

MATERIALS AND METHODS 6

EXPERIMENTAL DATA 8

General procedure for the anti-Markovnikov hydration of olefins 8

Preparation of acridines and acridinium salts 8

9-(2-Chlorophenyl)acridine (**1**) 8

9-(2,4,6-Trichlorophenyl)acridine (**2**) 9

10-Phenyl-9-(2,4,6-trichlorophenyl)acridin-10-ium tetrafluoroborate (**3**$^+$$BF_4^-$) 10

3,6-Dimethyl-9-(2,4,6-trichlorophenyl)acridine (**4**) 10

9-(2-Chlorophenyl)acridin-10-ium tetrafluoroborate (**1**$H^+BF_4^-$) 12

9-(2,4,6-Trichlorophenyl)acridin-10-ium tetrafluoroborate (**2**$H^+BF_4^-$) 12

3,6-Dimethyl-9-(2,4,6-trichlorophenyl)acridin-10-ium tetrafluoroborate (**4**$H^+BF_4^-$) 13

Preparation of HAT catalysts 14

2,4,6-Triisopropylbenzenethiol (TRIPSH) 14

1,2-Bis(2,4,6-triisopropylphenyl)disulfane ($TRIP_2S_2$) 14

1,2-Bis(perfluorophenyl)disulfane ($(C_6F_5)_2S_2$) 15

2-Phenylmalononitrile 16

9-Phenyl-9H-fluorene 16

Preparation of olefins 18

Olefin **5a** 18

Olefin **5b** 18

Olefin **5c** 18

Olefin **5d** 19

Olefin **5h** 20

Olefin **5j** 20

Olefin **5k** 21

Olefin **5m** 22

Olefin **5n** 22

Olefin **5o** 23

Olefin **5p** 24

Olefin **5s** 24

Olefin **5t** 25

Olefin **10** 26

1-Pentyl-1-cyclopentene 26

Preparation of other compounds 27
2-((3-Methylbut-3-en-1-yl)oxy)ethan-1-ol 27
Reaction optimization and mechanistic investigations 28
Optimization of conditions for anti-Markovnikov hydration reaction 28
Role of excess acid in the reaction 30
Probing the regioisomeric ratio (r.r.) in the reaction with trisubstituted olefins 32
Comparative performance of acridinium catalysts in the hydration of olefins **5p** and **5s** 33
Anti-Markovnikov hydration reaction 35
Primary alcohol **6a** 35
Primary alcohol **6b** 36
Primary alcohol **6c** 36
Primary alcohol **6d** 37
Primary alcohol **6e** 38
Primary alcohol **6f** 39
Primary alcohol **6g** 40
Primary alcohol **6h** 40
Primary alcohol **6i** 41
Primary alcohol **6j** 42
Primary alcohol **6k** 43
Secondary alcohol **6l** 44
Primary alcohol **6m** 44
Primary alcohol **6n** 45
Primary alcohol **6o** 46
Primary alcohol **6p** 47
Secondary alcohol **6q** 48
Secondary alcohol **6r** 49
Secondary alcohol **6s** 50
Primary alcohol **6t** 51
Other anti-Markovnikov hydrofunctionalization reactions 52
Methyl ether **7** 52
Ethyl ether **8** 52
Isopropyl ether **9** 53
List of substrates that failed in anti-Markovnikov hydration reaction 54
Photochemical and electrochemical studies 55
Cyclic voltammograms of alkenes for oxidation potential series 55
Cyclic voltammograms of acridinium salts 62

Spectrophotometric titration of acridine **4** 66
Absorption and emission spectra of acridinium salts 69
Determination of excited-state reduction potentials of acridinium salts 71

DFT calculations 75

Alkene oxidation potentials series 75
Analysis of the hydration reaction with alkenes containing electron-rich aromatic rings 78
Cartesian coordinates and the Gibbs free energies of the DFT-optimized structures 81

SPECTROSCOPIC DATA 125

^{1}H NMR of acridine **2** 125
^{13}C NMR of acridine **2** 126
^{1}H NMR of acridinium **3**$^{+}BF_4^-$ 127
^{13}C NMR of acridinium **3**$^{+}BF_4^-$ 128
^{19}F NMR of acridinium **3**$^{+}BF_4^-$ 129
^{1}H NMR of acridine **4** 130
^{13}C NMR of acridine **4** 131
^{1}H NMR of acridinium **1**$H^+BF_4^-$ 132
^{13}C NMR of acridinium **1**$H^+BF_4^-$ 133
^{19}F NMR of acridinium **1**$H^+BF_4^-$ 134
^{1}H NMR of acridinium **2**$H^+BF_4^-$ 135
^{13}C NMR of acridinium **2**$H^+BF_4^-$ 136
^{19}F NMR of acridinium **2**$H^+BF_4^-$ 137
^{1}H NMR of acridinium **4**$H^+BF_4^-$ 138
^{13}C NMR of acridinium **4**$H^+BF_4^-$ 139
^{19}F NMR of acridinium **4**$H^+BF_4^-$ 140
^{1}H NMR of olefin **5c** 141
^{13}C NMR of olefin **5c** 142
^{19}F NMR of olefin **5c** 143
^{1}H NMR of olefin **5d** 144
^{13}C NMR of olefin **5d** 145
^{1}H NMR of olefin **5h** 146
^{13}C NMR of olefin **5h** 147
^{1}H NMR of olefin **5j** 148
^{13}C NMR of olefin **5j** 149
^{1}H NMR of olefin **5n** 150
^{13}C NMR of olefin **5n** 151
^{1}H NMR of 1-pentyl-1-cyclopentene 152
^{13}C NMR of 1-pentyl-1-cyclopentene 153

^{1}H NMR of 2-((3-methylbut-3-en-1-yl)oxy)ethan-1-ol 154
^{13}C NMR of 2-((3-methylbut-3-en-1-yl)oxy)ethan-1-ol 155
^{1}H NMR of primary alcohol **6a** 156
^{13}C NMR of primary alcohol **6a** 157
^{1}H NMR of primary alcohol **6b** 158
^{13}C NMR of primary alcohol **6b** 159
^{1}H NMR of primary alcohol **6c** 160
^{13}C NMR of primary alcohol **6c** 161
^{19}F NMR of primary alcohol **6c** 162
^{1}H NMR of primary alcohol **6d** 163
^{13}C NMR of primary alcohol **6d** 164
^{1}H NMR of primary alcohol **6e** 165
^{13}C NMR of primary alcohol **6e** 166
^{1}H NMR of primary alcohol **6f** 167
^{13}C NMR of primary alcohol **6f** 168
^{1}H NMR of primary alcohol **6g** 169
^{13}C NMR of primary alcohol **6g** 170
^{1}H NMR of primary alcohol **6h** 171
^{13}C NMR of primary alcohol **6h** 172
^{1}H NMR of primary alcohol **6i** 173
^{13}C NMR of primary alcohol **6i** 174
^{1}H NMR of primary alcohol **6j** 175
^{13}C NMR of primary alcohol **6j** 176
^{1}H NMR of primary alcohol **6k** 177
^{13}C NMR of primary alcohol **6k** 178
^{1}H NMR of secondary alcohol **6l** 179
^{13}C NMR of secondary alcohol **6l** 180
^{1}H NMR of primary alcohol **6m** 181
^{13}C NMR of primary alcohol **6m** 182
^{1}H NMR of primary alcohol **6n** (diastereoisomer 1) 183
^{13}C NMR of primary alcohol **6n** (diastereoisomer 1) 184
^{1}H NMR of primary alcohol **6n** (diastereoisomer 2) 185
^{13}C NMR of primary alcohol **6n** (diastereoisomer 2) 186
^{1}H NMR of primary alcohol **6o** 187
^{13}C NMR of primary alcohol **6o** 188
^{1}H NMR of primary alcohol **6p** 189
^{13}C NMR of primary alcohol **6p** 190
^{19}F NMR of primary alcohol **6p** 191

^{1}H NMR of secondary alcohol **6q** 192
^{13}C NMR of secondary alcohol **6q** 193
^{1}H NMR of tertiary alcohol **6q'** 194
^{13}C NMR of tertiary alcohol **6q'** 195
^{1}H NMR of secondary alcohol **6r** 196
^{13}C NMR of secondary alcohol **6r** 197
^{1}H NMR of secondary alcohol **6s** 198
^{13}C NMR of secondary alcohol **6s** 199
^{1}H NMR of tertiary alcohol **6s'** 200
^{13}C NMR of tertiary alcohol **6s'** 201
^{1}H NMR of primary alcohol **6t** 202
^{13}C NMR of primary alcohol **6t** 203
^{1}H NMR of methyl ether **7** 204
^{13}C NMR of methyl ether **7** 205
^{1}H NMR of ethyl ether **8** 206
^{13}C NMR of ethyl ether **8** 207
^{1}H NMR of isopropyl ether **9** 208
^{13}C NMR of isopropyl ether **9** 209

SUPPLEMENTARY REFERENCES 210

MATERIALS AND METHODS

All air- and moisture-insensitive reactions were carried out under ambient atmosphere and monitored by thin-layer chromatography (TLC). High-resolution mass spectra were obtained using *Q Exactive Plus* from *Thermo*. Concentration under reduced pressure was performed by rotary evaporation at 25–40°C at an appropriate pressure. Purified compounds were further dried under high vacuum (0.010–0.005 mbar). Yields refer to purified and spectroscopically pure compounds, unless otherwise stated.

Solvents

Acetonitrile was purchased from *Fisher Scientific*. Anhydrous solvents were obtained from *Phoenix Solvent Drying Systems*. All deuterated solvents were purchased from *Euriso-Top*.

Chromatography

Thin layer chromatography (TLC) was performed using EMD TLC plates pre-coated with 250 µm thickness silica gel 60 F254 plates and visualized by fluorescence quenching under 254 nm UV light or permanganate stain. Flash chromatography was performed using silica gel (40–63 µm particle size) purchased from *Geduran®*.

Electrochemistry

For analytical experiments, a BASI™ epsilon E2 device was used in combination with a BASI Cell Stand C3, purchased from *Bioanalytic systems Inc*. For cyclic voltammetry a MF-2013 electrode (99.95 % Pt, 1.6 mm diameter) from *Bioanalytic systems Inc*. was used as working electrode. Potentials were measured versus a Ag/AgCl/NaCl(aq) (3 M) reference electrode, MF-2052 electrode RE-5B form *Bioanalytic systems Inc*.

NMR Spectroscopy

NMR spectra were recorded on a Bruker AscendTM 500 spectrometer operating at 500 MHz, 470 MHz and 125 MHz, for ^{1}H, ^{19}F, and ^{13}C acquisitions, respectively; a Bruker AVANCE NEO 600 spectrometer equipped with a cryogenically cooled cryoBBO probe operating at 600 MHz, 565 MHz and 151 MHz for ^{1}H, ^{19}F, and ^{13}C acquisitions, respectively. Chemical shifts are reported in ppm with the solvent residual peak as the internal standard.[1] For ^{1}H NMR: $CDCl_3$, 7.26; $(CD_3)_2SO$, 2.50; CD_3CN, 1.94; for ^{13}C NMR: $CDCl_3$, 77.16; $(CD_3)_2SO$, 39.52, CD_3CN, 1.32; ^{19}F NMR spectra were referenced using a unified chemical shift scale based on the ^{1}H resonance of tetramethylsilane (1% v/v solution in the respective solvent).[2] Data is reported as follows: s = singlet, d = doublet, t = triplet, q = quartet, quin = quintet, sext = sextet, sept = septet, m = multiplet, bs = broad singlet; coupling constants in Hz.

Photoreactions

Photochemical reactions were performed using a Penn PhD integrated Photoreactor M2.

Starting materials

All substrates were used as received from commercial suppliers, or prepared according to published

procedures, respectively, unless otherwise stated. Alkenes were purchased from *Sigma-Aldrich*, *TCI*, *Alfa Aesar, BLDPharm, or ChemImpex*.

EXPERIMENTAL DATA

General procedure for the anti-Markovnikov hydration of olefins

To a 4-mL borosilicate vial equipped with a Teflon-coated magnetic stir bar were added an alkene **5** (0.500 mmol, 1.00 equiv.), acridine catalyst **4** (9.7 mg, 25 µmol, 5.0 mol%), TRIPSH (5.9 mg, 25 µmol, 5.0 mol%), and acetone/H_2O mixture (1.5 mL, 5/1 v/v, c = 0.33 M). Aqueous HBF_4 (48%, 6.5 µL, 9.1 mg, 50 µmol, 10 mol%) was introduced into the reaction mixture via a Hamilton syringe, resulting in a yellow coloration of the solution. The vial was sealed with a septum-cap, and a gentle stream of argon was passed through the solution via a needle (Φ 0.80 × 120 mm) for 2 minutes. The vial was then placed in a photoreactor equipped with 450nm LED module and irradiated for 18-36 hours at approximately 25 °C, maintained by an in-built cooling fan. Upon completion of the irradiation, the reaction mixture was poured into aqueous HBF_4 solution (0.1 M, 5 mL), and the resulting heterogeneous phase was extracted with dichloromethane (4 × 1 mL).The combined organic phases were dried over Na_2SO_4, filtered, and concentrated under reduced pressure. The residue was analyzed by ^1H NMR spectroscopy to determine regioselectivity and subsequently purified by flash column chromatography on silica gel.

Preparation of acridines and acridinium salts

9-(2-Chlorophenyl)acridine (1)

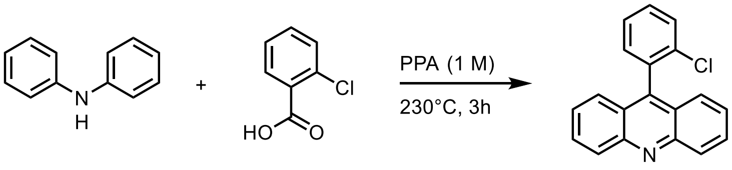

1

To a 4-mL borosilicate vial equipped with a Teflon-coated magnetic stir bar were added 2-chlorobenzoic acid (313 mg, 2.00 mmol, 1.00 equiv.), diphenylamine (440 mg, 2.60 mmol, 1.30 equiv.), and polyphosphoric acid (PPA, 4.1 g, 2.0 mL, c = 1.0 M). The vial was sealed with a septum cap, placed in a metal heating block, and heated at 230°C for 3 hours. Subsequently, the reaction mixture was cooled to 25 °C and diluted with water (2 mL). The resulting mixture was triturated with a spatula for 2 minutes to form a suspension, which was then poured into water (10 mL). Concentrated aqueous ammonia (30 wt.%) was added dropwise until the pH of the mixture reached 10. The heterogeneous phase was extracted with dichloromethane (5 × 5 mL). The combined organic phases were dried over Na_2SO_4, filtered, and concentrated under reduced pressure. The residue was purified by flash column chromatography on silica gel, eluting with toluene/EtOAc (40/1 v/v) + 0.5% Et_3N, to afford 234 mg of the title compound **1** as a beige solid (yield: 40%).

R_f = 0.17 (toluene/EtOAc (40/1 v/v) + 0.5% Et_3N, UV).

NMR Spectroscopy:

^1H NMR (500 MHz, $CDCl_3$, 25 ºC, δ): 8.30 (d, J = 8.8 Hz, 2H), 7.79 (ddd, J = 8.8, 6.4, 1.4 Hz, 2H), 7.67 (dd, J = 8.0, 1.4 Hz, 1H), 7.59 – 7.48 (m, 4H), 7.48 – 7.42 (m, 2H), 7.37 (dd, J = 7.5, 1.8 Hz, 1H).

^{13}**C NMR** (125 MHz, $CDCl_3$, 25 ºC, δ): 149.0, 144.2, 135.2, 134.4, 132.2, 130.2, 130.1, 130.0, 127.0, 126.4, 126.2, 125.1.

HRMS-EI(m/z) calc'd for $C_{19}H_{12}Cl_1N_1[M]^+$, 289.0653; found, 289.0657; deviation: –1.4 ppm.

The analytical data obtained is consistent with that reported in the literature.[3]

9-(2,4,6-Trichlorophenyl)acridine (2)

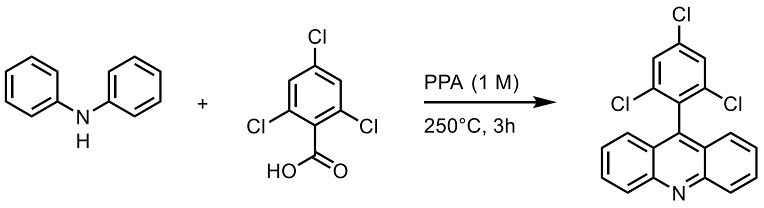

2

To a 4-mL borosilicate vial equipped with a Teflon-coated magnetic stir bar were added 2,4,6-trichlorobenzoic acid (451 mg, 2.00 mmol, 1.00 equiv.), diphenylamine (440 mg, 2.60 mmol, 1.30 equiv.), and polyphosphoric acid (PPA, 4.1 g, 2.0 mL, c = 1.0 M). The vial was sealed with a septum cap, placed in a metal heating block, and heated at 250°C for 3 hours. Subsequently, the reaction mixture was cooled to 25 °C and diluted with water (2 mL). The resulting mixture was triturated with a spatula for 2 minutes to form a suspension, which was poured into water (10 mL). Concentrated aqueous ammonia (30 wt.%) was added dropwise until the pH of the mixture reached 10. The heterogeneous phase was extracted with dichloromethane (5 × 5 mL). The combined organic phases were dried over Na_2SO_4, filtered, and concentrated under reduced pressure. The residue was purified by flash column chromatography on silica gel, eluting with toluene/MTBE (35/1 v/v) + 0.5% Et_3N, to afford 373 mg of the title compound **2** as a beige solid (yield: 52%). If purity of the isolated compound is insufficient, **2** can be efficiently recrystallized from acetonitrile.

R*f* = 0.27 (toluene/MTBE (35/1 v/v) + 0.5% Et_3N, UV).

NMR Spectroscopy:

^{1}**H NMR** (500 MHz, $CDCl_3$, 25 ºC, δ): 8.33 (d, *J* = 8.8 Hz, 2H), 7.84 – 7.78 (m, 2H), 7.63 (s, 2H), 7.54 – 7.43 (m, 4H).

^{13}**C NMR** (125 MHz, $CDCl_3$, 25 ºC, δ): 148.9, 140.3, 136.4, 135.8, 132.7, 130.3, 130.2, 128.5, 126.8, 125.0, 124.3.

HRMS-ESI(m/z) calc'd for $C_{19}H_{11}Cl_3N_1[M+H]^+$, 357.9952; found, 357.9953; deviation: 0.3 ppm.

10-Phenyl-9-(2,4,6-trichlorophenyl)acridin-10-ium tetrafluoroborate ($3^{+}BF_4^{-}$)

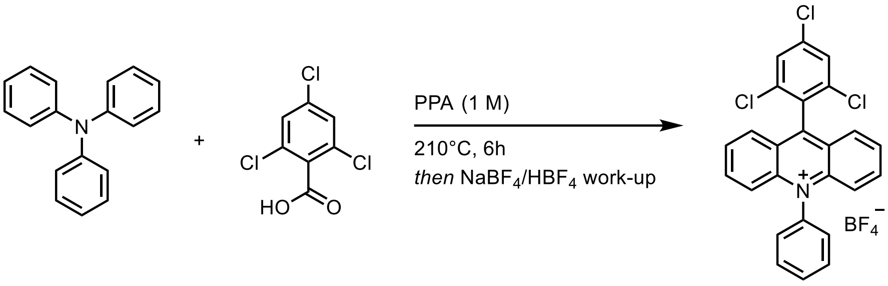

3^{+}BF$_4^{-}$

To a 4-mL borosilicate vial equipped with a Teflon-coated magnetic stir bar were added 2,4,6-trichlorobenzoic acid (451 mg, 2.00 mmol, 1.00 equiv.), triphenylamine (638 mg, 2.60 mmol, 1.30 equiv.), and polyphosphoric acid (PPA, 4.1 g, 2.0 mL, c = 1.0 M). The vial was sealed with a septum cap, placed in a metal heating block, and heated at 210°C for 6 hours. Subsequently, the reaction mixture was cooled to 25 °C and diluted with water (2 mL). The resulting mixture was triturated with a spatula for 2 minutes to form a suspension, which was poured into an aqueous $NaBF_4$ solution (10 wt.%, 10 mL). The suspension was sonicated for 5 minutes and placed in a refrigerator for 15 minutes. The resulting mixture was filtered on a Büchner funnel, and the solid residue was washed sequentially with aqueous HBF_4 solution (1 M, 3 mL) and diethyl ether (5 × 3 mL). The solid was collected and dried at 100°C under high vacuum for 24 hours to afford 219 mg of the title compound **3^{+}BF$_4^{-}$** as a bright-yellow solid (yield: 21%).

NMR Spectroscopy:

^{1}H NMR (600 MHz, $CDCl_3$, 25 ºC, δ): 8.23 (ddd, *J* = 9.2, 6.6, 1.5 Hz, 2H), 7.96 – 7.90 (m, 5H), 7.89 – 7.86 (m, 2H), 7.75 (s, 2H), 7.73 – 7.71 (m, 2H), 7.69 (d, *J* = 9.2 Hz, 2H).

^{13}C NMR (150 MHz, $CDCl_3$, 25 ºC, δ): 156.1, 142.3, 139.7, 138.5, 136.7, 135.6, 132.5, 131.9, 129.8, 129.6, 129.3, 127.9, 127.8, 125.5, 120.7.

^{19}F NMR (470 MHz, $CDCl_3$, 25 ºC, δ): –154.1.

HRMS-ESI(m/z) calc'd for $C_{25}H_{15}Cl_3N_1$ $[M]^+$, 434.0265; found, 434.0265; deviation: 0.0 ppm.

3,6-Dimethyl-9-(2,4,6-trichlorophenyl)acridine (4)

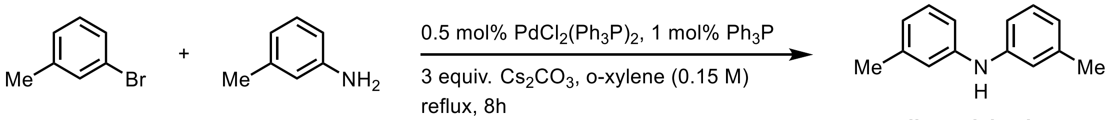

di-*m*-tolylamine

The compound was synthesized in two steps.

Step 1: Di-*m*-tolylamine was synthesized using a modified literature procedure.[4] To a 100-mL round-bottom flask equipped with a Teflon-coated magnetic stir bar and a reflux condenser were added $PdCl_2(Ph_3P)_2$ (17.6 mg, 25.0 µmol, 0.500 mol%), triphenylphosphine (Ph_3P, 13.1 mg, 50.0 µmol, 1.00 mol%), caesium carbonate (Cs_2CO_3, 4.89 g, 15.0 mmol, 3.00 equiv.), and *o*-xylene (33 mL, c = 0.15 M). The reaction vessel was

evacuated and backfilled with argon twice to remove dissolved oxygen. Subsequently, 1-bromo-3-methylbenzene (607 µL, 855 mg, 5.00 mmol, 1.00 equiv.) and *m*-toluidine (574 µL, 563 mg, 5.25 mmol, 1.05 equiv.) were added. The reaction flask was placed in a pre-heated oil bath, and the mixture was refluxed under an argon atmosphere for 8 hours. Subsequently, the reaction mixture was cooled to 25°C and poured into water (25 mL). The aqueous and organic phases were separated, and the aqueous layer was extracted with MTBE (3 × 10 mL). The combined organic layers were dried over Na_2SO_4, filtered, and concentrated under reduced pressure. The crude residue was used in the subsequent step without further purification.

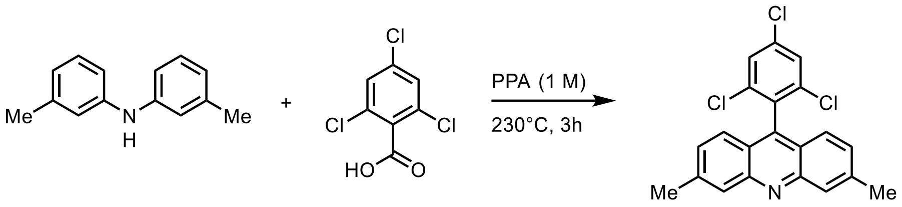

4

Step 2: Crude di-*m*-tolylamine obtained from *Step 1* (986 mg, 5.00 mmol, 1.00 equiv.), 2,4,6-trichlorobenzoic acid (1.13 g, 5.00 mmol, 1.00 equiv.), and polyphosphoric acid (PPA, 10.3 g, 5.00 mL, c = 1.00 M) were added to a 20-mL borosilicate vial equipped with a Teflon-coated magnetic stir bar. The vial was sealed with a cap, placed in a metal heating block, and heated at 230°C for 3 hours. Subsequently, the reaction mixture was cooled to 25 °C and diluted with water (10 mL). The resulting mixture was triturated with a spatula for 2 minutes to form a suspension, which was then poured into water (50 mL). Concentrated aqueous ammonia (30 wt.%) was added dropwise until the pH reached 10. The heterogeneous mixture was filtered on a Büchner funnel, and the solid residue was washed with water (2 × 5 mL). The solid was dried on the filter for 2 hours and subsequently purified by flash column chromatography on silica gel, eluting with toluene/MTBE (40/1 v/v) + 0.5% Et_3N. The collected fractions were concentrated under reduced pressure, and the resulting solid (pale-green to dark-green) was washed with pentane/acetone (5/1 v/v, 2 × 1 mL) to afford 697 mg of the title compound **4** as a beige solid (yield: 36%). If purity of the isolated compound is insufficient, **4** can be efficiently recrystallized from acetonitrile.

$\mathbf{R}_f$ = 0.20 (toluene/MTBE (40/1 v/v) + 0.5% Et_3N, UV).

NMR Spectroscopy:

^{1}**H NMR** (500 MHz, $CDCl_3$, 25 ºC, δ): 8.06 (s, 2H), 7.61 (s, 2H), 7.35 – 7.28 (m, 4H), 2.61 (s, 6H).

^{13}**C NMR** (125 MHz, $CDCl_3$, 25 ºC, δ): 149.3, 140.8, 139.9, 136.5, 135.7, 133.1, 129.3, 128.6, 128.4, 124.7, 122.5, 22.3.

HRMS-EI(m/z) calc'd for $C_{21}H_{14}Cl_3N_1$ $[M]^+$, 385.0186; found, 385.0190; deviation: 1.0 ppm.

9-(2-Chlorophenyl)acridin-10-ium tetrafluoroborate ($1H^+BF_4^-$)

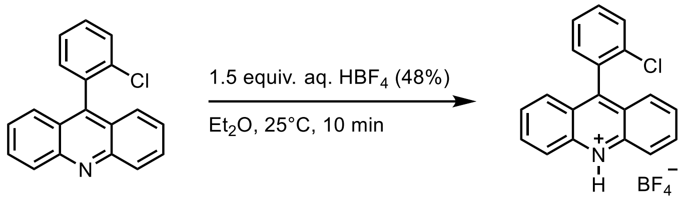

To a 4-mL borosilicate vial equipped with a Teflon-coated magnetic stir bar were added acridine **1** (14.5 mg, 50.0 µmol, 1.00 equiv.) and diethyl ether (1 mL). Aqueous HBF_4 (48%, 9.7 µL, 14 mg, 75 µmol, 1.5 equiv.) was introduced into the reaction mixture via a Hamilton syringe, causing the precipitation of a yellow solid. The mixture was stirred for an additional 10 minutes, after which stirring was stopped, and the ether supernatant was decanted. The resulting solid was washed with diethyl ether (2 × 1 mL), collected, and dried at 100°C under high vacuum for 24 hours to afford 18 mg of the title compound **1**$H^+BF_4^-$ as a bright-yellow solid (yield: 95%).

NMR Spectroscopy:

^{1}H NMR (500 MHz, $CDCl_3$, 25 ºC, δ): 8.63 (d, *J* = 8.9 Hz, 2H), 8.16 – 8.10 (m, 2H), 7.76 – 7.66 (m, 6H), 7.61 (td, *J* = 7.6, 0.9 Hz, 1H), 7.39 (dd, *J* = 7.6, 1.4 Hz, 1H).

^{13}C NMR (150 MHz, $CDCl_3$, 25 ºC, δ): 154.7, 141.9, 136.3, 133.6, 132.7, 131.8, 131.3, 130.6, 128.4, 127.5, 127.3, 125.4, 123.3.

^{19}F NMR (470 MHz, $CDCl_3$, 25 ºC, δ): –148.4.

HRMS-ESI(m/z) calc'd for $C_{19}H_{13}Cl_1N_1$ $[M]^+$, 290.0731; found, 290.0729; deviation: –0.7 ppm.

9-(2,4,6-Trichlorophenyl)acridin-10-ium tetrafluoroborate ($2H^+BF_4^-$)

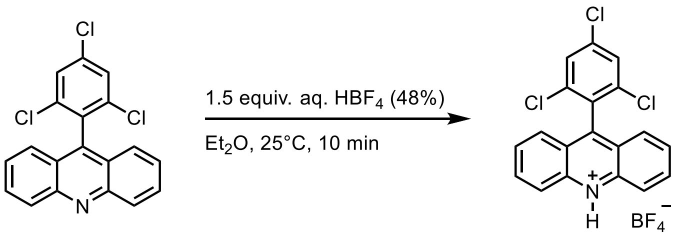

To a 4-mL borosilicate vial equipped with a Teflon-coated magnetic stir bar were added acridine **2** (17.9 mg, 50.0 µmol, 1.00 equiv.) and diethyl ether (1 mL). Aqueous HBF_4 (48%, 9.7 µL, 14 mg, 75 µmol, 1.5 equiv.) was introduced into the reaction mixture via a Hamilton syringe, leading to the precipitation of a yellow solid. The mixture was stirred for an additional 10 minutes, after which stirring was stopped, and the ether supernatant was decanted. The resulting solid was washed with diethyl ether (2 × 1 mL), collected, and dried at 100°C under high vacuum for 24 hours to afford 22 mg of the title compound **2**$H^+BF_4^-$ as a bright-yellow solid (yield: 99%).

NMR Spectroscopy:

^{1}H NMR (600 MHz, $CDCl_3$, 25 ºC, δ): 8.77 (d, *J* = 8.9 Hz, 1H), 8.26 (ddd, *J* = 8.9, 6.7, 1.2 Hz, 2H), 7.84 (ddd, *J* = 8.9, 6.7, 1.2 Hz, 2H), 7.74 – 7.70 (m, 4H).

^{13}C NMR (150 MHz, $CDCl_3$, 25 ºC, δ): 153.4, 140.2, 138.2, 138.1, 135.5, 129.6, 129.6, 129.3, 126.2, 125.1, 122.1.

^{19}F NMR (470 MHz, $CDCl_3$, 25 ºC, δ): –148.1.

HRMS-ESI(m/z) calc'd for $C_{19}H_{11}Cl_3N_1$ $[M]^+$, 357.9952; found, 357.9950; deviation: –0.6 ppm.

3,6-Dimethyl-9-(2,4,6-trichlorophenyl)acridin-10-ium tetrafluoroborate (4H$^+$BF$_4^-$)

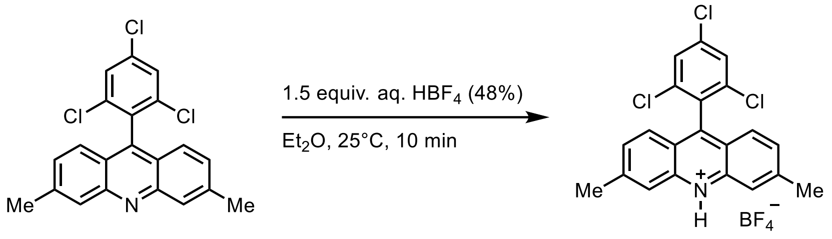

4 **4**H$^+$BF$_4^-$

To a 4-mL borosilicate vial equipped with a Teflon-coated magnetic stir bar were added acridine **4** (19.3 mg, 50.0 µmol, 1.00 equiv.) and diethyl ether (1 mL). Aqueous HBF_4 (48%, 9.7 µL, 14 mg, 75 µmol, 1.5 equiv.) was introduced into the reaction mixture via a Hamilton syringe, inducing the precipitation of a yellow solid. The mixture was stirred for an additional 10 minutes, after which stirring was stopped, and the ether supernatant was decanted. The solid was washed with diethyl ether (2 × 1 mL), collected, and dried at 100°C under high vacuum for 24 hours to afford 23 mg of the title compound **4**H$^+$BF$_4^-$ as a bright-yellow solid (yield: 97%).

NMR Spectroscopy:

^{1}H NMR (500 MHz, $CDCl_3$, 25 ºC, δ): 8.44 (s, 2H), 7.69 (s, 2H), 7.58 (d, *J* = 8.8 Hz, 2H), 7.53 (d, *J* = 8.8 Hz, 2H), 2.75 (s, 6H).

^{13}C NMR (125 MHz, $CDCl_3$, 25 ºC, δ): 151.5, 150.4, 140.7, 137.8, 135.5, 131.8, 130.0, 129.2, 125.8, 123.0, 120.5, 23.1.

^{19}F NMR (470 MHz, $CDCl_3$, 25 ºC, δ): –148.4.

HRMS-ESI(m/z) calc'd for $C_{21}H_{15}Cl_3N_1$ $[M]^+$, 386.0265; found, 386.0263; deviation: –0.5 ppm.

Preparation of HAT catalysts

2,4,6-Triisopropylbenzenethiol (TRIPSH)

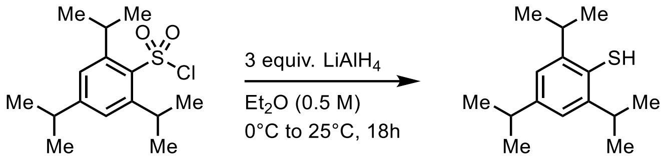

The compound was synthesized using a modified literature procedure.[5] To a dry 100-mL round-bottom flask equipped with a Teflon-coated magnetic stir bar was added lithium aluminum hydride ($LiAlH_4$, 759 mg, 20.0 mmol, 2.00 equiv.). Dry diethyl ether (Et_2O, 10.0 mL) was introduced to form a suspension, and the mixture was cooled to 0°C in an ice bath. A solution of 2,4,6-triisopropylbenzene-1-sulfonyl chloride (3.02 g, 10.0 mmol, 1.00 equiv.) in Et_2O (10.0 mL) was added slowly to the suspension. After the addition was complete, an additional portion of $LiAlH_4$ (380 mg, 10.0 mmol, 1.00 equiv.) was added. The reaction mixture was allowed to warm to 25°C and stirred for 18 hours. Subsequently, the reaction mixture was diluted with Et_2O (20 mL) and cooled to 0°C in an ice bath. Water (4.6 mL, 4 mL per gram of $LiAlH_4$) was added to the mixture, followed by aqueous NaOH solution (15 wt.%, 1.2 mL, 1 mL per gram of $LiAlH_4$). The mixture was stirred for 10 minutes at 0°C, followed by the addition of aqueous HCl (1 M, 50 mL). The reaction mixture was allowed to warm to 25°C over 1 hour. The layers were separated, and the aqueous layer was extracted with Et_2O (3 × 5 mL). The combined organic layers were dried over anhydrous Na_2SO_4, filtered, and concentrated under reduced pressure. The crude residue was purified by flash column chromatography on silica gel, eluting with pentane, to afford 2.34 g of the title compound **TRIPSH** as a colorless liquid (yield: 99%).

R*f* = 0.80 (pentane, $KMnO_4$).

NMR Spectroscopy:

^{1}H NMR (500 MHz, $CDCl_3$, 25 ºC, δ): 7.03 (s, 2H), 3.53 (hept, *J* = 6.9 Hz, 2H), 3.09 (s, 1H), 2.89 (hept, *J* = 6.9 Hz, 1H), 1.31 – 1.23 (m, 18H).

^{13}C NMR (125 MHz, $CDCl_3$, 25 ºC, δ): 148.2, 147.2, 124.4, 121.5, 34.3, 32.0, 24.2, 23.4.

HRMS-EI(m/z) calc'd for $C_{15}H_{24}S_1$ $[M]^+$, 236.1593; found, 236.1597; deviation: 1.3 ppm.

The analytical data obtained is consistent with that reported in the literature.[5]

1,2-Bis(2,4,6-triisopropylphenyl)disulfane (TRIP$_2$S$_2$)

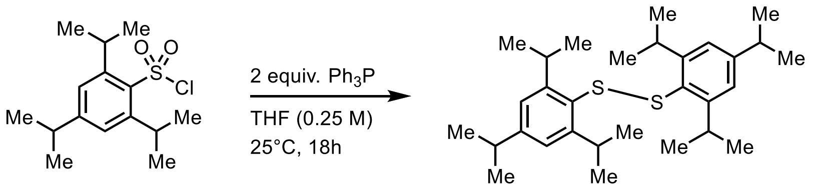

The compound was synthesized using a modified literature procedure.[6] To a dry 25-mL round-bottom flask equipped with a Teflon-coated magnetic stir bar was added triphenylphosphine (Ph_3P, 1.05 g, 4.00 mmol,

2.00 equiv.). A solution of 2,4,6-triisopropylbenzene-1-sulfonyl chloride (606 mg, 2.00 mmol, 1.00 equiv.) in dry tetrahydrofuran (THF, 8.0 mL) was added dropwise over 2 minutes. The flask was sealed with a septum, and the reaction mixture was stirred at 25 °C for 12 hours. Subsequently, the reaction mixture was concentrated under reduced pressure, and the resulting residue was dissolved in ethyl acetate (EtOAc, 4 mL). Hexane (40 mL) was added dropwise to the solution, inducing the precipitation of triphenylphosphine oxide, which was removed by filtration on a Büchner funnel. The filtrate was concentrated under reduced pressure, and the crude product was purified by flash column chromatography on silica gel, eluting with pentane, to afford 225 mg of the title compound **TRIP$_2$S$_2$** as a pale-yellow solid (yield: 48%).

R*f* = 0.85 (pentane, $KMnO_4$).

NMR Spectroscopy:

^{1}H NMR (500 MHz, $CDCl_3$, 25 ºC, δ): 6.92 (s, 4H), 3.54 (hept, *J* = 6.9 Hz, 4H), 2.83 (hept, *J* = 6.9 Hz, 2H), 1.20 (d, *J* = 6.9 Hz, 12H), 1.00 (d, *J* = 7.5 Hz, 24H).

^{13}C NMR (125 MHz, $CDCl_3$, 25 ºC, δ): 153.5, 150.7, 129.4, 121.9, 34.6, 31.5, 24.0.

HRMS-APPI(m/z) calc'd for $C_{30}H_{46}S_2$ $[M]^+$, 470.3035; found, 470.3037; deviation: 0.4 ppm.

The analytical data obtained is consistent with that reported in the literature.[6]

1,2-Bis(perfluorophenyl)disulfane (($C_6F_5)_2S_2$)

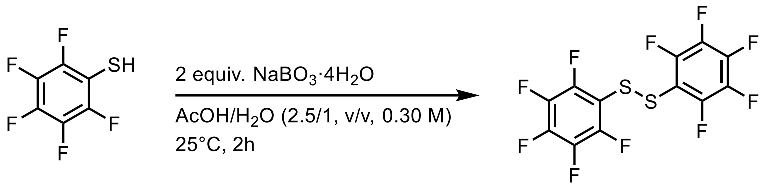

The compound was synthesized using a modified literature procedure.[7] Pentafluorobenzenethiol (600 mg, 3.00 mmol, 1.00 equiv.) was added to a solution of sodium perborate monohydrate ($NaBO_3 \cdot 4H_2O$, 923 mg, 6.00 mmol, 2.00 equiv.) in a mixture of acetic acid (AcOH, 7.5 mL) and water (3.0 mL) at 25 °C. The reaction mixture was stirred for 2 hours. After completion, the mixture was concentrated under reduced pressure and poured into saturated aqueous $NaHCO_3$ solution (10 mL). The resulting heterogeneous mixture was extracted with ethyl acetate (EtOAc, 3 × 5 mL). The combined organic layers were dried over anhydrous Na_2SO_4, filtered, and concentrated under reduced pressure. The residue was purified by flash column chromatography on silica gel, eluting with pentane, to afford 595 mg of the title compound **($C_6F_5)_2S_2$** as a pale-yellow solid (yield: 100%).

R*f* = 0.48 (pentane, UV).

NMR Spectroscopy:

^{13}C NMR (125 MHz, $CDCl_3$, 25 ºC, δ): 147.6 (dm, *J* = 250.1 Hz), 143.5 (dm, *J* = 259.9 Hz), 137.9 (dm, *J* = 256.9 Hz), 110.7 – 110.1 (m).

^{19}F NMR (470 MHz, $CDCl_3$, 25 ºC, δ): –131.15 – –131.36 (m), –147.60 – –147.89 (m), –159.23 – –159.50 (m).

HRMS-EI(m/z) calc'd for $C_{12}S_2F_{10}$ $[M]^+$, 397.9276; found, 397.9283; deviation: 1.8 ppm.

The analytical data obtained is consistent with that reported in the literature.[7]

2-Phenylmalononitrile

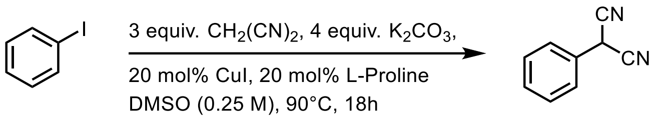

The compound was synthesized using a modified literature procedure.[8] To a dry 25-mL round-bottom flask equipped with a Teflon-coated magnetic stir bar were added copper(I) iodide (CuI, 76.2 mg, 0.400 mmol, 20.0 mol%), L-proline (46.1 mg, 0.400 mmol, 20.0 mol%), and potassium carbonate (K_2CO_3, 1.11 g, 8.00 mmol, 4.00 equiv.). The flask was sealed with a septum, evacuated, and backfilled with argon. Dimethyl sulfoxide (DMSO, 8.0 mL, c = 0.25 M) was added, followed by malononitrile (378 µL, 396 mg, 6.00 mmol, 3.00 equiv.) and iodobenzene (224 µL, 408 mg, 2.00 mmol, 1.00 equiv.). The reaction mixture was heated at 90°C in an oil bath for 18 hours. Subsequently, the mixture was cooled to 25 °C and aqueous HCl (1 M) was added dropwise until the pH reached 2–3. The resulting solution was extracted with ethyl acetate (EtOAc, 4 × 5 mL). The combined organic layers were dried over anhydrous sodium sulfate (Na_2SO_4), filtered, and concentrated under reduced pressure. The residue was purified by flash column chromatography on silica gel, eluting with cyclohexane/EtOAc (6/1 v/v), to afford 255 mg of the title compound **2-Phenylmalononitrile** as an off-white solid (yield: 90%).

R_f = 0.18 (cyclohexane/EtOAc (6/1 v/v), $KMnO_4$).

NMR Spectroscopy:

^{1}H NMR (500 MHz, $CDCl_3$, 25 ºC, δ): 7.53 – 7.49 (m, 5H), 5.08 (s, 1H).

^{13}C NMR (125 MHz, $CDCl_3$, 25 ºC, δ): 130.5, 130.2, 127.3, 126.3, 111.9, 28.2.

HRMS-EI(m/z) calc'd for $C_9H_6N_2$ $[M]^+$, 142.0525; found, 142.0527; deviation: 1.4 ppm.

The analytical data obtained is consistent with that reported in the literature.[8]

9-Phenyl-9H-fluorene

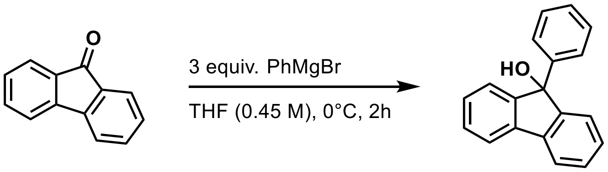

The compound was synthesized in two steps using a modified literature procedure.[9]

Step 1: To a dry 25-mL round-bottom flask equipped with a Teflon-coated magnetic stir bar and a reflux

condenser were added magnesium turnings (364 mg, 15.0 mmol, 3.00 equiv.) and anhydrous tetrahydrofuran (THF, 7.5 mL, c = 2.0 M). To activate the magnesium, 1,2-dibromoethane (65.0 µL, 141 mg, 0.750 mmol, 5.00 mol%) was added, followed by the dropwise addition of bromobenzene (1.58 mL, 2.36 g, 15.0 mmol, 3.00 equiv.). The reaction mixture was refluxed for 2 hours or until the magnesium turnings had completely dissolved. Subsequently, the mixture was cooled to 0°C in an ice bath, and a solution of 9-fluorenone (901 mg, 5.00 mmol, 1.00 equiv.) in anhydrous THF (3.5 mL) was added dropwise. After the addition was complete, the reaction mixture was allowed to warm to 25 °C and stirred for an additional 2 hours. The reaction mixture was slowly poured into saturated aqueous NH_4Cl solution (50 mL), and the resulting solution was extracted with dichloromethane (4 × 5 mL). The combined organic layers were dried over anhydrous Na_2SO_4, filtered, and concentrated under reduced pressure. The residue was used in the next step without further purification.

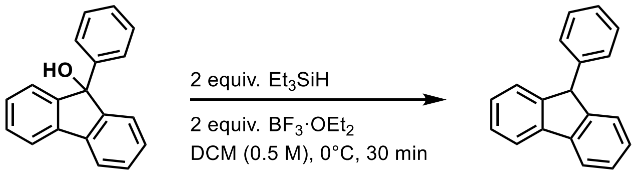

Step 2: The crude 9-phenyl-9H-fluoren-9-ol obtained in *Step 1* was dissolved in dichloromethane (10 mL, c = 0.5 M) and cooled to 0°C in an ice bath. Triethylsilane (Et_3SiH, 1.60 mL, 1.16 g, 10.0 mmol, 2.00 equiv.) and $BF_3{\cdot}OEt_2$ (1.23 mL, 1.42 g, 10.0 mmol, 2.00 equiv.) were added sequentially. The reaction mixture was stirred at 0°C for 30 minutes and then slowly poured into saturated aqueous $NaHCO_3$ solution (20 mL). The layers were separated, and the aqueous phase was extracted with dichloromethane (3 × 5 mL). The combined organic layers were dried over anhydrous Na_2SO_4, filtered, and concentrated under reduced pressure. The residue was purified by flash column chromatography on silica gel, eluting with cyclohexane, to afford 1.05 g of the title compound **9-Phenyl-9H-fluorene** as a yellow solid (yield: 87%).

R*f* = 0.17 (cyclohexane, UV).

NMR Spectroscopy:

^{1}H NMR (500 MHz, $CDCl_3$, 25 ºC, δ): 7.84 (d, *J* = 7.6 Hz, 2H), 7.42 (t, *J* = 7.3 Hz, 2H), 7.36 (d, *J* = 7.4 Hz, 2H), 7.33 – 7.24 (m, 5H), 7.15 – 7.11 (m, 2H), 5.09 (s, 1H).

^{13}C NMR (125 MHz, $CDCl_3$, 25 ºC, δ): 148.0, 141.7, 141.2, 128.8, 128.5, 127.4, 127.0, 125.5, 120.0, 54.6.

HRMS-EI(m/z) calc'd for $C_{19}H_{14}$ $[M]^+$, 242.1090; found, 242.1089; deviation: –0.4 ppm.

The analytical data obtained is consistent with that reported in the literature.[9]

Preparation of olefins

Olefin 5a

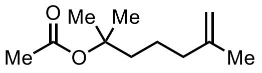

5a

This compound was generously provided to us by the Max-Planck-Institut für Kohlenforschung.

NMR Spectroscopy:

^{1}H NMR (500 MHz, $CDCl_3$, 25 ºC, δ): 4.66 (s, 1H), 4.63 (s, 1H), 1.96 (t, *J* = 7.6 Hz, 2H), 1.91 (s, 3H), 1.70 – 1.65 (m, 5H), 1.45 – 1.39 (m, 2H), 1.39 (s, 6H).

^{13}C NMR (125 MHz, $CDCl_3$, 25 ºC, δ): 170.4, 145.5, 110.1, 82.3, 40.4, 37.9, 26.1, 22.4, 22.3, 21.8.

HRMS-EI(m/z) calc'd for $C_{11}H_{20}O_2Na_1$ $[M+Na]^+$, 207.1355; found, 207.1356; deviation: 0.5 ppm.

The analytical data obtained is consistent with that reported in the literature.[10]

Olefin 5b

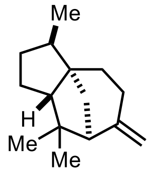

5b

This compound ((+)-*β*-Cedrene) was generously provided to us by the Max-Planck-Institut für Kohlenforschung.

NMR Spectroscopy:

^{1}H NMR (500 MHz, $CDCl_3$, 25 ºC, δ): 4.60 (t, *J* = 2.5 Hz, 1H), 4.53 (t, *J* = 2.5 Hz, 1H), 2.38 – 2.29 (m, 2H), 2.21 (d, *J* = 4.5 Hz, 1H), 1.93 – 1.86 (m, 1H), 1.86 – 1.76 (m, 2H), 1.75 – 1.67 (m, 1H), 1.62 – 1.28 (m, 5H), 1.22 (d, *J* = 11.4 Hz, 1H), 0.98 (s, 3H), 0.96 (s, 3H), 0.86 (d, *J* = 7.2 Hz, 3H).

^{13}C NMR (125 MHz, $CDCl_3$, 25 ºC, δ): 151.9, 107.8, 60.9, 56.6, 54.5, 45.2, 42.5, 42.3, 37.12, 33.8, 29.9, 26.76, 26.0, 25.9, 15.5.

HRMS-EI(m/z) calc'd for $C_{15}H_{24}$ $[M]^+$, 204.1873; found, 204.1875; deviation: 1.0 ppm.

The analytical data obtained is consistent with that reported in the literature.[11]

Olefin 5c

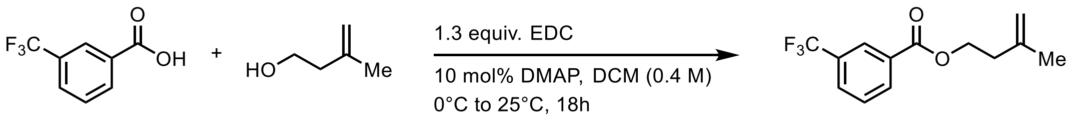

5c

The compound was synthesized using a modified literature procedure.[12] To a 20-mL borosilicate vial equipped with a Teflon-coated magnetic stir bar were added *N*-(3-dimethylaminopropyl)-*N*-ethylcarbodiimide hydrochloride (EDC, 623 mg, 3.25 mmol, 1.30 equiv.), 4-dimethylaminopyridine (DMAP, 30.5 mg, 0.250 mmol, 10.0 mol%), and dichloromethane (6.2 mL). The mixture was cooled to 0°C in an ice bath, and with stirring, 3-(trifluoromethyl)benzoic acid (475 mg, 2.50 mmol, 1.00 equiv.) was added, followed by 3-methylbut-3-en-1-ol (300 µL, 258 mg, 3.00 mmol, 1.20 equiv.). The reaction mixture was allowed to warm to 25°C and stirred for 18 hours. Subsequently, the mixture was washed with aqueous HCl solution (1 M, 3 mL), followed by saturated aqueous $NaHCO_3$ solution (3 mL). The organic layer was dried over anhydrous Na_2SO_4, filtered, and concentrated under reduced pressure. The residue was purified by flash column chromatography on silica gel, eluting with hexanes/EtOAc (20/1 v/v), to afford 600 mg of the title compound **5c** as a colorless liquid (yield: 93%).

R*f* = 0.47 (hexanes/EtOAc (20/1 v/v), $KMnO_4$).

NMR Spectroscopy:

^{1}H NMR (500 MHz, $CDCl_3$, 25 ºC, δ): 8.29 (s, 1H), 8.21 (d, *J* = 7.8 Hz, 1H), 7.80 (d, *J* = 7.8 Hz, 1H), 7.58 (t, *J* = 7.8 Hz, 1H), 4.85 (s, 1H), 4.81 (s, 1H), 4.47 (t, *J* = 6.8 Hz, 2H), 2.50 (t, *J* = 6.8 Hz, 2H), 1.81 (s, 3H).

^{13}C NMR (125 MHz, $CDCl_3$, 25 ºC, δ): 165.4, 141.6, 132.9, 131.4, 131.2 (q, *J* = 32.9 Hz), 129.5 (q, *J* = 3.6 Hz), 129.2, 126.6 (q, *J* = 3.8 Hz), 123.8 (q, *J* = 272.2 Hz), 112.8, 63.8, 36.9, 22.6.

^{19}F NMR (470 MHz, $CDCl_3$, 25 ºC, δ): –62.9.

HRMS-ESI(m/z) calc'd for $C_{13}H_{13}O_2F_3Na_1$ $[M+Na]^+$, 281.0760; found, 281.0759; deviation: –0.4 ppm.

Olefin 5d

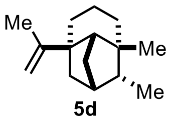

5d

This compound ((-)-Longifolene I2) was generously provided to us by the Max-Planck-Institut für Kohlenforschung.

NMR Spectroscopy:

^{1}H NMR (500 MHz, $CDCl_3$, 25 ºC, δ): 4.72 – 4.71 (m, 1H), 4.71 – 4.69 (m, 1H), 1.77 – 1.73 (m, 1H), 1.71 – 1.67 (m, 4H), 1.65 – 1.56 (m, 4H), 1.50 – 1.39 (m, 3H), 1.31 – 1.24 (m, 2H), 1.19 (td, *J* = 12.8, 4.0 Hz, 1H), 1.12 (td, *J* = 12.8, 4.0 Hz, 1H), 0.90 (s, 3H), 0.86 (d, *J* = 7.3 Hz, 3H).

^{13}C NMR (125 MHz, $CDCl_3$, 25 ºC, δ): 154.8, 108.0, 52.2, 46.8, 46.1, 41.8, 39.6, 38.7, 36.4, 34.8, 34.4, 25.8, 19.9, 18.4, 16.3.

HRMS-EI(m/z) calc'd for $C_{15}H_{24}$ $[M]^+$, 204.1873; found, 204.1871; deviation: –1.0 ppm.

Olefin 5h

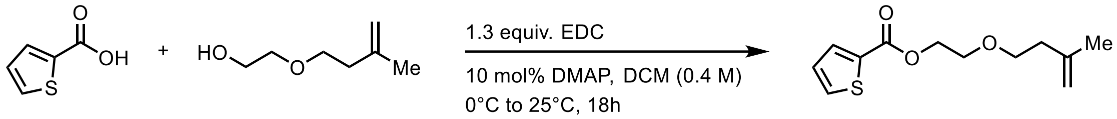

5h

The compound was synthesized using a modified literature procedure.[12] To a 20-mL borosilicate vial equipped with a Teflon-coated magnetic stir bar were added *N*-(3-dimethylaminopropyl)-*N*-ethylcarbodiimide hydrochloride (EDC, 249 mg, 1.30 mmol, 1.30 equiv.), 4-dimethylaminopyridine (DMAP, 18.3 mg, 0.150 mmol, 15.0 mol%), and dichloromethane (2.5 mL). The mixture was cooled to 0°C in an ice bath, and with stirring, thiophene-2-carboxylic acid (128 mg, 1.00 mmol, 1.00 equiv.) was added, followed by 2-((3-methylbut-3-en-1-yl)oxy)ethan-1-ol (156 mg, 1.20 mmol, 1.20 equiv.). The reaction mixture was allowed to warm to 25°C and stirred for 18 hours. Subsequently, the mixture was washed with aqueous HCl solution (1 M, 3 mL), followed by saturated aqueous $NaHCO_3$ solution (3 mL). The organic layer was dried over anhydrous Na_2SO_4, filtered, and concentrated under reduced pressure. The residue was purified by flash column chromatography on silica gel, eluting with hexanes/EtOAc (5/1 v/v), to afford 218 mg of the title compound **5h** as a colorless liquid (yield: 91%).

R*f* = 0.35 (hexanes/EtOAc (5/1 v/v), $KMnO_4$).

NMR Spectroscopy:

^{1}H NMR (500 MHz, $CDCl_3$, 25 ºC, δ): 7.82 (dd, *J* = 3.7, 1.3 Hz, 1H), 7.56 (dd, *J* = 4.9, 1.3 Hz, 1H), 7.10 (dd, *J* = 4.9, 3.7 Hz, 1H), 4.77 (s, 1H), 4.73 (s, 1H), 4.47 – 4.42 (m, 2H), 3.78 – 3.75 (m, 2H), 3.64 (t, *J* = 6.9 Hz, 2H), 2.32 (t, *J* = 6.9 Hz, 2H), 1.75 (s, 3H).

^{13}C NMR (125 MHz, $CDCl_3$, 25 ºC, δ): 162.3, 142.9, 133.8, 133.7, 132.6, 127.9, 111.7, 70.0, 68.7, 64.5, 37.8, 22.9.

HRMS-ESI(m/z) calc’d for $C_{12}H_{16}O_3Na_1S_1$ $[M+Na]^+$, 263.0712; found, 263.0712; deviation: 0.0 ppm.

Olefin 5j

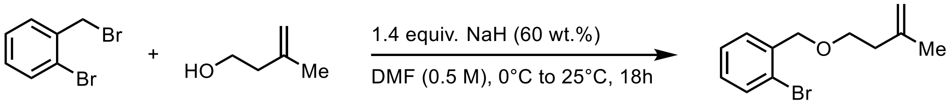

5j

To a 25-mL round-bottom flask equipped with a Teflon-coated magnetic stir bar was added NaH (60 wt.%, 168 mg, 4.20 mmol, 1.40 equiv.). The flask was evacuated, backfilled with argon twice, and sealed with a septum. Under an argon atmosphere, dry *N,N*-dimethylformamide (DMF, 6.0 mL) was added. The mixture was cooled to 0°C in an ice bath, and with stirring, 3-methylbut-3-en-1-ol (450 μL, 388 mg, 4.50 mmol, 1.50 equiv.) was added dropwise (caution: gas evolution). The reaction mixture was allowed to warm to 25 °C and stirred for 1 hour. The mixture was again cooled to 0°C in an ice bath, and with stirring, 1-bromo-2-(bromomethyl)benzene (750 mg, 3.00 mmol, 1.00 equiv.) was added. The reaction mixture was then allowed

to warm to 25 °C and stirred for 18 hours. Subsequently, the mixture was carefully poured into water (20 mL). The resulting heterogeneous phase was extracted with MTBE (3 × 10 mL). The combined organic layers were dried over anhydrous Na_2SO_4, filtered, and concentrated under reduced pressure. The residue was purified by flash column chromatography on silica gel, eluting with hexanes to hexanes/EtOAc (25/1 v/v), to afford 512 mg of the title compound **5j** as a colorless liquid (yield: 67%).

R*f* = 0.46 (hexanes/EtOAc (25/1 v/v), $KMnO_4$).

NMR Spectroscopy:

^{1}H NMR (500 MHz, $CDCl_3$, 25 ºC, δ): 7.53 (d, *J* = 7.9 Hz, 1H), 7.49 (d, *J* = 7.7 Hz, 1H), 7.31 (t, *J* = 7.6 Hz, 1H), 7.14 (td, *J* = 7.7, 1.8 Hz, 1H), 4.81 (s, 1H), 4.78 (s, 1H), 4.59 (s, 2H), 3.68 (td, *J* = 6.9, 1.2 Hz, 2H), 2.40 (t, *J* = 6.9 Hz, 2H), 1.78 (s, 3H).

^{13}C NMR (125 MHz, $CDCl_3$, 25 ºC, δ): 142.9, 138.0, 132.6, 129.1, 128.9, 127.5, 122.7, 111.7, 72.2, 69.5, 38.0, 22.9.

HRMS-EI(m/z) calc'd for $C_{12}H_{15}O_1Br_1$ $[M]^+$, 254.0301; found, 254.0302; deviation: 0.4 ppm.

Olefin 5k

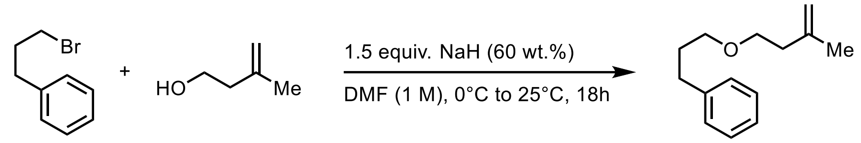

5k

To a 25-mL round-bottom flask equipped with a Teflon-coated magnetic stir bar was added NaH (60 wt.%, 600 mg, 15.0 mmol, 1.50 equiv.). The flask was evacuated, backfilled with argon twice, and sealed with a septum. Under an argon atmosphere, dry *N,N*-dimethylformamide (DMF, 10 mL) was added. The mixture was cooled to 0°C in an ice bath, and with stirring, 3-methylbut-3-en-1-ol (1.01 mL, 861 mg, 10.0 mmol, 1.00 equiv.) was added dropwise (caution: gas evolution). The reaction mixture was allowed to warm to 25 °C and stirred for 1 hour. The mixture was again cooled to 0°C in an ice bath, and with stirring, (3-bromopropyl)benzene (1.67 mL, 2.19 g, 11.0 mmol, 1.10 equiv.) was added dropwise. The reaction mixture was then allowed to warm to 25 °C and stirred for 18 hours. Subsequently, the mixture was carefully poured into water (20 mL). The resulting heterogeneous phase was extracted with MTBE (3 × 10 mL). The combined organic layers were dried over anhydrous Na_2SO_4, filtered, and concentrated under reduced pressure. The residue was purified by flash column chromatography on silica gel, eluting with hexanes to hexanes/EtOAc (30/1 v/v), to afford 1.09 g of the title compound **5k** as a colorless liquid (yield: 54%).

R*f* = 0.32 (hexanes/EtOAc (30/1 v/v), $KMnO_4$).

NMR Spectroscopy:

^{1}H NMR (500 MHz, $CDCl_3$, 25 ºC, δ): 7.31 – 7.27 (m, 2H), 7.23 – 7.17 (m, 3H), 4.80 (s, 1H), 4.75 (s, 1H), 3.54 (t, *J* = 7.0 Hz, 2H), 3.45 (t, *J* = 6.4 Hz, 2H), 2.70 (t, *J* = 7.7 Hz, 2H), 2.33 (t, *J* = 7.0 Hz, 2H), 1.96 –

1.87 (m, 2H), 1.78 (s, 3H).

^{13}C NMR (125 MHz, $CDCl_3$, 25 ºC, δ): 143.2, 142.2, 128.6, 128.4, 125.9, 111.5, 70.1, 69.5, 38.0, 32.5, 31.4, 22.9.

HRMS-ESI(m/z) calc'd for $C_{14}H_{20}O_1Na_1$ $[M+Na]^+$, 227.1406; found, 227.1407; deviation: 0.4 ppm.

The analytical data obtained is consistent with that reported in the literature.[13]

Olefin 5m

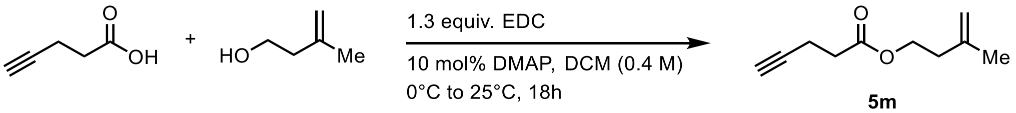

5m

The compound was synthesized using a modified literature procedure.[12] To a 20-mL borosilicate vial equipped with a Teflon-coated magnetic stir bar were added *N*-(3-dimethylaminopropyl)-*N*-ethylcarbodiimide hydrochloride (EDC, 498 mg, 2.60 mmol, 1.30 equiv.), 4-dimethylaminopyridine (DMAP, 36.7 mg, 0.300 mmol, 15.0 mol%), and dichloromethane (5.0 mL). The mixture was cooled to 0°C in an ice bath, and with stirring, pent-4-ynoic acid (196 mg, 2.00 mmol, 1.00 equiv.) was added, followed by 3-methylbut-3-en-1-ol (240 μL, 207 mg, 2.40 mmol, 1.20 equiv.). The reaction mixture was allowed to warm to 25°C and stirred for 18 hours. Subsequently, the mixture was washed with aqueous HCl solution (1 M, 3 mL), followed by saturated aqueous $NaHCO_3$ solution (3 mL). The organic layer was dried over anhydrous Na_2SO_4, filtered, and concentrated under reduced pressure. The residue was purified by flash column chromatography on silica gel, eluting with hexanes/EtOAc (10/1 v/v), to afford 300 mg of the title compound **5m** as a colorless liquid (yield: 90%).

R*f* = 0.39 (hexanes/EtOAc (10/1 v/v), $KMnO_4$).

NMR Spectroscopy:

^{1}H NMR (500 MHz, $CDCl_3$, 25 ºC, δ): 4.79 (s, 1H), 4.73 (s, 1H), 4.21 (t, *J* = 6.9 Hz, 2H), 2.56 – 2.51 (m, 2H), 2.51 – 2.46 (m, 2H), 2.34 (t, *J* = 6.9 Hz, 2H), 1.96 (t, *J* = 2.5 Hz, 1H), 1.75 (s, 3H).

^{13}C NMR (125 MHz, $CDCl_3$, 25 ºC, δ): 171.8, 141.7, 112.5, 82.6, 69.1, 63.0, 36.8, 33.5, 22.6, 14.5.

HRMS-ESI(m/z) calc'd for $C_{10}H_{14}O_2Na_1$ $[M+Na]^+$, 189.0886; found, 189.0886; deviation: 0.0 ppm.

The analytical data obtained is consistent with that reported in the literature.[14]

Olefin 5n

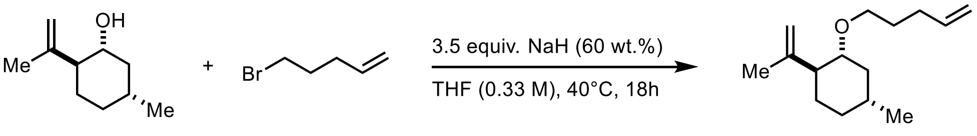

5n

To a 25-mL round-bottom flask equipped with a Teflon-coated magnetic stir bar was added NaH (60 wt.%,

560 mg, 14.0 mmol, 3.50 equiv.). The flask was evacuated, backfilled with argon twice, and sealed with a septum. Under an argon atmosphere, dry tetrahydrofuran (THF, 12.0 mL) was added. The mixture was cooled to 0°C in an ice bath, and with stirring, isopulegol (676 μL, 617 mg, 4.00 mmol, 1.00 equiv.) was added dropwise (caution: gas evolution). The reaction mixture was allowed to warm to 25 °C and stirred for 1 hour. 5-Bromopent-1-ene (1.42 mL, 1.79 g, 12.0 mmol, 3.00 equiv.) was added dropwise and the mixture was heated at 40°C in an oil bath for 18 hours under an argon atmosphere. Subsequently, the reaction mixture was cooled down to 25°C and carefully poured into water (20 mL). The resulting heterogeneous phase was extracted with MTBE (3 × 10 mL). The combined organic layers were dried over anhydrous Na_2SO_4, filtered, and concentrated under reduced pressure. The residue was purified by flash column chromatography on silica gel, eluting with hexanes to hexanes/EtOAc (30/1 v/v), to afford 213 mg of the title compound **5n** as a colorless liquid (yield: 24%).

R*f* = 0.36 (hexanes/EtOAc (30/1 v/v), $KMnO_4$).

NMR Spectroscopy:

^{1}H NMR (500 MHz, $CDCl_3$, 25 ºC, δ): 5.86 – 5.75 (m, 1H), 5.04 – 4.97 (m, 1H), 4.96 – 4.90 (m, 1H), 4.75 (s, 2H), 3.57 (dt, *J* = 9.2, 6.4 Hz, 1H), 3.28 (dt, *J* = 9.2, 6.4 Hz, 1H), 3.13 (td, *J* = 10.3, 4.1 Hz, 1H), 2.12 – 2.04 (m, 3H), 1.99 (ddd, *J* = 12.5, 10.3, 3.6 Hz, 1H), 1.72 (s, 3H), 1.67 – 1.55 (m, 4H), 1.47 – 1.37 (m, 1H), 1.37 – 1.27 (m, 1H), 0.97 – 0.84 (m, 5H).

^{13}C NMR (125 MHz, $CDCl_3$, 25 ºC, δ): 148.3, 138.8, 114.6, 110.8, 79.9, 68.0, 51.8, 40.5, 34.6, 31.7, 31.3, 30.5, 29.6, 22.5, 20.4.

HRMS-ESI(m/z) calc'd for $C_{15}H_{26}O_1Na_1$ $[M+Na]^+$, 245.1876; found, 245.1876; deviation: 0.0 ppm.

Olefin 5o

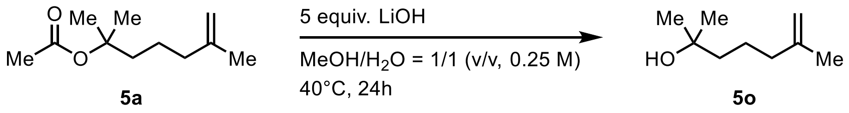

The compound was synthesized using a modified literature procedure.[15] To a 50-mL round-bottom flask equipped with a Teflon-coated magnetic stir bar were added olefin **5a** (921 mg, 5.00 mmol, 1.00 equiv.), LiOH (599 mg, 25.0 mmol, 5.00 equiv.), and a $MeOH/H_2O$ mixture (20 mL, 1/1 v/v, c = 0.25 M). The flask was sealed with a septum and the mixture was heated at 40°C in an oil bath for 24 hours. Subsequently, the reaction mixture was cooled down to 25°C and poured into saturated aqueous $NaHCO_3$ solution (10 mL). The resulting solution was extracted with MTBE (4 × 5 mL). The combined organic layers were dried over anhydrous Na_2SO_4, filtered, and concentrated under reduced pressure (100 mbar at 40°C as the product is volatile) to afford 682 mg of the title compound **5o** as a colorless liquid (yield: 96%).

NMR Spectroscopy:

^{1}H NMR (500 MHz, $CDCl_3$, 25 ºC, δ): 4.70 (s, 1H), 4.67 (s, 1H), 2.01 (t, *J* = 7.1 Hz, 2H), 1.70 (s, 3H), 1.53 – 1.40 (m, 5H), 1.20 (s, 6H).

^{13}**C NMR** (125 MHz, $CDCl_3$, 25 ºC, δ): 145.9, 110.1, 71.1, 43.6, 38.3, 29.4, 22.4, 22.4.

HRMS-ESI(m/z) calc'd for $C_{15}H_{26}O_1Na_1$ $[M+Na]^+$, 165.1250; found, 165.1251; deviation: 0.6 ppm.

The analytical data obtained is consistent with that reported in the literature.[16]

Olefin 5p

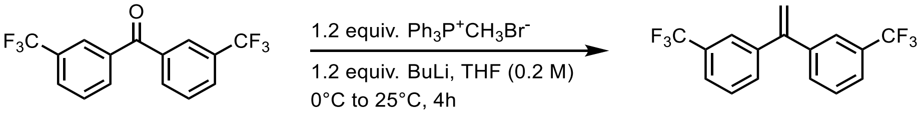

5p

To a 50-mL round-bottom flask equipped with a Teflon-coated magnetic stir bar was added methyltriphenylphosphonium bromide ($Ph_3P^+CH_3Br^-$, 1.29 g, 3.60 mmol, 1.20 equiv.). The flask was evacuated, backfilled with argon twice, and sealed with a septum. Under an argon atmosphere, dry tetrahydrofuran (THF, 15 mL) was added, forming a suspension. The mixture was cooled to 0°C in an ice bath and a solution of *n*-butyllithium (BuLi, 1.44 mL, 2.5 M in hexanes, 3.60 mmol, 1.20 equiv.) was added dropwise. The reaction mixture was allowed to warm to 25 °C and stirred for 1 hour. The solution was cooled down to 0°C in an ice bath again, and bis(trifluoromethyl)benzophenone (955 mg, 3.00 mmol, 1.00 equiv.) was added in one portion. The reaction mixture was allowed to warm to 25 °C and stirred for 3 hours. Subsequently, the reaction was quenched with water (5 mL) and poured into saturated aqueous $NaHCO_3$ solution (20 mL). The resulting solution was extracted with MTBE (4 × 5 mL). The combined organic layers were dried over anhydrous Na_2SO_4, filtered, and concentrated under reduced pressure. The residue was purified by flash column chromatography on silica gel, eluting with pentane, to afford 579 mg of the title compound **5p** as a colorless liquid (yield: 61%).

R*f* = 0.50 (pentane, UV).

NMR Spectroscopy:

^{1}**H NMR** (500 MHz, $CDCl_3$, 25 ºC, δ): 7.64 – 7.59 (m, 4H), 7.51 – 7.45 (m, 4H), 5.61 (s, 2H).

^{13}**C NMR** (125 MHz, $CDCl_3$, 25 ºC, δ): 147.9, 141.6, 131.6, 131.1 (q, J = 32.2 Hz), 129.1, 125.1 – 124.8 (m), 124.2 (q, J = 272.4 Hz), 117.3.

^{19}**F NMR** (470 MHz, $CDCl_3$, 25 ºC, δ): –62.7.

HRMS-EI(m/z) calc'd for $C_{16}H_{10}F_6$ $[M]^+$, 316.0681; found, 316.0685; deviation: 1.3 ppm.

The analytical data obtained is consistent with that reported in the literature.[17]

Olefin 5s

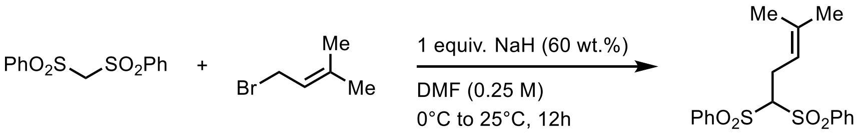

5s

The compound was synthesized using a modified literature procedure.[18] To a 25-mL round-bottom flask equipped with a Teflon-coated magnetic stir bar was added NaH (60 wt.% in mineral oil, 80.0 mg, 2.00 mmol, 1.00 equiv.). The flask was evacuated, backfilled with argon twice, and sealed with a septum. Under an argon atmosphere, dry *N,N*-dimethylformamide (DMF, 8.0 mL) was added, forming a suspension. The mixture was cooled down to 0°C in an ice bath, and bis(phenylsulfonyl)methane (593 mg, 2.00 mmol, 1.00 equiv.) was added in three portions (caution: gas evolution). The reaction mixture was allowed to warm to 25 °C and stirred for 30 minutes. 1-Bromo-3-methyl-2-butene (231 µL, 298 mg, 2.00 mmol, 1.00 equiv.) was added, and the mixture was stirred at 25°C for 12 hours. Subsequently, the reaction was quenched by the dropwise addition of water (5 mL) and poured into saturated aqueous $NaHCO_3$ solution (10 mL). The resulting heterogeneous phase was extracted with dichloromethane (4 × 5 mL). The combined organic layers were dried over anhydrous sodium sulfate (Na_2SO_4), filtered, and concentrated under reduced pressure. The residue was purified by flash column chromatography on silica gel, eluting with cyclohexane/MTBE (2/1 v/v), to afford 597 mg of the title compound **5s** as a colorless solid (yield: 82%).

R*f* = 0.21 (cyclohexane/MTBE (2/1, v/v), $KMnO_4$).

NMR Spectroscopy:

^{1}H NMR (500 MHz, $CDCl_3$, 25 ºC, δ): 7.98 – 7.92 (m, 4H), 7.71 – 7.67 (m, 2H), 7.62 – 7.53 (m, 4H), 5.11 – 4.98 (m, 1H), 4.42 (t, *J* = 6.1 Hz, 1H), 2.87 (t, *J* = 7.1 Hz, 2H), 1.59 (s, 3H), 1.47 (s, 3H).

^{13}C NMR (125 MHz, $CDCl_3$, 25 ºC, δ): 138.3, 136.2, 134.7, 129.8, 129.2, 118.3, 84.3, 25.8, 24.9, 17.9.

HRMS-ESI(m/z) calc'd for $C_{18}H_{20}O_4S_2Na_1$ $[M+Na]^+$, 387.0695; found, 387.0693; deviation: –0.5 ppm.

The analytical data obtained is consistent with that reported in the literature.[19]

Olefin 5t

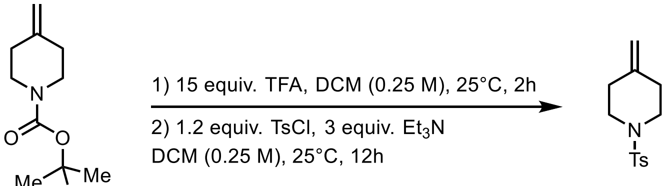

5t

The compound was synthesized using a modified literature procedure.[20] The compound was synthesized using a modified literature procedure. To a 25-mL round-bottom flask equipped with a Teflon-coated magnetic stir bar were added *tert.*-butyl 4-methylenepiperidine-1-carboxylate (592 mg, 3.00 mmol, 1.00 equiv.), dichloromethane (DCM, 12 mL, c = 0.25 M), and 2,2,2-trifluoroacetic acid (TFA, 3.45 mL, 5.13 g, 45.0 mmol, 15.0 equiv.). The reaction mixture was stirred at 25 °C for 2 hours and then concentrated under reduced pressure. The resulting slurry was re-dissolved in DCM (12 mL, c = 0.25 M), and to this solution were added 4-methylbenzene-1-sulfonyl chloride (TsCl, 686 mg, 3.60 mmol, 1.20 equiv.) and triethylamine (Et_3N, 1.25 mL, 911 mg, 9.00 mmol, 3.00 equiv.). The reaction mixture was stirred at 25°C for 12 hours. Subsequently, the mixture was poured into aqueous HCl solution (1 M, 30 mL). The layers were separated,

and the aqueous phase was extracted with DCM (4 × 5 mL). The combined organic layers were washed with saturated aqueous $NaHCO_3$ solution, dried over anhydrous Na_2SO_4, filtered, and concentrated under reduced pressure. The residue was triturated with hexanes (5 mL) for 1 minute, filtered, and dried under reduced pressure to afford 725 mg of the title compound **5t** as a colorless solid (yield: 96%).

NMR Spectroscopy:

^{1}**H NMR** (500 MHz, $CDCl_3$, 25 ºC, δ): 7.63 (d, *J* = 8.1 Hz, 2H), 7.31 (d, *J* = 8.1 Hz, 2H), 4.68 (s, 2H), 3.04 (t, *J* = 5.8 Hz, 4H), 2.42 (s, 3H), 2.29 (t, *J* = 5.8 Hz, 4H).

^{13}**C NMR** (125 MHz, $CDCl_3$, 25 ºC, δ): 143.6, 143.6, 133.6, 129.8, 127.8, 110.1, 47.8, 34.0, 21.7.

HRMS-ESI(m/z) calc'd for $C_{13}H_{17}O_2N_1S_1Na_1$ $[M+Na]^+$, 274.0872; found, 274.0871; deviation: –0.4 ppm.

The analytical data obtained is consistent with that reported in the literature.[21]

Olefin 10

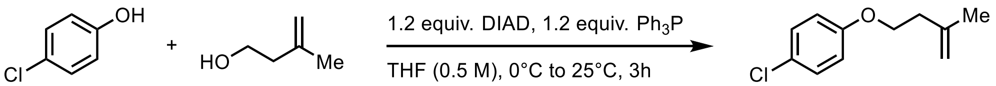

10

The compound was synthesized using a modified literature procedure.[22] To a 25-mL round-bottom flask equipped with a Teflon-coated magnetic stir bar were added 4-chlorophenol (386 mg, 3.00 mmol, 1.00 equiv.), triphenylphosphine (Ph_3P, 944 mg, 3.60 mmol, 1.20 equiv.), and dry tetrahydrofuran (THF, 6.0 mL, c = 0.50 M). 3-Methyl-3-buten-1-ol (361 μL, 310 mg, 3.60 mmol, 1.20 equiv.) was added, and the mixture was cooled to 0°C in an ice bath. Diisopropyl azodicarboxylate (DIAD, 709 μL, 728 mg, 3.60 mmol, 1.20 equiv.) was added dropwise to the reaction mixture. The mixture was allowed to warm to 25 °C and stirred for 3 hours. Subsequently, the reaction mixture was concentrated under reduced pressure. The residue was purified by flash column chromatography on silica gel, eluting with pentane/ethyl acetate (50/1 v/v), to afford 512 mg of the title compound **10** as a colorless liquid (yield: 87%).

$\mathbf{R}_f$ = 0.29 (pentane/EtOAc (50/1, v/v), $KMnO_4$).

NMR Spectroscopy:

^{1}**H NMR** (500 MHz, $CDCl_3$, 25 ºC, δ): 7.23 (d, *J* = 8.3 Hz, 2H), 6.84 (d, *J* = 8.3 Hz, 2H), 4.86 (s, 1H), 4.80 (s, 1H), 4.05 (t, *J* = 6.9 Hz, 2H), 2.50 (t, *J* = 6.9 Hz, 2H), 1.81 (s, 3H).

^{13}**C NMR** (125 MHz, $CDCl_3$, 25 ºC, δ): 157.7, 142.1, 129.4, 125.6, 116.0, 112.3, 66.9, 37.2, 22.9.

HRMS-EI(m/z) calc'd for $C_{11}H_{13}O_1Cl_1$ $[M]^+$, 196.0649; found, 196.0649; deviation: 0.0 ppm.

The analytical data obtained is consistent with that reported in the literature.[22]

1-Pentyl-1-cyclopentene

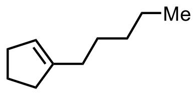

This compound was generously provided to us by the Max-Planck-Institut für Kohlenforschung.

NMR Spectroscopy:

^{1}H NMR (500 MHz, $CDCl_3$, 25 ºC, δ): 5.40 – 5.21 (m, 1H), 2.34 – 2.27 (m, 2H), 2.27 – 2.20 (m, 2H), 2.09 – 2.02 (m, 2H), 1.90 – 1.82 (m, 2H), 1.49 – 1.41 (m, 2H), 1.38 – 1.24 (m, 4H), 0.90 (t, *J* = 7.1 Hz, 3H).

^{13}C NMR (125 MHz, $CDCl_3$, 25 ºC, δ): 145.3, 123.1, 35.2, 32.6, 32.0, 31.3, 27.7, 23.6, 22.8, 14.2.

HRMS-EI(m/z) calc'd for $C_{10}H_{18}$ $[M]^+$, 138.1403; found, 138.1405; deviation: 1.4 ppm.

Preparation of other compounds

2-((3-Methylbut-3-en-1-yl)oxy)ethan-1-ol

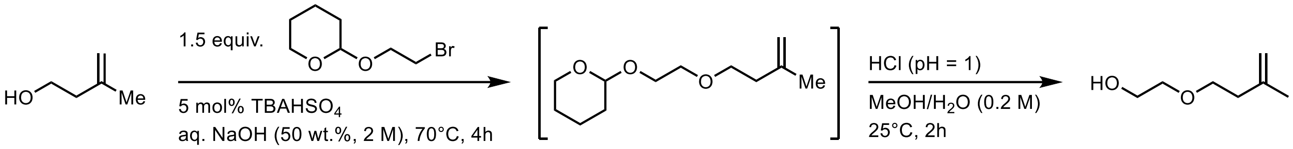

To a 10-mL round-bottom flask equipped with a Teflon-coated magnetic stir bar were added 2-(2-bromoethoxy)tetrahydro-2H-pyran (1.13 mL, 1.57 g, 7.50 mmol, 1.50 equiv.), 3-methyl-3-buten-1-ol (501 μL, 431 mg, 5.00 mmol, 1.00 equiv.), tetrabutylammonium hydrogen sulfate ($TBAHSO_4$, 84.9 mg, 0.250 mmol, 5.00 mol%), and aqueous NaOH solution (50 wt.%, 2.5 mL). The flask was sealed with a septum, and the mixture was heated at 70°C in an oil bath for 4 hours. Subsequently, the reaction mixture was cooled to 25 °C and transferred into a glass beaker containing water (15 mL). While stirring, concentrated hydrochloric acid (36 wt.%) was added dropwise until the pH reached 1. Methanol (10 mL) was then added to the solution, and the resulting mixture was stirred for 2 hours. Subsequently, the reaction mixture was extracted with diethyl ether (4 × 5 mL). The combined organic layers were dried over anhydrous Na_2SO_4, filtered, and concentrated under reduced pressure (500 mbar at 40°C, as the product is volatile). The residue was purified by flash column chromatography on silica gel, eluting with pentane/MTBE (3/2 v/v), to afford 323 mg of **2-((3-methylbut-3-en-1-yl)oxy)ethan-1-ol** as a colorless liquid (yield: 50%).

R_f = 0.14 (pentane/MTBE (3/2 v/v), $KMnO_4$).

NMR Spectroscopy:

^{1}H NMR (500 MHz, $CDCl_3$, 25 ºC, δ): 4.76 (s, 1H), 4.71 (s, 1H), 3.70 (t, *J* = 4.5 Hz, 2H), 3.58 (t, *J* = 7.0 Hz, 2H), 3.53 (t, *J* = 4.5 Hz, 2H), 2.42 (br s, 1H), 2.29 (t, *J* = 7.0 Hz, 2H), 1.73 (s, 3H).

^{13}C NMR (125 MHz, $CDCl_3$, 25 ºC, δ): 142.8, 111.7, 71.9, 69.6, 61.8, 37.8, 22.7.

HRMS-EI(m/z) calc'd for $C_7H_{14}O_2$ $[M]^+$, 130.0988; found, 130.0990; deviation: 1.5 ppm.

Reaction optimization and mechanistic investigations

Optimization of conditions for anti-Markovnikov hydration reaction

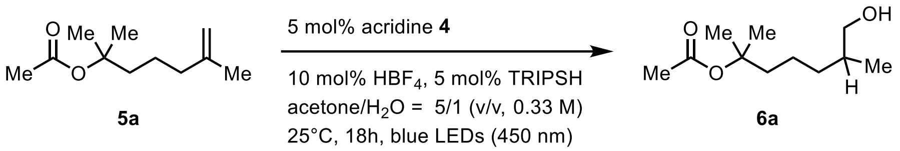

Optimization of the anti-Markovnikov hydration reaction was conducted on the model transformation of olefin **5a** to alcohol **6a**. Different photocatalysts, HAT catalysts, supplementary acids, solvents, and substrate concentrations were tested, varying one parameter at a time. The standard reaction procedure was as follows.

To a 1.5-mL borosilicate vial equipped with a Teflon-coated magnetic stir bar were added alkene **5a** (18.4 mg, 0.100 mmol, 1.00 equiv.), acridine catalyst **4** (1.9 mg, 5.0 µmol, 5.0 mol%), TRIPSH (1.2 mg, 5.0 µmol, 5.0 mol%), and an acetone/H_2O mixture (300 µL, 5/1 v/v, c = 0.330 M). Aqueous HBF_4 (1.0 M, 10 µL, 10 µmol, 10 mol%) was introduced into the reaction mixture via a Hamilton syringe, resulting in a yellow coloration of the solution. The vial was sealed with a septum cap, and a gentle stream of argon was passed through the solution via a needle (Φ 0.80 × 120 mm) for 30 seconds. The vial was placed in a photoreactor equipped with a 450 nm LED module and irradiated for 18 hours at approximately 25 °C, maintained by an in-built cooling fan. Upon completion of irradiation, the reaction mixture was concentrated under reduced pressure, and the residue was analyzed by ^{1}H NMR spectroscopy to determine reaction yield and conversion, using mesitylene as an internal standard.

Table S1. Screening of photocatalysts.

Entry	Photocatalyst	Yield **6a** (Conversion of **5a**)
1	Acridine **4** (5 mol%) + HBF_4 (10 mol%)	95% (100%)
2	Acridinium **3**$^+BF_4^-$ (5 mol%)	51% (71%)
3	Acridine **2** (5 mol%) + HBF_4 (10 mol%)	45% (54%)
4	Acridine **1** (5 mol%) + HBF_4 (10 mol%)	40% (57%)
5	Nicewicz catalyst (Acr_{Nic}, 5 mol%)	12% (24%)
6	Fukuzumi catalyst (Acr_{Fuk}, 5 mol%)	<5% (15%)

Table S2. Screening of HAT catalysts.

Entry	HAT catalyst	Yield **6a** (Conversion of **5a**)
1	TRIPSH (5 mol%)	95% (100%)
2	$TRIP_2S_2$ (2.5 mol%)	93% (100%)
3	[*p*-OMePh]$_2S_2$ (2.5 mol%)	92% (100%)

4	[*p*-Tol]$_2$S$_2$ (2.5 mol%)	86% (100%)
5	[C$_6$F$_5$]$_2$S$_2$ (2.5 mol%)	87% (100%)
6	2-Phenylmalononitrile (5 mol%)	83% (100%)
7	9-phenyl-9H-fluorene (5 mol%)	10% (34%)
8	*N*-Hydroxyphthalimide (5 mol%)	10% (28%)
9	TRIPSH (10 mol%)	93% (100%)
10	TRIPSH (20 mol%)	87% (100%)

Table S3. Screening of supplementary acids.

Entry	Acid	Yield **6a** (Conversion of **5a**)
1	HBF$_4$ (10 mol%)	95% (100%)
2	HPF$_6$ (10 mol%)	94% (100%)
3	Trifluoroacetic acid (TFA, 10 mol%)	89% (100%)
4	H$_3$PO$_4$ (10 mol%)	88% (100%)
5	Acetic acid (AcOH, 10 mol%)	79% (100%)

Table S4. Screening of solvents and concentrations.

Entry	Solvent	Yield **6a** (Conversion of **5a**)
1	Acetone/H_2O (5/1, v/v)	95% (100%)
2	MeCN/H_2O (5/1, v/v)	76% (100%)
3	DMF/H_2O (5/1, v/v)	10% (58%)
4	DMSO/H_2O (5/1, v/v)	16% (100%)
5	DCE/H_2O (5/1, v/v)	0% (31%)
6	PhF/H_2O (5/1, v/v)	0% (50%)
7	Sulfolane/H_2O (5/1, v/v)	82% (95%)
8	Methyl acetate/H_2O (5/1, v/v)	70% (100%)
9	H_2O	5% (46%)
10	Acetone/H_2O (1/1, v/v)	86% (100%)
11	Acetone/H_2O (10/1, v/v)	95% (100%)
12	Acetone/H_2O (20/1, v/v)	96% (100%)
13	Acetone/H_2O (5/1, v/v, 0.2 M)	96% (100%)
14	Acetone/H_2O (5/1, v/v, 0.1 M)	92% (100%)

Table S5. Control experiments.

Entry	Condition	Yield **6a** (Conversion of **5a**)
1	Standard conditions	95% (100%)
2	**4**$H^+BF_4^-$ (5 mol%), no acid	70% (100%)
3	No acridine **4**	0% (11%)
4	No TRIPSH	8% (37%)
5	No HBF_4	28% (43%)
6	No HBF_4, 10 mol% K_2CO_3	0% (40%)
7	No light	0% (10%)

Role of excess acid in the reaction

The standard reaction conditions for the anti-Markovnikov hydration of olefins require the use of 5 mol% acridine **4** and 10 mol% tetrafluoroboric acid (HBF_4). Control experiments confirmed that both acridine **4** and HBF_4 are necessary for the transformation (Table S5). However, it has been noted that replacing the acridine **4** + HBF_4 combination with 5 mol% of the preformed acridinium salt **4**$H^+BF_4^-$, a bench-stable solid, reduced the yield to 70% (vs. 95% under the standard conditions). This observation posed questions about the role of excess acid in the reaction.

The mechanism for the anti-Markovnikov hydration of olefins is shown in Figure S1. At least two mechanistic steps may be influenced by excess acid: (1) the acid-base equilibrium between acridine **4** and acridinium **4**H^+ and (2) protonation of thiolate in the HAT cycle to regenerate TRIPSH. Romero and Nicewicz previously reported that thiolate protonation could be the rate-limiting step in acridinium-mediated anti-Markovnikov hydroetherification.[23] If a similar rate limitation applies to this transformation, increasing the acidity of the reaction medium should affect product formation.

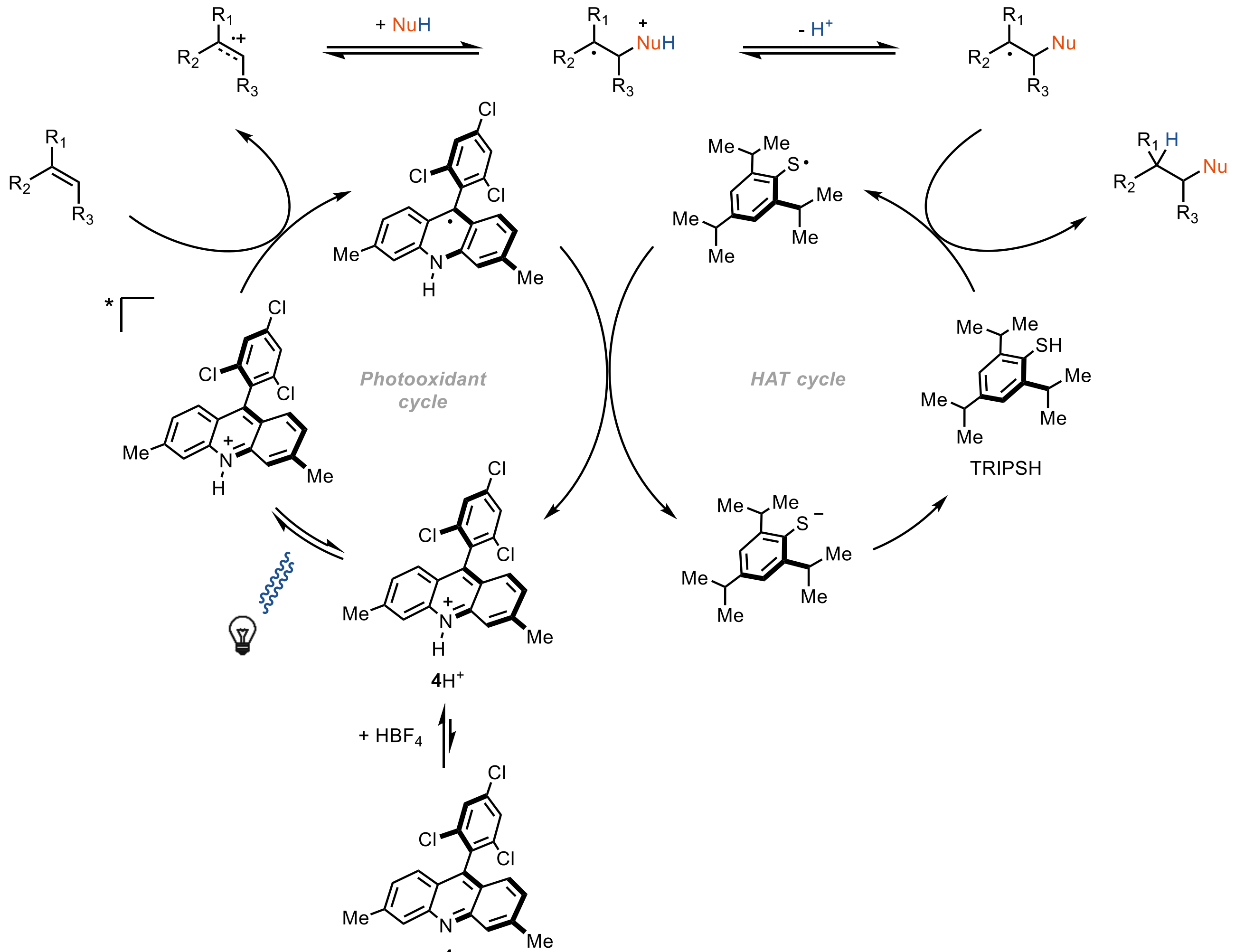

Figure S1. Proposed mechanism of the anti-Markovnikov hydration reaction.

To test this hypothesis, the model hydration of **5a** to **6a** was performed using 5 mol% acridinium **3**$^+$BF$_4^-$ in the absence and presence of additional HBF$_4$. The reaction procedure was as follows.

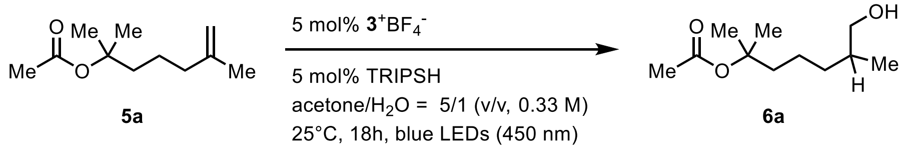

To a 1.5-mL borosilicate vial equipped with a Teflon-coated magnetic stir bar were added alkene **5a** (18.4 mg, 0.100 mmol, 1.00 equiv.), acridine catalyst **3**$^+$BF$_4^-$ (2.6 mg, 5.0 µmol, 5.0 mol%), TRIPSH (1.2 mg, 5.0 µmol, 5.0 mol%), and an acetone/H$_2$O mixture (300 µL, 5/1 v/v, c = 0.330 M). The vial was sealed with a septum cap, and a gentle stream of argon was passed through the solution via a needle (Φ 0.80 × 120 mm) for 30 seconds. The vial was placed in a photoreactor equipped with a 450 nm LED module and irradiated for 18 hours at approximately 25 °C, maintained by an in-built cooling fan. Upon completion of irradiation, the reaction mixture was concentrated under reduced pressure, and the residue was analyzed by ^{1}H NMR spectroscopy to determine reaction yield and conversion, using mesitylene as an internal standard.

Acridinium **3**$^+$BF$_4^-$ was chosen to eliminate the influence of the acid-base equilibrium between **4** and **4**H$^+$BF$_4^-$. No significant difference in yield was observed, suggesting that the effect of excess acid is primarily associated with the protonation equilibrium of acridine **4**.

Table S6. Control experiments with excess acid.

Entry	Photocatalyst	Yield **6a** (Conversion of **5a**)
1	Acridinium **3**$^+$BF$_4^-$ (5 mol%)	51% (71%)
2	Acridinium **3**$^+$BF$_4^-$ (5 mol%) + HBF$_4$ (5 mol%)	53% (77%)

Acridines are weak bases, with the pK$_a$ of acridine reported as 5.6 in water.[24] To determine the basicity of acridine **4** in an acetone/water = 5/1 (v/v) mixture, a spectrophotometric titration was conducted using HBF$_4$ as the acid (pages S66-S69). Analysis of the obtained titration curve yielded the value of pK$_a$ = 3.26.

Under the reaction conditions, 5 mol% of the acridinium catalyst is used which corresponds to a **4**H$^+$ concentration of 16.5 mM. Defining x as the concentration of dissociated acridinium and expressing K_a through concentrations, the quadratic equation can solved to determine x.

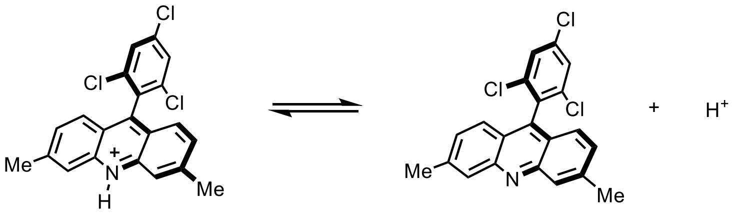

(0.0165 - x) (x) (x)

$$K_a = 5.5 \cdot 10^{-4} = \frac{x^2}{0.0165 - x}; \quad x = 0.00275$$

The use of 5 mol% **4**H$^+$BF$_4^-$ results in the dissociation of approximately 2.75 mM acridinium, leaving 13.75 mM (83%) in the protonated form. As the reaction proceeds, concurrent photocatalyst degradation reduces the acridinium concentration, further shifting the equilibrium toward dissociation. Applying the same calculation to the system with an additional 5 mol% HBF$_4$ (standard conditions) yields an equilibrium concentration of 15.99 mM for **4**H$^+$BF$_4^-$, which indicates that 97% of acridine remains protonated. Based on these data, we posit that excess acid serves to maintain the catalyst in its active form by ensuring near-complete protonation of acridine **4** in solution.

Probing the regioisomeric ratio (r.r.) in the reaction with trisubstituted olefins

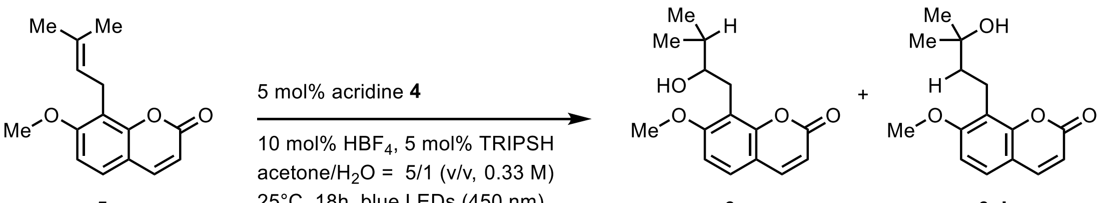

5q **6q** **6q'**

The developed anti-Markovnikov hydration reaction exhibited lower regioselectivity when applied to trisubstituted olefins compared to disubstituted analogues. To investigate whether the observed regioisomeric ratio (r.r.) was influenced by specific reaction parameters, a systematic screening of reaction conditions was conducted using the model transformation of olefin **5q** to alcohol **6q**. Different photocatalysts, HAT catalysts, and solvents, were tested, varying one parameter at a time. The standard reaction procedure was as follows.

To a 1.5-mL borosilicate vial equipped with a Teflon-coated magnetic stir bar were added alkene **5q** (24.4 mg, 0.100 mmol, 1.00 equiv.), acridine catalyst **4** (1.9 mg, 5.0 µmol, 5.0 mol%), TRIPSH (1.2 mg, 5.0 µmol, 5.0 mol%), and an acetone/H_2O mixture (300 µL, 5/1 v/v, c = 0.330 M). Aqueous HBF_4 (1.0 M, 10 µL, 10 µmol, 10 mol%) was introduced into the reaction mixture via a Hamilton syringe, resulting in a yellow coloration of the solution. The vial was sealed with a septum cap, and a gentle stream of argon was passed through the solution via a needle (Φ 0.80 × 120 mm) for 30 seconds. The vial was placed in a photoreactor equipped with a 450 nm LED module and irradiated for 18 hours at approximately 25 °C, maintained by an in-built cooling fan. Upon completion of irradiation, the reaction mixture was concentrated under reduced pressure, and the residue was analyzed by ^{1}H NMR spectroscopy to determine reaction yield and regioisomeric ratio, using mesitylene as an internal standard. The regioisomeric ratio (r.r.) was defined as the ratio of the anti-Markovnikov product (**6q**) to the Markovnikov product (**6q'**).

Table S7. Screening of conditions.

Entry	Condition	Yield **6q** (r.r.)
1	Standard conditions	93% (2.7/1)
2	$[C_6F_5]_2S_2$ (2.5 mol%)	90% (2.8/1)
3	2-Phenylmalononitrile (5 mol%)	25% (2.2/1)
4	Fukuzumi catalyst (Acr_{Fuk}, 5 mol%)	32% (2.7/1)
5	Nicewicz catalyst (Acr_{Nic}, 5 mol%)	93% (2.8/1)
6	MeCN/H_2O (5/1, v/v)	79% (2.9/1)
7	Sulfolane/H_2O (5/1, v/v)	13% (2.6/1)
8	Methyl acetate/H_2O (5/1, v/v)	35% (2.8/1)

Comparative performance of acridinium catalysts in the hydration of olefins 5p and 5s

Performance of the developed acridinium catalyst **4** on substrates beyond unconjugated 1,1-disubstituted olefins was evaluated by conducting the hydration reaction with styrene (**5p**) and a trisubstituted alkene (**5s**) under standard conditions. For each substrate, three catalysts were tested: the acridinium catalyst **4**$H^+BF_4^-$ (generated in-situ from acridine **4** and aq. HBF_4), the Nicewicz catalyst, and the Fukuzumi catalyst. Efficiency of each catalyst was assessed based on the yield of the respective product, as determined by ^{1}H NMR spectroscopy of the crude reaction mixture.

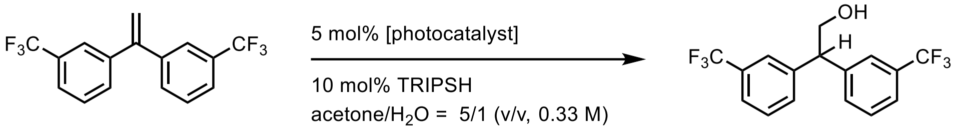

5p **6p**

To a 4-mL borosilicate vial equipped with a Teflon-coated magnetic stir bar were added alkene **5p** (158 mg, 0.500 mmol, 1.00 equiv.), the respective photocatalyst (25 µmol, 5.0 mol%), TRIPSH (11.8 mg, 50.0 µmol, 10.0 mol%), and acetone/H_2O mixture (1.5 mL, 5/1 v/v, c = 0.33 M). In case of acridine **4**, aqueous HBF_4 (48%, 6.5 µL, 9.1 mg, 50 µmol, 10 mol%) was introduced into the reaction mixture via a Hamilton syringe, resulting in a yellow coloration of the solution. The vial was sealed with a septum-cap, and a gentle stream of argon was passed through the solution via a needle (Φ 0.80 × 120 mm) for 2 minutes. The vial was then placed in the photoreactor equipped with 450nm LED module and irradiated for 18 hours at approximately 25 °C, maintained by an in-built cooling fan. Upon completion of the irradiation, the reaction mixture was poured into aqueous HBF_4 solution (0.1 M, 5 mL), and the resulting heterogeneous phase was extracted with dichloromethane (4 × 1 mL).The combined organic phases were dried over Na_2SO_4, filtered, and concentrated under reduced pressure. The residue was analyzed by ^{1}H NMR spectroscopy to determine reaction yield and conversion, using mesitylene as an internal standard.

Table S8. Preparation of alcohol **6p** with different photocatalysts.

Entry	Catalyst	Yield **6p** (Conversion of **5p**)
1	Acridine **4** (5 mol%) + HBF_4 (10 mol%)	84% (90%)
2	Nicewicz catalyst (Acr$_{Nic}$, 5 mol%)	29% (30%)
3	Fukuzumi catalyst (Acr$_{Fuk}$, 5 mol%)	7% (8%)

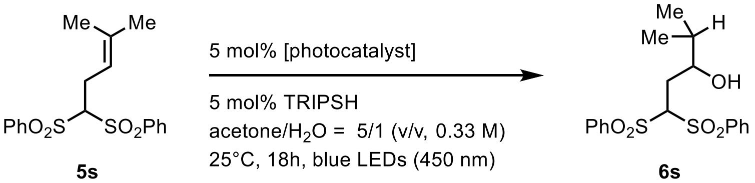

5s **6s**

To a 4-mL borosilicate vial equipped with a Teflon-coated magnetic stir bar were added alkene **5s** (182 mg, 0.500 mmol, 1.00 equiv.), the respective photocatalyst (25 µmol, 5.0 mol%), TRIPSH (5.9 mg, 25 µmol, 5.0 mol%), and acetone/H_2O mixture (1.5 mL, 5/1 v/v, c = 0.33 M). In case of acridine **4**, aqueous HBF_4 (48%, 6.5 µL, 9.1 mg, 50 µmol, 10 mol%) was introduced into the reaction mixture via a Hamilton syringe, resulting in a yellow coloration of the solution. The vial was sealed with a septum-cap, and a gentle stream of argon was passed through the solution via a needle (Φ 0.80 × 120 mm) for 2 minutes. The vial was then placed in the photoreactor equipped with 450nm LED module and irradiated for 18 hours at approximately 25 °C, maintained by an in-built cooling fan. Upon completion of the irradiation, the reaction mixture was poured into aqueous HBF_4 solution (0.1 M, 5 mL), and the resulting heterogeneous phase was extracted with dichloromethane (4 × 1 mL).The combined organic phases were dried over Na_2SO_4, filtered, and

concentrated under reduced pressure. The residue was analyzed by ^{1}H NMR spectroscopy to determine reaction yield and conversion, using mesitylene as an internal standard.

Table S9. Preparation of alcohol **6s** with different photocatalysts.

Entry	Catalyst	Yield **6s** (Conversion of **5s**)
1	Acridine **4** (5 mol%) + HBF_4 (10 mol%)	96% (100%)
2	Nicewicz catalyst (Acr_{Nic}, 5 mol%)	9% (10%)
3	Fukuzumi catalyst (Acr_{Fuk}, 5 mol%)	4% (7%)

Anti-Markovnikov hydration reaction

Primary alcohol 6a

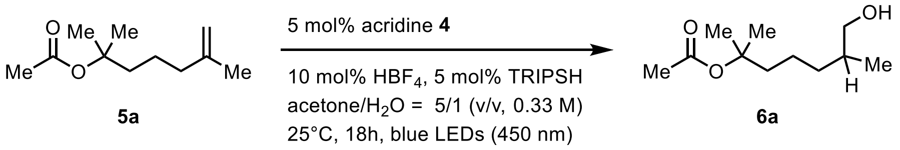

To a 4-mL borosilicate vial equipped with a Teflon-coated magnetic stir bar were added alkene **5a** (92.1 mg, 0.500 mmol, 1.00 equiv.), acridine catalyst **4** (9.7 mg, 25 µmol, 5.0 mol%), TRIPSH (5.9 mg, 25 µmol, 5.0 mol%), and acetone/H_2O mixture (1.5 mL, 5/1 v/v, c = 0.33 M). Aqueous HBF_4 (48%, 6.5 µL, 9.1 mg, 50 µmol, 10 mol%) was introduced into the reaction mixture via a Hamilton syringe, resulting in a yellow coloration of the solution. The vial was sealed with a septum-cap, and a gentle stream of argon was passed through the solution via a needle (Φ 0.80 × 120 mm) for 2 minutes. The vial was then placed in a photoreactor equipped with 450nm LED module and irradiated for 18 hours at approximately 25 °C, maintained by an in-built cooling fan. Upon completion of the irradiation, the reaction mixture was poured into aqueous HBF_4 solution (0.1 M, 5 mL), and the resulting heterogeneous phase was extracted with dichloromethane (4 × 1 mL).The combined organic phases were dried over Na_2SO_4, filtered, and concentrated under reduced pressure. The residue was analyzed by ^{1}H NMR spectroscopy to determine regioselectivity (>25:1 r.r.) and subsequently purified by flash column chromatography on silica gel, eluting with hexanes/MTBE (2/1 v/v), to afford 89 mg of alcohol **6a** as a colorless liquid (yield: 88%).

R_f = 0.12 (hexanes/MTBE (2/1 v/v), $KMnO_4$).

NMR Spectroscopy:

^{1}H NMR (500 MHz, $CDCl_3$, 25 ºC, δ): 3.46 (dd, *J* = 10.5, 5.8 Hz, 1H), 3.38 (dd, *J* = 10.5, 6.5 Hz, 1H), 1.93 (s, 3H), 1.79 (br s, 1H), 1.73 – 1.66 (m, 2H), 1.63 – 1.56 (m, 1H), 1.39 (s, 6H), 1.37 – 1.32 (m, 2H), 1.31 – 1.21 (m, 1H), 1.12 – 1.04 (m, 1H), 0.89 (d, *J* = 6.8 Hz, 3H).

^{13}C NMR (125 MHz, $CDCl_3$, 25 ºC, δ): 170.7, 82.5, 68.3, 41.0, 35.7, 33.4, 26.1, 26.1, 22.5, 21.3, 16.7.

HRMS-ESI(m/z) calc'd for $C_{11}H_{22}O_3Na_1$ $[M+Na]^+$, 225.1461; found, 225.1461; deviation: 0.0 ppm.

Primary alcohol 6b

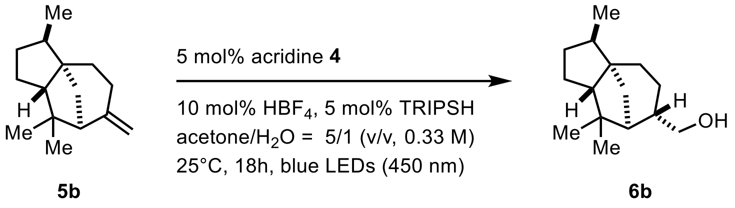

To a 4-mL borosilicate vial equipped with a Teflon-coated magnetic stir bar were added alkene **5b** (102 mg, 0.500 mmol, 1.00 equiv.), acridine catalyst **4** (9.7 mg, 25 µmol, 5.0 mol%), TRIPSH (5.9 mg, 25 µmol, 5.0 mol%), and acetone/H_2O mixture (1.5 mL, 5/1 v/v, c = 0.33 M). Aqueous HBF_4 (48%, 6.5 µL, 9.1 mg, 50 µmol, 10 mol%) was introduced into the reaction mixture via a Hamilton syringe, resulting in a yellow coloration of the solution. The vial was sealed with a septum-cap, and a gentle stream of argon was passed through the solution via a needle (Φ 0.80 × 120 mm) for 2 minutes. The vial was then placed in a photoreactor equipped with 450nm LED module and irradiated for 18 hours at approximately 25 °C, maintained by an in-built cooling fan. Upon completion of the irradiation, the reaction mixture was poured into aqueous HBF_4 solution (0.1 M, 5 mL), and the resulting heterogeneous phase was extracted with dichloromethane (4 × 1 mL). The combined organic phases were dried over Na_2SO_4, filtered, and concentrated under reduced pressure. The residue was analyzed by ^1H NMR spectroscopy to determine regioselectivity (>25:1 r.r.) and subsequently purified by flash column chromatography on silica gel, eluting with hexanes/EtOAc (10/1 v/v) +2% AcOH, to afford 90 mg of alcohol **6b** as a colorless liquid (yield: 81%).

$\mathbf{R}_{\boldsymbol{f}}$ = 0.17 (hexanes/EtOAc (10/1 v/v) +2% AcOH), $KMnO_4$).

NMR Spectroscopy:

^{1}H NMR (500 MHz, $CDCl_3$, 25 ºC, δ): 3.67 – 3.61 (m, 2H), 1.90 – 1.63 (m, 7H), 1.53 – 1.19 (m, 7H), 1.13 (d, *J* = 11.2, 1H), 1.07 (s, 3H), 0.95 (s, 3H), 0.84 (d, *J* = 7.1 Hz, 3H).

^{13}C NMR (125 MHz, $CDCl_3$, 25 ºC, δ): 67.8, 58.1, 55.0, 51.7, 47.6, 47.3, 43.8, 42.4, 37.2, 33.5, 28.8, 27.3, 25.8, 24.8, 15.5.

HRMS-EI(m/z) calc'd for $C_{15}H_{26}O_1$ $[M]^+$, 222.1978; found, 222.1979; deviation: 0.5 ppm.

The analytical data obtained is consistent with that reported in the literature.[25]

Primary alcohol 6c

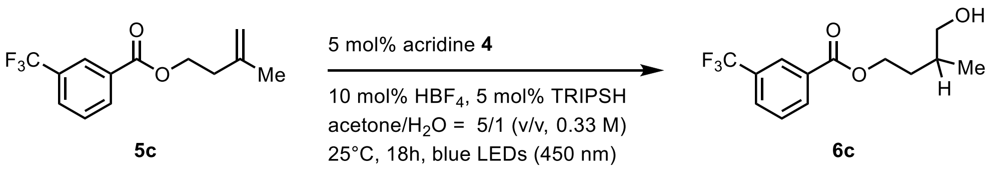

To a 4-mL borosilicate vial equipped with a Teflon-coated magnetic stir bar were added alkene **5c** (129.1 mg, 0.500 mmol, 1.00 equiv.), acridine catalyst **4** (9.7 mg, 25 µmol, 5.0 mol%), TRIPSH (5.9 mg, 25 µmol, 5.0 mol%), and acetone/H_2O mixture (1.5 mL, 5/1 v/v, c = 0.33 M). Aqueous HBF_4 (48%, 6.5 µL, 9.1 mg, 50

µmol, 10 mol%) was introduced into the reaction mixture via a Hamilton syringe, resulting in a yellow coloration of the solution. The vial was sealed with a septum-cap, and a gentle stream of argon was passed through the solution via a needle (Φ 0.80 × 120 mm) for 2 minutes. The vial was then placed in a photoreactor equipped with 450nm LED module and irradiated for 24 hours at approximately 25 °C, maintained by an in-built cooling fan. Upon completion of the irradiation, the reaction mixture was poured into aqueous HBF_4 solution (0.1 M, 5 mL), and the resulting heterogeneous phase was extracted with dichloromethane (4 × 1 mL). The combined organic phases were dried over Na_2SO_4, filtered, and concentrated under reduced pressure. The residue was analyzed by ^1H NMR spectroscopy to determine regioselectivity (>25:1 r.r.) and subsequently purified by flash column chromatography on silica gel, eluting with hexanes/MTBE (2/1 v/v), to afford 103 mg of alcohol **6c** as a colorless liquid (yield: 75%).

R*f* = 0.11 (hexanes/MTBE (2/1 v/v)), $KMnO_4$).

NMR Spectroscopy:

^{1}H NMR (500 MHz, $CDCl_3$, 25 ºC, δ): 8.28 (s, 1H), 8.21 (d, *J* = 7.7 Hz, 1H), 7.81 (d, *J* = 7.7 Hz, 1H), 7.58 (t, *J* = 7.7 Hz, 1H), 4.49 – 4.40 (m, 2H), 3.56 (d, *J* = 6.0 Hz, 2H), 2.01 – 1.93 (m, 1H), 1.91 – 1.81 (m, 1H), 1.68 – 1.56 (m, 2H), 1.02 (d, *J* = 6.8 Hz, 3H).

^{13}C NMR (125 MHz, $CDCl_3$, 25 ºC, δ): 165.5, 132.9, 131.3, 131.2 (q, *J* = 32.9 Hz), 129.6 (q, *J* = 3.6 Hz), 129.2, 126.6 (q, *J* = 3.9 Hz), 123.8 (q, *J* = 272.6 Hz), 68.0, 64.0, 33.1, 32.23, 16.6.

^{19}F NMR (470 MHz, $CDCl_3$, 25 ºC, δ): –62.8

HRMS-ESI(m/z) calc'd for $C_{13}H_{15}O_3F_3$ $[M+Na]^+$, 299.0865; found, 299.0863; deviation: –0.7 ppm.

Primary alcohol 6d

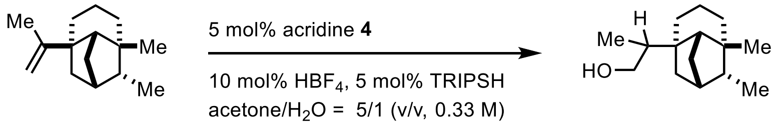

5d 6d

To a 4-mL borosilicate vial equipped with a Teflon-coated magnetic stir bar were added alkene **5d** (102 mg, 0.500 mmol, 1.00 equiv.), acridine catalyst **4** (9.7 mg, 25 µmol, 5.0 mol%), TRIPSH (5.9 mg, 25 µmol, 5.0 mol%), and acetone/H_2O mixture (1.5 mL, 5/1 v/v, c = 0.33 M). Aqueous HBF_4 (48%, 6.5 µL, 9.1 mg, 50 µmol, 10 mol%) was introduced into the reaction mixture via a Hamilton syringe, resulting in a yellow coloration of the solution. The vial was sealed with a septum-cap, and a gentle stream of argon was passed through the solution via a needle (Φ 0.80 × 120 mm) for 2 minutes. The vial was then placed in a photoreactor equipped with 450nm LED module and irradiated for 18 hours at approximately 25 °C, maintained by an in-built cooling fan. Upon completion of the irradiation, the reaction mixture was poured into aqueous HBF_4 solution (0.1 M, 5 mL), and the resulting heterogeneous phase was extracted with dichloromethane (4 × 1 mL). The combined organic phases were dried over Na_2SO_4, filtered, and concentrated under reduced pressure. The residue was analyzed by ^1H NMR spectroscopy to determine regioselectivity (>25:1 r.r.) and subsequently purified by flash column chromatography on silica gel, eluting

with hexanes/EtOAc (15/1 v/v) +2% AcOH, to afford 100 mg of alcohol **6d** as a colorless solid (yield: 90%, mixture of two diastereoisomers, 1.3:1 d.r.).

R*f* = 0.13 (hexanes/EtOAc (15/1 v/v) +2% AcOH, $KMnO_4$).

NMR Spectroscopy:

^{1}H NMR (500 MHz, $CDCl_3$, 25 ºC, δ): 3.79 (dd, *J* = 10.3, 3.8 Hz, 1H), 3.67 (dd, *J* = 10.2, 3.0 Hz, 1H), 3.37 (dd, *J* = 10.3, 8.7 Hz, 1H), 3.27 (dd, *J* = 10.2, 9.2 Hz, 1H), 1.73 (dd, *J* = 15.5, 3.3 Hz, 2H), 1.63 – 1.31 (m, 22H), 1.26 – 1.18 (m, 2H), 1.15 (dd, *J* = 12.5, 2.6 Hz, 1H), 1.09 – 0.99 (m, 3H), 0.94 (d, *J* = 6.9 Hz, 3H), 0.89 (d, *J* = 6.8 Hz, 3H), 0.86 – 0.80 (m, 12H).

^{13}C NMR (125 MHz, $CDCl_3$, 25 ºC, δ): 66.1, 65.6, 52.2, 51.8, 46.1, 45.9, 45.4, 44.4, 42.7, 42.4, 41.4, 41.4, 40.9, 40.1, 40.0, 39.7, 36.5, 36.1, 33.7, 33.1, 26.9, 26.5, 25.9, 25.8, 17.9, 17.7, 16.3, 16.3, 12.8, 11.9.

HRMS-ESI(m/z) calc'd for $C_{15}H_{26}O_1Na_1$ $[M+Na]^+$, 245.1876; found, 245.1876; deviation: 0.0 ppm.

Primary alcohol 6e

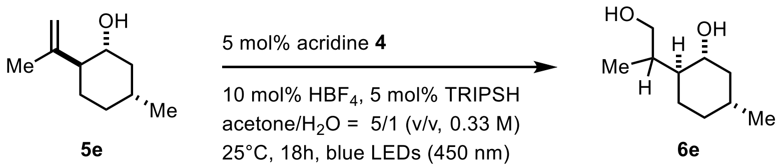

To a 4-mL borosilicate vial equipped with a Teflon-coated magnetic stir bar were added alkene **5e** (77.1 mg, 0.500 mmol, 1.00 equiv.), acridine catalyst **4** (9.7 mg, 25 µmol, 5.0 mol%), TRIPSH (5.9 mg, 25 µmol, 5.0 mol%), and acetone/H_2O mixture (1.5 mL, 5/1 v/v, c = 0.33 M). Aqueous HBF_4 (48%, 6.5 µL, 9.1 mg, 50 µmol, 10 mol%) was introduced into the reaction mixture via a Hamilton syringe, resulting in a yellow coloration of the solution. The vial was sealed with a septum-cap, and a gentle stream of argon was passed through the solution via a needle (Φ 0.80 × 120 mm) for 2 minutes. The vial was then placed in a photoreactor equipped with 450nm LED module and irradiated for 18 hours at approximately 25 °C, maintained by an in-built cooling fan. Upon completion of the irradiation, the reaction mixture was poured into aqueous HBF_4 solution (0.1 M, 5 mL), and the resulting heterogeneous phase was extracted with dichloromethane (4 × 1 mL). The combined organic phases were dried over Na_2SO_4, filtered, and concentrated under reduced pressure. The residue was analyzed by ^{1}H NMR spectroscopy to determine regioselectivity (>25:1 r.r.) and subsequently purified by flash column chromatography on silica gel, eluting with hexanes/EtOAc (2/1 v/v) +2% AcOH, to afford 36 mg of alcohol **6e** as a colorless solid (yield: 42%)

R*f* = 0.19 (hexanes/EtOAc (2/1 v/v) +2% AcOH, $KMnO_4$).

NMR Spectroscopy:

^{1}H NMR (500 MHz, $CDCl_3$, 25 ºC, δ): 3.91 (br s, 2H), 3.63 (dd, *J* = 10.7, 5.4 Hz, 1H), 3.56 (dd, *J* = 10.7, 3.3 Hz, 1H), 3.43 (td, *J* = 10.4, 4.3 Hz, 1H), 1.98 – 1.90 (m, 1H), 1.86 – 1.78 (m, 1H), 1.68 – 1.59 (m, 1H), 1.55 (dq, *J* = 13.3, 3.4 Hz, 1H), 1.46 – 1.37 (m, 1H), 1.37 – 1.31 (m, 1H), 1.27 – 1.16 (m, 1H), 1.01

– 0.82 (m, 8H).

^{13}C NMR (125 MHz, $CDCl_3$, 25 ºC, δ): 70.1, 67.2, 48.7, 44.6, 38.8, 34.7, 31.6, 29.7, 22.2, 12.1.

HRMS-EI(m/z) calc'd for $C_{10}H_{20}O_2$ $[M]^+$, 172.1458; found, 172.1457; deviation: –0.6 ppm.

The analytical data obtained is consistent with that reported in the literature.[26]

Primary alcohol 6f

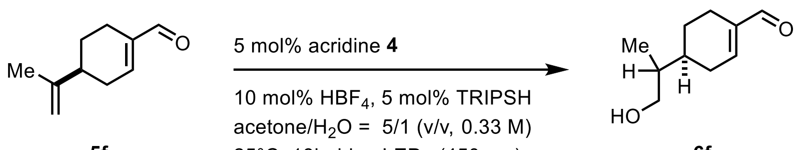

5f **6f**

To a 4-mL borosilicate vial equipped with a Teflon-coated magnetic stir bar were added alkene **5f** (75.1 mg, 0.500 mmol, 1.00 equiv.), acridine catalyst **4** (9.7 mg, 25 µmol, 5.0 mol%), TRIPSH (5.9 mg, 25 µmol, 5.0 mol%), and acetone/H_2O mixture (1.5 mL, 5/1 v/v, c = 0.33 M). Aqueous HBF_4 (48%, 6.5 µL, 9.1 mg, 50 µmol, 10 mol%) was introduced into the reaction mixture via a Hamilton syringe, resulting in a yellow coloration of the solution. The vial was sealed with a septum-cap, and a gentle stream of argon was passed through the solution via a needle (Φ 0.80 × 120 mm) for 2 minutes. The vial was then placed in a photoreactor equipped with 450nm LED module and irradiated for 18 hours at approximately 25 °C, maintained by an in-built cooling fan. Upon completion of the irradiation, the reaction mixture was poured into aqueous HBF_4 solution (0.1 M, 5 mL), and the resulting heterogeneous phase was extracted with dichloromethane (4 × 1 mL). The combined organic phases were dried over Na_2SO_4, filtered, and concentrated under reduced pressure. The residue was analyzed by ^{1}H NMR spectroscopy to determine regioselectivity (>25:1 r.r.) and subsequently purified by flash column chromatography on silica gel, eluting with MTBE/hexanes (3/2 v/v), to afford 66 mg of alcohol **6f** as a colorless liquid (yield: 76%, mixture of two diastereoisomers, 1:1 d.r.).

R_f = 0.15 (MTBE/hexanes (3/2 v/v), $KMnO_4$).

NMR Spectroscopy:

^{1}H NMR (500 MHz, $CDCl_3$, 25 ºC, δ): 9.39 (s, 2H), 6.80 – 6.78 (m, 2H), 3.67 – 3.59 (m, 2H), 3.57 – 3.50 (m, 2H), 2.48 – 2.31 (m, 4H), 2.20 – 1.97 (m, 4H), 1.90 – 1.79 (m, 2H), 1.77 – 1.56 (m, 6H), 1.33 – 1.13 (m, 2H), 0.93 (dd, J = 8.1, 6.9 Hz, 6H).

^{13}C NMR (125 MHz, $CDCl_3$, 25 ºC, δ): 194.2, 194.2, 151.4, 151.4, 141.6, 141.6, 66.0, 66.0, 39.8, 39.6, 35.2, 35.1, 31.1, 29.0, 25.8, 23.9, 22.0, 21.8, 13.5, 13.2.

HRMS-EI(m/z) calc'd for $C_{10}H_{16}O_2$ $[M]^+$, 168.1145; found, 168.1145; deviation: 0.0 ppm.

Primary alcohol 6g

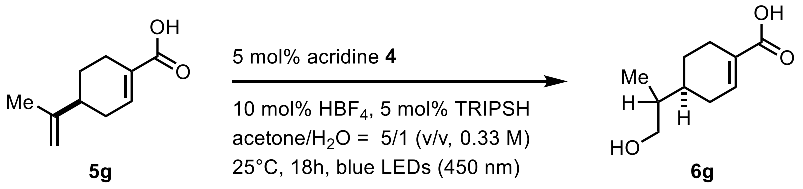

To a 4-mL borosilicate vial equipped with a Teflon-coated magnetic stir bar were added alkene **5g** (83.1 mg, 0.500 mmol, 1.00 equiv acridine catalyst **4** (9.7 mg, 25 µmol, 5.0 mol%), TRIPSH (5.9 mg, 25 µmol, 5.0 mol%), and acetone/H_2O mixture (1.5 mL, 5/1 v/v, c = 0.33 M). Aqueous HBF_4 (48%, 6.5 µL, 9.1 mg, 50 µmol, 10 mol%) was introduced into the reaction mixture via a Hamilton syringe, resulting in a yellow coloration of the solution. The vial was sealed with a septum-cap, and a gentle stream of argon was passed through the solution via a needle (Φ 0.80 × 120 mm) for 2 minutes. The vial was then placed in a photoreactor equipped with 450nm LED module and irradiated for 18 hours at approximately 25 °C, maintained by an in-built cooling fan. Upon completion of the irradiation, the reaction mixture was poured into aqueous HBF_4 solution (0.1 M, 5 mL), and the resulting heterogeneous phase was extracted with dichloromethane (4 × 1 mL). The combined organic phases were dried over Na_2SO_4, filtered, and concentrated under reduced pressure. The residue was analyzed by ^1H NMR spectroscopy to determine regioselectivity (>25:1 r.r.) and subsequently purified by flash column chromatography on silica gel, eluting with dichloromethane/EtOAc (5/1 v/v) + 2% AcOH, to afford 83 mg of alcohol **6g** as a colorless solid (yield: 90%, mixture of two diastereoisomers, 1:1 d.r.).

R*f* = 0.18 (dichloromethane/EtOAc (5/1 v/v) + 2% AcOH, $KMnO_4$).

NMR Spectroscopy:

^{1}H NMR (500 MHz, $CDCl_3$, 25 ºC, δ): 7.12 – 7.08 (m, 2H), 5.67 (br s, 2H), 3.68 – 3.62 (m, 2H), 3.58 – 3.52 (m, 2H), 2.50 – 2.43 (m, 2H), 2.33 – 2.12 (m, 4H), 2.10 – 1.94 (m, 2H), 1.88 – 1.78 (m, 2H), 1.71 – 1.54 (m, 4H), 1.38 – 1.17 (m, 2H), 0.94 (dd, *J* = 10.2, 6.6 Hz, 6H).

^{13}C NMR (125 MHz, $CDCl_3$, 25 ºC, δ): 172.4, 142.1, 142.1, 129.8, 129.8, 66.2, 66.1, 39.9, 39.7, 34.4, 34.4, 30.6, 28.6, 26.4, 24.6, 24.5, 13.6, 13.2.

HRMS-EI(m/z) calc'd for $C_{10}H_{15}O_3$ $[M]^+$, 183.1027; found, 183.1027; deviation: 0.0 ppm.

Primary alcohol 6h

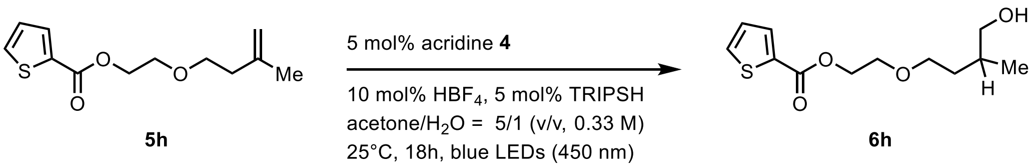

To a 4-mL borosilicate vial equipped with a Teflon-coated magnetic stir bar were added alkene **5h** (120 mg, 0.500 mmol, 1.00 equiv.), acridine catalyst **4** (9.7 mg, 25 µmol, 5.0 mol%), TRIPSH (5.9 mg, 25 µmol, 5.0 mol%), and acetone/H_2O mixture (1.5 mL, 5/1 v/v, c = 0.33 M). Aqueous HBF_4 (48%, 6.5 µL, 9.1 mg, 50

μmol, 10 mol%) was introduced into the reaction mixture via a Hamilton syringe, resulting in a yellow coloration of the solution. The vial was sealed with a septum-cap, and a gentle stream of argon was passed through the solution via a needle (Φ 0.80 × 120 mm) for 2 minutes. The vial was then placed in a photoreactor equipped with 450nm LED module and irradiated for 18 hours at approximately 25 °C, maintained by an in-built cooling fan. Upon completion of the irradiation, the reaction mixture was poured into aqueous HBF_4 solution (0.1 M, 5 mL), and the resulting heterogeneous phase was extracted with dichloromethane (4 × 1 mL). The combined organic phases were dried over Na_2SO_4, filtered, and concentrated under reduced pressure. The residue was analyzed by ^{1}H NMR spectroscopy to determine regioselectivity (>25:1 r.r.) and subsequently purified by flash column chromatography on silica gel, eluting with MTBE/hexanes (3/2 v/v), to afford 108 mg of alcohol **6h** as a colorless liquid (yield: 84%).

R*f* = 0.14 (MTBE/hexanes (3/2 v/v), $KMnO_4$).

NMR Spectroscopy:

^{1}H NMR (500 MHz, $CDCl_3$, 25 ºC, δ): 7.84 (dd, *J* = 3.8, 1.3 Hz, 1H), 7.55 (dd, *J* = 5.0, 1.3 Hz, 1H), 7.09 (dd, *J* = 5.0, 3.7 Hz, 1H), 4.48 – 4.38 (m, 2H), 3.74 (t, *J* = 4.9 Hz, 2H), 3.65 – 3.59 (m, 1H), 3.59 – 3.53 (m, 1H), 3.49 (dt, *J* = 9.9, 4.6 Hz, 1H), 3.45 – 3.39 (m, 1H), 2.52 (br s, 1H), 1.83 – 1.74 (m, 1H), 1.70 – 1.62 (m, 1H), 1.59 – 1.51 (m, 1H), 0.91 (d, *J* = 6.9 Hz, 3H).

^{13}C NMR (125 MHz, $CDCl_3$, 25 ºC, δ): 162.3, 133.8, 133.6, 132.7, 127.9, 69.8, 68.9, 68.0, 64.2, 34.2, 33.9, 17.3.

HRMS-CI(m/z) calc'd for $C_{12}H_{19}O_4S_1$ $[M+H]^+$, 259.0999; found, 259.0999; deviation: 0.0 ppm.

Primary alcohol 6i

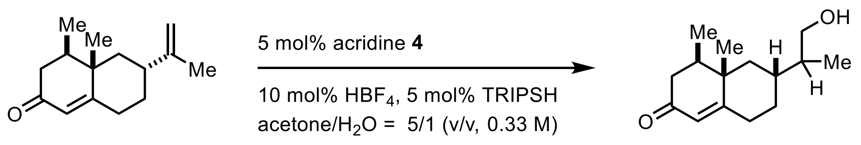

To a 4-mL borosilicate vial equipped with a Teflon-coated magnetic stir bar were added alkene **5i** (109 mg, 0.500 mmol, 1.00 equiv.), acridine catalyst **4** (9.7 mg, 25 μmol, 5.0 mol%), TRIPSH (5.9 mg, 25 μmol, 5.0 mol%), and acetone/H_2O mixture (1.5 mL, 5/1 v/v, c = 0.33 M). Aqueous HBF_4 (48%, 6.5 μL, 9.1 mg, 50 μmol, 10 mol%) was introduced into the reaction mixture via a Hamilton syringe, resulting in a yellow coloration of the solution. The vial was sealed with a septum-cap, and a gentle stream of argon was passed through the solution via a needle (Φ 0.80 × 120 mm) for 2 minutes. The vial was then placed in a photoreactor equipped with 450nm LED module and irradiated for 18 hours at approximately 25 °C, maintained by an in-built cooling fan. Upon completion of the irradiation, the reaction mixture was poured into aqueous HBF_4 solution (0.1 M, 5 mL), and the resulting heterogeneous phase was extracted with dichloromethane (4 × 1 mL). The combined organic phases were dried over Na_2SO_4, filtered, and concentrated under reduced pressure. The residue was analyzed by ^{1}H NMR spectroscopy to determine regioselectivity (>25:1 r.r.) and subsequently purified by flash column chromatography on silica gel, eluting

with MTBE/cyclohexane (2/1 v/v), to afford 103 mg of alcohol **6i** as a colorless liquid (yield: 87%, mixture of two diastereoisomers, 1:1 d.r.).

R*f* = 0.22 (MTBE/cyclohexane (2/1 v/v), $KMnO_4$).

NMR Spectroscopy:

> **^{1}H NMR** (500 MHz, $CDCl_3$, 25 ºC, δ): 5.73 (br s, 2H), 3.63 – 3.56 (m, 2H), 3.55 – 3.47 (m, 2H), 2.51 – 2.39 (m, 2H), 2.32 (ddd, *J* = 15.0, 4.2, 2.4 Hz, 2H), 2.30 – 2.16 (m, 4H), 2.01 – 1.92 (m, 2H), 1.91 – 1.77 (m, 8H), 1.57 – 1.49 (m, 2H), 1.25 – 1.00 (m, 10H), 0.94 (d, *J* = 6.9 Hz, 6H), 0.90 (d, *J* = 6.9 Hz, 3H), 0.87 (d, *J* = 7.0 Hz, 3H).

> **^{13}C NMR** (125 MHz, $CDCl_3$, 25 ºC, δ): 200.0, 171.6, 171.6, 124.5, 66.1, 65.8, 43.4, 42.1, 41.4, 40.6, 40.3, 40.3, 39.5, 39.3, 34.4, 33.9, 33.3, 33.2, 30.8, 28.4, 17.0, 17.0, 15.1, 15.0, 13.7, 13.1.

HRMS-EI(m/z) calc'd for $C_{15}H_{24}O_2$ $[M]^+$, 236.1771; found, 236.1772; deviation: 0.4 ppm.

The analytical data obtained is consistent with that reported in the literature.[27]

Primary alcohol 6j

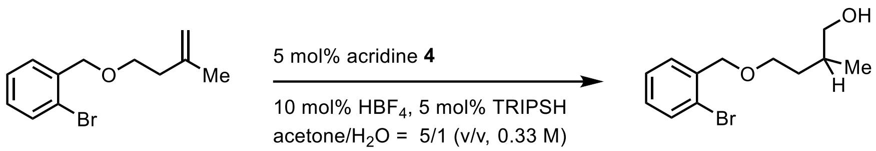

5j **6j**

To a 4-mL borosilicate vial equipped with a Teflon-coated magnetic stir bar were added alkene **5j** (128 mg, 0.500 mmol, 1.00 equiv.), acridine catalyst **4** (9.7 mg, 25 µmol, 5.0 mol%), TRIPSH (5.9 mg, 25 µmol, 5.0 mol%), and acetone/H_2O mixture (1.5 mL, 5/1 v/v, c = 0.33 M). Aqueous HBF_4 (48%, 6.5 µL, 9.1 mg, 50 µmol, 10 mol%) was introduced into the reaction mixture via a Hamilton syringe, resulting in a yellow coloration of the solution. The vial was sealed with a septum-cap, and a gentle stream of argon was passed through the solution via a needle (Φ 0.80 × 120 mm) for 2 minutes. The vial was then placed in a photoreactor equipped with 450nm LED module and irradiated for 18 hours at approximately 25 °C, maintained by an in-built cooling fan. Upon completion of the irradiation, the reaction mixture was poured into aqueous HBF_4 solution (0.1 M, 5 mL), and the resulting heterogeneous phase was extracted with dichloromethane (4 × 1 mL). The combined organic phases were dried over Na_2SO_4, filtered, and concentrated under reduced pressure. The residue was analyzed by ^{1}H NMR spectroscopy to determine regioselectivity (>25:1 r.r.) and subsequently purified by flash column chromatography on silica gel, eluting with cyclohexane/MTBE (3/1 v/v), to afford 106 mg of alcohol **6j** as a colorless liquid (yield: 78%).

R*f* = 0.16 (cyclohexane/MTBE (3/1 v/v), $KMnO_4$).

NMR Spectroscopy:

> **^{1}H NMR** (500 MHz, $CDCl_3$, 25 ºC, δ): 7.53 (dd, *J* = 7.9, 1.2 Hz, 1H), 7.45 (dd, *J* = 7.6, 1.7 Hz, 1H), 7.31 (td, *J* = 7.5, 1.3 Hz, 1H), 7.14 (td, *J* = 7.7, 1.8 Hz, 1H), 4.58 (s, 2H), 3.69 – 3.63 (m, 1H), 3.63 – 3.58 (m,

1H), 3.49 (qd, J = 10.8, 5.8 Hz, 2H), 2.22 (br s, 1H), 1.89 – 1.79 (m, 1H), 1.79 – 1.70 (m, 1H), 1.62 – 1.53 (m, 1H), 0.94 (d, J = 6.8 Hz, 3H).

^{13}C NMR (125 MHz, $CDCl_3$, 25 ºC, δ): 137.6, 132.7, 129.2, 129.1, 127.6, 122.9, 72.5, 69.3, 68.2, 34.0, 33.9, 17.2.

HRMS-ESI(m/z) calc'd for $C_{12}H_{17}O_2Br_1Na_1$ $[M+Na]^+$, 295.0304; found, 295.0302; deviation: –0.7 ppm.

Primary alcohol 6k

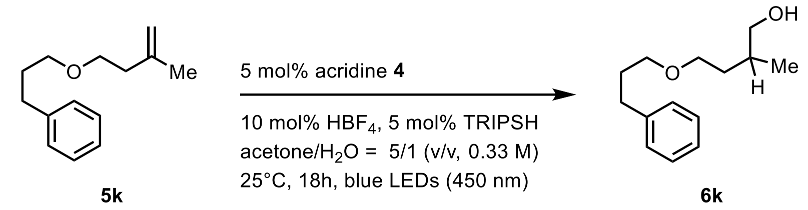

To a 4-mL borosilicate vial equipped with a Teflon-coated magnetic stir bar were added alkene **5k** (102 mg, 0.500 mmol, 1.00 equiv.), acridine catalyst **4** (9.7 mg, 25 µmol, 5.0 mol%), TRIPSH (5.9 mg, 25 µmol, 5.0 mol%), and acetone/H_2O mixture (1.5 mL, 5/1 v/v, c = 0.33 M). Aqueous HBF_4 (48%, 6.5 µL, 9.1 mg, 50 µmol, 10 mol%) was introduced into the reaction mixture via a Hamilton syringe, resulting in a yellow coloration of the solution. The vial was sealed with a septum-cap, and a gentle stream of argon was passed through the solution via a needle (Φ 0.80 × 120 mm) for 2 minutes. The vial was then placed in a photoreactor equipped with 450nm LED module and irradiated for 18 hours at approximately 25 °C, maintained by an in-built cooling fan. Upon completion of the irradiation, the reaction mixture was poured into aqueous HBF_4 solution (0.1 M, 5 mL), and the resulting heterogeneous phase was extracted with dichloromethane (4 × 1 mL). The combined organic phases were dried over Na_2SO_4, filtered, and concentrated under reduced pressure. The residue was analyzed by ^{1}H NMR spectroscopy to determine regioselectivity (>25:1 r.r.) and subsequently purified by flash column chromatography on silica gel, eluting with cyclohexane/MTBE (2/1 v/v), to afford 95 mg of alcohol **6k** as a colorless liquid (yield: 86%).

R*f* = 0.21 (cyclohexane/MTBE (2/1 v/v), $KMnO_4$).

NMR Spectroscopy:

^{1}H NMR (500 MHz, $CDCl_3$, 25 ºC, δ): 7.33 – 7.28 (m, 2H), 7.24 – 7.19 (m, 3H), 3.59 – 3.52 (m, 2H), 3.52 – 3.42 (m, 4H), 2.98 (br s, 1H), 2.71 (t, J = 7.5 Hz, 2H), 1.98 – 1.89 (m, 2H), 1.88 – 1.78 (m, 1H), 1.72 – 1.64 (m, 1H), 1.64 – 1.55 (m, 1H), 0.96 (d, J = 6.9 Hz, 3H).

^{13}C NMR (125 MHz, $CDCl_3$, 25 ºC, δ): 141.9, 128.6, 128.5, 125.9, 70.3, 69.4, 68.2, 34.5, 34.4, 32.4, 31.3, 17.4.

HRMS-ESI(m/z) calc'd for $C_{14}H_{12}O_2Na_1$ $[M+Na]^+$, 245.1512; found, 245.1511; deviation: –0.4 ppm.

Secondary alcohol 6l

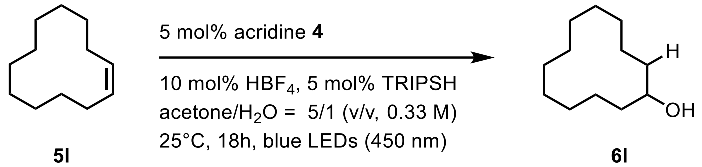

5l **6l**

To a 4-mL borosilicate vial equipped with a Teflon-coated magnetic stir bar were added alkene **5l** (83.2 mg, 0.500 mmol, 1.00 equiv.), acridine catalyst **4** (9.7 mg, 25 µmol, 5.0 mol%), TRIPSH (5.9 mg, 25 µmol, 5.0 mol%), and acetone/H_2O mixture (1.5 mL, 5/1 v/v, c = 0.33 M). Aqueous HBF_4 (48%, 6.5 µL, 9.1 mg, 50 µmol, 10 mol%) was introduced into the reaction mixture via a Hamilton syringe, resulting in a yellow coloration of the solution. The vial was sealed with a septum-cap, and a gentle stream of argon was passed through the solution via a needle (Φ 0.80 × 120 mm) for 2 minutes. The vial was then placed in a photoreactor equipped with 450nm LED module and irradiated for 18 hours at approximately 25 °C, maintained by an in-built cooling fan. Upon completion of the irradiation, the reaction mixture was poured into aqueous HBF_4 solution (0.1 M, 5 mL), and the resulting heterogeneous phase was extracted with dichloromethane (4 × 1 mL). The combined organic phases were dried over Na_2SO_4, filtered, and concentrated under reduced pressure. The residue was analyzed by ^{1}H NMR spectroscopy to determine regioselectivity (>25:1 r.r.) and subsequently purified by flash column chromatography on silica gel, eluting with hexanes/EtOAc (10/1 v/v) +2% AcOH, to afford 80 mg of alcohol **6l** as a colorless solid (yield: 87%).

R*f* = 0.17 (hexanes/EtOAc (10/1 v/v) +2% AcOH, $KMnO_4$).

Melting point:

NMR Spectroscopy:

^{1}H NMR (500 MHz, $CDCl_3$, 25 ºC, δ): 3.87 – 3.81 (m, 1H), 1.81 (br s, 1H), 1.71 – 1.61 (m, 2H), 1.49 – 1.23 (m, 20H).

^{13}C NMR (125 MHz, $CDCl_3$, 25 ºC, δ): 69.3, 32.6, 24.4, 24.0, 23.5, 23.4, 21.1.

HRMS-CI(m/z) calc'd for $C_{12}H_{28}O_1N_1$ $[M+NH_4]^+$, 202.2165; found, 202.2168; deviation: 1.5 ppm.

The analytical data obtained is consistent with that reported in the literature.[28]

Primary alcohol 6m

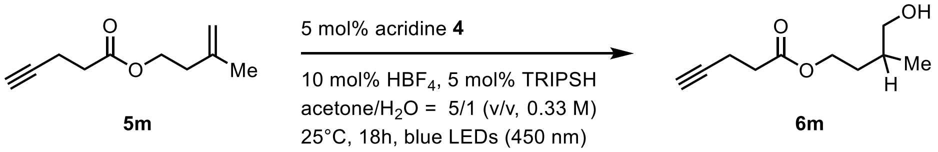

5m **6m**

To a 4-mL borosilicate vial equipped with a Teflon-coated magnetic stir bar were added alkene **5m** (83.1 mg, 0.500 mmol, 1.00 equiv.), acridine catalyst **4** (9.7 mg, 25 µmol, 5.0 mol%), TRIPSH (5.9 mg, 25 µmol, 5.0 mol%), and acetone/H_2O mixture (1.5 mL, 5/1 v/v, c = 0.33 M). Aqueous HBF_4 (48%, 6.5 µL, 9.1 mg, 50 µmol, 10 mol%) was introduced into the reaction mixture via a Hamilton syringe, resulting in a yellow

coloration of the solution. The vial was sealed with a septum-cap, and a gentle stream of argon was passed through the solution via a needle (Φ 0.80 × 120 mm) for 2 minutes. The vial was then placed in a photoreactor equipped with 450nm LED module and irradiated for 24 hours at approximately 25 °C, maintained by an in-built cooling fan. Upon completion of the irradiation, the reaction mixture was poured into aqueous HBF_4 solution (0.1 M, 5 mL), and the resulting heterogeneous phase was extracted with dichloromethane (4 × 1 mL). The combined organic phases were dried over Na_2SO_4, filtered, and concentrated under reduced pressure. The residue was analyzed by ^{1}H NMR spectroscopy to determine regioselectivity (>25:1 r.r.) and subsequently purified by flash column chromatography on silica gel, eluting with cyclohexane/MTBE (1/1 v/v), to afford 55 mg of alcohol **6m** as a pale-yellow liquid (yield: 60%).

R*f* = 0.15 (cyclohexane/MTBE (1/1 v/v), $KMnO_4$).

NMR Spectroscopy:

^{1}H NMR (500 MHz, $CDCl_3$, 25 ºC, δ): 4.24 – 4.10 (m, 2H), 3.50 – 3.46 (m, 2H), 2.56 – 2.51 (m, 2H), 2.51 – 2.46 (m, 2H), 1.97 (t, *J* = 2.6 Hz, 1H), 1.85 – 1.65 (m, 3H), 1.51 – 1.43 (m, 1H), 0.94 (d, *J* = 6.6 Hz, 3H).

^{13}C NMR (125 MHz, $CDCl_3$, 25 ºC, δ): 172.0, 82.6, 69.2, 67.9, 63.2, 33.5, 32.9, 32.1, 16.6, 14.5.

HRMS-ESI(m/z) calc'd for $C_{10}H_{16}O_3Na_1$ $[M+Na]^+$, 207.0992; found, 207.0990; deviation: –1.0 ppm.

Primary alcohol 6n

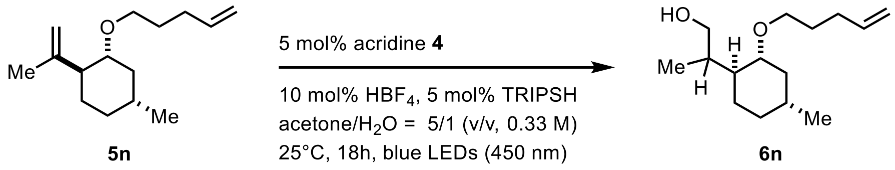

To a 4-mL borosilicate vial equipped with a Teflon-coated magnetic stir bar were added alkene **5n** (111 mg, 0.500 mmol, 1.00 equiv.), acridine catalyst **4** (9.7 mg, 25 µmol, 5.0 mol%), TRIPSH (5.9 mg, 25 µmol, 5.0 mol%), and acetone/H_2O mixture (1.5 mL, 5/1 v/v, c = 0.33 M). Aqueous HBF_4 (48%, 6.5 µL, 9.1 mg, 50 µmol, 10 mol%) was introduced into the reaction mixture via a Hamilton syringe, resulting in a yellow coloration of the solution. The vial was sealed with a septum-cap, and a gentle stream of argon was passed through the solution via a needle (Φ 0.80 × 120 mm) for 2 minutes. The vial was then placed in a photoreactor equipped with 450nm LED module and irradiated for 18 hours at approximately 25 °C, maintained by an in-built cooling fan. Upon completion of the irradiation, the reaction mixture was poured into aqueous HBF_4 solution (0.1 M, 5 mL), and the resulting heterogeneous phase was extracted with dichloromethane (4 × 1 mL). The combined organic phases were dried over Na_2SO_4, filtered, and concentrated under reduced pressure. The residue was analyzed by ^{1}H NMR spectroscopy to determine regioselectivity (>25:1 r.r.) and subsequently purified by flash column chromatography on silica gel, eluting with cyclohexane/MTBE (7/1 v/v + 2% AcOH to 5/1 v/v + 2% AcOH), to afford 73 mg of alcohol **6n** (diastereoisomer 1) and 26 mg of alcohol **6n** (diastereoisomer 2) as colorless liquids (yield: 83%, two diastereoisomers separated, 3:1 d.r.).

R*f* (diastereoisomer 1) = 0.23 (cyclohexane/MTBE (5/1 v/v) + 2% AcOH, $KMnO_4$).

R*f* (diastereoisomer 2) = 0.17 (cyclohexane/MTBE (5/1 v/v) + 2% AcOH, $KMnO_4$).

NMR Spectroscopy (diastereoisomer 1)

^{1}H NMR (500 MHz, $CDCl_3$, 25 ºC, δ): 5.87 – 5.71 (m, 1H), 5.04 – 4.99 (m, 1H), 4.98 – 4.92 (m, 1H), 3.63 (dt, *J* = 9.0, 6.8 Hz, 1H), 3.58 – 3.51 (m, 2H), 3.39 (br s, 1H), 3.30 (dt, *J* = 9.0, 6.8 Hz, 1H), 3.06 (td, *J* = 10.5, 4.1 Hz, 1H), 2.15 – 2.07 (m, 3H), 1.81 – 1.75 (m, 1H), 1.70 – 1.59 (m, 3H), 1.56 (dq, *J* = 13.0, 3.6 Hz, 1H), 1.52 – 1.45 (m, 1H), 1.37 – 1.30 (m, 1H), 1.25 (qd, *J* = 13.0, 3.6 Hz, 1H), 0.95 – 0.85 (m, 7H), 0.80 (q, *J* = 12.1 Hz, 1H).

^{13}C NMR (125 MHz, $CDCl_3$, 25 ºC, δ): 138.2, 115.0, 78.4, 67.8, 67.4, 46.1, 40.0, 39.5, 34.9, 31.4, 30.6, 30.5, 29.2, 22.3, 12.1.

HRMS-ESI(m/z) calc'd for $C_{15}H_{28}O_2Na_1$ $[M+Na]^+$, 263.1981; found, 263.1981; deviation: 0.0 ppm.

NMR Spectroscopy (diastereoisomer 2)

^{1}H NMR (500 MHz, $CDCl_3$, 25 ºC, δ): 5.86 – 5.76 (m, 1H), 5.05 – 4.99 (m, 1H), 4.98 – 4.94 (m, 1H), 3.63 (dt, *J* = 9.2, 6.5 Hz, 1H), 3.54 (dd, *J* = 10.6, 6.6 Hz, 1H), 3.47 (dd, *J* = 10.6, 6.6 Hz, 1H), 3.29 (dt, *J* = 9.2, 6.5 Hz, 1H), 3.04 (td, *J* = 10.5, 4.2 Hz, 1H), 2.15 – 2.09 (m, 3H), 2.09 – 2.04 (m, 1H), 1.89 (br s, 1H), 1.71 – 1.59 (m, 5H), 1.48 – 1.40 (m, 1H), 1.39 – 1.29 (m, 1H), 1.07 – 0.97 (m, 1H), 0.92 (d, *J* = 6.6 Hz, 3H), 0.89 – 0.81 (m, 4H).

^{13}C NMR (125 MHz, $CDCl_3$, 25 ºC, δ): 138.5, 114.9, 79.4, 67.8, 67.3, 43.9, 40.5, 35.6, 34.6, 31.5, 30.6, 29.5, 25.5, 22.5, 12.5.

HRMS-ESI(m/z) calc'd for $C_{15}H_{28}O_2Na_1$ $[M+Na]^+$, 263.1981; found, 263.1981; deviation: 0.0 ppm.

Primary alcohol 6o

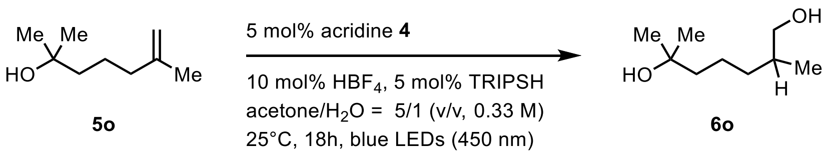

To a 4-mL borosilicate vial equipped with a Teflon-coated magnetic stir bar were added alkene **5o** (71.1 mg, 0.500 mmol, 1.00 equiv.), acridine catalyst **4** (9.7 mg, 25 µmol, 5.0 mol%), TRIPSH (5.9 mg, 25 µmol, 5.0 mol%), and acetone/H_2O mixture (1.5 mL, 5/1 v/v, c = 0.33 M). Aqueous HBF_4 (48%, 6.5 µL, 9.1 mg, 50 µmol, 10 mol%) was introduced into the reaction mixture via a Hamilton syringe, resulting in a yellow coloration of the solution. The vial was sealed with a septum-cap, and a gentle stream of argon was passed through the solution via a needle (Φ 0.80 × 120 mm) for 2 minutes. The vial was then placed in a photoreactor equipped with 450nm LED module and irradiated for 18 hours at approximately 25 °C, maintained by an in-built cooling fan. Upon completion of the irradiation, the reaction mixture was poured into aqueous HBF_4 solution (0.1 M, 5 mL), and the resulting heterogeneous phase was extracted with

dichloromethane (4 × 1 mL). The combined organic phases were dried over Na_2SO_4, filtered, and concentrated under reduced pressure. The residue was analyzed by ^{1}H NMR spectroscopy to determine regioselectivity (>25:1 r.r.) and subsequently purified by flash column chromatography on silica gel, eluting with MTBE/pentane (5/1 v/v), to afford 49 mg of alcohol **6o** as a colorless liquid (yield: 61%).

R*f* = 0.15 (MTBE/pentane (5/1 v/v), $KMnO_4$).

NMR Spectroscopy:

^{1}H NMR (500 MHz, $CDCl_3$, 25 ºC, δ): 3.47 (dd, *J* = 10.5, 6.2 Hz, 1H), 3.41 (dd, *J* = 10.5, 6.2 Hz, 1H), 2.02 – 1.54 (m, 3H), 1.48 – 1.36 (m, 4H), 1.36 – 1.29 (m, 1H), 1.19 (s, 6H), 1.15 – 1.05 (m, 1H), 0.90 (d, *J* = 6.7 Hz, 3H).

^{13}C NMR (125 MHz, $CDCl_3$, 25 ºC, δ): 71.1, 68.2, 44.2, 35.8, 33.7, 29.4, 29.3, 21.7, 16.7.

HRMS-ESI(m/z) calc'd for $C_9H_{20}O_2Na_1$ $[M+Na]^+$, 183.1356; found, 183.1354; deviation: –1.1 ppm.

The analytical data obtained is consistent with that reported in the literature.[29]

Primary alcohol 6p

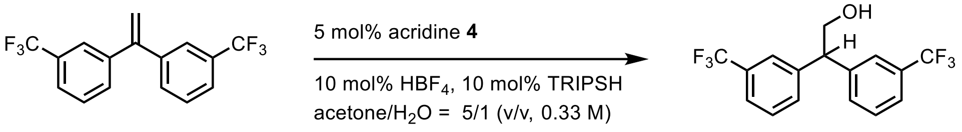

5p **6p**

To a 4-mL borosilicate vial equipped with a Teflon-coated magnetic stir bar were added alkene **5p** (158 mg, 0.500 mmol, 1.00 equiv.), acridine catalyst **4** (9.7 mg, 25 µmol, 5.0 mol%), TRIPSH (11.8 mg, 50.0 µmol, 10.0 mol%), and acetone/H_2O mixture (1.5 mL, 5/1 v/v, c = 0.33 M). Aqueous HBF_4 (48%, 6.5 µL, 9.1 mg, 50 µmol, 10 mol%) was introduced into the reaction mixture via a Hamilton syringe, resulting in a yellow coloration of the solution. The vial was sealed with a septum-cap, and a gentle stream of argon was passed through the solution via a needle (Φ 0.80 × 120 mm) for 2 minutes. The vial was then placed in a photoreactor equipped with 450nm LED module and irradiated for 18 hours at approximately 25 °C, maintained by an in-built cooling fan. Upon completion of the irradiation, the reaction mixture was poured into aqueous HBF_4 solution (0.1 M, 5 mL), and the resulting heterogeneous phase was extracted with dichloromethane (4 × 1 mL). The combined organic phases were dried over Na_2SO_4, filtered, and concentrated under reduced pressure. The residue was analyzed by ^{1}H NMR spectroscopy to determine regioselectivity (>25:1 r.r.) and subsequently purified by flash column chromatography on silica gel, eluting with pentane/EtOAc (4/1 v/v), to afford 129 mg of alcohol **6p** as a colorless liquid (yield: 77%).

R*f* = 0.24 (pentane/EtOAc (4/1 v/v), $KMnO_4$).

NMR Spectroscopy:

^{1}H NMR (500 MHz, $CDCl_3$, 25 ºC, δ): 7.56 – 7.51 (m, 4H), 7.49 – 7.42 (m, 4H), 4.33 (t, *J* = 6.9 Hz, 1H), 4.21 (dd, *J* = 6.9, 5.3 Hz, 2H), 1.66 (t, *J* = 5.3 Hz, 1H).

^{13}C NMR (125 MHz, $CDCl_3$, 25 ºC, δ): 141.9, 131.8, 131.3 (q, *J* = 32.2 Hz), 129.5, 125.2 (q, *J* = 3.7 Hz), 124.2 (q, *J* = 272.7 Hz), 124.2 (q, *J* = 3.8 Hz), 65.7, 53.2.

^{19}F NMR (470 MHz, $CDCl_3$, 25 ºC, δ): –62.6.

HRMS-ESI(m/z) calc'd for $C_{16}H_{12}O_1F_6Na_1$ $[M+Na]^+$, 357.0685; found, 357.0688; deviation: 0.8 ppm.

Secondary alcohol 6q

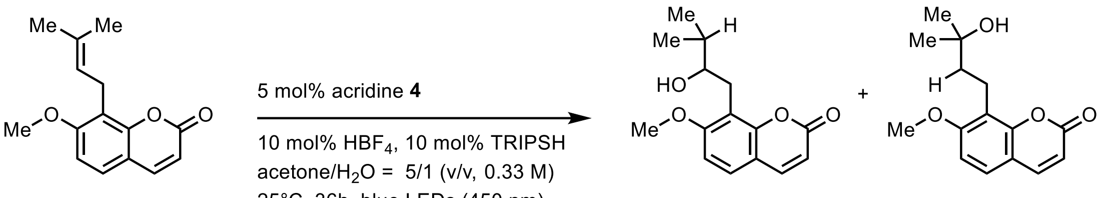

5q **6q** **6q'**

To a 4-mL borosilicate vial equipped with a Teflon-coated magnetic stir bar were added alkene **5q** (122 mg, 0.500 mmol, 1.00 equiv.), acridine catalyst **4** (9.7 mg, 25 µmol, 5.0 mol%), TRIPSH (11.8 mg, 50.0 µmol, 10.0 mol%), and acetone/H_2O mixture (1.5 mL, 5/1 v/v, c = 0.33 M). Aqueous HBF_4 (48%, 6.5 µL, 9.1 mg, 50 µmol, 10 mol%) was introduced into the reaction mixture via a Hamilton syringe, resulting in a yellow coloration of the solution. The vial was sealed with a septum-cap, and a gentle stream of argon was passed through the solution via a needle (Φ 0.80 × 120 mm) for 2 minutes. The vial was then placed in the photoreactor equipped with 450nm LED module and irradiated for 36 hours at approximately 25 °C, maintained by an in-built cooling fan. Upon completion of the irradiation, the reaction mixture was poured into aqueous HBF_4 solution (0.1 M, 5 mL), and the resulting heterogeneous phase was extracted with dichloromethane (4 × 1 mL). The combined organic phases were dried over Na_2SO_4, filtered, and concentrated under reduced pressure. The residue was analyzed by ^{1}H NMR spectroscopy to determine regioselectivity (2.8:1 r.r.) and subsequently purified by flash column chromatography on silica gel, eluting with dichloromethane/EtOAc (6/1 v/v to 2/1 v/v), to afford 79 mg of alcohol **6q** (anti-Markovnikov isomer) and 26 mg of alcohol **6q'** (Markovnikov isomer) as colorless solids (combined yield: 80%).

R_f (**6q**) = 0.61 (dichloromethane/EtOAc (2/1 v/v), $KMnO_4$).

R_f (**6q'**) = 0.42 (dichloromethane/EtOAc (2/1 v/v), $KMnO_4$).

NMR Spectroscopy (6q):

^{1}H NMR (500 MHz, $CDCl_3$, 25 ºC, δ): 7.61 (d, *J* = 9.5 Hz, 1H), 7.32 (d, *J* = 8.6 Hz, 1H), 6.85 (d, *J* = 8.6 Hz, 1H), 6.22 (d, *J* = 9.5 Hz, 1H), 3.91 (s, 3H), 3.65 – 3.59 (m, 1H), 3.06 (dd, *J* = 13.6, 3.5 Hz, 1H), 2.93 (dd, *J* = 13.6, 9.4 Hz, 1H), 1.91 (br s, 1H), 1.81 – 1.70 (m, 1H), 1.02 (dd, *J* = 6.8, 2.2 Hz, 6H).

^{13}C NMR (125 MHz, $CDCl_3$, 25 ºC, δ): 161.2, 160.7, 153.5, 143.9, 126.9, 116.1, 113.2, 107.4, 76.7, 56.3, 34.2, 28.1, 19.0, 17.3.

HRMS-CI(m/z) calc'd for $C_{15}H_{18}O_4Na_1$ $[M+Na]^+$, 285.1097; found, 285.1095; deviation: –0.7 ppm.

NMR Spectroscopy (6q'):

^{1}H NMR (500 MHz, $CDCl_3$, 25 ºC, δ): 7.62 (d, *J* = 9.4 Hz, 1H), 7.29 (d, *J* = 8.6 Hz, 1H), 6.83 (d, *J* = 8.6 Hz, 1H), 6.23 (d, *J* = 9.4 Hz, 1H), 3.92 (s, 3H), 2.95 – 2.90 (m, 2H), 1.81 – 1.68 (m, 3H), 1.31 (s, 6H).

^{13}C NMR (125 MHz, $CDCl_3$, 25 ºC, δ): 161.5, 160.3, 153.1, 144.0, 126.4, 118.9, 113.1, 113.1, 107.4, 71.2, 56.2, 42.7, 29.2, 17.8.

HRMS-CI(m/z) calc'd for $C_{15}H_{18}O_4Na_1$ $[M+Na]^+$, 285.1097; found, 285.1097; deviation: 0.0 ppm.

The analytical data obtained is consistent with that reported in the literature.[30]

Secondary alcohol 6r

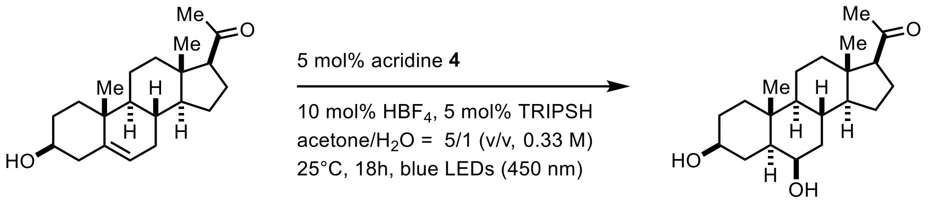

5r 6r

To a 4-mL borosilicate vial equipped with a Teflon-coated magnetic stir bar were added alkene **5r** (158 mg, 0.500 mmol, 1.00 equiv.), acridine catalyst **4** (9.7 mg, 25 µmol, 5.0 mol%), TRIPSH (5.9 mg, 25 µmol, 5.0 mol%), and acetone/H_2O mixture (1.5 mL, 5/1 v/v, c = 0.33 M). Aqueous HBF_4 (48%, 6.5 µL, 9.1 mg, 50 µmol, 10 mol%) was introduced into the reaction mixture via a Hamilton syringe, resulting in a yellow coloration of the solution. The vial was sealed with a septum-cap, and a gentle stream of argon was passed through the solution via a needle (Φ 0.80 × 120 mm) for 2 minutes. The vial was then placed in the photoreactor equipped with 450nm LED module and irradiated for 18 hours at approximately 25 °C, maintained by an in-built cooling fan. Upon completion of the irradiation, the reaction mixture was poured into aqueous HBF_4 solution (0.1 M, 5 mL), and the resulting heterogeneous phase was extracted with dichloromethane (4 × 1 mL). The combined organic phases were dried over Na_2SO_4, filtered, and concentrated under reduced pressure. The residue was analyzed by ^{1}H NMR spectroscopy to determine regioselectivity (one isomer detected) and subsequently purified by flash column chromatography on silica gel, eluting with EtOAc/dichloromethane (2/1 v/v), to afford 117 mg of alcohol **6r** as a colorless solid (yield: 70%).

R_f = 0.20 (EtOAc/dichloromethane (2/1 v/v), $KMnO_4$).

NMR Spectroscopy (6r):

^{1}H NMR (500 MHz, $CDCl_3$, 25 ºC, δ): 3.81 – 3.78 (m, 1H), 3.63 (tt, *J* = 10.7, 5.0 Hz, 1H), 2.51 (t, *J* = 8.9 Hz, 1H), 2.19 – 2.12 (m, 1H), 2.10 (s, 3H), 2.03 – 1.99 (m, 1H), 1.85 – 1.56 (m, 11H), 1.48 – 1.34 (m, 3H), 1.29 – 1.19 (m, 1H), 1.19 – 1.11 (m, 3H), 1.02 (s, 3H), 0.97 (td, *J* = 14.7, 5.0 Hz, 1H), 0.75 – 0.67 (m, 1H), 0.62 (s, 3H).

^{13}C NMR (125 MHz, $CDCl_3$, 25 ºC, δ): 209.8, 71.9, 71.7, 63.9, 56.5, 54.2, 47.5, 44.4, 39.6, 39.1, 38.6, 35.6, 35.44, 31.6, 31.6, 30.6, 24.5, 22.9, 21.2, 15.9, 13.6.

HRMS-EI(m/z) calc'd for $C_{21}H_{34}O_3$ [M]$^+$, 334.2502; found, 334.2502; deviation: 0.0 ppm.

Secondary alcohol 6s

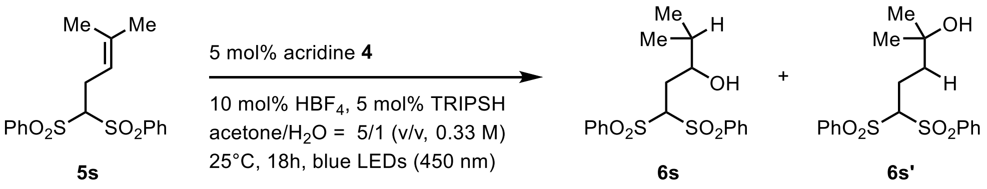

To a 4-mL borosilicate vial equipped with a Teflon-coated magnetic stir bar were added alkene **5s** (182 mg, 0.500 mmol, 1.00 equiv.), acridine catalyst **4** (9.7 mg, 25 µmol, 5.0 mol%), TRIPSH (5.9 mg, 25 µmol, 5.0 mol%), and acetone/H_2O mixture (1.5 mL, 5/1 v/v, c = 0.33 M). Aqueous HBF_4 (48%, 6.5 µL, 9.1 mg, 50 µmol, 10 mol%) was introduced into the reaction mixture via a Hamilton syringe, resulting in a yellow coloration of the solution. The vial was sealed with a septum-cap, and a gentle stream of argon was passed through the solution via a needle (Φ 0.80 × 120 mm) for 2 minutes. The vial was then placed in the photoreactor equipped with 450nm LED module and irradiated for 18 hours at approximately 25 °C, maintained by an in-built cooling fan. Upon completion of the irradiation, the reaction mixture was poured into aqueous HBF_4 solution (0.1 M, 5 mL), and the resulting heterogeneous phase was extracted with dichloromethane (4 × 1 mL). The combined organic phases were dried over Na_2SO_4, filtered, and concentrated under reduced pressure. The residue was analyzed by ^{1}H NMR spectroscopy to determine regioselectivity (1.2:1 r.r.) and subsequently purified by flash column chromatography on silica gel, eluting with cyclohexane/dichloromethane/EtOAc (3/1/1 v/v to 1/1/1 v/v), to afford 84 mg of alcohol **6s** (anti-Markovnikov isomer) as a colorless liquid and 65 mg of alcohol **6s'** (Markovnikov isomer) as a colorless solid (combined yield: 78%).

R*f* (**6s**) = 0.64 (cyclohexane/dichloromethane/EtOAc (1/1/1 v/v), $KMnO_4$).

R*f* (**6s'**) = 0.29 (cyclohexane/dichloromethane/EtOAc (1/1/1 v/v), $KMnO_4$).

NMR Spectroscopy (6s):

^{1}H NMR (500 MHz, $CDCl_3$, 25 ºC, δ): 7.95 – 7.90 (m, 4H), 7.71 – 7.65 (m, 2H), 7.58 – 7.52 (m, 4H), 4.98 – 4.94 (m, 1H), 3.56 – 3.50 (m, 1H), 2.34 – 2.23 (m, 2H), 1.87 (br s, 1H), 1.67 – 1.58 (m, 1H), 0.88 (dd, *J* = 7.9, 6.8 Hz, 6H).

^{13}C NMR (125 MHz, $CDCl_3$, 25 ºC, δ): 138.0, 137.9, 134.7, 134.6, 129.7, 129.6, 129.2, 129.2, 80.5, 73.8, 34.4, 30.0, 18.4, 17.6.

HRMS-ESI(m/z) calc'd for $C_{18}H_{22}O_5S_2Na_1$ [M+Na]$^+$, 405.0801; found, 405.0803; deviation: 0.5 ppm.

NMR Spectroscopy (6s'):

^{1}H NMR (500 MHz, $CDCl_3$, 25 ºC, δ): 7.94 (d, *J* = 7.2 Hz, 4H), 7.68 (t, *J* = 7.5 Hz, 2H), 7.56 (t, *J* = 7.9 Hz, 4H), 4.83 (t, *J* = 5.6 Hz, 1H), 2.35 – 2.29 (m, 2H), 1.83 – 1.76 (m, 2H), 1.60 (br s, 1H), 1.16 (s, 6H).

^{13}C NMR (125 MHz, $CDCl_3$, 25 ºC, δ): 138.0, 134.6, 129.7, 129.2, 83.2, 71.0, 40.5, 29.3, 21.1.

HRMS-ESI(m/z) calc'd for $C_{18}H_{22}O_5S_2Na_1$ [M+Na]$^+$, 405.0801; found, 405.0802; deviation: 0.2 ppm.

The analytical data obtained is consistent with that reported in the literature.[31]

Primary alcohol 6t

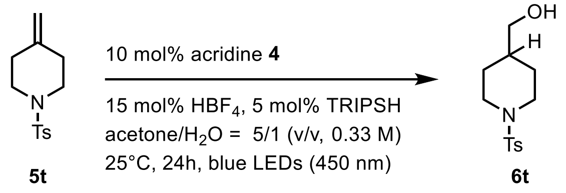

To a 4-mL borosilicate vial equipped with a Teflon-coated magnetic stir bar were added alkene **5t** (126 mg, 0.500 mmol, 1.00 equiv.), acridine catalyst **4** (19.3 mg, 50.0 µmol, 10.0 mol%), TRIPSH (5.9 mg, 25 µmol, 5.0 mol%), and acetone/H_2O mixture (1.5 mL, 5/1 v/v, c = 0.33 M). Aqueous HBF_4 (48%, 9.7 µL, 14 mg, 75 µmol, 15 mol%) was introduced into the reaction mixture via a Hamilton syringe, resulting in a yellow coloration of the solution. The vial was sealed with a septum-cap, and a gentle stream of argon was passed through the solution via a needle (Φ 0.80 × 120 mm) for 2 minutes. The vial was then placed in a photoreactor equipped with 450nm LED module and irradiated for 24 hours at approximately 25 °C, maintained by an in-built cooling fan. Upon completion of the irradiation, the reaction mixture was poured into aqueous HBF_4 solution (0.1 M, 5 mL), and the resulting heterogeneous phase was extracted with dichloromethane (4 × 1 mL). The combined organic phases were dried over Na_2SO_4, filtered, and concentrated under reduced pressure. The residue was analyzed by ^{1}H NMR spectroscopy to determine regioselectivity (>25:1 r.r.) and subsequently purified by flash column chromatography on silica gel, eluting with dichloromethane/EtOAc (8/1 v/v) + 2% AcOH, to afford 45 mg of alcohol **6t** as a colorless solid (yield: 33%).

R*f* (**6t**) = 0.23 (dichloromethane/EtOAc (8/1 v/v) + 2% AcOH, $KMnO_4$).

NMR Spectroscopy (6t):

^{1}H NMR (500 MHz, $CDCl_3$, 25 ºC, δ): 7.61 (d, *J* = 8.2 Hz, 2H), 7.30 (d, *J* = 8.2 Hz, 2H), 3.82 – 3.72 (m, 2H), 3.43 (d, *J* = 6.2 Hz, 2H), 2.41 (s, 3H), 2.22 (td, *J* = 12.0, 2.5 Hz, 2H), 1.83 (br s, 1H), 1.80 – 1.72 (m, 2H), 1.44 – 1.35 (m, 1H), 1.30 (qd, *J* = 12.0, 4.1 Hz, 2H).

^{13}C NMR (125 MHz, $CDCl_3$, 25 ºC, δ): 143.6, 133.2, 129.7, 127.8, 67.1, 46.2, 37.9, 28.2, 21.6.

HRMS-ESI(m/z) calc'd for $C_{13}H_{19}N_1O_3S_1Na_1$ [M+Na]$^+$, 292.0977; found, 292.0982; deviation: 1.7 ppm.

The analytical data obtained is consistent with that reported in the literature.[32]

Other anti-Markovnikov hydrofunctionalization reactions

Methyl ether 7

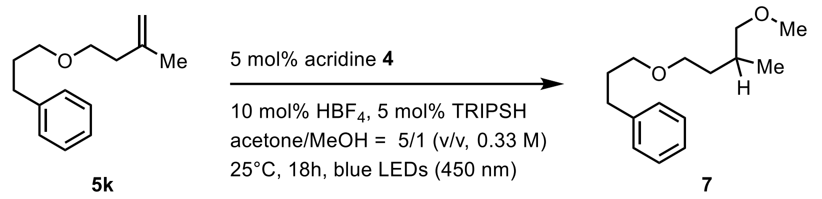

To a 4-mL borosilicate vial equipped with a Teflon-coated magnetic stir bar were added alkene **5k** (102 mg, 0.500 mmol, 1.00 equiv.), acridine catalyst **4** (9.7 mg, 25 µmol, 5.0 mol%), TRIPSH (5.9 mg, 25 µmol, 5.0 mol%), and acetone/MeOH mixture (1.5 mL, 5/1 v/v, c = 0.33 M). Aqueous HBF_4 (48%, 6.5 µL, 9.1 mg, 50 µmol, 10 mol%) was introduced into the reaction mixture via a Hamilton syringe, resulting in a yellow coloration of the solution. The vial was sealed with a septum-cap, and a gentle stream of argon was passed through the solution via a needle (Φ 0.80 × 120 mm) for 2 minutes. The vial was then placed in a photoreactor equipped with 450nm LED module and irradiated for 18 hours at approximately 25 °C, maintained by an in-built cooling fan. Upon completion of the irradiation, the reaction mixture was poured into aqueous HBF_4 solution (0.1 M, 5 mL), and the resulting heterogeneous phase was extracted with dichloromethane (4 × 1 mL). The combined organic phases were dried over Na_2SO_4, filtered, and concentrated under reduced pressure. The residue was analyzed by ^{1}H NMR spectroscopy to determine regioselectivity (>25:1 r.r.) and subsequently purified by flash column chromatography on silica gel, eluting with cyclohexane/MTBE (20/1 v/v), to afford 85 mg of ether **7** as a colorless liquid (yield: 72%).

R*f* = 0.19 (cyclohexane/MTBE (20/1 v/v), $KMnO_4$).

NMR Spectroscopy:

^{1}H NMR (500 MHz, $CDCl_3$, 25 ºC, δ): 7.31 – 7.27 (m, 2H), 7.22 – 7.17 (m, 3H), 3.50 – 3.45 (m, 2H), 3.45 – 3.40 (m, 2H), 3.34 (s, 3H), 3.27 (dd, *J* = 9.2, 6.3 Hz, 1H), 3.20 (dd, *J* = 9.2, 6.3 Hz, 1H), 2.70 (t, *J* = 7.5 Hz, 2H), 1.94 – 1.87 (m, 3H), 1.77 – 1.68 (m, 1H), 1.46 – 1.37 (m, 1H), 0.96 (d, *J* = 6.8 Hz, 3H).

^{13}C NMR (125 MHz, $CDCl_3$, 25 ºC, δ): 142.2, 128.6, 128.4, 125.8, 78.5, 70.0, 69.1, 58.9, 33.7, 32.5, 31.5, 30.7, 17.3.

HRMS-ESI(m/z) calc'd for $C_{15}H_{24}O_2Na_1$ $[M+Na]^+$, 259.1668; found, 259.1668; deviation: 0.0 ppm.

Ethyl ether 8

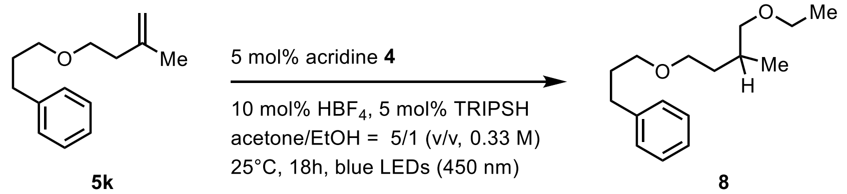

To a 4-mL borosilicate vial equipped with a Teflon-coated magnetic stir bar were added alkene **5k** (102 mg,

0.500 mmol, 1.00 equiv.), acridine catalyst **4** (9.7 mg, 25 µmol, 5.0 mol%), TRIPSH (5.9 mg, 25 µmol, 5.0 mol%), and acetone/EtOH mixture (1.5 mL, 5/1 v/v, c = 0.33 M). Aqueous HBF_4 (48%, 6.5 µL, 9.1 mg, 50 µmol, 10 mol%) was introduced into the reaction mixture via a Hamilton syringe, resulting in a yellow coloration of the solution. The vial was sealed with a septum-cap, and a gentle stream of argon was passed through the solution via a needle (Φ 0.80 × 120 mm) for 2 minutes. The vial was then placed in a photoreactor equipped with 450nm LED module and irradiated for 18 hours at approximately 25 °C, maintained by an in-built cooling fan. Upon completion of the irradiation, the reaction mixture was poured into aqueous HBF_4 solution (0.1 M, 5 mL), and the resulting heterogeneous phase was extracted with dichloromethane (4 × 1 mL). The combined organic phases were dried over Na_2SO_4, filtered, and concentrated under reduced pressure. The residue was analyzed by ^{1}H NMR spectroscopy to determine regioselectivity (>25:1 r.r.) and subsequently purified by flash column chromatography on silica gel, eluting with cyclohexane/MTBE (20/1 v/v), to afford 68 mg of ether **8** as a colorless liquid (yield: 54%).

R*f* = 0.21 (cyclohexane/MTBE (20/1 v/v), $KMnO_4$).

NMR Spectroscopy:

^{1}H NMR (500 MHz, $CDCl_3$, 25 ºC, δ): 7.31 – 7.26 (m, 2H), 7.22 – 7.17 (m, 3H), 3.50 – 3.44 (m, 4H), 3.44 – 3.40 (m, 2H), 3.30 (dd, *J* = 9.2, 6.4 Hz, 1H), 3.23 (dd, *J* = 9.2, 6.4 Hz, 1H), 2.70 (t, *J* = 7.3 Hz, 2H), 1.94 – 1.85 (m, 3H), 1.78 – 1.69 (m, 1H), 1.45 – 1.35 (m, 1H), 1.20 (t, *J* = 7.0 Hz, 3H), 0.96 (d, *J* = 6.7 Hz, 3H).

^{13}C NMR (125 MHz, $CDCl_3$, 25 ºC, δ): 142.2, 128.6, 128.4, 125.8, 76.3, 70.1, 69.2, 66.4, 33.8, 32.5, 31.5, 30.9, 17.4, 15.3.

HRMS-ESI(m/z) calc'd for $C_{16}H_{26}O_2Na_1$ $[M+Na]^+$, 273.1825; found, 273.1824; deviation: –0.4 ppm.

Isopropyl ether 9

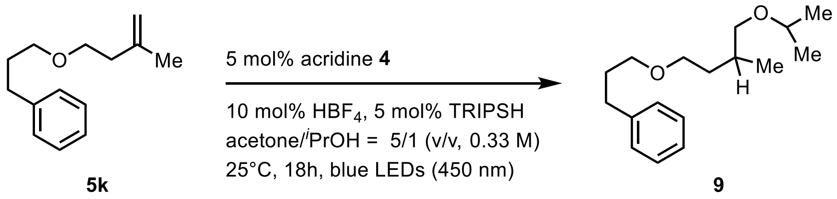

To a 4-mL borosilicate vial equipped with a Teflon-coated magnetic stir bar were added alkene **5k** (102 mg, 0.500 mmol, 1.00 equiv.), acridine catalyst **4** (9.7 mg, 25 µmol, 5.0 mol%), TRIPSH (5.9 mg, 25 µmol, 5.0 mol%), and acetone/^{i}PrOH mixture (1.5 mL, 5/1 v/v, c = 0.33 M). Aqueous HBF_4 (48%, 6.5 µL, 9.1 mg, 50 µmol, 10 mol%) was introduced into the reaction mixture via a Hamilton syringe, resulting in a yellow coloration of the solution. The vial was sealed with a septum-cap, and a gentle stream of argon was passed through the solution via a needle (Φ 0.80 × 120 mm) for 2 minutes. The vial was then placed in a photoreactor equipped with 450nm LED module and irradiated for 18 hours at approximately 25 °C, maintained by an in-built cooling fan. Upon completion of the irradiation, the reaction mixture was poured into aqueous HBF_4 solution (0.1 M, 5 mL), and the resulting heterogeneous phase was extracted with

dichloromethane (4 × 1 mL). The combined organic phases were dried over Na_2SO_4, filtered, and concentrated under reduced pressure. The residue was analyzed by ^{1}H NMR spectroscopy to determine regioselectivity (>25:1 r.r.) and subsequently purified by flash column chromatography on silica gel, eluting with cyclohexane/MTBE (40/1 v/v), to afford 57 mg of ether **9** as a colorless liquid (yield: 43%).

R_f = 0.10 (cyclohexane/MTBE (40/1 v/v), $KMnO_4$).

NMR Spectroscopy:

^{1}H NMR (500 MHz, $CDCl_3$, 25 ºC, δ): 7.31 – 7.26 (m, 2H), 7.21 – 7.16 (m, 3H), 3.53 (hept, *J* = 6.2 Hz, 1H), 3.50 – 3.45 (m, 2H), 3.44 – 3.40 (m, 2H), 3.29 (dd, *J* = 9.1, 6.3 Hz, 1H), 3.21 (dd, *J* = 9.1, 6.3 Hz, 1H), 2.70 (t, *J* = 7.7 Hz, 2H), 1.93 – 1.86 (m, 2H), 1.86 – 1.79 (m, 1H), 1.78 – 1.70 (m, 1H), 1.44 – 1.34 (m, 1H), 1.15 (d, *J* = 6.2 Hz, 6H), 0.95 (d, *J* = 6.6 Hz, 3H).

^{13}C NMR (125 MHz, $CDCl_3$, 25 ºC, δ): 142.2, 128.6, 128.4, 125.8, 73.9, 71.6, 70.1, 69.3, 33.9, 32.5, 31.5, 31.2, 22.3, 22.2, 17.4.

HRMS-ESI(m/z) calc'd for $C_{17}H_{28}O_2Na_1$ $[M+Na]^+$, 287.1981; found, 287.1981; deviation: 0.0 ppm.

List of substrates that failed in anti-Markovnikov hydration reaction

Several alkenes failed to yield the corresponding alcohols under the standard reaction conditions (yield <5%). Two distinct patterns were identified among these unsuccessful transformations.

The first group comprised alkenes of electron-deficient character, characterized by oxidation potentials exceeding the effective operational range of the reaction (E > + 2.40 V vs. SCE). These alkenes typically contained heteroatoms (*e.g.,* O, Cl) at the *α*-position to the double bond or strong electron-withdrawing substituents at the *β*-position (*e.g.,* -CN, $-CO_2H$, $-SO_2R$, *etc.*)

The second group consisted of alkenes bearing functional groups with oxidation potentials significantly lower than that of the alkene itself. Examples of such functionalities include unprotected amines, sulfides, electron-rich aromatic rings, *etc.,* which likely underwent preferential oxidation under the reaction conditions, thus impeding the desired transformation or leading to a degradation of the starting material.

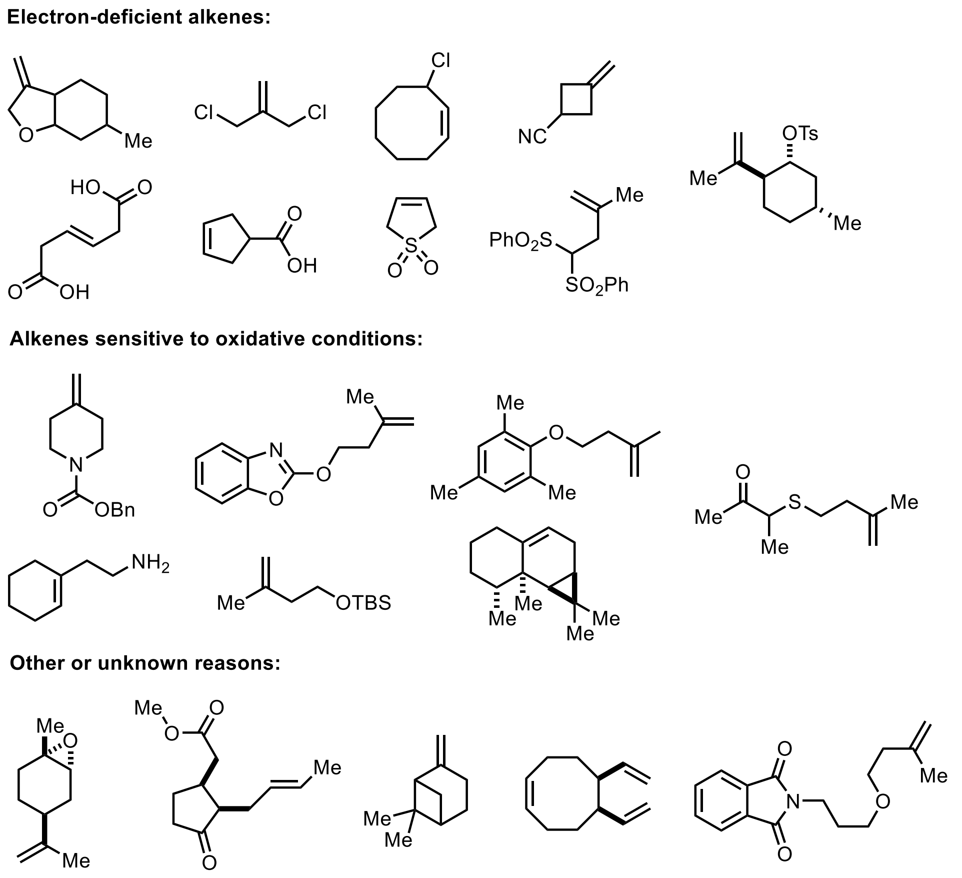

Figure S2. Unsuccessful substrates.

Photochemical and electrochemical studies

Cyclic voltammograms of alkenes for oxidation potential series

Cyclic voltammetry measurements were conducted on an Autolab PGSTAT204 potentiostat, using a platinum working electrode, a silver/silver chloride (Ag/AgCl) reference electrode, and a platinum sheet auxiliary electrode. For each measurement series, the quasi-reference electrode was calibrated by recording a cyclic voltammogram (CV) of a ferrocene solution (Fc, 10.0 mM) in acetonitrile (MeCN, 5.00 mL) containing tetrabutylammonium tetrafluoroborate ($TBABF_4$, 100 mM) as the electrolyte, with a scan rate of 100 mV/s. Prior to measurement, the solution was purged with argon for 5 minutes to remove dissolved oxygen. The resulting half-wave potential of the ferrocene/ferrocenium couple was measured as $E_{1/2}$ (Fc^+/Fc) = +0.463 V. This value was compared to the literature reference potential ($E_{1/2}$ (Fc^+/Fc) = +0.380 V vs SCE),[33] establishing a correction factor of -0.083 V for conversion of the measured redox potentials in the following series to the potentials against SCE.

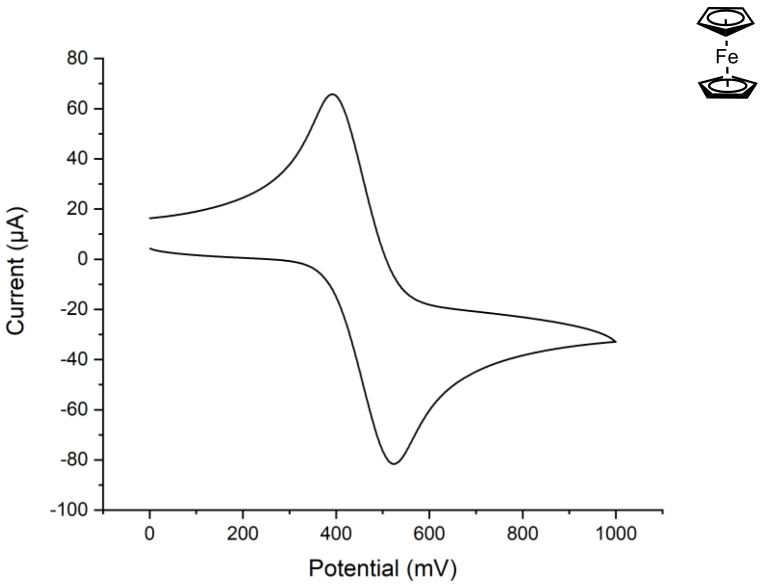

Figure S3. Cyclic voltammogram of ferrocene in MeCN (alkene series). Scan rate 100 mV·s^{-1}.

Cyclic voltammograms were then recorded for a number of alkenes. Measurements were conducted on solutions of an alkene (10.0 mM) in acetonitrile (5.00 mL) containing tetrabutylammonium tetrafluoroborate ($TBABF_4$, 100 mM) as the electrolyte, with scan rates of 100 mV/s. Prior to each measurement, the solution was purged with argon for 5 minutes to remove dissolved oxygen.

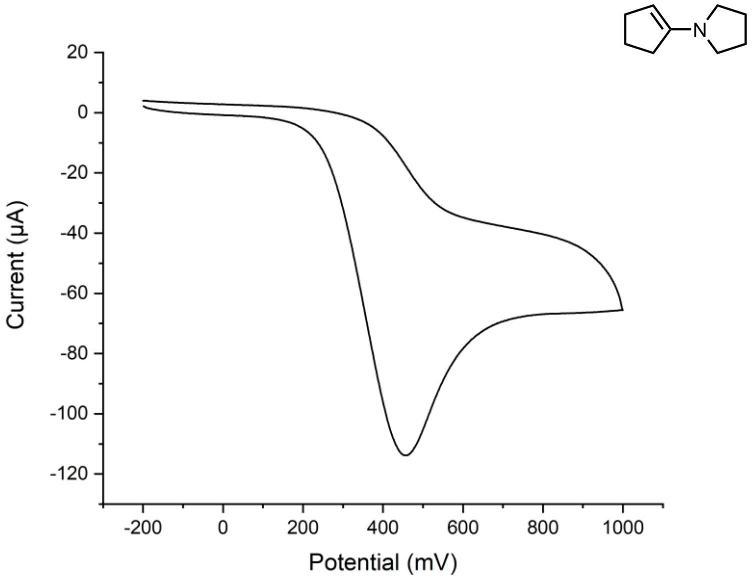

Figure S4. Cyclic voltammogram of 1-pyrrolidino-1-cyclopentene in MeCN. Scan rate 100 mV·s^{-1}.

The measured $E_{p/2}$ = 0.341 V, which corresponds to 0.258 V versus SCE in MeCN.

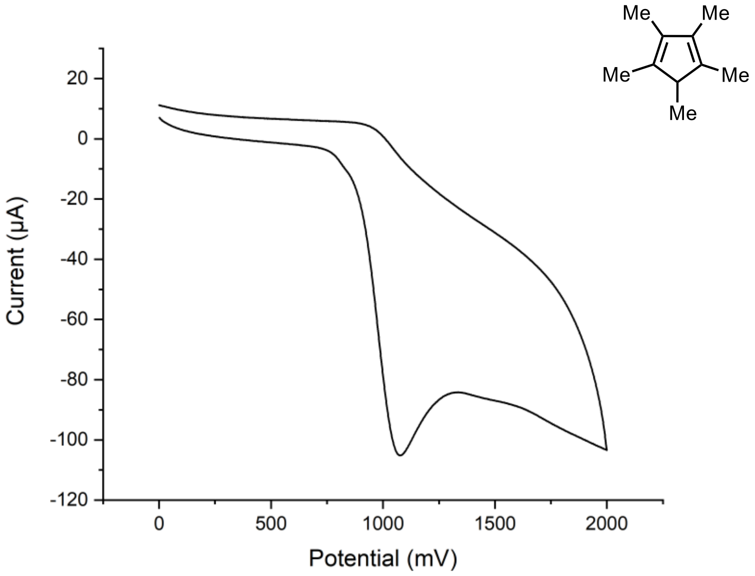

Figure S5. Cyclic voltammogram of pentamethylcyclopentadiene in MeCN. Scan rate 100 mV·s^{-1}.

The measured $E_{p/2}$ = 0.962 V, which corresponds to 0.879 V versus SCE in MeCN.

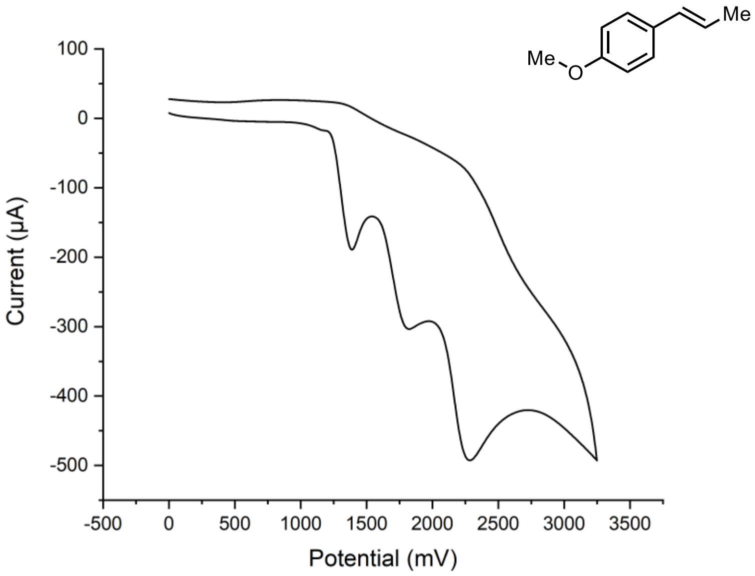

Figure S6. Cyclic voltammogram of trans-anethole in MeCN. Scan rate 100 mV·s^{-1}.

The measured $E_{p/2}$ = 1.297 V, which corresponds to 1.214 V versus SCE in MeCN.

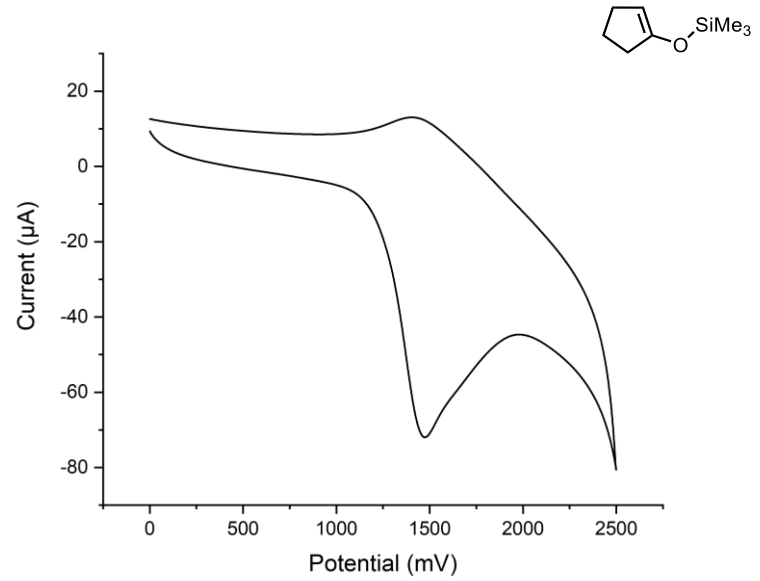

Figure S7. Cyclic voltammogram of 1-(trimethylsiloxy)cyclopentene in MeCN. Scan rate 100 mV·s^{-1}.

The measured $E_{p/2}$ = 1.330 V, which corresponds to 1.247 V versus SCE in MeCN.

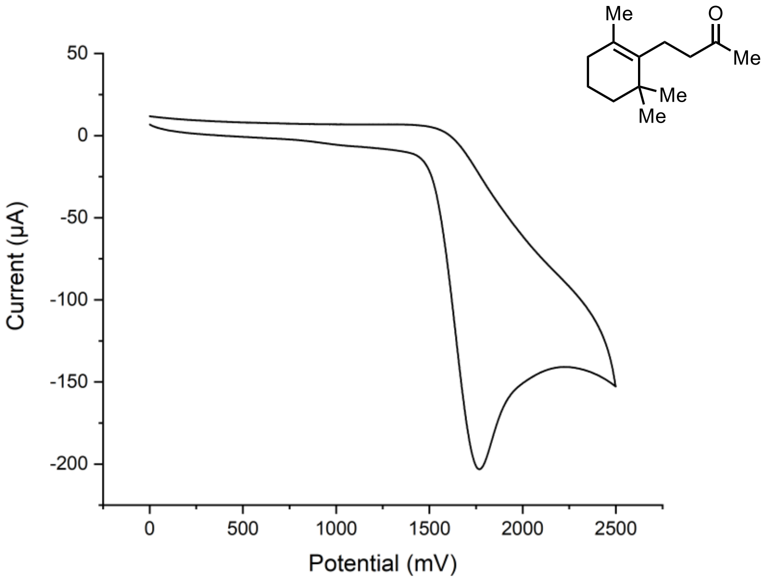

Figure S8. Cyclic voltammogram of dihydro-*β*-ionone in MeCN. Scan rate 100 mV·s^{-1}.

The measured $E_{p/2}$ = 1.622 V, which corresponds to 1.539 V versus SCE in MeCN.

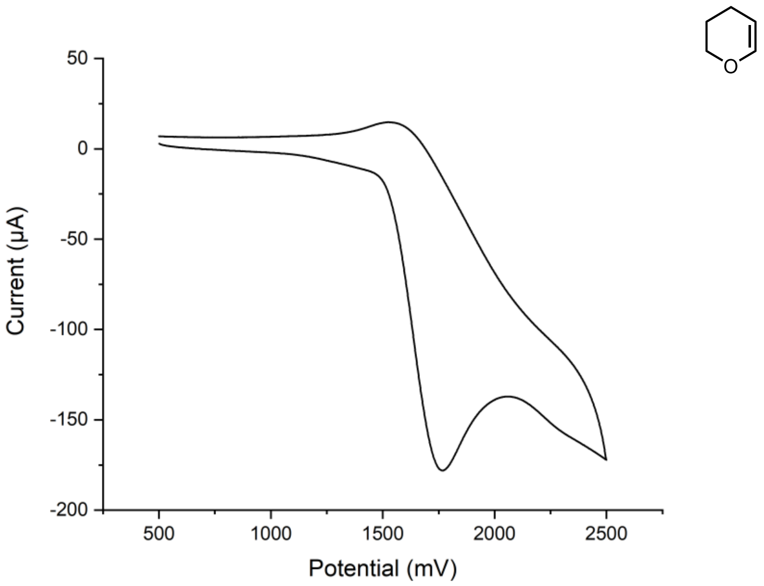

Figure S9. Cyclic voltammogram of dihydropyran in MeCN. Scan rate 100 mV·s^{-1}.

The measured $E_{p/2}$ = 1.621 V, which corresponds to 1.538 V versus SCE in MeCN.

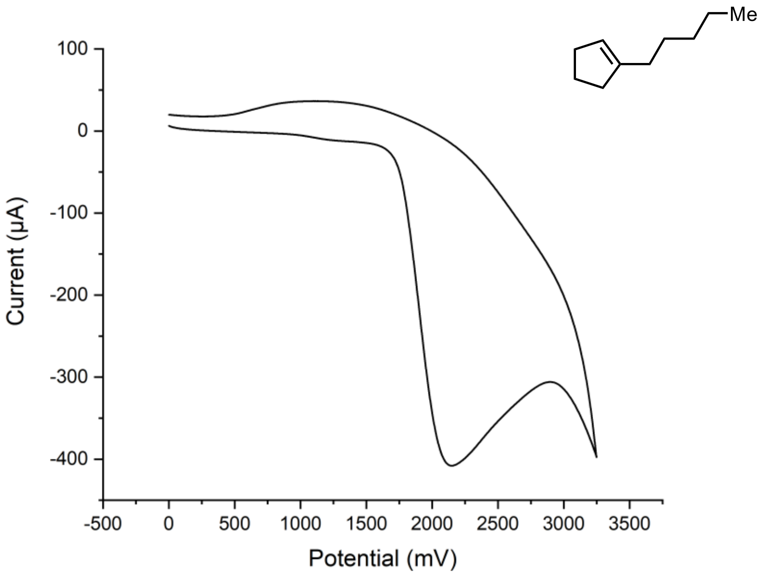

Figure S10. Cyclic voltammogram of 1-pentyl-1-cyclopentene in MeCN. Scan rate 100 mV·s^{-1}.

The measured $E_{p/2}$ = 1.892 V, which corresponds to 1.809 V versus SCE in MeCN.

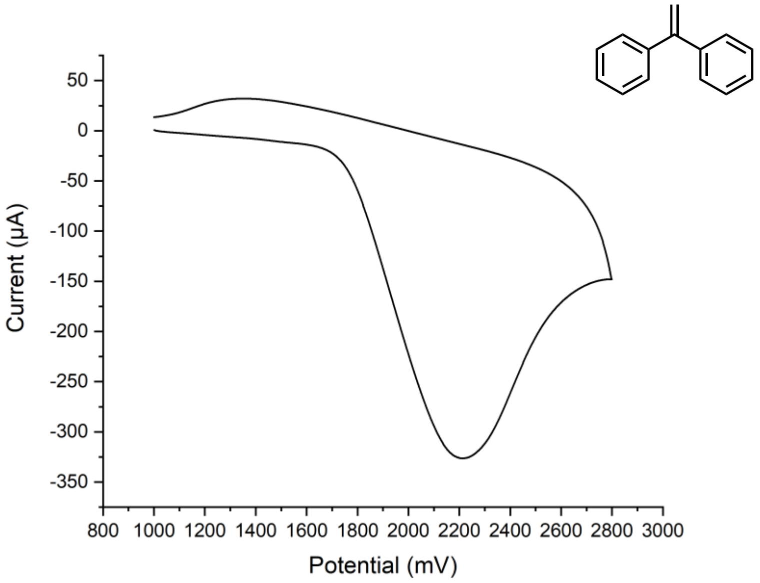

Figure S11. Cyclic voltammogram of 1,1-diphenylethylene in MeCN. Scan rate 100 mV·s^{-1}.

The measured $E_{p/2}$ = 1.930 V, which corresponds to 1.847 V versus SCE in MeCN.

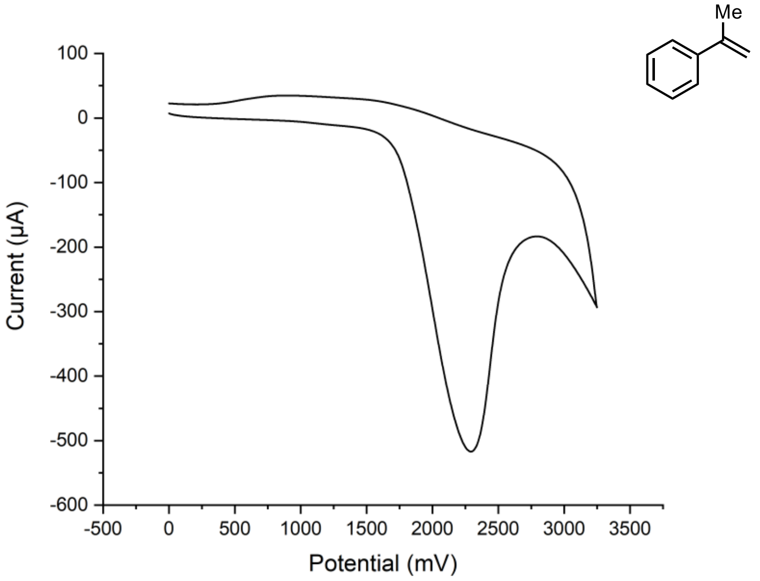

Figure S12. Cyclic voltammogram of *α*-methylstyrene in MeCN. Scan rate 100 mV·s^{-1}.

The measured $E_{p/2}$ = 1.968 V, which corresponds to 1.885 V versus SCE in MeCN.

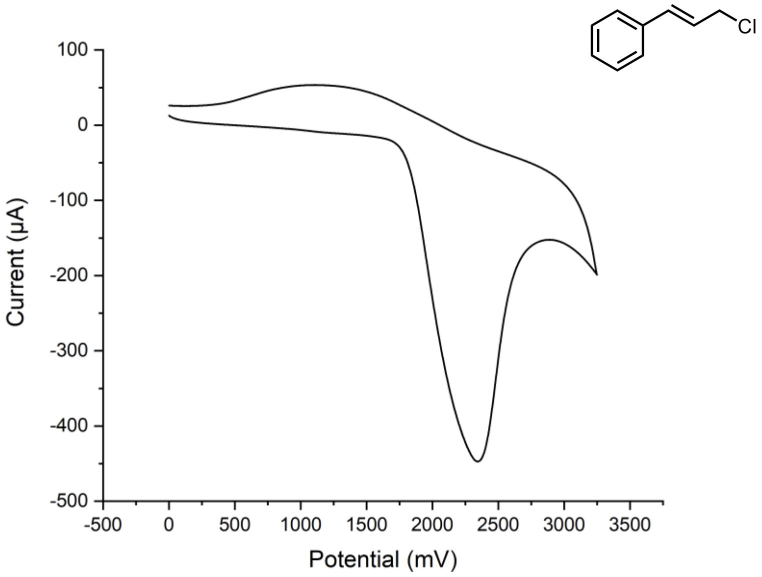

Figure S13. Cyclic voltammogram of cinnamylchloride in MeCN. Scan rate 100 mV·s^{-1}.

The measured $E_{p/2}$ = 1.994 V, which corresponds to 1.911 V versus SCE in MeCN.

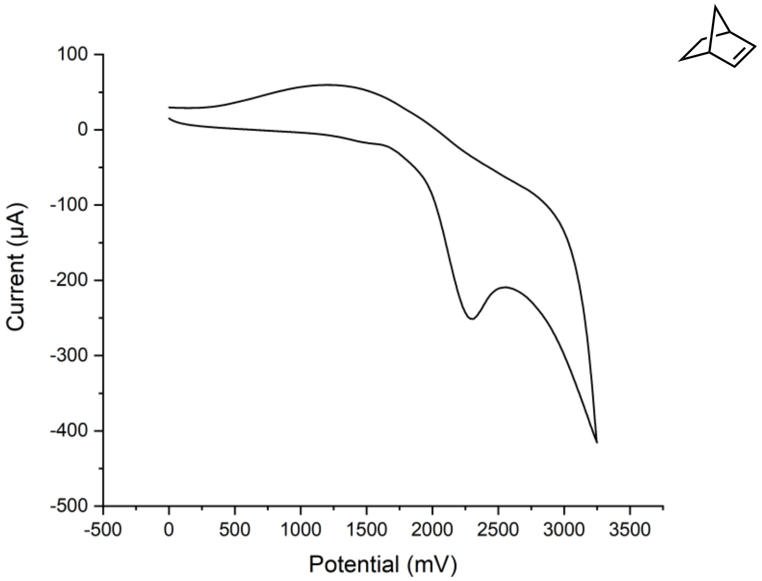

Figure S14. Cyclic voltammogram of norbornene in MeCN. Scan rate 100 mV·s^{-1}.

The measured $E_{p/2}$ = 2.067 V, which corresponds to 1.984 V versus SCE in MeCN.

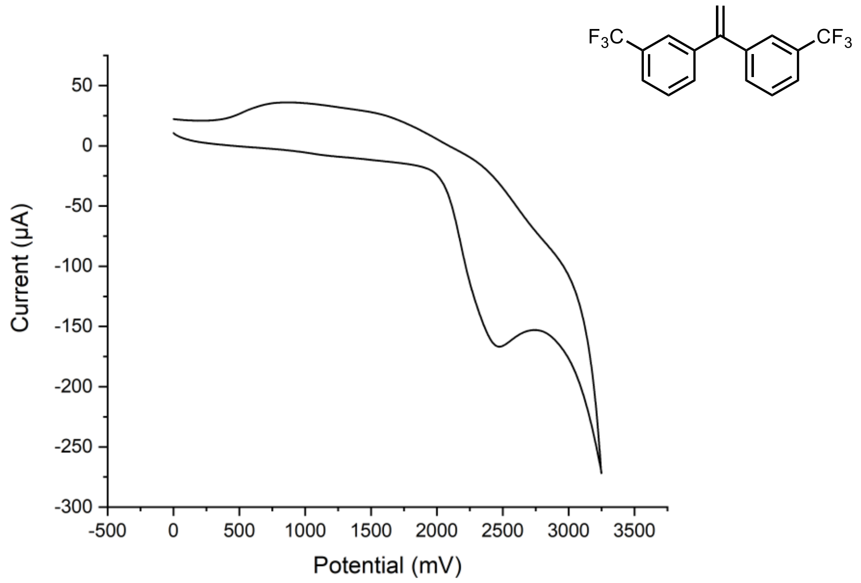

Figure S15. Cyclic voltammogram of 1,1-bis(3-(trifluoromethyl)phenyl)ethene in MeCN. Scan rate 100 $mV{\cdot}s^{-1}$.

The measured $E_{p/2}$ = 2.185 V, which corresponds to 2.102 V versus SCE in MeCN.

Cyclic voltammograms of acridinium salts

Cyclic voltammetry measurements were conducted on an Autolab PGSTAT204 potentiostat, using a platinum working electrode, a silver/silver chloride (Ag/AgCl) reference electrode, and a platinum sheet auxiliary electrode. The quasi-reference electrode was calibrated by recording a cyclic voltammogram (CV) of a ferrocene solution (Fc, 1.5 mM) in acetonitrile (MeCN, 3.0 mL) containing tetrabutylammonium tetrafluoroborate ($TBABF_4$, 45 mM) as the electrolyte, with a scan rate of 100 mV/s. Prior to measurement, the solution was purged with argon for 5 minutes to remove dissolved oxygen. The resulting half-wave potential of the ferrocene/ferrocenium couple was measured as $E_{1/2}$ (Fc^+/Fc) = +0.455 V. This value was compared to the literature reference potential ($E_{1/2}$ (Fc^+/Fc) = +0.380 V vs SCE),[33] establishing a correction factor of -0.075 V for conversion of the measured redox potentials to the potentials against SCE.

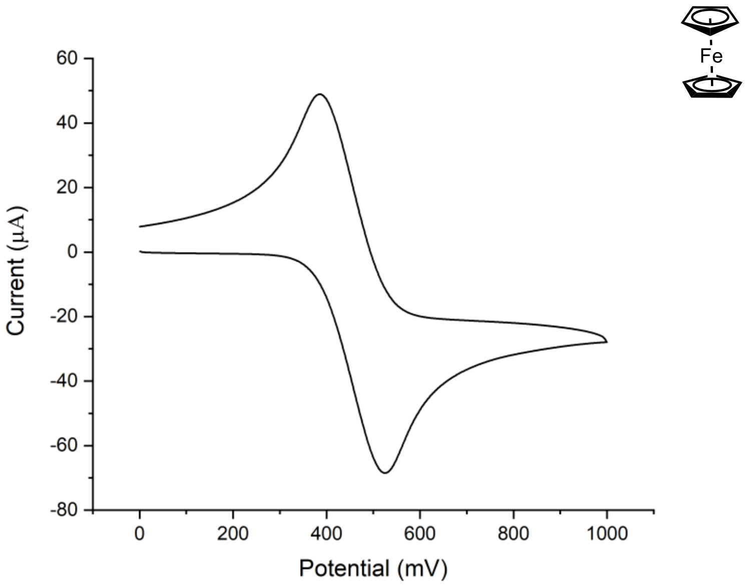

Figure S16. Cyclic voltammogram of ferrocene in MeCN. Scan rate 100 mV·s^{-1}.

Cyclic voltammograms were then recorded for all acridinium salts used in this study. To ensure consistency in evaluating and comparing different $E_{1/2}$ values, CV measurements were performed for all catalysts, including previously reported ones. Measurements were conducted on solutions of an acridinium salt (1.5 mM) in acetonitrile (3.0 mL) containing tetrabutylammonium tetrafluoroborate ($TBABF_4$, 45 mM) as the electrolyte, with scan rates of 100 or 500 mV/s. Prior to each measurement, the solution was purged with argon for 5 minutes to remove dissolved oxygen.

For *N*-H^+ acridiniums, partial dissociation in solution is possible and may affect the measured values. Based on reported pK_a values for acridine (12.66 in MeCN) and 9-chloroacridine (11.07 in MeCN),[34] we assume that, in the absence of water, dissociation of our samples in acetonitrile is negligible.

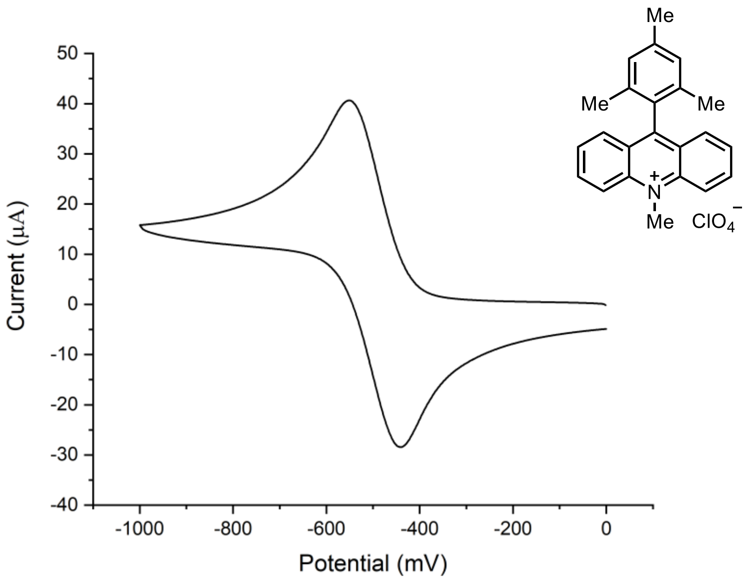

Figure S17. Cyclic voltammogram of Fukuzumi catalyst in MeCN. Scan rate 100 mV·s^{-1}.

The measured $E_{1/2}$ (Acr_{Fuk}^{+}/Acr_{Fuk}) = –0.496 V, which corresponds to –0.571 V versus SCE in MeCN.

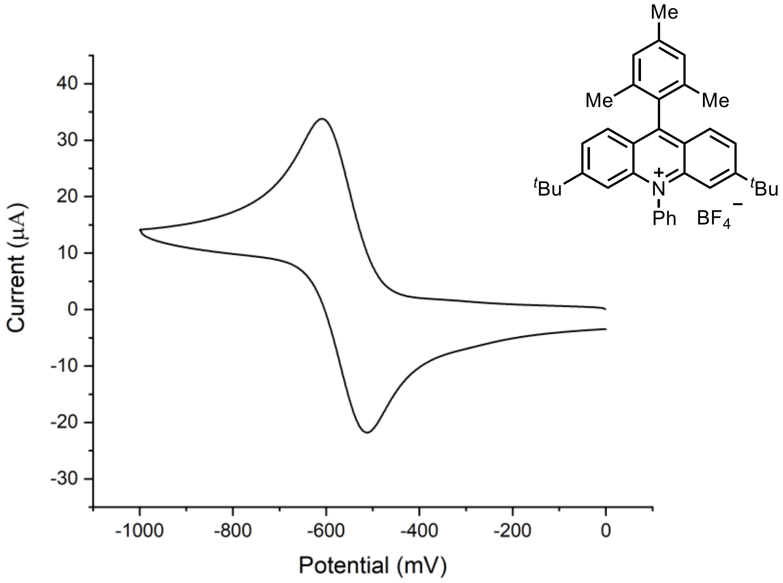

Figure S18. Cyclic voltammogram of Nicewicz catalyst in MeCN. Scan rate 100 mV·s^{-1}.

The measured $E_{1/2}$ (Acr_{Nic}^{+}/Acr_{Nic}) = –0.561 V, which corresponds to –0.636 V versus SCE in MeCN.

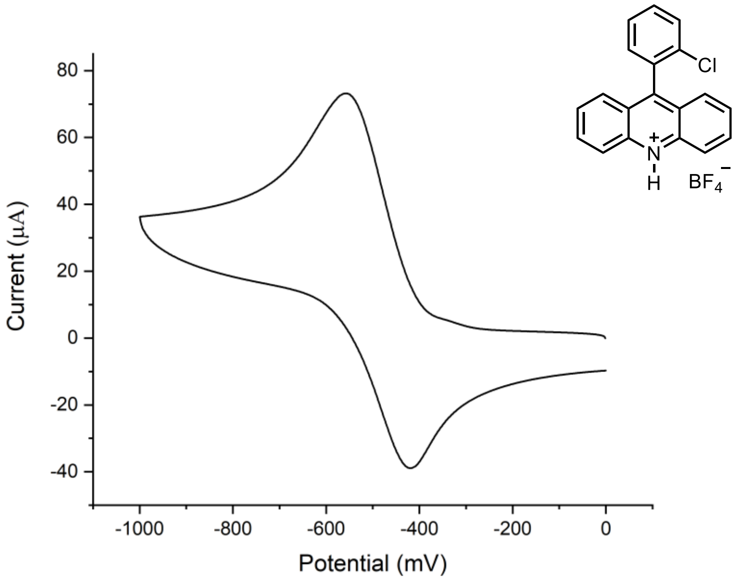

Figure S19. Cyclic voltammogram of **1**$H^+BF_4^-$ in MeCN. Scan rate 500 mV·s^{-1}.

The measured $E_{1/2}$ (**1**H^+/**1**H) = –0.488 V, which corresponds to –0.563 V versus SCE in MeCN.

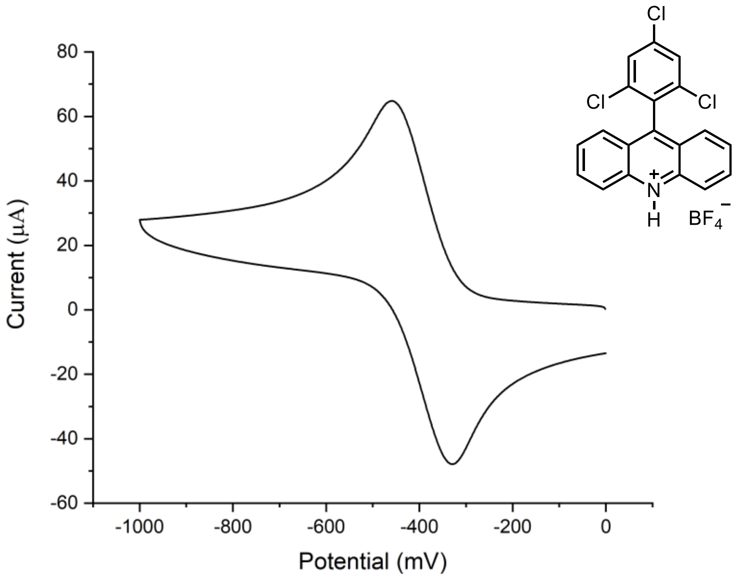

Figure S20. Cyclic voltammogram of **2**$H^+BF_4^-$ in MeCN. Scan rate 500 mV·s^{-1}.

The measured $E_{1/2}$ (**2**H^+/**2**H) = –0.394 V, which corresponds to –0.469 V versus SCE in MeCN.

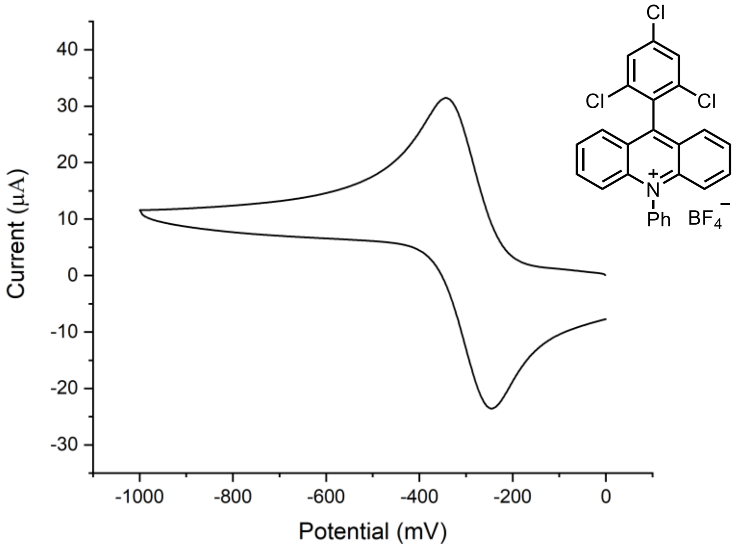

Figure S21. Cyclic voltammogram of **3**$^+BF_4^-$ in MeCN. Scan rate 100 mV·s^{-1}.

The measured $E_{1/2}$ (**3**$^+$/**3**) = –0.294 V, which corresponds to –0.369 V versus SCE in MeCN.

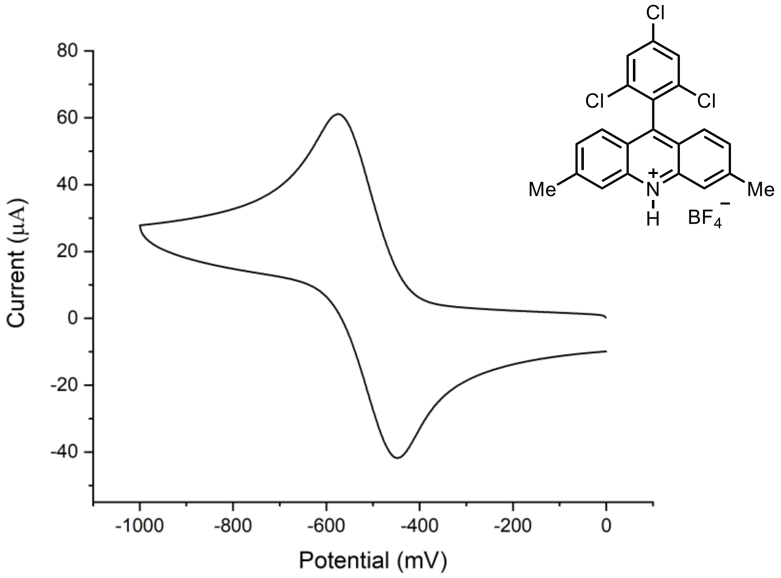

Figure S22. Cyclic voltammogram of **4**$H^+BF_4^-$ in MeCN. Scan rate 500 mV·s^{-1}.

The measured $E_{1/2}$ (**4**H^+/**4**H) = –0.512 V, which corresponds to –0.587 V versus SCE in MeCN.

Spectrophotometric titration of acridine 4

All UV-Vis spectra were recorded on a Shimadzu UV-2600 spectrophotometer equipped with a temperature controller, using a quartz cuvette (10 × 10 mm, 3.5 mL) with a PTFE stopper.

A 50 µM solution of acridine **4** was prepared in an acetone/water = 5/1 (v/v) mixture, and its UV-Vis spectrum

was recorded to establish a baseline. HBF_4 (prepared as 7.5 mM or 750 mM stock solutions in acetone/water = 5/1 (v/v)) was then incrementally added to a 3 mL aliquot of the acridine solution. After each addition, the solution was gently mixed, and the absorption spectrum was recorded after 30 seconds to allow equilibration. Titration continued until saturation was reached, as indicated by the absence of further changes in the absorption spectrum upon additional HBF_4 additions. The results are shown in Figure S23.

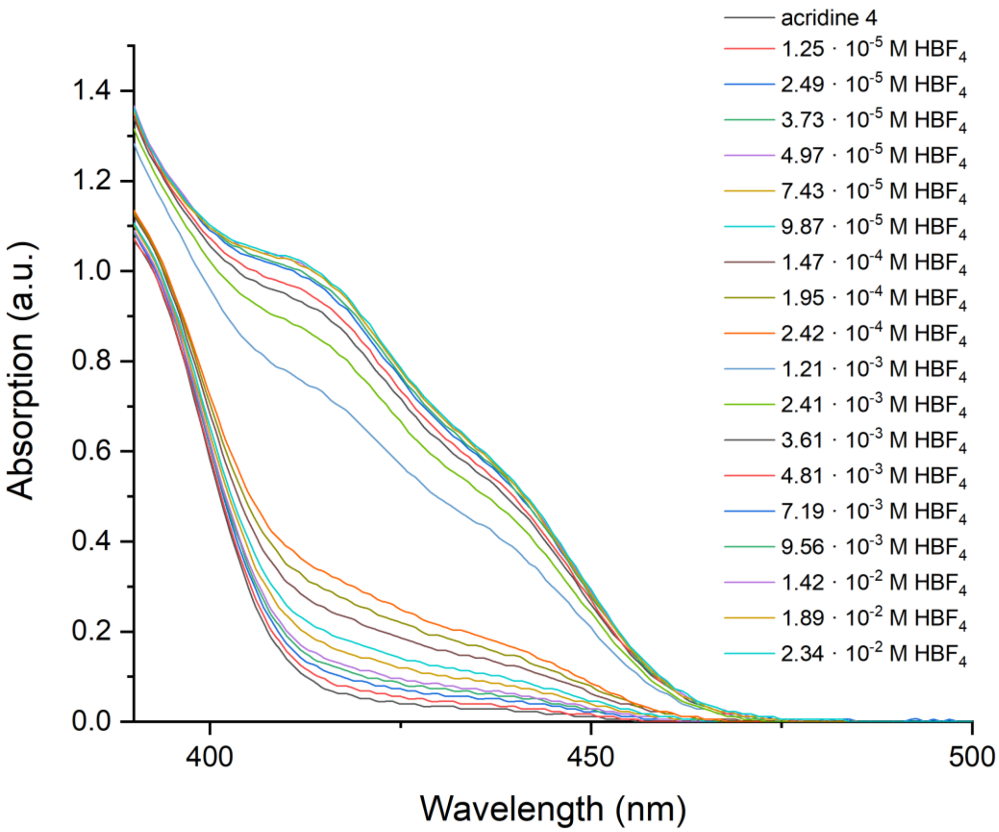

Figure S23. Normalized UV-Vis spectra for the spectrophotometric titration of acridine **4**.

Subsequently, absorption at λ = 414 nm where neutral acridine **4** absorbs minimally but acridinium has a local maximum was plotted against –log [HBF_4], as shown in Figure S24. Absorption in the equivalence point was determined as $A_{eq} = A_0 + ½ \cdot (A_{max} – A_0)$, where A_0 is the absorption in the absence of acridinium (100% acridine), A_{max} is the maximum absorption (100% acridinium). Applying the Henderson–Hasselbalch equation,

$$pK_a = -\log[H^+] - \log\frac{[Acr]}{[AcrH^+]}$$

pK_a was found as the value of –log [HBF_4] in the equivalence point. As such, pK_a of acridine **4** in acetone/water = 5/1 was identified to be 3.26.

Table S10. Results of acridine **4** spectrophotometric titration.

HBF_4 concentration (M)	–log [HBF_4]	Absorption at 414 nm (a.u.)
0	N.A.	0.085
$1.25 \cdot 10^{-5}$	4.90	0.102
$2.49 \cdot 10^{-5}$	4.60	0.124
$3.73 \cdot 10^{-5}$	4.43	0.136
$4.97 \cdot 10^{-5}$	4.30	0.153
$7.43 \cdot 10^{-5}$	4.13	0.181
$9.87 \cdot 10^{-5}$	4.01	0.209
$1.47 \cdot 10^{-4}$	3.83	0.260
$1.95 \cdot 10^{-4}$	3.71	0.305
$2.42 \cdot 10^{-4}$	3.62	0.339
$1.21 \cdot 10^{-3}$	2.92	0.740
$2.41 \cdot 10^{-3}$	2.62	0.853
$3.61 \cdot 10^{-3}$	2.44	0.915
$4.81 \cdot 10^{-3}$	2.32	0.938
$7.19 \cdot 10^{-3}$	2.14	0.972
$9.56 \cdot 10^{-3}$	2.02	0.977
$1.42 \cdot 10^{-2}$	1.85	0.994
$1.89 \cdot 10^{-2}$	1.72	0.994
$2.34 \cdot 10^{-2}$	1.63	1.000

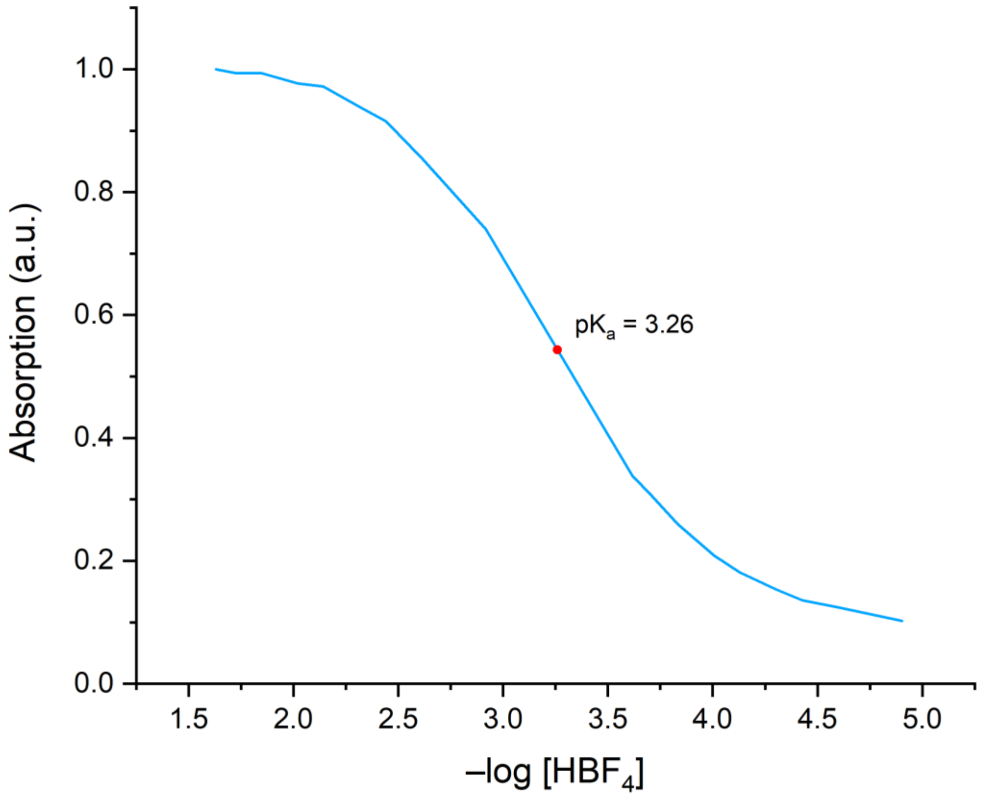

Figure S24. Titration curve of acridine **4** in acetone/water = 5/1 (v/v).

Absorption and emission spectra of acridinium salts

UV-Vis spectra were recorded on a Shimadzu UV-2600 spectrophotometer equipped with a temperature controller, using a quartz cuvette (10 × 10 mm, 3.5 mL) with a PTFE stopper. All samples were measured at a concentration of 50 μM in acetonitrile as solvent.

For *N*-H^+ acridiniums, partial dissociation in solution is possible and may affect the measured spectra. Based on reported pK_a values for acridine (12.66 in MeCN) and 9-chloroacridine (11.07 in MeCN),[34] we assume that, in the absence of water, dissociation of our samples in acetonitrile is negligible.

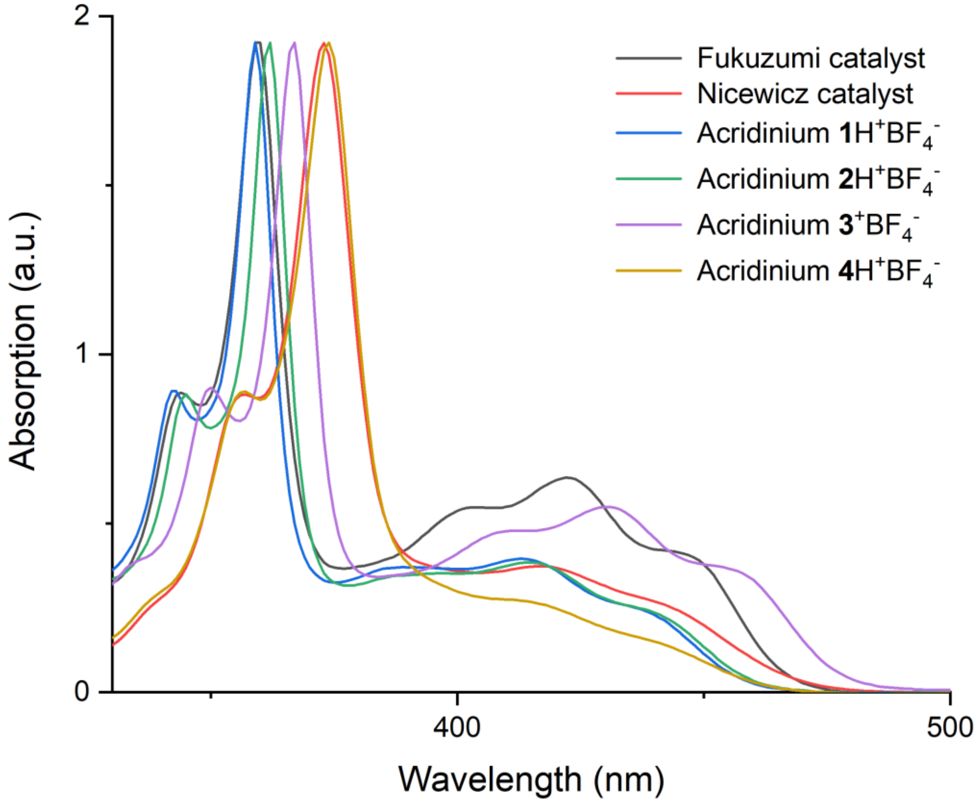

Figure S25. Normalized UV-Vis spectra of acridinium salts (50 µM) in MeCN.

Emission spectra were recorded on an Edinburgh Instruments FS5 spectrofluorometer, using a quartz cuvette (Hellma fluorescence quartz cuvette, 10 × 10 mm, 3.5 mL) with a PTFE stopper in acetonitrile as solvent. All samples were measured at a concentration of 10 µM.

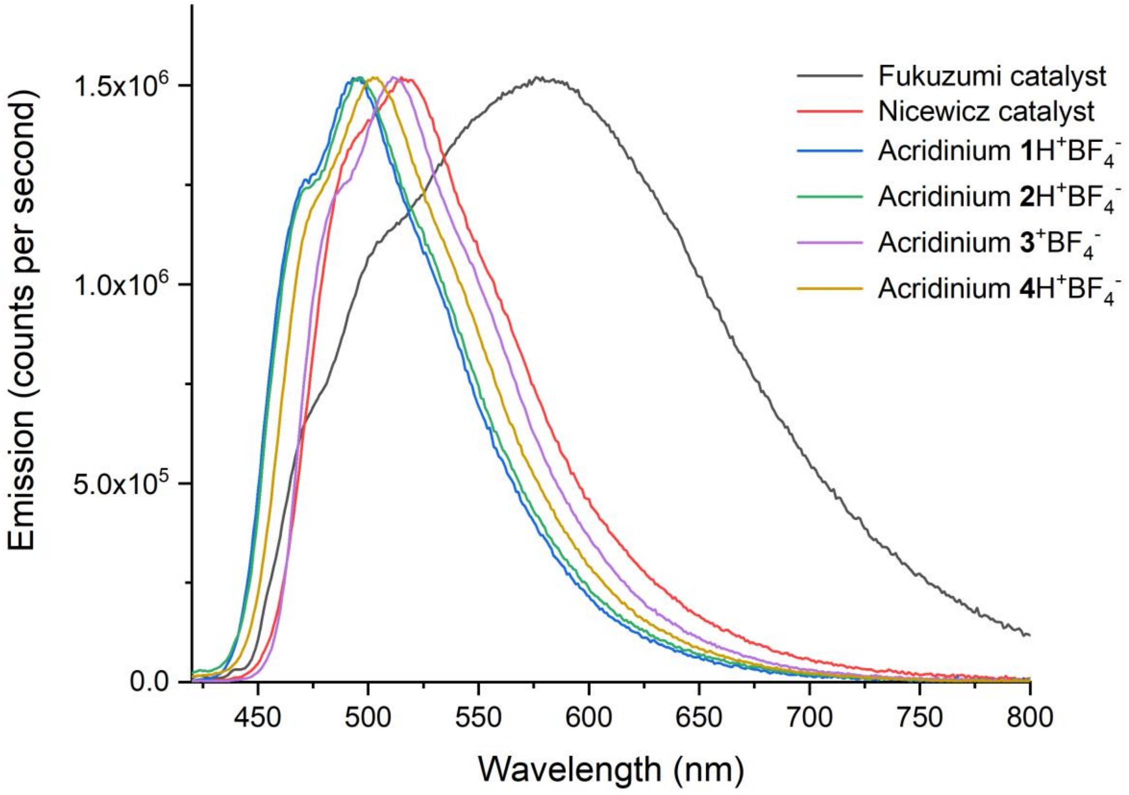

Figure S26. Normalized emission spectra of acridinium salts (10 μM) in MeCN.

Determination of excited-state reduction potentials of acridinium salts

Excited-state reduction potentials (E^*_{red}) were calculated from the ground-state half-wave reduction potential ($E_{1/2}$) and the excitation energy ($E_{0,0}$). $E_{0,0}$ values were determined as the energy of the wavelength at which the substrate's normalized UV-Vis absorption and emission spectra intersect.[35] To ensure consistency in evaluating and comparing different E^*_{red} values, $E_{0,0}$ values were determined for all catalysts, including previously reported ones.

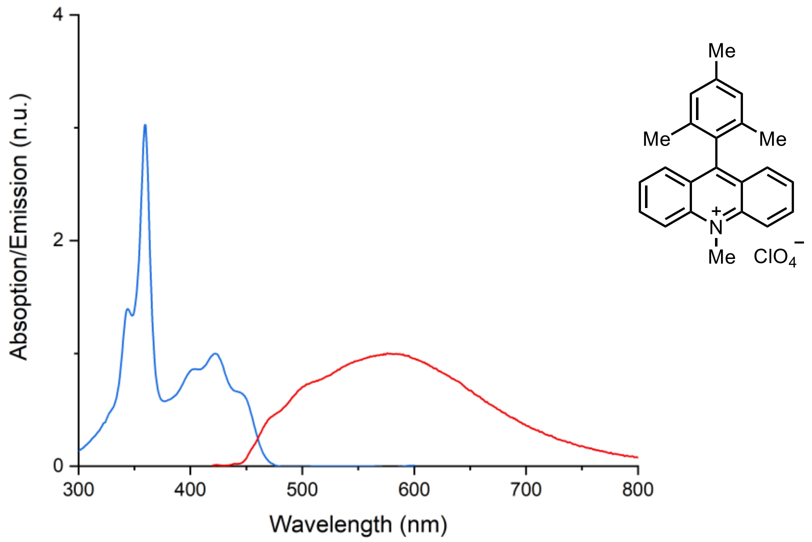

Figure S27. Normalized UV-Vis (blue) and emission (red) spectra of Fukuzumi catalyst in MeCN.

Both spectra intersect at the wavelength of 460 nm, which corresponds to the $E_{0,0}$ of 2.696 eV. Therefore, E^*_{red} (Acr_{Fuk}^+/Acr_{Fuk}) = 2.696 + (–0.571) = 2.125 V versus SCE in MeCN.

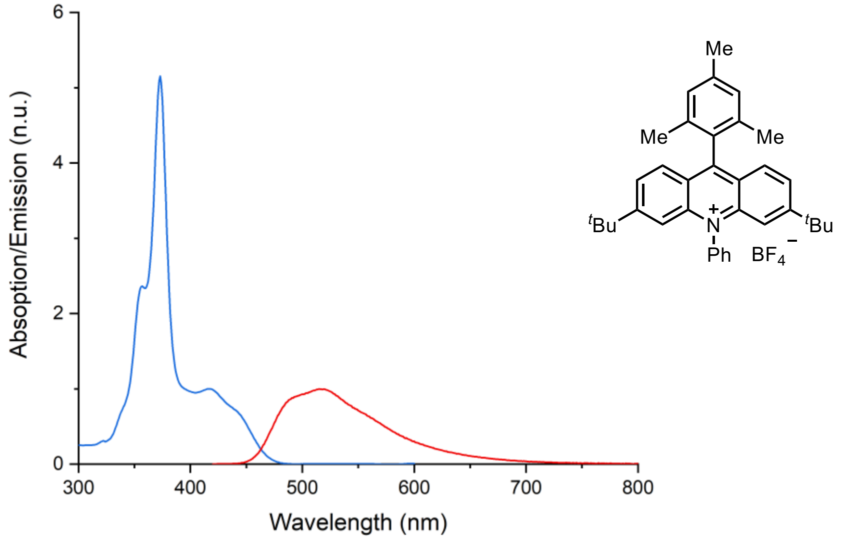

Figure S28. Normalized UV-Vis (blue) and emission (red) spectra of Nicewicz catalyst in MeCN.

Both spectra intersect at the wavelength of 464 nm, which corresponds to the $E_{0,0}$ of 2.672 eV. Therefore, E^*_{red} (Acr_{Nic}^+/Acr_{Nic}) = 2.672 + (–0.636) = 2.036 V versus SCE in MeCN.

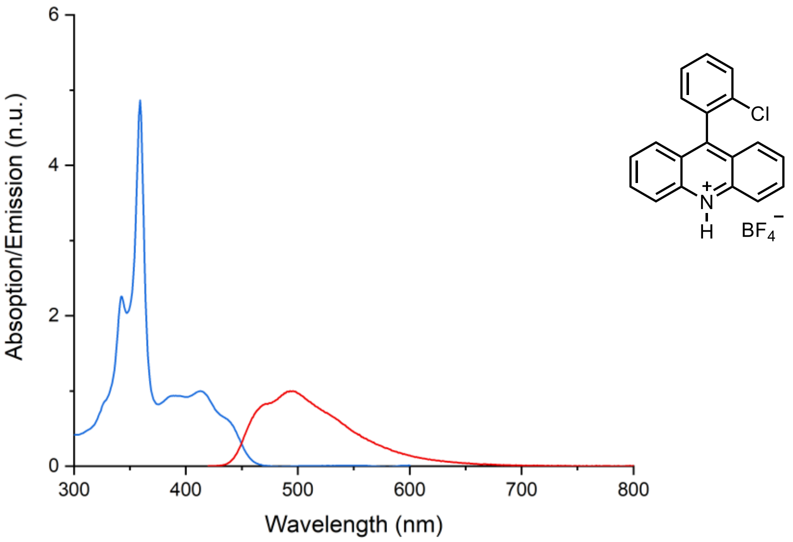

Figure S29. Normalized UV-Vis (blue) and emission (red) of **1**$H^+BF_4^-$ in MeCN.

Both spectra intersect at the wavelength of 450 nm, which corresponds to the $E_{0,0}$ of 2.756 eV. Therefore, E^*_{red} (**1**H^+/**1**H) = 2.756 + (–0.563) = 2.193 V versus SCE in MeCN.

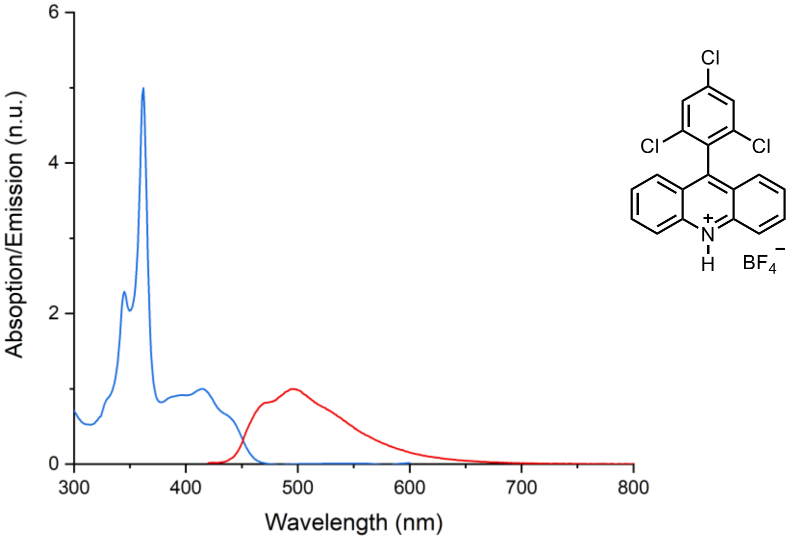

Figure S30. Normalized UV-Vis (blue) and emission (red) of **2**$H^+BF_4^-$ in MeCN.

Both spectra intersect at the wavelength of 451 nm, which corresponds to the $E_{0,0}$ of 2.749 eV. Therefore, E^*_{red} (**2**H^+/**2**H) = 2.749 + (–0.469) = 2.280 V versus SCE in MeCN.

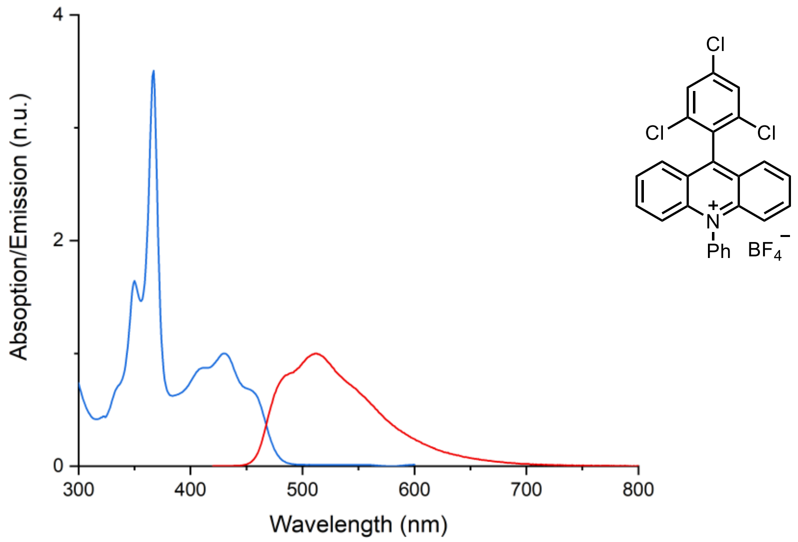

Figure S31. Normalized UV-Vis (blue) and emission (red) of **3**$^+$BF$_4^-$ in MeCN.

Both spectra intersect at the wavelength of 468 nm, which corresponds to the $E_{0,0}$ of 2.650 eV. Therefore, E^*_{red} (**3**$^+$/**3**) = 2.650 + (–0.369) = 2.281 V versus SCE in MeCN.

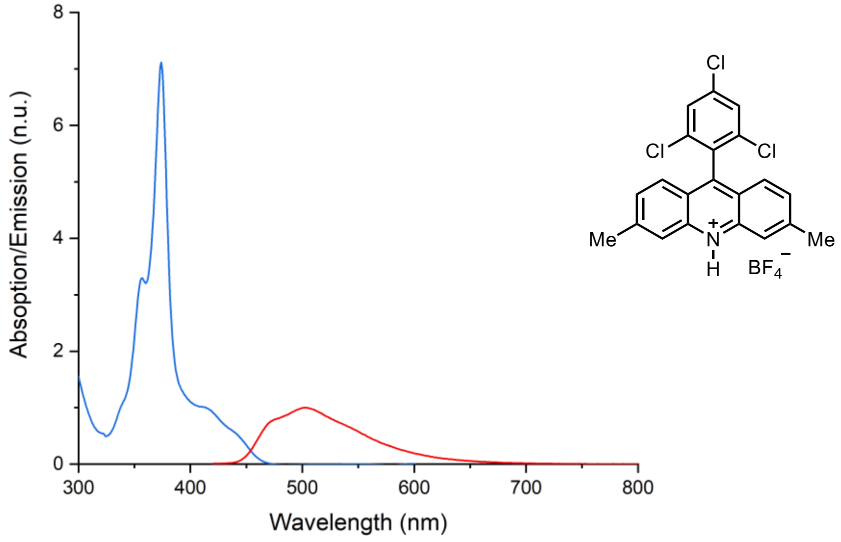

Figure S32. Normalized UV-Vis (blue) and emission (red) spectra of **4**H$^+$BF$_4^-$ in MeCN.

Both spectra intersect at the wavelength of 453 nm, which corresponds to the $E_{0,0}$ of 2.737 eV. Therefore, E^*_{red} (**4**H$^+$/**4**H) = 2.737 + (–0.587) = 2.150 V versus SCE in MeCN.

Table S11. Photophysical properties of the acridinium salts. Summary.

Catalyst	$E_{1/2}$ (vs. SCE), V	$E_{0,0}$, eV	E^*_{red} (vs. SCE), V
Fukuzumi catalyst	–0.571	2.696	2.13
Nicewicz catalyst	–0.636	2.672	2.04
**1**$H^+BF_4^-$	–0.563	2.756	2.19
**2**$H^+BF_4^-$	–0.469	2.749	2.28
**3**$^+BF_4^-$	–0.369	2.650	2.28
**4**$H^+BF_4^-$	–0.587	2.737	2.15

DFT calculations

All density functional theory (DFT) calculations were performed on the Max-Planck-Institut für Kohlenforschung computer cluster using the ORCA program package (Version 5.0-Stable).[36] Specifically, the (U)ωB97X functional[37,38] with D4 dispersion correction[39,40] and def2-TZVP(-f) basis set[41] were used to conduct geometry optimization and frequency calculation. Single-point energy calculations were carried out on the optimized geometries using the (U)DLPNO-CCSD(T) method[42-45] and def2-QZVPP basis set.[41] The solvation effect was considered using the SMD solvation model[47] (solvent: acetonitrile). The libint2 library was used for the computation of 2-el integrals.[48] The RIJCOSX approximation with the def2/J[49] and def2-TZVP/C or def2-QZVPP/C[50] auxiliary basis set were applied. Very tight SCF convergence criteria were set for all calculations. All structures were confirmed to be at local minima (ground state) by the absence of imaginary frequencies. Images were generated using Chemcraft 1.8.[51]

Alkene oxidation potentials series

To investigate the oxidation potentials of various alkenes, we employed a combination of experimental and computational approaches. Experimental data were obtained through cyclic voltammetry, but the operational range of the available instrument limited direct measurements to oxidation potentials below approximately 2.1 V. To estimate oxidation potentials exceeding this range, density functional theory (DFT) calculations were performed.

The numerical values of the DFT-computed oxidation potentials were derived according to the method described by Nicewicz and co-authors.[52] For each alkene, geometry optimizations were performed for both its reduced form (neutral molecule) and its oxidized form (radical cation). Subsequently, Gibbs free energies at 298 K were calculated for each species. The final oxidation potential relative to the saturated calomel electrode (SCE) was calculated using the following equation:

$$E_{CALC}^{ox} \frac{-\left(G_{298}\,(reduced\ form) - G_{298}\,(oxidized\ form)\right)}{nF} - E_{1/2}^{SHE} + E_{1/2}^{SCE}$$

Where $G_{298}\,(reduced\ form)$ is the Gibbs free energy of the neutral alkene at 298K, $G_{298}\,(oxidized\ form)$ is the Gibbs free energy of the alkene's radical cation at 298K, n is the number of electrons transferred ($n = 1$ in all cases), F is the Faraday constant ($F = 96485.33\ \mathrm{C \cdot mol^{-1}}$), $E_{1/2}^{SHE}$ is the absolute potential of the standard

hydrogen electrode ($E_{1/2}^{SHE} = 4.281\ V$),[50] and $E_{1/2}^{SCE}$ is the conversion factor between the standard hydrogen and saturated calomel electrodes ($E_{1/2}^{SCE} = -0.141\ V$ in acetonitrile).[53]

To ensure the reliability of computational predictions and their alignment with experimentally obtained values, an empirical correlation was established between the two datasets. A representative subset of alkenes, for which both experimental and computational oxidation potentials could be determined, was selected.

Table S12. Calculated and experimental oxidation potentials of a number of alkenes.

Entry	Structure	Name	E_{CALC} (vs. SCE), V	E_{EXP} (vs. SCE), V
1	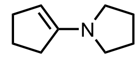	1-Pyrrolidino-1-cyclopentene	0.18	0.26
2	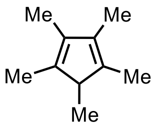	Pentamethylcyclopentadiene	0.77	0.88
3	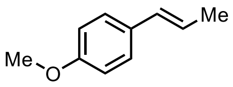	trans-Anethole	1.18	1.21
4	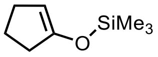	1-(Trimethylsiloxy)cyclopentene	1.20	1.25
5	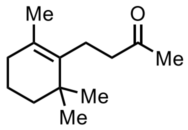	Dihydro-β-ionone	1.48	1.54
6	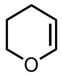	Dihydropyran	1.42	1.54
7	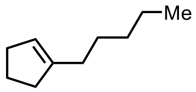	1-Pentyl-1-cyclopentene	1.69	1.81
8	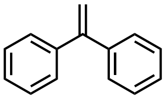	1,1-Diphenylethylene	1.78	1.85
9	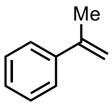	α-Methylstyrene	1.82	1.89
10	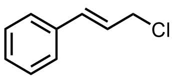	Cinnamylchloride	1.85	1.91
11	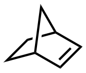	Norbornene	1.91	1.98
12	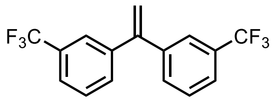	1,1-bis(3-(trifluoromethyl)phenyl)ethene	2.00	2.10

The experimentally measured values were plotted against the corresponding DFT-calculated values, and a linear regression analysis was conducted. This analysis yielded a correlation curve, spanning the

experimental range of 0.2 – 2.1 V (coefficient of determination, $R^2 > 0.99$).

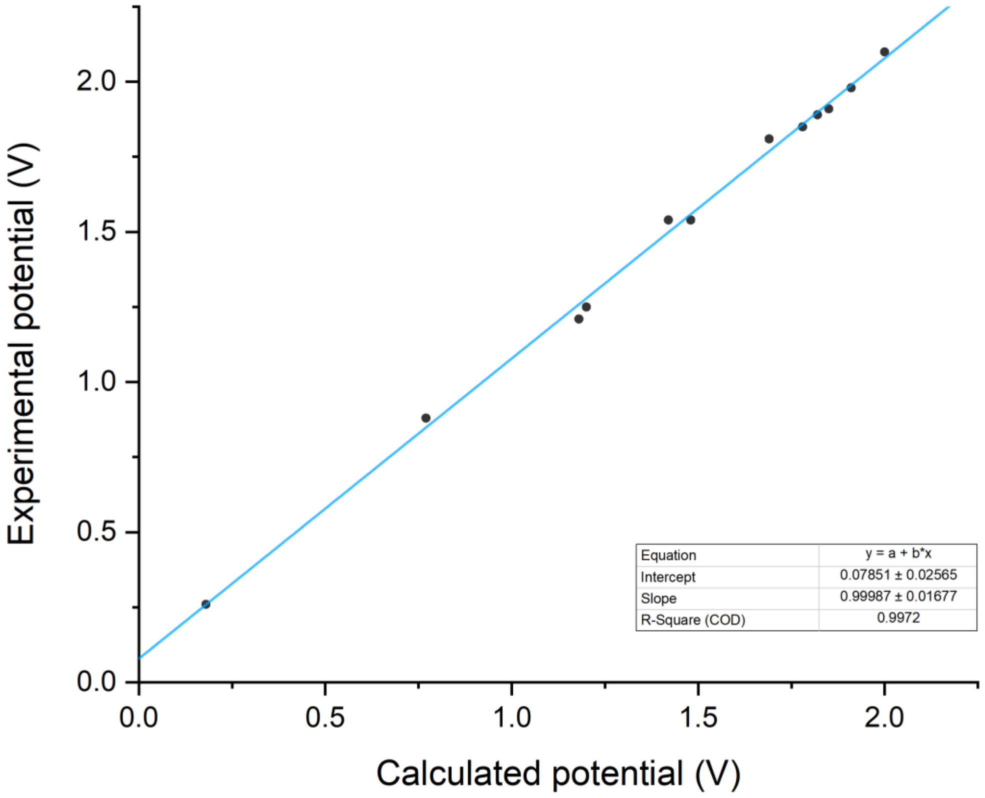

Figure S33. Alkenes' calculated and experimental oxidation potentials approximated by a linear function.

The obtained regression equation, $y = 0.99987x + 0.07851$, where y represents the experimentally measured value and x the DFT-calculated potential, was used to predict experimental oxidation potentials (E_{PRED}) for alkenes with values beyond the measurable range.

$$E_{PRED} = \ 0.99987 \cdot E_{calc} + 0.07851$$

This approach assumes that the correlation derived within the measurable range is consistent and remains valid for higher oxidation potentials (> 2.1 V).

Table S13. Predicted oxidation potentials of a number of alkenes.

Entry	Structure	Name	E_{CALC} (vs. SCE), V	E_{PRED} (vs. SCE), V
1	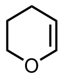	Dihydropyran	1.42	1.50
2	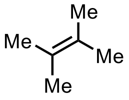	2,3-Dimethyl-2-butene	1.44	1.52

3	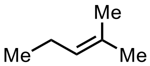	2-Methyl-2-pentene	1.71	1.79
4	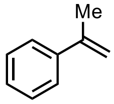	α-Methylstyrene	1.82	1.90
5	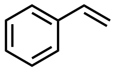	Styrene	1.86	1.94
6	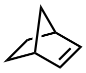	Norbornene	1.91	1.99
7	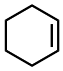	Cyclohexene	1.97	2.05
8	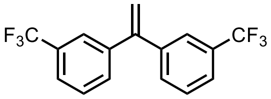	1,1-bis(3-(trifluoromethyl)phenyl)ethene	2.00	2.08
9	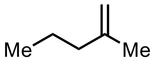	2-Methyl-1-pentene	2.08	2.16
10	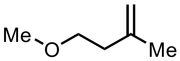	4-Methoxy-2-methylbut-1-ene	2.16	2.24
11	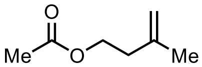	3-Methylbut-3-en-1-yl acetate	2.25	2.33
12	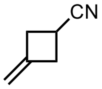	3-Methylenecyclobutanecarbonitrile	2.35	2.43
13	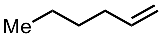	1-Hexene	2.42	2.50

To maintain consistency in the comparison of oxidation potentials across various alkenes, the predicted experimental values (E_{PRED}) derived from the regression equation were uniformly employed throughout this study, even for alkenes where direct experimental values were available.

Analysis of the hydration reaction with alkenes containing electron-rich aromatic rings

A natural question arising from the oxidative nature of the developed catalyst **4**H^+ was whether the hydration reaction could tolerate substrates bearing electron-rich functional groups. To explore this, we examined the reaction of osthol (**5q**), an alkene bearing an electron-rich aromatic system. The reaction successfully produced alcohol **6q** in 80% yield, but achieving near full conversion required the increase in TRIPSH loading to 10 mol% (from the standard 5 mol%) and doubling the reaction time to 36 hours (from 18 hours). These adjustments were unexpected, as the alkene was not electron-deficient and was anticipated to react efficiently under standard conditions.

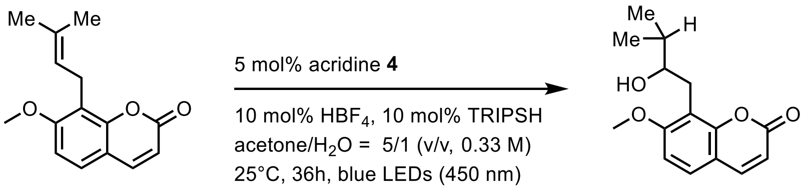

5q **6q**, 80% (r.r. 2.8:1)

Computational analysis of **5q** revealed that its highest occupied molecular orbital (HOMO) is predominantly localized on the aromatic ring and the conjugated system, rather than on the double bond. Instead, HOMO-1 is located on the double bond.

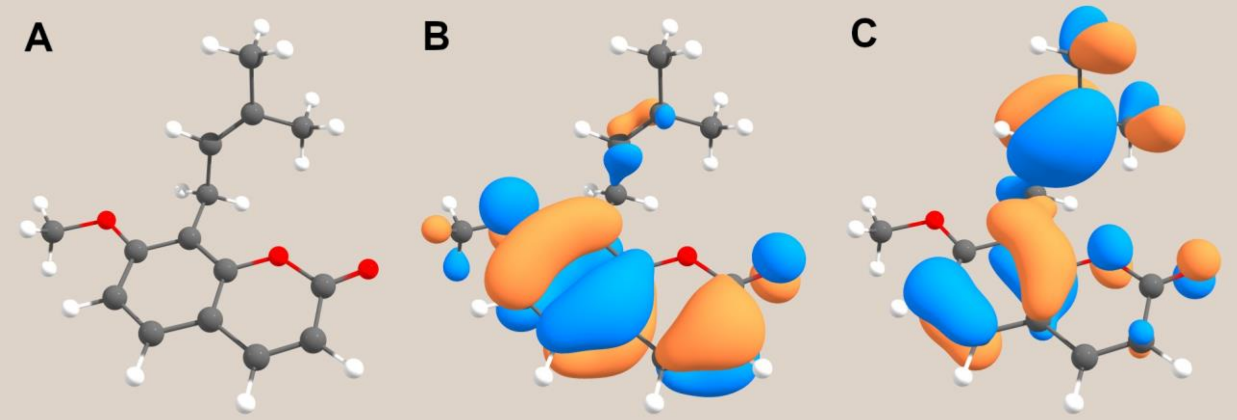

Figure S34. Substrate **5q** (A) and its highest molecular orbitals: HOMO (B) and HOMO-1 (C).

The oxidation potential of **5q**, as predicted according to the regression equation derived before, was determined to be E_{PRED} = 1.67 V. For comparison, a structurally related compound lacking the alkene moiety in the side chain, 7-methoxy-8-methyl-2H-chromen-2-one, displayed a nearly identical oxidation potential E_{PRED} = 1.65 V, while a similar alkene without an aromatic ring (2-methylpent-2-ene) was shown to have a higher oxidation potential E_{PRED} = 1.79 V. Collectively, these data suggest that the oxidation of the aromatic ring in **5q** is more favorable than the oxidation of the alkene.

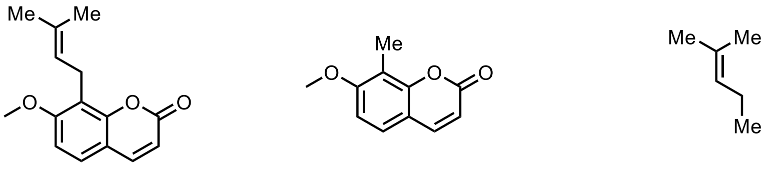

5q — E_{PRED} = 1.67 V; 7-methoxy-8-methyl-2*H*-chromen-2-one — E_{PRED} = 1.65 V; 2-methylpent-2-ene — E_{PRED} = 1.79 V

Given that the potential of the acridinium catalyst **4**H^+ (E^*_{red} = 2.15 V) used in the reaction is significantly higher than any of these values, oxidation is possible for both the aromatic and alkene fragments. Thus, we hypothesize that the prolonged reaction time is due to the presence of a competing oxidation process involving the aromatic system. This process does not appear to yield any detectable chemical products under the reaction conditions but likely inhibits the desired transformation.

While the reaction of the trisubstituted alkene **5q** was successful, disubstituted alkenes presented greater

challenge. Disubstituted alkenes typically exhibit higher oxidation potentials compared to their trisubstituted counterparts, suggesting that their oxidation would occur at significantly lower rates compared to the oxidation of electron-rich aromatic systems. In this study, the reaction of the disubstituted alkene **5k** under standard conditions successfully afforded product **6k** in 86% yield.

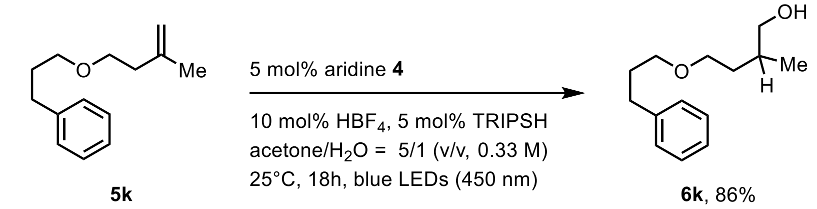

However, the introduction of an alkene with a more electron-rich aromatic ring, such as **10**, led to unsatisfactory results. The reaction of **10** to form **11** progressed slowly, with only 10% yield of the desired alcohol observed after 18 hours of irradiation, as determined by ^{1}H NMR spectroscopic analysis of the crude reaction mixture. No by-products were detected, and 88% of the starting material **10** remained unreacted.

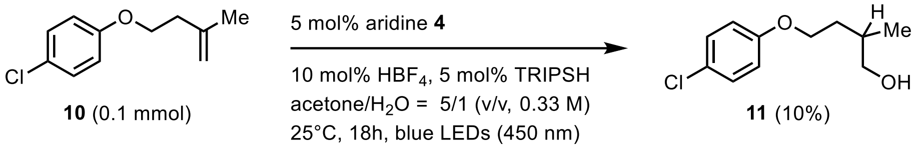

This outcome can be rationalized by comparing the predicted oxidation potentials of the aromatic system in **10** to that of a representative disubstituted alkene, 4-methoxy-2-methylbut-1-ene.

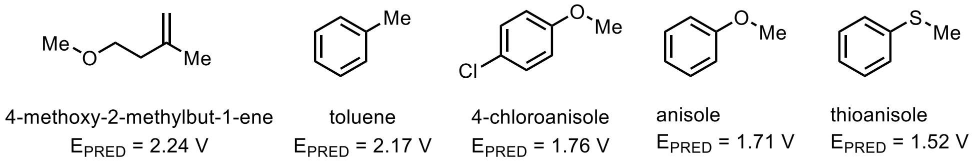

The oxidation potential of 4-chloroanisole (a structural analog of the aromatic ring in **10**) is approximately 0.5 V lower than that of 4-methoxy-2-methylbut-1-ene. Assuming that single-electron transfer between acridinium catalyst **4**H$^+$ and the substrate is an equilibrium process,

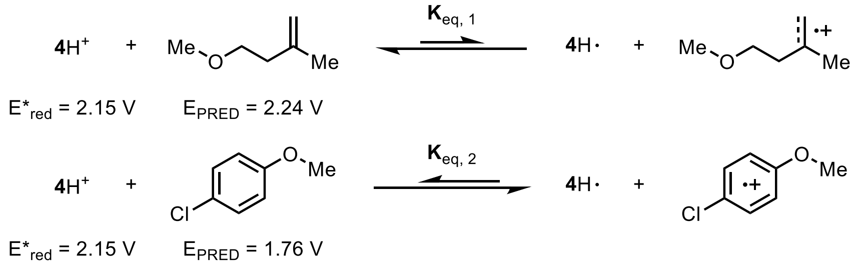

the equilibrium constant for the oxidation of 4-chloroanisole ($K_{eq,2}$) relative to that of the alkene ($K_{eq,1}$) can be expressed as:

$$\frac{K_{eq,2}}{K_{eq,1}} = e^{-nF\Delta E/_{RT}} = e^{18.68} \approx 1.3 \cdot 10^8$$

The difference in oxidation potentials of 0.48 V means that oxidation of 4-chloroanisole is approximately 130 million times more thermodynamically favorable than oxidation of 4-methoxy-2-methylbut-1-ene, which explains the experimentally observed stalling of the hydration reaction of **10**.

Cartesian coordinates and the Gibbs free energies of the DFT-optimized structures

1-Pyrrolidino-1-cyclopentene (neutral)

G_{298} = –405.911540 Hartree

C	1.680041000	-1.797070000	1.836131000
C	0.540479000	-1.241616000	2.713861000
C	0.018133000	-0.006853000	1.960996000
C	0.414013000	-0.290412000	0.527602000
C	1.321454000	-1.284985000	0.459986000
N	-0.050105000	0.490331000	-0.498987000
C	-1.349487000	1.178641000	-0.440373000
C	-1.801533000	1.251594000	-1.903548000
C	-0.494484000	1.161002000	-2.695111000
C	0.320637000	0.170434000	-1.870975000
H	1.738305000	-2.889079000	1.889842000
H	2.655610000	-1.414270000	2.166760000
H	0.857549000	-1.010871000	3.733229000
H	-0.259655000	-1.985063000	2.774190000
H	-1.056244000	0.138996000	2.089066000
H	0.511242000	0.915242000	2.292852000
H	1.811304000	-1.619759000	-0.447026000
H	-1.232049000	2.178505000	-0.008329000
H	-2.067911000	0.629579000	0.172456000
H	-2.432791000	0.389938000	-2.138746000
H	-2.371051000	2.158412000	-2.114192000
H	0.009020000	2.132166000	-2.714494000
H	-0.640519000	0.827734000	-3.724016000

H	0.052937000	-0.868104000	-2.120549000
H	1.399942000	0.279017000	-2.011713000

1-Pyrrolidino-1-cyclopentene (radical cation)

G_{298} = –405.742453 Hartree

C	1.761615000	-1.676367000	1.851329000
C	0.563973000	-1.248936000	2.722486000
C	-0.121175000	-0.113241000	1.940321000
C	0.310046000	-0.357861000	0.520530000
C	1.383808000	-1.258847000	0.477944000
N	-0.226428000	0.223969000	-0.522568000
C	-1.336650000	1.190638000	-0.441136000
C	-1.764949000	1.370282000	-1.894141000
C	-0.466121000	1.155647000	-2.674609000
C	0.214461000	0.018987000	-1.917304000
H	1.994395000	-2.742612000	1.911563000
H	2.679736000	-1.141194000	2.135637000
H	0.864845000	-0.938058000	3.722594000
H	-0.127692000	-2.088138000	2.821937000
H	-1.206435000	-0.108884000	2.046499000
H	0.245002000	0.873709000	2.243391000
H	1.893681000	-1.569360000	-0.424541000
H	-0.945360000	2.117036000	-0.011188000
H	-2.118290000	0.800450000	0.209735000
H	-2.497908000	0.605090000	-2.162201000
H	-2.208394000	2.351428000	-2.061936000
H	0.155662000	2.053464000	-2.634075000
H	-0.634220000	0.894892000	-3.719359000
H	-0.140628000	-0.961312000	-2.245004000
H	1.301860000	0.042727000	-1.966991000

Pentamethylcyclopentadiene (neutral)

G_{298} = –389.878300 Hartree

C	-1.070998000	0.285464000	0.456175000
C	-0.322950000	0.960876000	-0.635945000
C	0.930819000	0.479313000	-0.676178000
C	1.096342000	-0.561069000	0.405713000
C	-0.263018000	-0.600464000	1.063083000
C	-2.498165000	0.617480000	0.767106000
C	-0.952565000	2.002349000	-1.509351000
C	2.047814000	0.836478000	-1.604861000
C	-0.561429000	-1.522885000	2.202919000
C	1.564771000	-1.919360000	-0.122885000
H	1.836159000	-0.204140000	1.137238000
H	-2.600824000	1.667914000	1.059470000
H	-3.137255000	0.468998000	-0.109305000
H	-2.890852000	0.002223000	1.578442000
H	-0.247872000	2.398564000	-2.242266000
H	-1.808856000	1.590407000	-2.053654000
H	-1.328394000	2.840413000	-0.913337000
H	2.953144000	1.100603000	-1.047569000
H	2.309921000	-0.007906000	-2.252265000
H	1.789827000	1.680385000	-2.247331000
H	-1.540042000	-1.323663000	2.643636000
H	-0.548903000	-2.570106000	1.879726000
H	0.190851000	-1.427614000	2.993368000
H	1.642808000	-2.648841000	0.687743000
H	2.547986000	-1.836942000	-0.593745000
H	0.861687000	-2.308476000	-0.865927000

Pentamethylcyclopentadiene (radical cation)

G_{298} = –389.687637 Hartree

C	-1.078834000	0.322268000	0.425952000
C	-0.366041000	0.958179000	-0.620299000
C	0.938117000	0.466663000	-0.649757000
C	1.118501000	-0.552480000	0.430573000
C	-0.237511000	-0.587269000	1.065523000
C	-2.498443000	0.624751000	0.752680000
C	-0.963899000	1.976709000	-1.524329000
C	2.011229000	0.825931000	-1.595151000
C	-0.561973000	-1.493918000	2.182332000
C	1.612330000	-1.912123000	-0.076180000
H	1.840185000	-0.164929000	1.162413000
H	-2.581944000	1.652566000	1.120103000
H	-3.113675000	0.555608000	-0.148556000
H	-2.899770000	-0.049939000	1.507210000
H	-0.229197000	2.402598000	-2.205830000
H	-1.764963000	1.518949000	-2.113304000
H	-1.417339000	2.780898000	-0.937910000
H	2.988963000	0.759946000	-1.113063000
H	2.007327000	0.099760000	-2.419302000
H	1.869837000	1.817777000	-2.024269000
H	-1.490765000	-1.229702000	2.685432000
H	-0.657565000	-2.514958000	1.790898000
H	0.260738000	-1.511646000	2.902764000
H	1.705364000	-2.612912000	0.755163000
H	2.592802000	-1.805906000	-0.543573000
H	0.916530000	-2.326822000	-0.809520000

trans-Anethole (neutral)

G_{298} = –462.641030 Hartree

C	-1.899823000	0.200867000	0.032112000
C	-1.825729000	-1.196155000	0.001608000
C	-0.579687000	-1.813220000	0.001785000
C	0.571205000	-1.028201000	0.028068000
C	0.519373000	0.362322000	0.050554000
C	-0.748345000	0.961863000	0.057383000
O	-3.014745000	-1.855962000	-0.021785000
C	-2.971547000	-3.280600000	-0.045874000
C	1.780905000	1.130046000	0.066934000
C	1.913293000	2.449587000	-0.063962000
C	3.227735000	3.169157000	-0.039985000
H	-2.876428000	0.673348000	0.040246000
H	-0.486677000	-2.891117000	-0.017061000
H	1.539035000	-1.521184000	0.028407000
H	-0.839433000	2.042137000	0.089901000
H	-4.009237000	-3.612410000	-0.062111000
H	-2.460140000	-3.645844000	-0.941701000
H	-2.478152000	-3.676606000	0.846994000
H	2.680938000	0.528437000	0.189275000
H	1.029842000	3.070288000	-0.201556000
H	4.060367000	2.475099000	0.099123000
H	3.387943000	3.719874000	-0.973385000
H	3.253385000	3.907703000	0.768704000

trans-Anethole (radical cation)

G_{298} = –462.435005 Hartree

C	-1.871106000	0.237647000	-0.000480000
C	-1.789818000	-1.184585000	-0.000318000
C	-0.529160000	-1.832106000	-0.000071000
C	0.599500000	-1.069060000	0.000029000

C	0.547061000	0.357107000	-0.000114000
C	-0.739052000	0.983674000	-0.000384000
O	-2.941212000	-1.813741000	-0.000418000
C	-2.966879000	-3.260478000	-0.000262000
C	1.770658000	1.073107000	0.000021000
C	1.883145000	2.433827000	-0.000055000
C	3.179879000	3.148443000	0.000089000
H	-2.853362000	0.695042000	-0.000679000
H	-0.460221000	-2.910901000	0.000038000
H	1.572463000	-1.548000000	0.000224000
H	-0.816014000	2.063121000	-0.000517000
H	-4.021292000	-3.525963000	-0.000359000
H	-2.481228000	-3.641653000	-0.899205000
H	-2.481443000	-3.641458000	0.898879000
H	2.681344000	0.480108000	0.000205000
H	0.988658000	3.049365000	-0.000228000
H	4.029778000	2.465158000	0.000281000
H	3.236291000	3.805334000	-0.875689000
H	3.236016000	3.805487000	0.875769000

1-(Trimethylsiloxy)cyclopentene (neutral)

G_{298} = –678.104347 Hartree

C	1.635295000	-1.201457000	2.227552000
C	0.697609000	-0.441857000	3.196043000
C	-0.281647000	0.333118000	2.295628000
C	-0.194622000	-0.419950000	0.994612000
C	0.834398000	-1.264995000	0.949326000
O	-1.119380000	-0.165047000	0.026248000
Si	-0.692321000	0.458257000	-1.480448000
C	-2.326993000	0.846296000	-2.284959000

C	0.342587000	1.988721000	-1.212173000
C	0.250861000	-0.804248000	-2.481958000
H	1.904246000	-2.190005000	2.611409000
H	2.574529000	-0.656443000	2.069239000
H	1.241535000	0.205807000	3.886110000
H	0.138291000	-1.167100000	3.793215000
H	-1.303192000	0.352818000	2.685108000
H	0.026217000	1.373902000	2.136126000
H	1.099509000	-1.880378000	0.098020000
H	-2.173203000	1.258751000	-3.287335000
H	-2.936310000	-0.057678000	-2.384232000
H	-2.892981000	1.577621000	-1.700205000
H	0.626451000	2.429453000	-2.173800000
H	1.263790000	1.753511000	-0.669972000
H	-0.207145000	2.744995000	-0.643791000
H	-0.266291000	-1.768864000	-2.490479000
H	0.336439000	-0.460920000	-3.518997000
H	1.262616000	-0.958904000	-2.098075000

1-(Trimethylsiloxy)cyclopentene (radical cation)

G_{298} = –677.897640 Hartree

C	1.752149000	-0.730457000	2.301569000
C	0.500400000	-0.947003000	3.177517000
C	-0.613478000	-0.154050000	2.475544000
C	-0.144054000	-0.045081000	1.065158000
C	1.230934000	-0.377017000	0.969791000
O	-0.921869000	0.328191000	0.124176000
Si	-0.605042000	0.542115000	-1.606231000
C	-2.258413000	1.113737000	-2.205373000
C	0.715438000	1.836065000	-1.742462000

C	-0.116806000	-1.130449000	-2.237992000
H	2.435841000	-1.582929000	2.262749000
H	2.353588000	0.122162000	2.657156000
H	0.654805000	-0.623069000	4.205405000
H	0.244874000	-2.008080000	3.186931000
H	-1.607515000	-0.600950000	2.530111000
H	-0.691624000	0.870460000	2.862759000
H	1.817872000	-0.341049000	0.061353000
H	-2.220217000	1.277551000	-3.287232000
H	-3.032934000	0.368944000	-2.001981000
H	-2.545852000	2.055833000	-1.730091000
H	0.796233000	2.146990000	-2.789823000
H	1.695698000	1.471177000	-1.426992000
H	0.461301000	2.720152000	-1.150484000
H	-0.861541000	-1.884572000	-1.967688000
H	-0.057448000	-1.094047000	-3.331124000
H	0.857946000	-1.449219000	-1.860537000

Dihydro-β-ionone (neutral)

G_{298} = –582.716645 Hartree

C	-1.065692000	0.872050000	-2.525683000
C	-2.468963000	0.517226000	-2.055417000
C	-2.552063000	0.687790000	-0.547008000
C	-1.586612000	-0.240264000	0.204652000
C	-0.199606000	-0.232593000	-0.443919000
C	0.022797000	0.271610000	-1.664503000
C	1.379587000	0.315186000	-2.321910000
C	-1.519754000	0.250875000	1.658977000
C	-2.138974000	-1.676168000	0.204077000
C	0.923550000	-0.854792000	0.368420000

C	1.792133000	0.180692000	1.078325000
C	2.904390000	-0.409388000	1.913416000
C	3.698477000	0.570146000	2.740347000
O	3.143913000	-1.601789000	1.932260000
H	-0.940302000	1.963424000	-2.539139000
H	-0.913270000	0.542302000	-3.559701000
H	-3.207974000	1.151305000	-2.554778000
H	-2.701046000	-0.516675000	-2.330499000
H	-2.306457000	1.727530000	-0.297319000
H	-3.569628000	0.504842000	-0.185291000
H	2.177083000	-0.119885000	-1.721879000
H	1.350699000	-0.210222000	-3.283037000
H	1.654797000	1.353248000	-2.543166000
H	-1.108033000	1.262461000	1.715905000
H	-2.530253000	0.277418000	2.079847000
H	-0.916989000	-0.402873000	2.293740000
H	-1.471194000	-2.360802000	0.733985000
H	-3.112258000	-1.707143000	0.704765000
H	-2.266937000	-2.056381000	-0.812420000
H	0.510456000	-1.534810000	1.115235000
H	1.559279000	-1.469459000	-0.271171000
H	2.263696000	0.858377000	0.357444000
H	1.196858000	0.825078000	1.732836000
H	3.074817000	0.905893000	3.575524000
H	4.600504000	0.099670000	3.132253000
H	3.955647000	1.454045000	2.151237000

Dihydro-β-ionone (radical cation)

G_{298} = –582.499623 Hartree

C	-1.009250000	0.731115000	-2.541443000

C	-2.437721000	0.533044000	-2.059067000
C	-2.506107000	0.768183000	-0.559852000
C	-1.595297000	-0.182700000	0.230042000
C	-0.212292000	-0.249463000	-0.343230000
C	0.045590000	0.154233000	-1.682390000
C	1.385339000	0.006763000	-2.291623000
C	-1.596726000	0.245607000	1.703469000
C	-2.130158000	-1.639504000	0.134072000
C	0.894983000	-0.818768000	0.468089000
C	1.711812000	0.297402000	1.141448000
C	2.941718000	-0.249021000	1.839015000
C	3.715806000	0.744830000	2.662935000
O	3.272957000	-1.412453000	1.741893000
H	-0.762182000	1.807153000	-2.569251000
H	-0.848078000	0.385594000	-3.567652000
H	-3.094758000	1.228851000	-2.584769000
H	-2.774816000	-0.476205000	-2.307529000
H	-2.209517000	1.799876000	-0.340475000
H	-3.527074000	0.639180000	-0.192193000
H	1.444259000	-1.020973000	-2.680499000
H	1.498029000	0.681173000	-3.141016000
H	2.208532000	0.135392000	-1.591631000
H	-1.206888000	1.259546000	1.817344000
H	-2.630279000	0.238494000	2.058515000
H	-1.021570000	-0.428804000	2.339470000
H	-1.561786000	-2.307439000	0.783393000
H	-3.172243000	-1.633890000	0.463381000
H	-2.089305000	-2.030875000	-0.883888000
H	0.497716000	-1.477463000	1.239029000

H	1.560790000	-1.406484000	-0.164985000
H	2.055291000	1.044436000	0.419237000
H	1.115279000	0.840902000	1.877465000
H	3.121088000	1.009802000	3.542902000
H	4.666273000	0.317116000	2.981579000
H	3.883260000	1.663277000	2.094622000

Dihydropyran (neutral)

G_{298} = –270.073404 Hartree

C	-0.200093000	0.552062000	1.025853000
C	-0.197113000	-0.966427000	1.028745000
O	-0.832477000	-1.503408000	-0.144141000
C	-0.463111000	-0.892276000	-1.304868000
C	0.210219000	0.253086000	-1.396234000
C	0.583668000	1.062982000	-0.183220000
H	0.231437000	0.912037000	1.963249000
H	-1.236173000	0.902841000	0.978659000
H	-0.750575000	-1.375513000	1.874704000
H	0.831781000	-1.345442000	1.063225000
H	-0.793341000	-1.455353000	-2.172506000
H	0.455837000	0.618095000	-2.387879000
H	1.661204000	0.995902000	0.006811000
H	0.365814000	2.120454000	-0.361400000

Dihydropyran (radical cation)

G_{298} = –269.858715 Hartree

C	-0.208688000	0.554124000	1.030578000
C	-0.176446000	-0.953630000	1.067947000
O	-0.714618000	-1.526671000	-0.188674000
C	-0.471560000	-0.927273000	-1.299316000
C	0.206898000	0.296675000	-1.390076000

C	0.559015000	1.062842000	-0.186094000
H	0.237646000	0.922249000	1.955601000
H	-1.245049000	0.899579000	0.994349000
H	-0.811724000	-1.380784000	1.839590000
H	0.835997000	-1.353234000	1.151539000
H	-0.844087000	-1.445745000	-2.178208000
H	0.455183000	0.654145000	-2.382892000
H	1.647243000	0.948788000	-0.049292000
H	0.397267000	2.127975000	-0.374052000

1-Pentyl-1-cyclopentene (neutral)

G_{298} = –391.061386 Hartree

C	-1.012068000	-1.560907000	-1.160810000
C	-1.844505000	-2.703782000	-0.548548000
C	-1.296249000	-2.877634000	0.884088000
C	0.084279000	-2.274069000	0.786325000
C	0.251608000	-1.561680000	-0.326472000
C	1.470987000	-0.791899000	-0.737516000
C	1.198211000	0.701111000	-0.963459000
C	0.738468000	1.431549000	0.295229000
C	0.509241000	2.923972000	0.064793000
C	0.086883000	3.661900000	1.332496000
H	-1.519394000	-0.592974000	-1.062566000
H	-0.810261000	-1.706323000	-2.226722000
H	-1.664688000	-3.622560000	-1.114243000
H	-2.918443000	-2.506836000	-0.571902000
H	-1.902097000	-2.332382000	1.619178000
H	-1.284270000	-3.924809000	1.200952000
H	0.834878000	-2.378426000	1.564864000
H	2.251346000	-0.912149000	0.021488000

H	1.859940000	-1.220232000	-1.669631000
H	2.115197000	1.170672000	-1.336840000
H	0.445644000	0.820349000	-1.751546000
H	-0.187876000	0.978723000	0.668599000
H	1.489435000	1.296780000	1.084534000
H	1.427865000	3.373718000	-0.330663000
H	-0.257632000	3.054366000	-0.708171000
H	0.854555000	3.579714000	2.108960000
H	-0.078970000	4.726029000	1.139473000
H	-0.842082000	3.247777000	1.738107000

1-Pentyl-1-cyclopentene (radical cation)

G_{298} = –390.836736 Hartree

C	-1.085506000	-1.384646000	-0.908124000
C	-1.894160000	-2.651036000	-0.582384000
C	-1.223058000	-3.217461000	0.680575000
C	0.117071000	-2.621956000	0.694885000
C	0.216227000	-1.562469000	-0.230240000
C	1.437037000	-0.789069000	-0.494960000
C	1.216265000	0.679897000	-0.866265000
C	0.751701000	1.522621000	0.320041000
C	0.513746000	2.982309000	-0.058988000
C	0.084519000	3.837548000	1.130249000
H	-1.542727000	-0.486869000	-0.461521000
H	-0.968351000	-1.161415000	-1.971646000
H	-1.795424000	-3.368372000	-1.399468000
H	-2.952994000	-2.443478000	-0.436492000
H	-1.727958000	-2.876596000	1.602038000
H	-1.195248000	-4.308756000	0.755479000
H	0.929204000	-2.927098000	1.345839000

H	2.138252000	-0.902495000	0.335783000
H	1.892882000	-1.313403000	-1.353516000
H	2.162052000	1.075348000	-1.247093000
H	0.493471000	0.750225000	-1.684777000
H	-0.171816000	1.102030000	0.736370000
H	1.505422000	1.469377000	1.114915000
H	1.430819000	3.394844000	-0.495718000
H	-0.252425000	3.029243000	-0.841780000
H	0.850669000	3.835705000	1.912323000
H	-0.086267000	4.876274000	0.832631000
H	-0.843399000	3.459697000	1.571839000

1,1-Diphenylethylene (neutral)

G_{298} = –539.661239 Hartree

C	-2.709389000	1.992327000	-1.410976000
C	-1.730499000	2.848743000	-1.901905000
C	-0.392198000	2.478914000	-1.822323000
C	-0.036675000	1.261713000	-1.256433000
C	-1.013115000	0.388479000	-0.772830000
C	-2.352640000	0.772519000	-0.849374000
C	-0.629829000	-0.936823000	-0.210508000
C	0.593242000	-1.009093000	0.636713000
C	-1.357845000	-2.026662000	-0.460617000
C	1.478960000	-2.081337000	0.521414000
C	2.601314000	-2.165061000	1.336663000
C	2.855834000	-1.174649000	2.278311000
C	1.982282000	-0.098551000	2.396652000
C	0.864004000	-0.013731000	1.577744000
H	-3.755293000	2.277529000	-1.458160000
H	-2.008223000	3.801787000	-2.339913000

H	0.378756000	3.140651000	-2.203363000
H	1.009713000	0.982136000	-1.199042000
H	-3.120216000	0.115841000	-0.454105000
H	-1.102315000	-2.987885000	-0.027295000
H	-2.229113000	-1.987055000	-1.105788000
H	1.294121000	-2.848466000	-0.222938000
H	3.282834000	-3.002585000	1.229323000
H	3.733726000	-1.237287000	2.912710000
H	2.173246000	0.678769000	3.129219000
H	0.189320000	0.829775000	1.676823000

1,1-Diphenylethylene (radical cation)

G_{298} = –539.433437 Hartree

C	-2.735027000	1.918038000	-1.446924000
C	-1.763839000	2.847848000	-1.820866000
C	-0.411277000	2.555257000	-1.671961000
C	-0.024137000	1.341091000	-1.135335000
C	-0.993486000	0.392631000	-0.758282000
C	-2.357231000	0.692776000	-0.937076000
C	-0.577526000	-0.891542000	-0.221645000
C	0.598060000	-0.998178000	0.612524000
C	-1.322506000	-2.028879000	-0.517375000
C	1.371034000	-2.179711000	0.634798000
C	2.495732000	-2.255618000	1.427363000
C	2.853463000	-1.175056000	2.236904000
C	2.086891000	-0.011676000	2.247701000
C	0.973095000	0.083969000	1.437728000
H	-3.786464000	2.155901000	-1.559965000
H	-2.066152000	3.802006000	-2.238946000
H	0.339030000	3.272661000	-1.983605000

H	1.027733000	1.097481000	-1.048196000
H	-3.114019000	-0.021116000	-0.634633000
H	-1.017274000	-3.008045000	-0.170090000
H	-2.230922000	-1.962759000	-1.103712000
H	1.108222000	-3.011990000	-0.006465000
H	3.102364000	-3.153602000	1.422255000
H	3.731392000	-1.246699000	2.869826000
H	2.358376000	0.813161000	2.896096000
H	0.354468000	0.972050000	1.469881000

α-Methylstyrene (neutral)

G_{298} = –348.278403 Hartree

C	-1.696784000	1.236584000	-1.304467000
C	-0.803543000	2.299389000	-1.394764000
C	0.441223000	2.196284000	-0.786639000
C	0.788840000	1.043063000	-0.091796000
C	-0.104306000	-0.025324000	0.018871000
C	-1.348757000	0.086904000	-0.608990000
C	0.267681000	-1.254181000	0.777514000
C	-0.655351000	-2.013868000	1.368479000
C	1.732200000	-1.598235000	0.867022000
H	-2.667154000	1.299060000	-1.786242000
H	-1.073921000	3.196654000	-1.941588000
H	1.149122000	3.016135000	-0.853075000
H	1.765167000	0.984262000	0.375051000
H	-2.047874000	-0.740891000	-0.568423000
H	-0.372917000	-2.913768000	1.906322000
H	-1.710598000	-1.762418000	1.348089000
H	2.276020000	-0.858745000	1.462153000
H	1.867869000	-2.573621000	1.337450000

H	2.193082000	-1.617283000	-0.124969000

α-Methylstyrene (radical cation)

G_{298} = –348.049030 Hartree

C	-1.785124000	1.350876000	-1.049855000
C	-0.796282000	2.284845000	-1.370882000
C	0.542015000	2.068770000	-1.009229000
C	0.889585000	0.929817000	-0.331776000
C	-0.094183000	-0.044914000	0.003072000
C	-1.447895000	0.202409000	-0.377990000
C	0.289728000	-1.226389000	0.706867000
C	-0.631330000	-2.229101000	0.965661000
C	1.703291000	-1.421102000	1.167119000
H	-2.815369000	1.534160000	-1.329912000
H	-1.066302000	3.189372000	-1.905323000
H	1.295361000	2.803649000	-1.266252000
H	1.922558000	0.765385000	-0.056082000
H	-2.226495000	-0.505890000	-0.132983000
H	-0.312581000	-3.119003000	1.496657000
H	-1.665224000	-2.182386000	0.653138000
H	2.018290000	-0.594069000	1.807848000
H	1.798177000	-2.354481000	1.719806000
H	2.381780000	-1.451946000	0.310115000

Cinnamylchloride (neutral)

G_{298} = –807.454076 Hartree

C	-1.421845000	1.920901000	-1.027855000
C	-0.396242000	2.760879000	-1.454696000
C	0.924459000	2.412129000	-1.202723000
C	1.215521000	1.229725000	-0.532607000
C	0.196043000	0.375268000	-0.107737000

C	-1.130319000	0.741461000	-0.359551000
C	0.563794000	-0.874085000	0.586602000
C	-0.263154000	-1.862895000	0.920776000
C	0.202844000	-3.069248000	1.655400000
Cl	-0.019658000	-4.558954000	0.632632000
H	-2.456373000	2.190491000	-1.213796000
H	-0.628491000	3.683690000	-1.975851000
H	1.731390000	3.061039000	-1.526897000
H	2.248767000	0.959585000	-0.336428000
H	-1.944279000	0.107223000	-0.026736000
H	1.619095000	-0.973740000	0.833918000
H	-1.321645000	-1.826557000	0.679667000
H	1.261567000	-3.021441000	1.900710000
H	-0.381477000	-3.255470000	2.555172000

Cinnamylchloride (radical cation)

G_{298} = –807.223460 Hartree

C	-1.436248000	1.847408000	-1.049534000
C	-0.402922000	2.726939000	-1.413034000
C	0.941209000	2.428171000	-1.138718000
C	1.252358000	1.257219000	-0.503288000
C	0.221370000	0.343660000	-0.124834000
C	-1.139406000	0.671888000	-0.417170000
C	0.606568000	-0.848478000	0.526765000
C	-0.260553000	-1.830308000	0.940786000
C	0.207933000	-3.042182000	1.653206000
Cl	-0.283903000	-4.484876000	0.690452000
H	-2.464107000	2.105557000	-1.273364000
H	-0.649239000	3.656168000	-1.915544000
H	1.718785000	3.123946000	-1.429355000

H	2.280237000	0.997994000	-0.275616000
H	-1.933838000	-0.007534000	-0.138781000
H	1.667427000	-0.991143000	0.711832000
H	-1.327886000	-1.752758000	0.764315000
H	1.288226000	-3.073455000	1.769758000
H	-0.286015000	-3.128215000	2.622126000

Norbornene (neutral)

G_{298} = –272.180118 Hartree

C	-1.044058000	0.789909000	0.539788000
C	-1.058365000	-0.763839000	0.549543000
C	0.182167000	-1.128120000	-0.319100000
C	1.389086000	-0.676858000	0.484297000
C	1.401613000	0.656944000	0.475883000
C	0.202991000	1.120564000	-0.332892000
C	0.118800000	-0.009560000	-1.374983000
H	-1.949095000	1.188106000	0.073959000
H	-0.963637000	1.218718000	1.540690000
H	-0.985482000	-1.181423000	1.555743000
H	-1.970777000	-1.151134000	0.088935000
H	0.197957000	-2.156939000	-0.677587000
H	2.032826000	-1.334866000	1.057922000
H	2.057631000	1.309901000	1.041314000
H	0.237699000	2.144463000	-0.703878000
H	0.969313000	-0.021599000	-2.060664000
H	-0.818669000	-0.004267000	-1.938970000

Norbornene (radical cation)

G_{298} = –271.947417 Hartree

C	-1.024832000	0.786588000	0.555485000
C	-1.038588000	-0.760678000	0.565621000

C	0.193319000	-1.146092000	-0.343883000
C	1.327766000	-0.716210000	0.482633000
C	1.341279000	0.697110000	0.473787000
C	0.214437000	1.138192000	-0.357445000
C	0.130655000	-0.009535000	-1.387256000
H	-1.907537000	1.204020000	0.071022000
H	-0.940506000	1.217917000	1.552853000
H	-0.960879000	-1.180362000	1.568480000
H	-1.929112000	-1.168752000	0.087523000
H	0.196506000	-2.180345000	-0.673635000
H	1.989921000	-1.345574000	1.065951000
H	2.016119000	1.320885000	1.048507000
H	0.236900000	2.168164000	-0.699621000
H	0.970748000	-0.021501000	-2.081665000
H	-0.816195000	-0.003828000	-1.928357000

1,1-bis(3-(trifluoromethyl)phenyl)ethene (neutral)

G_{298} = –1213.197991 Hartree

C	-2.688205000	1.992460000	-1.446521000
C	-1.698939000	2.850440000	-1.904102000
C	-0.367976000	2.482981000	-1.749186000
C	-0.040008000	1.275116000	-1.152733000
C	-1.035278000	0.402229000	-0.704855000
C	-2.366598000	0.780348000	-0.847806000
C	-0.678371000	-0.917830000	-0.115613000
C	0.568828000	-1.001933000	0.694794000
C	-1.431836000	-1.999196000	-0.316625000
C	1.458751000	-2.055131000	0.504194000
C	2.614060000	-2.138063000	1.270184000
C	2.896680000	-1.185310000	2.240669000

C	2.011103000	-0.132805000	2.431188000
C	0.862354000	-0.036806000	1.658654000
H	-1.957138000	3.793553000	-2.369370000
H	0.417087000	3.143532000	-2.099594000
H	1.003148000	1.000909000	-1.043721000
H	-3.155003000	0.131102000	-0.483315000
H	-1.177469000	-2.951728000	0.136055000
H	-2.319486000	-1.965894000	-0.939148000
H	1.254609000	-2.796976000	-0.258245000
H	3.799761000	-1.255966000	2.835531000
H	2.221488000	0.618598000	3.183951000
H	0.181731000	0.794045000	1.809487000
C	3.551788000	-3.295556000	1.067505000
F	4.837210000	-2.965698000	1.309412000
F	3.263581000	-4.333244000	1.889472000
F	3.504787000	-3.786070000	-0.187882000
C	-4.140910000	2.342907000	-1.602940000
F	-4.331910000	3.582497000	-2.091619000
F	-4.773779000	1.492101000	-2.444799000
F	-4.810553000	2.278804000	-0.429928000

1,1-bis(3-(trifluoromethyl)phenyl)ethene (radical cation)

G_{298} = –1212.961913 Hartree

C	-2.728787000	1.968184000	-1.369910000
C	-1.783199000	2.915800000	-1.753317000
C	-0.428466000	2.624247000	-1.647255000
C	-0.020198000	1.402283000	-1.147540000
C	-0.971686000	0.438219000	-0.771015000
C	-2.337309000	0.728217000	-0.903263000
C	-0.533307000	-0.859413000	-0.278673000

C	0.642150000	-0.969412000	0.552839000
C	-1.258533000	-1.999576000	-0.614922000
C	1.426322000	-2.142141000	0.541194000
C	2.545516000	-2.216529000	1.336439000
C	2.892639000	-1.162195000	2.185833000
C	2.113999000	-0.012858000	2.222041000
C	1.004013000	0.092422000	1.406567000
H	-2.099988000	3.877664000	-2.137288000
H	0.306805000	3.355659000	-1.959976000
H	1.035710000	1.167839000	-1.086933000
H	-3.082031000	0.001772000	-0.600829000
H	-0.937704000	-2.985541000	-0.302359000
H	-2.170026000	-1.927008000	-1.196138000
H	1.176058000	-2.958895000	-0.122391000
H	3.767583000	-1.244631000	2.821216000
H	2.374879000	0.795748000	2.893643000
H	0.379828000	0.975490000	1.457850000
C	3.399786000	-3.457280000	1.330674000
F	4.714809000	-3.162015000	1.309301000
F	3.190726000	-4.206556000	2.435467000
F	3.152677000	-4.244707000	0.269701000
C	-4.201983000	2.267520000	-1.479212000
F	-4.447692000	3.574467000	-1.676608000
F	-4.776713000	1.602401000	-2.506319000
F	-4.872368000	1.904242000	-0.365726000

2,3-Dimethyl-2-butene (neutral)

G_{298} = –235.349548 Hartree

C	-1.632551000	-0.003147000	-2.119913000
C	-1.780098000	-0.003393000	-0.788620000

C	-1.515240000	1.250700000	-2.954256000
H	-0.693157000	1.141548000	-3.670035000
H	-2.424956000	1.406264000	-3.546186000
H	-1.329718000	2.152480000	-2.373213000
C	-1.571985000	-1.251511000	-2.967397000
H	-0.573120000	-1.372538000	-3.402582000
H	-1.821444000	-2.164885000	-2.430323000
H	-2.268032000	-1.158573000	-3.808700000
C	-1.887615000	1.250439000	0.047204000
H	-2.705459000	1.144191000	0.768172000
H	-0.973459000	1.402824000	0.633125000
H	-2.073497000	2.153093000	-0.532414000
C	-1.847255000	-1.252174000	0.057584000
H	-2.842859000	-1.361541000	0.503071000
H	-1.614377000	-2.167753000	-0.483130000
H	-1.141286000	-1.168862000	0.891709000

2,3-Dimethyl-2-butene (radical cation)

G_{298} = –235.134243 Hartree

C	-1.752534000	0.018291000	-2.162893000
C	-1.656745000	0.018292000	-0.745430000
C	-1.903438000	1.274775000	-2.923826000
H	-0.896800000	1.582646000	-3.247488000
H	-2.482249000	1.096278000	-3.831516000
H	-2.336505000	2.087947000	-2.344173000
C	-1.678521000	-1.258100000	-2.906545000
H	-0.769236000	-1.805612000	-2.636092000
H	-2.519187000	-1.898867000	-2.611233000
H	-1.710153000	-1.097360000	-3.982313000
C	-1.505837000	1.274775000	0.015503000

H	-2.512473000	1.582641000	0.339176000
H	-0.927017000	1.096278000	0.923187000
H	-1.072778000	2.087950000	-0.564152000
C	-1.730760000	-1.258098000	-0.001777000
H	-2.640037000	-1.805617000	-0.272238000
H	-0.890085000	-1.898860000	-0.297079000
H	-1.699138000	-1.097357000	1.073991000

2-Methyl-2-pentene (neutral)

G_{298} = –235.348158 Hartree

C	-0.224196000	0.044881000	-2.125144000
C	-0.498211000	0.096308000	-0.819994000
H	-0.756225000	-0.842261000	-0.327707000
C	0.166722000	1.226963000	-2.972073000
H	1.106615000	1.018891000	-3.494624000
H	-0.589275000	1.404409000	-3.745452000
H	0.293164000	2.146711000	-2.401681000
C	-0.290459000	-1.258719000	-2.878616000
H	0.679946000	-1.493495000	-3.330264000
H	-0.579845000	-2.087275000	-2.228391000
H	-1.013067000	-1.194609000	-3.700165000
C	-0.471894000	1.293970000	0.089134000
H	-1.444402000	1.379280000	0.588895000
H	-0.331101000	2.217264000	-0.476248000
C	0.620322000	1.172079000	1.155768000
H	0.484302000	0.265293000	1.753480000
H	1.611131000	1.122927000	0.693787000
H	0.604369000	2.028789000	1.836152000

2-Methyl-2-pentene (radical cation)

G_{298} = –235.122644 Hartree

C	-0.142646000	0.002481000	-2.231192000
C	-0.195837000	0.085500000	-0.819862000
H	-0.297693000	-0.852671000	-0.278625000
C	-0.039560000	1.220811000	-3.055548000
H	-0.030661000	0.991126000	-4.119296000
H	-0.874448000	1.892938000	-2.822514000
H	0.871930000	1.767507000	-2.784631000
C	-0.191054000	-1.312819000	-2.887639000
H	0.734490000	-1.440849000	-3.465626000
H	-0.316265000	-2.137870000	-2.188877000
H	-0.997151000	-1.306223000	-3.631823000
C	-0.175628000	1.328786000	-0.045710000
H	-1.242214000	1.620642000	0.013090000
H	0.313986000	2.135127000	-0.595960000
C	0.382329000	1.164490000	1.366868000
H	-0.162409000	0.392044000	1.915104000
H	1.438236000	0.886257000	1.334146000
H	0.292494000	2.104130000	1.914952000

Styrene (neutral)

G_{298} = –309.050447 Hartree

C	-1.395816000	1.080043000	-0.855568000
C	-0.441509000	2.068390000	-1.084139000
C	0.867194000	1.866625000	-0.665376000
C	1.216636000	0.684407000	-0.022470000
C	0.269246000	-0.313902000	0.211144000
C	-1.045209000	-0.099320000	-0.215744000
C	0.698090000	-1.549820000	0.901156000
C	-0.067232000	-2.593673000	1.210460000
H	-2.420742000	1.231049000	-1.178530000

H	-0.719844000	2.989323000	-1.585811000
H	1.618821000	2.630194000	-0.837137000
H	2.240342000	0.529180000	0.304907000
H	-1.802501000	-0.857292000	-0.048849000
H	1.751788000	-1.575292000	1.171699000
H	0.356139000	-3.451846000	1.722262000
H	-1.125404000	-2.638064000	0.971998000

Styrene (radical cation)

G_{298} = –308.819770 Hartree

C	-1.410609000	1.042795000	-0.842342000
C	-0.438539000	2.032609000	-1.064810000
C	0.892836000	1.856224000	-0.652898000
C	1.251891000	0.697431000	-0.021308000
C	0.283695000	-0.325770000	0.217913000
C	-1.065405000	-0.121333000	-0.213656000
C	0.713093000	-1.499633000	0.880066000
C	-0.093905000	-2.563016000	1.191387000
H	-2.429854000	1.207676000	-1.169749000
H	-0.722684000	2.952184000	-1.565212000
H	1.622507000	2.635331000	-0.836748000
H	2.271048000	0.531231000	0.309190000
H	-1.810319000	-0.887016000	-0.042508000
H	1.761235000	-1.546938000	1.158990000
H	0.324174000	-3.421933000	1.704289000
H	-1.149161000	-2.589840000	0.947397000

Cyclohexene (neutral)

G_{298} = –234.160814 Hartree

C	-0.063030000	0.763727000	1.082941000
C	-0.003606000	-0.762590000	1.085879000

C	-0.682242000	-1.328462000	-0.162065000
C	-0.270817000	-0.587486000	-1.407708000
C	0.356794000	0.586162000	-1.389168000
C	0.691667000	1.328161000	-0.121401000
H	0.352269000	1.169000000	2.010280000
H	-1.111935000	1.080160000	1.034344000
H	1.046068000	-1.079577000	1.103350000
H	-0.476122000	-1.166526000	1.986040000
H	-0.445063000	-2.391462000	-0.277614000
H	-1.773442000	-1.273710000	-0.054355000
H	-0.503212000	-1.053896000	-2.362974000
H	0.645968000	1.052152000	-2.328963000
H	1.774080000	1.273192000	0.053605000
H	0.462625000	2.391154000	-0.252188000

Cyclohexene (radical cation)

G_{298} = –233.925787 Hartree

C	-0.075308000	0.762887000	1.099048000
C	0.007727000	-0.761666000	1.102411000
C	-0.628672000	-1.333508000	-0.161190000
C	-0.284069000	-0.622771000	-1.389505000
C	0.368469000	0.621543000	-1.370125000
C	0.638100000	1.333238000	-0.123610000
H	0.372692000	1.175346000	2.004423000
H	-1.124347000	1.075320000	1.080719000
H	1.055710000	-1.074461000	1.150148000
H	-0.496089000	-1.172969000	1.978563000
H	-0.454424000	-2.406406000	-0.301139000
H	-1.734139000	-1.252118000	-0.121749000
H	-0.568160000	-1.048670000	-2.347401000

H	0.710257000	1.046406000	-2.309357000
H	1.739088000	1.251768000	-0.016271000
H	0.473166000	2.406061000	-0.274964000

2-Methyl-1-pentene (neutral)

G_{298} = –235.345894 Hartree

C	-0.243544000	0.009591000	-1.720039000
C	-0.295546000	0.068235000	-0.390689000
H	-0.329637000	1.025543000	0.122244000
H	-0.305497000	-0.821700000	0.229511000
C	-0.197227000	-1.279172000	-2.503817000
H	-1.048564000	-1.279926000	-3.197562000
H	0.694056000	-1.251331000	-3.144612000
C	-0.200714000	-2.572476000	-1.697022000
H	-1.097014000	-2.611872000	-1.068872000
H	0.658630000	-2.584825000	-1.018171000
C	-0.230796000	1.256844000	-2.564025000
H	0.672200000	1.292608000	-3.182867000
H	-1.085109000	1.264871000	-3.249496000
H	-0.268451000	2.159574000	-1.950711000
C	-0.157079000	-3.803617000	-2.598876000
H	-0.154169000	-4.726360000	-2.011633000
H	-1.025482000	-3.833749000	-3.264977000
H	0.741443000	-3.802177000	-3.224407000

2-Methyl-1-pentene (radical cation)

G_{298} = –235.107030 Hartree

C	-0.210864000	-0.008831000	-1.799245000
C	-0.254252000	0.080320000	-0.388923000
H	-0.380367000	1.048778000	0.083540000
H	-0.160322000	-0.799870000	0.234984000

C	-0.136652000	-1.295436000	-2.503899000
H	-0.902469000	-1.254452000	-3.293610000
H	0.807159000	-1.244957000	-3.073816000
C	-0.229773000	-2.578567000	-1.689905000
H	-1.161312000	-2.578262000	-1.115664000
H	0.593874000	-2.618006000	-0.970895000
C	-0.262608000	1.237221000	-2.576977000
H	-0.035499000	1.085709000	-3.630664000
H	-1.292265000	1.618255000	-2.481746000
H	0.379170000	2.001579000	-2.128714000
C	-0.180986000	-3.805581000	-2.595488000
H	-0.245854000	-4.724780000	-2.008056000
H	-1.011984000	-3.800730000	-3.307513000
H	0.751557000	-3.834796000	-3.167189000

4-Methoxy-2-methylbut-1-ene (neutral)

G_{298} = –310.475729 Hartree

O	1.001819000	-1.276613000	-0.185064000
C	0.476432000	0.035379000	-0.330146000
C	-0.655699000	0.204289000	0.662978000
C	-1.344820000	1.545824000	0.645327000
C	-2.490663000	1.672021000	1.613498000
C	-0.989503000	2.553339000	-0.149683000
H	1.270768000	0.772683000	-0.148026000
H	0.117094000	0.177290000	-1.359142000
H	-1.406473000	-0.574044000	0.482348000
H	-0.272946000	0.024486000	1.674790000
H	-3.255980000	0.917591000	1.402345000
H	-2.148278000	1.499844000	2.639485000
H	-2.952520000	2.660107000	1.562087000

H	-1.522911000	3.499200000	-0.115181000
H	-0.167647000	2.480742000	-0.854287000
C	2.071204000	-1.508948000	-1.082266000
H	2.426233000	-2.528159000	-0.917910000
H	1.744774000	-1.408596000	-2.126324000
H	2.899665000	-0.809535000	-0.905561000

4-Methoxy-2-methylbut-1-ene (radical cation)

G_{298} = –310.233856 Hartree

O	1.061241000	-1.233544000	-0.121688000
C	0.489912000	0.040831000	-0.337347000
C	-0.574489000	0.235706000	0.725936000
C	-1.347928000	1.483877000	0.691719000
C	-2.483831000	1.648340000	1.608480000
C	-1.050664000	2.536318000	-0.204617000
H	1.260785000	0.819618000	-0.261984000
H	0.045956000	0.095817000	-1.341055000
H	-1.298410000	-0.591130000	0.721695000
H	-0.133667000	0.195397000	1.735506000
H	-3.392486000	1.568466000	0.989662000
H	-2.512852000	0.884698000	2.383554000
H	-2.500245000	2.658142000	2.029188000
H	-1.695194000	3.408425000	-0.236326000
H	-0.191710000	2.497900000	-0.863493000
C	2.079188000	-1.514356000	-1.070001000
H	2.483173000	-2.499766000	-0.833264000
H	1.676122000	-1.526027000	-2.090854000
H	2.885650000	-0.771811000	-1.015843000

3-Methylbut-3-en-1-yl acetate (neutral)

G_{298} = –423.703916 Hartree

C	2.271032000	-3.116278000	-0.925755000
C	1.878084000	-1.678308000	-1.109948000
O	0.900678000	-1.325892000	-0.262831000
O	2.365126000	-0.917057000	-1.915341000
C	0.435266000	0.038432000	-0.364732000
C	-0.697107000	0.189237000	0.627641000
C	-1.332884000	1.556597000	0.663668000
C	-2.494976000	1.675790000	1.612823000
C	-0.920969000	2.587018000	-0.072518000
H	1.402799000	-3.757525000	-1.094078000
H	3.064872000	-3.375717000	-1.623676000
H	2.610470000	-3.275461000	0.100330000
H	1.267014000	0.709342000	-0.141372000
H	0.102186000	0.220908000	-1.388128000
H	-1.474648000	-0.548608000	0.399160000
H	-0.332750000	-0.052464000	1.632877000
H	-3.286451000	0.970126000	1.339049000
H	-2.185306000	1.427410000	2.633452000
H	-2.911987000	2.684790000	1.610827000
H	-1.418395000	3.550102000	-0.001027000
H	-0.086969000	2.518789000	-0.763175000

3-Methylbut-3-en-1-yl acetate (radical cation)

G_{298} = –423.458695 Hartree

C	2.310517000	-3.104464000	-0.877270000
C	1.881297000	-1.686560000	-1.111520000
O	0.915475000	-1.317841000	-0.248173000
O	2.320028000	-0.944183000	-1.958173000
C	0.419124000	0.020976000	-0.409102000
C	-0.643640000	0.197132000	0.656533000

C	-1.348901000	1.485850000	0.694153000
C	-2.470551000	1.656068000	1.627232000
C	-0.994822000	2.576035000	-0.134369000
H	1.457513000	-3.771171000	-1.024201000
H	3.109102000	-3.366948000	-1.568450000
H	2.653425000	-3.220976000	0.153149000
H	1.239233000	0.729797000	-0.282700000
H	0.001073000	0.135006000	-1.411298000
H	-1.412756000	-0.584874000	0.591722000
H	-0.219921000	0.071423000	1.666010000
H	-3.379806000	1.780201000	1.019115000
H	-2.591382000	0.810792000	2.301534000
H	-2.358494000	2.596684000	2.177980000
H	-1.578063000	3.489598000	-0.089696000
H	-0.153368000	2.528684000	-0.815229000

3-Methylenecyclobutanecarbonitrile (neutral)

G_{298} = –287.025578 Hartree

C	-0.322115000	0.814105000	0.665494000
C	-0.381947000	-0.697538000	0.539072000
C	0.364610000	-0.676935000	-0.782843000
C	0.743262000	0.803316000	-0.472595000
C	-0.810442000	-1.664726000	1.331686000
C	0.568446000	1.765757000	-1.552112000
N	0.420655000	2.516204000	-2.411091000
H	0.002209000	1.228296000	1.619977000
H	-1.251544000	1.294453000	0.351704000
H	1.198321000	-1.368620000	-0.902447000
H	-0.306163000	-0.756216000	-1.641086000
H	1.757331000	0.893942000	-0.083674000

H	-0.699347000	-2.709045000	1.053633000
H	-1.283274000	-1.442993000	2.284282000

3-Methylenecyclobutanecarbonitrile (radical cation)

G_{298} = –286.776605 Hartree

C	-0.241919000	0.812706000	0.688110000
C	-0.334030000	-0.651464000	0.514726000
C	0.435282000	-0.664297000	-0.747442000
C	0.772329000	0.836587000	-0.495278000
C	-0.890647000	-1.669700000	1.299633000
C	0.493008000	1.763512000	-1.584194000
N	0.266394000	2.488858000	-2.445462000
H	0.092271000	1.165634000	1.666100000
H	-1.206137000	1.284055000	0.456493000
H	1.261328000	-1.377422000	-0.799532000
H	-0.228525000	-0.836640000	-1.603998000
H	1.797279000	0.976306000	-0.154728000
H	-0.809449000	-2.704595000	0.983817000
H	-1.407184000	-1.423540000	2.221756000

1-Hexene (neutral)

G_{298} = –235.341472 Hartree

C	0.355312000	-0.321645000	-1.815451000
C	0.071516000	0.117214000	-0.594170000
H	1.013447000	0.271514000	-2.450715000
H	0.483820000	1.048744000	-0.217126000
H	-0.582384000	-0.442041000	0.071231000
C	-0.172634000	-1.589324000	-2.421689000
H	0.672086000	-2.250167000	-2.654209000
H	-0.802452000	-2.112216000	-1.694049000
C	-0.961678000	-1.336494000	-3.709917000

H	-0.331989000	-0.781452000	-4.416114000
H	-1.823411000	-0.695688000	-3.487819000
C	-1.442741000	-2.627239000	-4.368126000
H	-0.576549000	-3.264215000	-4.582627000
H	-2.069548000	-3.181975000	-3.659989000
C	-2.223951000	-2.374917000	-5.654747000
H	-1.608335000	-1.846700000	-6.390189000
H	-2.557266000	-3.312106000	-6.110284000
H	-3.111309000	-1.763077000	-5.462338000

1-Hexene (radical cation)

G_{298} = –235.090086 Hartree

C	0.184633000	-0.223838000	-1.843808000
C	0.369423000	-0.038022000	-0.466931000
H	0.574448000	0.533634000	-2.521518000
H	0.729156000	0.912006000	-0.086548000
H	0.146247000	-0.840534000	0.229685000
C	-0.447351000	-1.406720000	-2.393830000
H	0.448460000	-2.074812000	-2.461365000
H	-1.094392000	-1.896308000	-1.659654000
C	-1.067853000	-1.263908000	-3.779793000
H	-0.354331000	-0.766095000	-4.444252000
H	-1.945630000	-0.613954000	-3.702044000
C	-1.472568000	-2.614654000	-4.363851000
H	-0.585396000	-3.254004000	-4.435025000
H	-2.164999000	-3.112170000	-3.675625000
C	-2.121588000	-2.477761000	-5.737678000
H	-1.439033000	-2.001959000	-6.448926000
H	-2.398549000	-3.455164000	-6.142403000
H	-3.028742000	-1.867520000	-5.684764000

5q (neutral)

G_{298} = –805.521791 Hartree

C	-1.745616000	-1.121987000	0.273481000
C	-1.651498000	-2.477950000	0.616401000
C	-0.465525000	-3.151470000	0.409576000
C	0.641835000	-2.499642000	-0.134094000
C	0.514187000	-1.150076000	-0.467907000
C	-0.663166000	-0.435473000	-0.289397000
C	1.909829000	-3.138984000	-0.366621000
C	2.938638000	-2.450715000	-0.886840000
C	2.795562000	-1.043965000	-1.225721000
O	1.574531000	-0.460927000	-0.998551000
O	3.663199000	-0.341187000	-1.692889000
O	-2.868799000	-0.389010000	0.458506000
C	-4.015893000	-1.044925000	1.000113000
C	-0.758153000	1.028360000	-0.651032000
C	-0.331664000	1.903298000	0.501002000
C	0.668231000	2.786032000	0.524431000
C	0.964737000	3.578069000	1.772209000
C	1.583676000	3.090496000	-0.631091000
H	-2.496278000	-3.000449000	1.042916000
H	-0.382641000	-4.200513000	0.673640000
H	2.015723000	-4.188942000	-0.111838000
H	3.907308000	-2.894100000	-1.076678000
H	-4.792119000	-0.282946000	1.057920000
H	-4.350074000	-1.855878000	0.347031000
H	-3.812275000	-1.433072000	2.001889000
H	-0.155059000	1.219627000	-1.536305000
H	-1.795608000	1.251648000	-0.909338000

H	-0.916762000	1.774290000	1.410675000
H	0.287946000	3.315369000	2.588370000
H	1.994234000	3.403134000	2.104009000
H	0.874503000	4.652647000	1.578057000
H	1.357220000	2.519062000	-1.529852000
H	1.531381000	4.156305000	-0.879462000
H	2.622183000	2.884240000	-0.349974000

5q (radical cation)

G_{298} = –805.300968 Hartree

C	-1.747627000	-1.137244000	0.255142000
C	-1.648825000	-2.522408000	0.609233000
C	-0.475894000	-3.163401000	0.410335000
C	0.646834000	-2.475117000	-0.138115000
C	0.516437000	-1.105968000	-0.487295000
C	-0.651595000	-0.415091000	-0.321378000
C	1.891590000	-3.121657000	-0.342413000
C	2.929010000	-2.424100000	-0.859086000
C	2.792637000	-1.010805000	-1.217964000
O	1.571249000	-0.421009000	-1.009372000
O	3.670060000	-0.333409000	-1.681772000
O	-2.830553000	-0.441658000	0.433696000
C	-4.017291000	-1.045109000	1.018911000
C	-0.779987000	1.043960000	-0.668825000
C	-0.342548000	1.900236000	0.496253000
C	0.668702000	2.769646000	0.519477000
C	0.972864000	3.550571000	1.771657000
C	1.583958000	3.072430000	-0.635441000
H	-2.500249000	-3.038255000	1.029083000
H	-0.360927000	-4.209788000	0.667418000

H	1.993963000	-4.167929000	-0.078251000
H	3.900775000	-2.867306000	-1.034846000
H	-4.745280000	-0.239369000	1.057949000
H	-4.369918000	-1.850987000	0.376063000
H	-3.787389000	-1.404300000	2.021712000
H	-0.187634000	1.245053000	-1.558673000
H	-1.821658000	1.257125000	-0.914353000
H	-0.928968000	1.771042000	1.404160000
H	0.295701000	3.288246000	2.587310000
H	2.001753000	3.364327000	2.098310000
H	0.891085000	4.626354000	1.582284000
H	1.344419000	2.521389000	-1.543507000
H	1.550376000	4.142865000	-0.864835000
H	2.618724000	2.842033000	-0.360233000

7-Methoxy-8-methyl-2H-chromen-2-one (neutral)

G_{298} = –649.824977 Hartree

C	-1.367732000	0.214954000	0.000325000
C	-1.351157000	-1.185063000	-0.000822000
C	-0.140595000	-1.848157000	-0.000856000
C	1.061427000	-1.141606000	0.000223000
C	1.009283000	0.255181000	0.001330000
C	-0.184225000	0.964730000	0.001396000
C	2.355597000	-1.771291000	0.000305000
C	3.478534000	-1.035283000	0.001410000
C	3.413184000	0.417103000	0.002543000
O	2.167457000	0.991101000	0.002437000
O	4.363230000	1.166575000	0.003585000
O	-2.505990000	0.946932000	0.000480000
C	-0.240768000	2.466762000	0.002614000

C	-3.752736000	0.249903000	-0.000202000
H	-2.272548000	-1.750577000	-0.001668000
H	-0.114803000	-2.932795000	-0.001727000
H	2.403637000	-2.855886000	-0.000536000
H	4.467855000	-1.473926000	0.001518000
H	0.757661000	2.900128000	0.003100000
H	-0.777452000	2.831831000	-0.876913000
H	-0.777660000	2.830393000	0.882616000
H	-4.522138000	1.020943000	0.000266000
H	-3.857281000	-0.368236000	-0.896009000
H	-3.857512000	-0.369561000	0.894663000

7-Methoxy-8-methyl-2H-chromen-2-one (radical cation)

G_{298} = –649.604682 Hartree

C	-1.360959000	0.200064000	0.000403000
C	-1.345823000	-1.231840000	-0.000769000
C	-0.152497000	-1.866587000	-0.000880000
C	1.067811000	-1.127059000	0.000157000
C	1.021378000	0.291866000	0.001452000
C	-0.158117000	0.983531000	0.001486000
C	2.329950000	-1.773387000	0.000078000
C	3.461734000	-1.032783000	0.001127000
C	3.412290000	0.431053000	0.002036000
O	2.173711000	1.019897000	0.002588000
O	4.374015000	1.151423000	0.004741000
O	-2.456235000	0.898105000	0.000622000
C	-0.249509000	2.475948000	0.002498000
C	-3.757641000	0.248415000	-0.000147000
H	-2.272848000	-1.786965000	-0.001506000
H	-0.096196000	-2.948719000	-0.001721000

H	2.368392000	-2.856666000	-0.000702000
H	4.449187000	-1.476181000	0.001318000
H	0.738993000	2.928954000	0.003010000
H	-0.796005000	2.820239000	-0.879006000
H	-0.796391000	2.819077000	0.884214000
H	-4.473515000	1.065937000	0.000213000
H	-3.863004000	-0.354491000	-0.901360000
H	-3.863452000	-0.355676000	0.900223000

Toluene (neutral)

G_{298} = –271.033671 Hartree

C	-1.233249000	0.006892000	-2.643894000
C	-2.386898000	-0.325823000	-1.941920000
C	-0.077232000	0.330113000	-1.942375000
H	-3.294341000	-0.584261000	-2.478578000
H	0.830283000	0.588132000	-2.479102000
C	-2.381737000	-0.333320000	-0.551885000
C	-0.077171000	0.320168000	-0.551794000
H	-3.286453000	-0.597884000	-0.011970000
H	0.831723000	0.570016000	-0.011979000
C	-1.228313000	-0.009584000	0.163065000
H	-1.234445000	0.010439000	-3.728924000
C	-1.234457000	0.008063000	1.670217000
H	-1.917064000	-0.745026000	2.070794000
H	-0.235809000	-0.179756000	2.070838000
H	-1.563297000	0.983421000	2.043221000

Toluene (radical cation)

G_{298} = –270.794475 Hartree

C	-1.223085000	-0.001262000	-2.609744000
C	-2.416127000	-0.348503000	-1.919161000

C	-0.044823000	0.347273000	-1.910499000
H	-3.299379000	-0.610888000	-2.488791000
H	0.848672000	0.607946000	-2.464206000
C	-2.431712000	-0.347453000	-0.558623000
C	-0.056247000	0.349618000	-0.550359000
H	-3.325265000	-0.607771000	-0.003765000
H	0.826631000	0.611738000	0.021657000
C	-1.253380000	0.001479000	0.166078000
H	-1.219871000	-0.004770000	-3.694069000
C	-1.237159000	0.013308000	1.638520000
H	-2.194206000	-0.273857000	2.069970000
H	-0.443272000	-0.656161000	1.993813000
H	-0.951084000	1.015032000	1.984831000

4-Chloroanisole (neutral)

G_{298} = –805.337367 Hartree

C	-3.013139000	-2.179476000	0.040574000
C	-2.729738000	-3.529404000	-0.027766000
C	-1.417932000	-3.982621000	-0.088837000
C	-0.384369000	-3.064398000	-0.081306000
C	-0.654600000	-1.694717000	-0.012820000
C	-1.974014000	-1.252950000	0.048400000
Cl	-4.041355000	-4.688000000	-0.037931000
O	0.428621000	-0.875987000	-0.011383000
C	0.189216000	0.529012000	0.048918000
H	-4.040072000	-1.836805000	0.088313000
H	-1.204777000	-5.043802000	-0.141934000
H	0.646635000	-3.397034000	-0.128954000
H	-2.213341000	-0.199374000	0.102066000
H	-0.390411000	0.868089000	-0.815006000

H	-0.330303000	0.801583000	0.972532000
H	1.171001000	1.000803000	0.033346000

4-Chloroanisole (radical cation)

G_{298} = –805.113259 Hartree

C	-3.028492000	-2.152129000	0.041708000
C	-2.715707000	-3.530095000	-0.026127000
C	-1.384713000	-3.995672000	-0.089665000
C	-0.374425000	-3.087899000	-0.084961000
C	-0.674340000	-1.688258000	-0.015586000
C	-2.026159000	-1.232846000	0.047011000
Cl	-3.989048000	-4.657773000	-0.032319000
O	0.358986000	-0.902560000	-0.013803000
C	0.191326000	0.542199000	0.051301000
H	-4.064538000	-1.840992000	0.089305000
H	-1.183258000	-5.058133000	-0.141022000
H	0.664893000	-3.389902000	-0.132301000
H	-2.252134000	-0.176676000	0.097915000
H	-0.370269000	0.880537000	-0.819282000
H	-0.313183000	0.806147000	0.980645000
H	1.202484000	0.938970000	0.035397000

Anisole (neutral)

G_{298} = –346.165030 Hartree

C	-0.113990000	-0.018841000	-2.237610000
C	-1.248929000	-0.374048000	-1.509237000
C	1.026534000	0.377740000	-1.556083000
H	-2.150733000	-0.687344000	-2.025464000
H	1.918669000	0.658129000	-2.107074000
C	-1.239114000	-0.332190000	-0.125694000
C	1.053365000	0.425969000	-0.163110000

H	-2.117201000	-0.607815000	0.448765000
H	1.957080000	0.740234000	0.342351000
C	-0.085354000	0.067570000	0.554806000
H	-0.123564000	-0.052534000	-3.321457000
O	-0.168846000	0.074595000	1.912568000
C	0.993354000	0.467539000	2.638752000
H	1.833205000	-0.203554000	2.434846000
H	1.278595000	1.497170000	2.402172000
H	0.725106000	0.400416000	3.692669000

Anisole (radical cation)

G_{298} = –345.942679 Hartree

C	-0.125333000	-0.017988000	-2.212947000
C	-1.279897000	-0.380386000	-1.491841000
C	1.056732000	0.384972000	-1.547690000
H	-2.172946000	-0.686829000	-2.022349000
H	1.927433000	0.657670000	-2.131680000
C	-1.262203000	-0.343391000	-0.130345000
C	1.105325000	0.434252000	-0.186933000
H	-2.121531000	-0.613281000	0.471912000
H	2.002754000	0.743797000	0.330776000
C	-0.064693000	0.066946000	0.546504000
H	-0.139319000	-0.047490000	-3.296309000
O	-0.142095000	0.072974000	1.842588000
C	1.000595000	0.474063000	2.651484000
H	1.830418000	-0.207081000	2.465362000
H	1.267105000	1.503702000	2.414229000
H	0.655832000	0.391103000	3.678437000

Thioanisole (neutral)

G_{298} = –668.791574 Hartree

C	-1.248185000	-0.001243000	-2.754387000
C	-2.359500000	-0.353082000	-2.001421000
C	-0.072346000	0.363342000	-2.103276000
H	-3.282431000	-0.639680000	-2.495230000
H	0.805387000	0.641776000	-2.677400000
C	-2.308957000	-0.344957000	-0.610008000
C	-0.012472000	0.374632000	-0.718691000
H	-3.190437000	-0.624595000	-0.047513000
H	0.908297000	0.660948000	-0.219555000
C	-1.132572000	0.019567000	0.038098000
H	-1.294431000	-0.009921000	-3.837977000
S	-0.948166000	0.068510000	1.795777000
C	-2.562968000	-0.444084000	2.403865000
H	-3.345264000	0.247972000	2.090743000
H	-2.804282000	-1.459720000	2.088881000
H	-2.487918000	-0.420999000	3.492540000

Thioanisole (radical cation)

G_{298} = –668.576141 Hartree

C	-1.238566000	-0.001139000	-2.727906000
C	-2.382538000	-0.358431000	-1.997774000
C	-0.053701000	0.366708000	-2.081166000
H	-3.288759000	-0.639831000	-2.520609000
H	0.819780000	0.641165000	-2.659900000
C	-2.357452000	-0.354438000	-0.626722000
C	-0.003627000	0.379273000	-0.710039000
H	-3.236614000	-0.629950000	-0.060192000
H	0.904396000	0.661817000	-0.188285000
C	-1.160630000	0.015428000	0.033923000
H	-1.275321000	-0.010618000	-3.811489000

S	-0.979550000	0.063546000	1.712923000
C	-2.550062000	-0.442499000	2.404577000
H	-3.332109000	0.253714000	2.100382000
H	-2.784235000	-1.460395000	2.090524000
H	-2.417256000	-0.405882000	3.486198000

SPECTROSCOPIC DATA

^{1}H NMR of acridine 2

$CDCl_3$, 500 MHz, 25 ºC

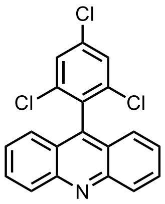

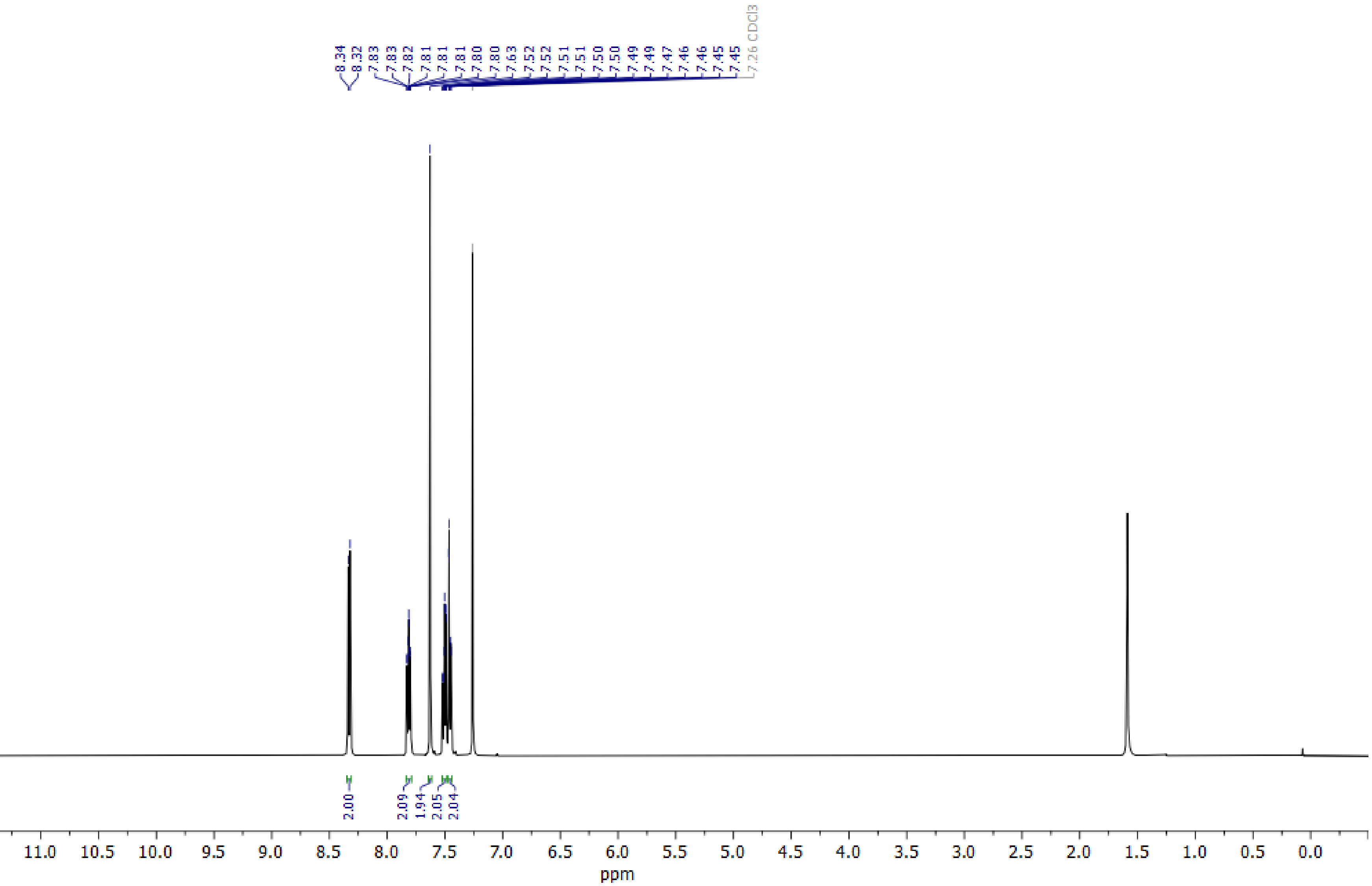

^{13}C NMR of acridine 2

$CDCl_3$, 125 MHz, 25 ºC

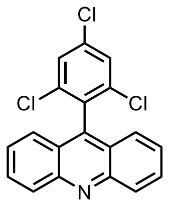

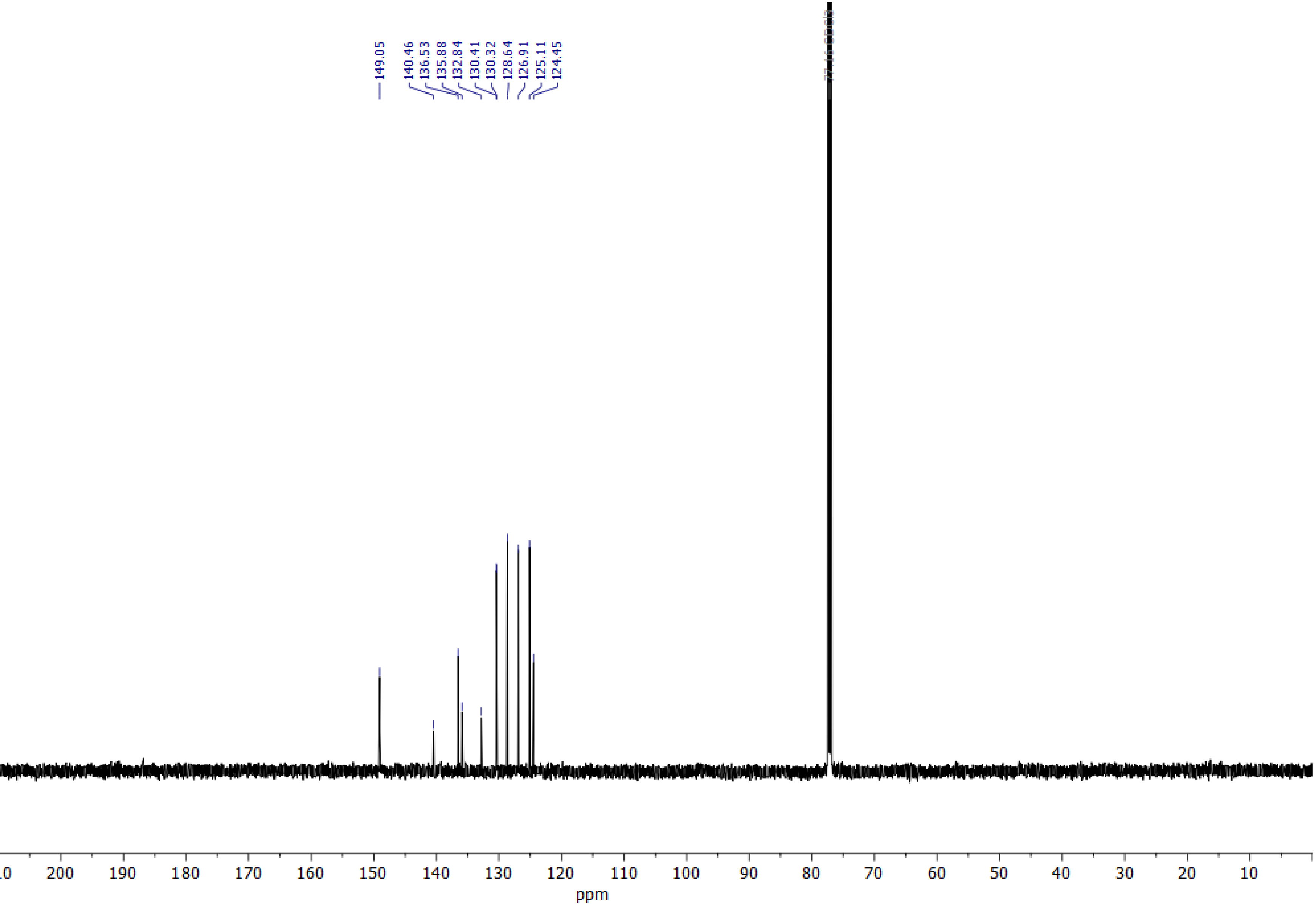

^{1}H NMR of acridinium $3^+BF_4^-$

$CDCl_3$, 600 MHz, 25 ºC

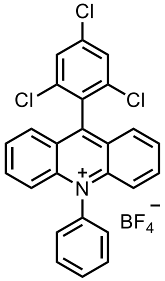

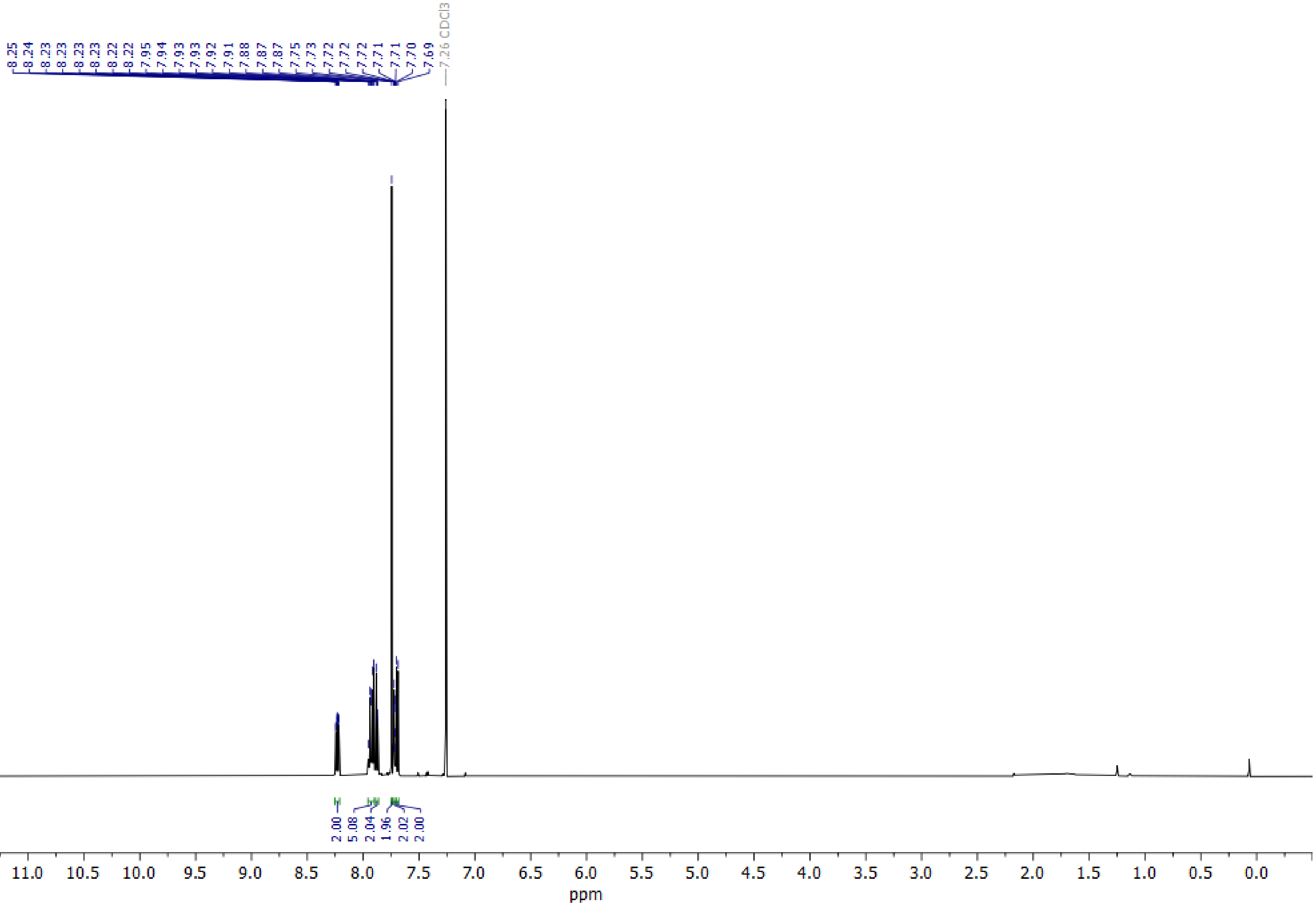

^{13}C NMR of acridinium $3^{+}BF_4^{-}$

$CDCl_3$, 125 MHz, 25 ºC

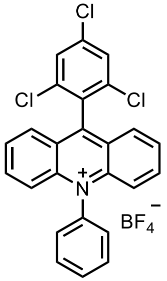

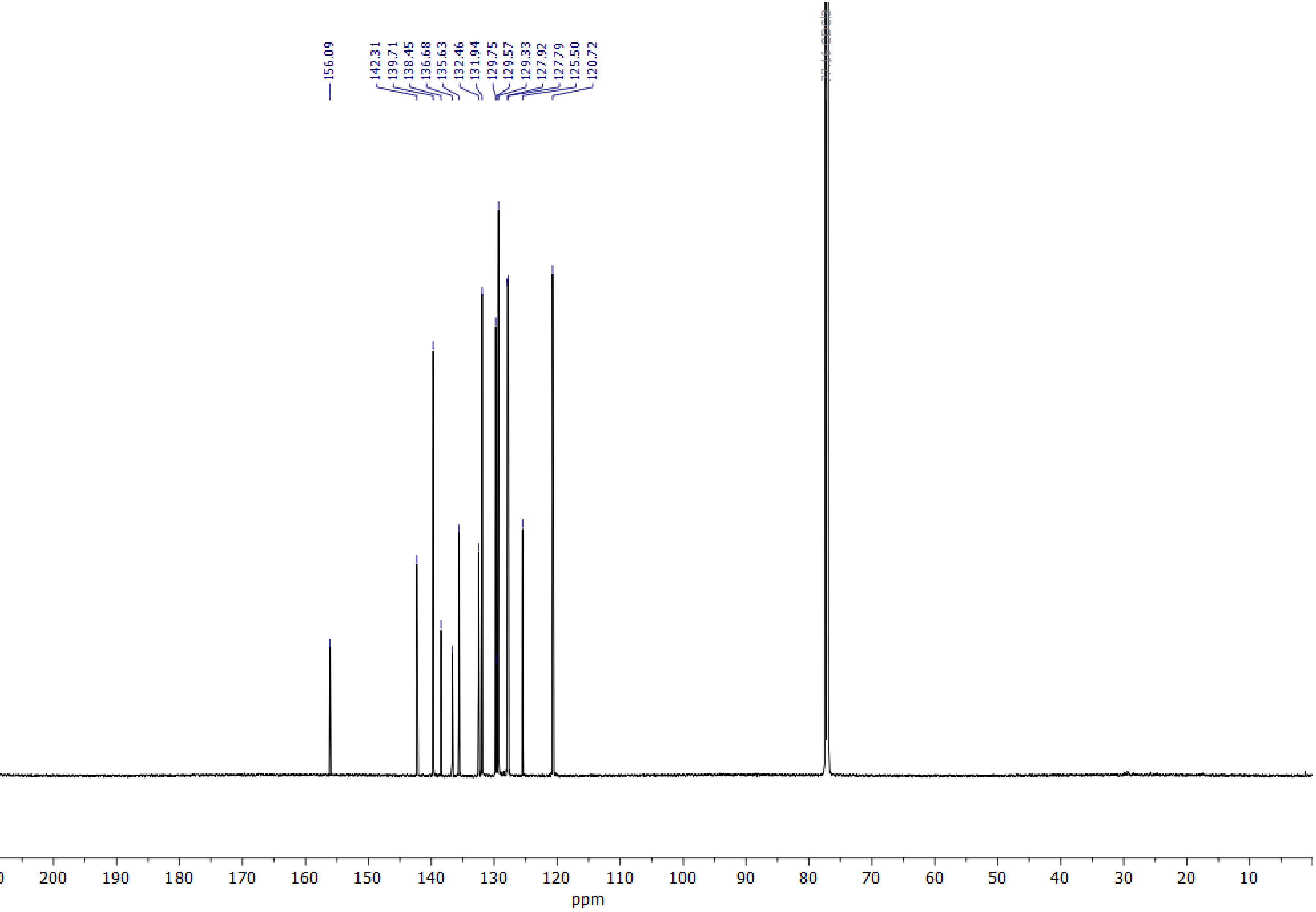

^{19}F NMR of acridinium $3^{+}BF_4^{-}$

$CDCl_3$, 470 MHz, 25 ºC

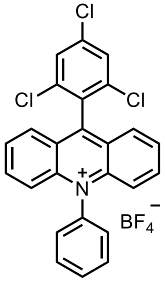

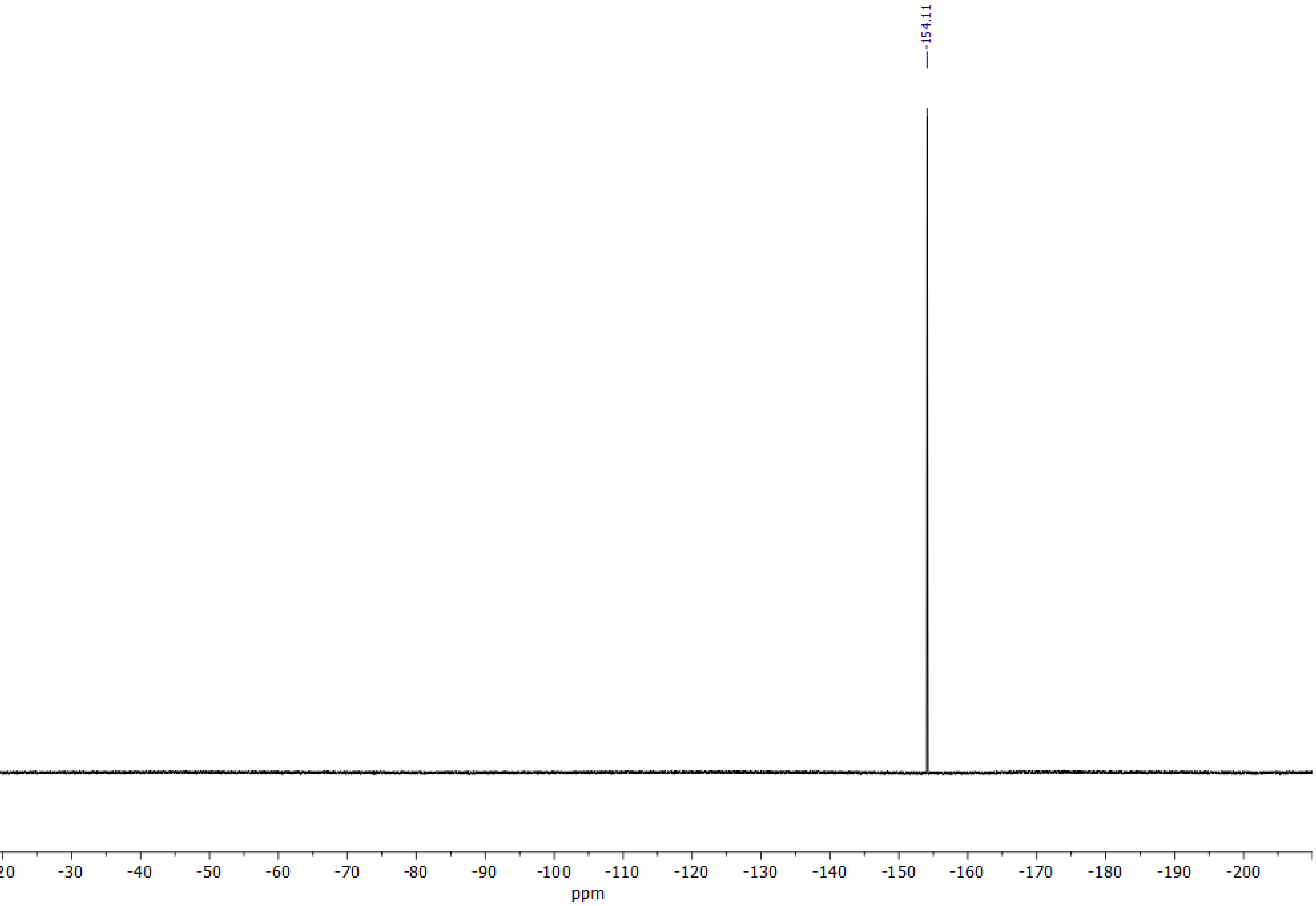

^{1}H NMR of acridine 4

$CDCl_3$, 500 MHz, 25 ºC

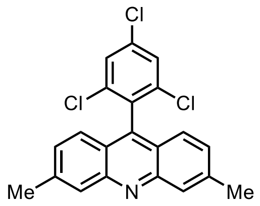

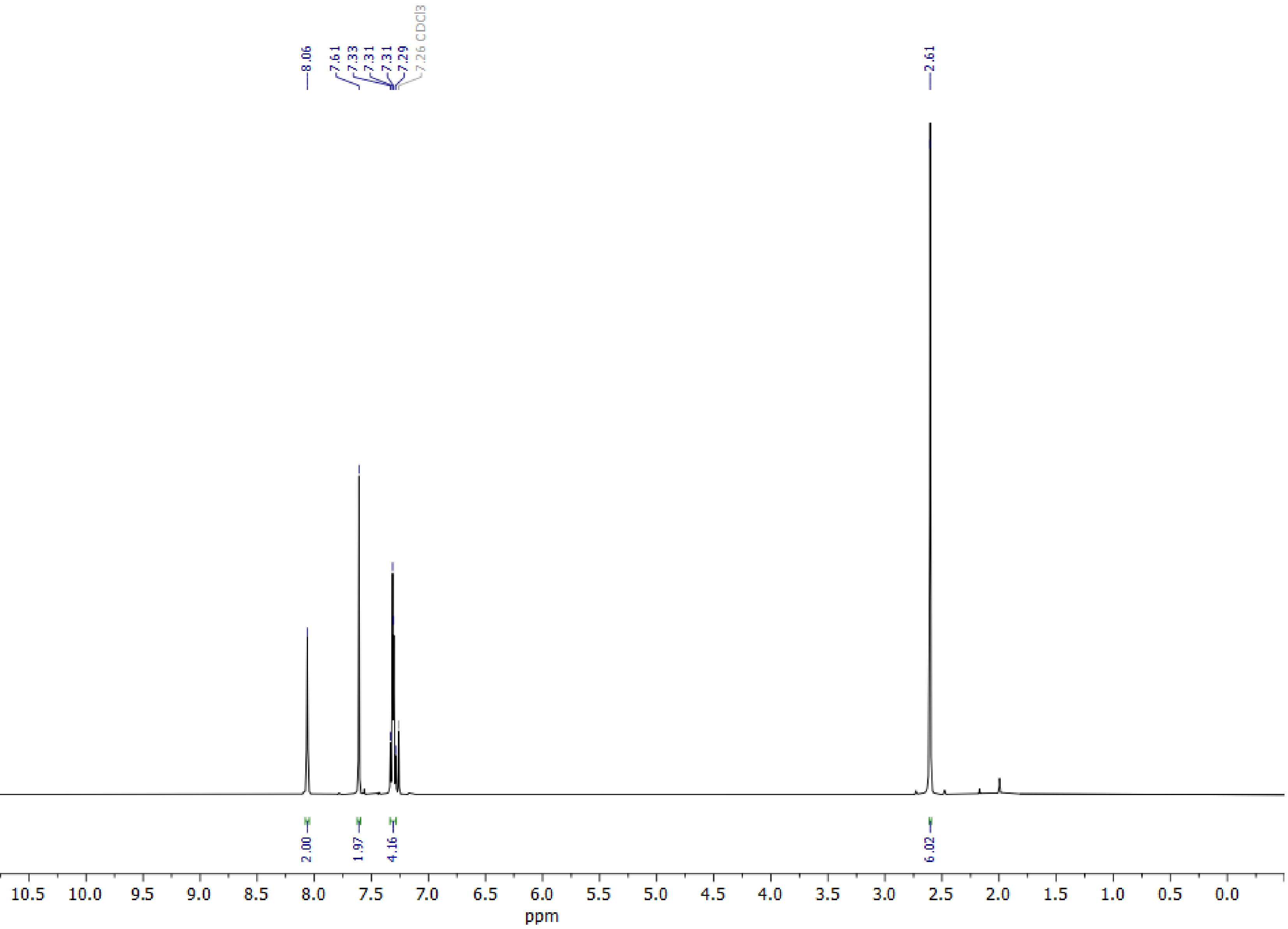

^{13}C NMR of acridine 4

$CDCl_3$, 125 MHz, 25 ºC

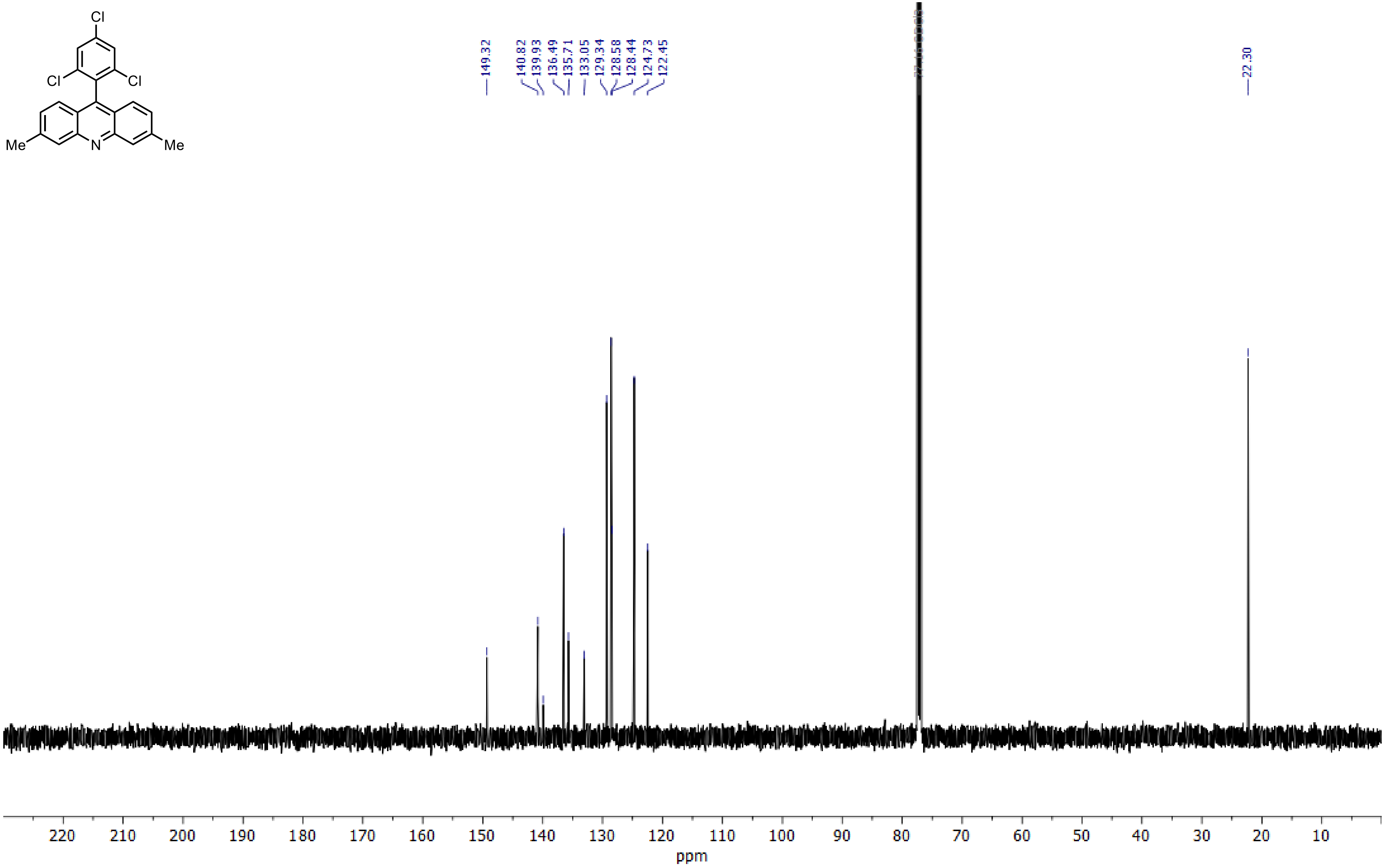

^{1}H NMR of acridinium $1H^{+}BF_4^{-}$

$CDCl_3$, 500 MHz, 25 ºC

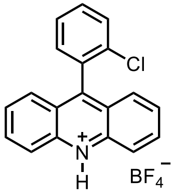

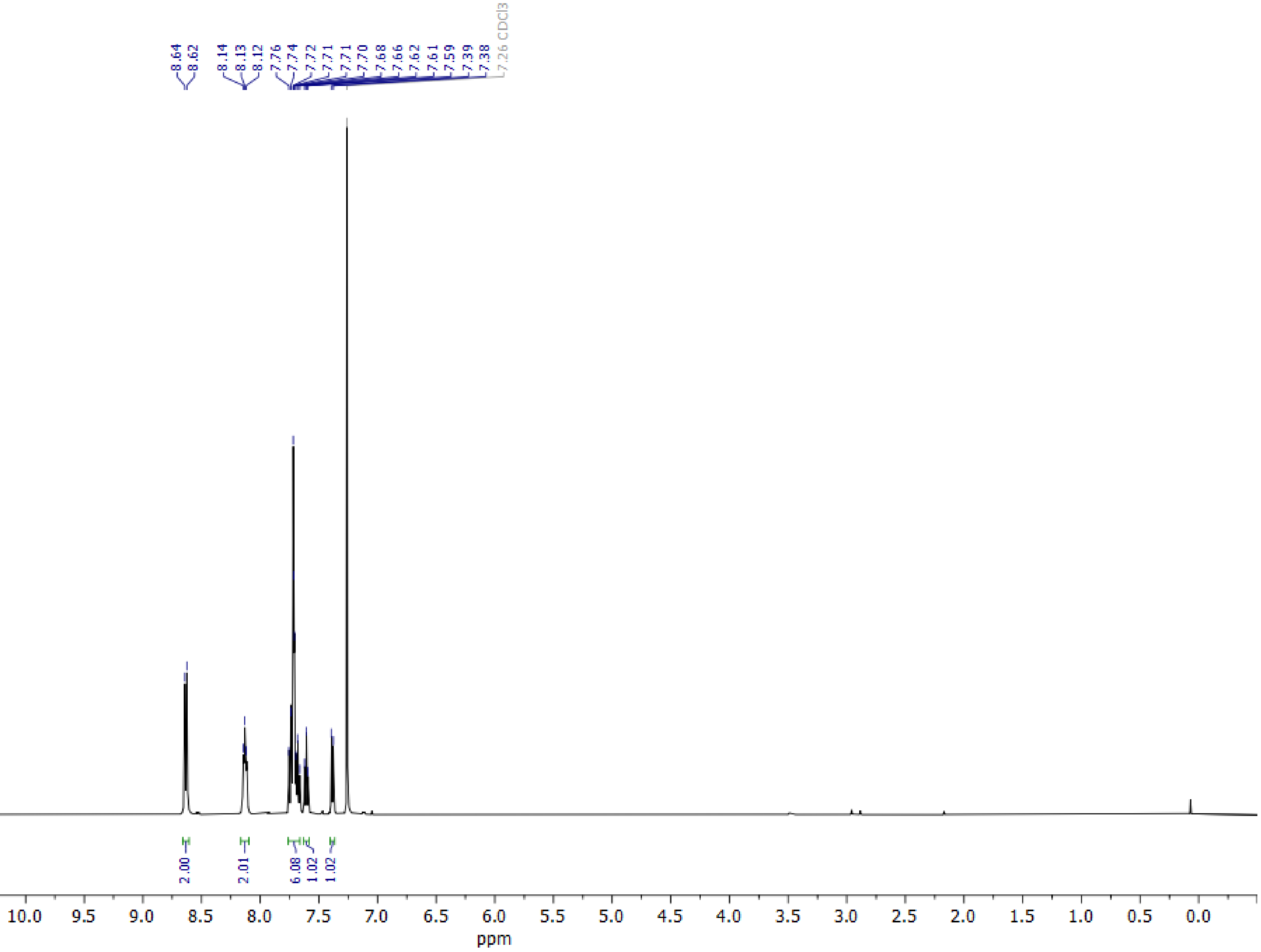

^{13}C NMR of acridinium 1H^{+}BF$_4^{-}$

CDCl$_3$, 150 MHz, 25 ºC

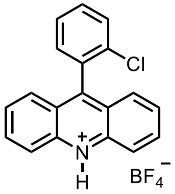

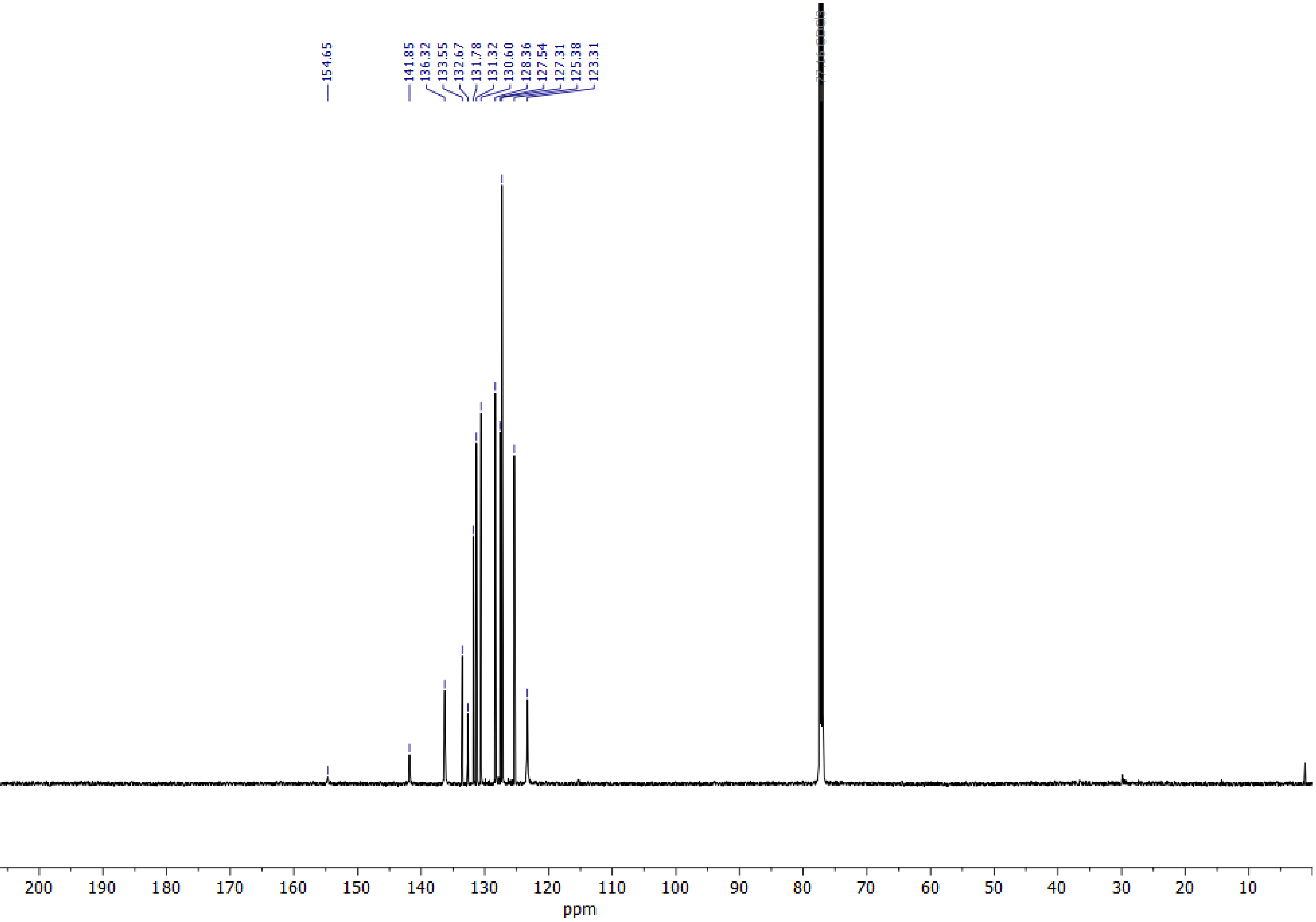

^{19}F NMR of acridinium $1H^+BF_4^-$

$CDCl_3$, 470 MHz, 25 ºC

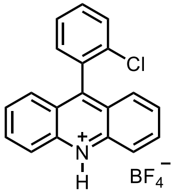

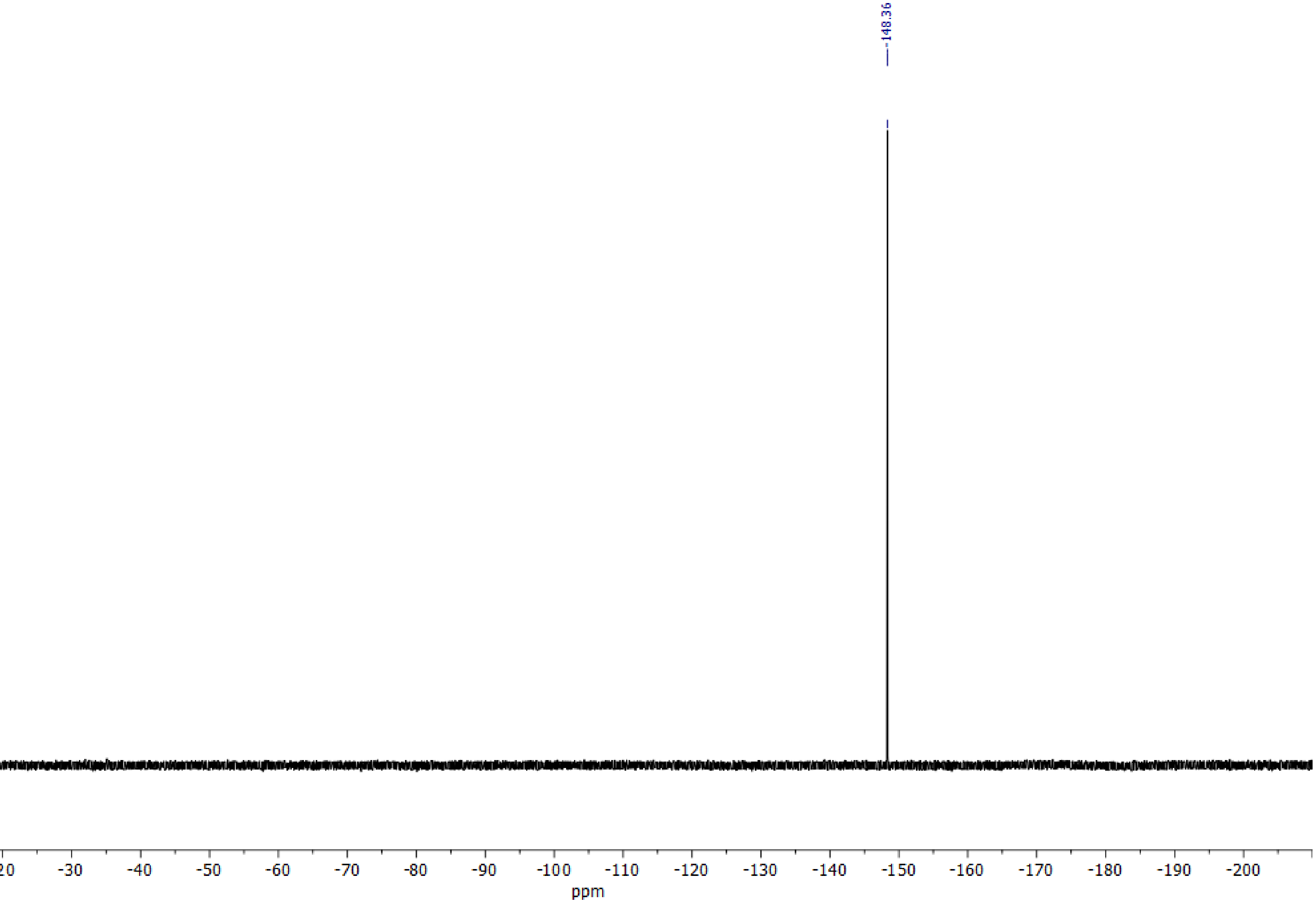

^{1}H NMR of acridinium $2H^+BF_4^-$

$CDCl_3$, 600 MHz, 25 ºC

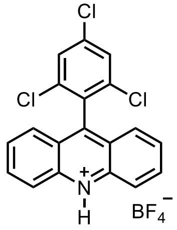

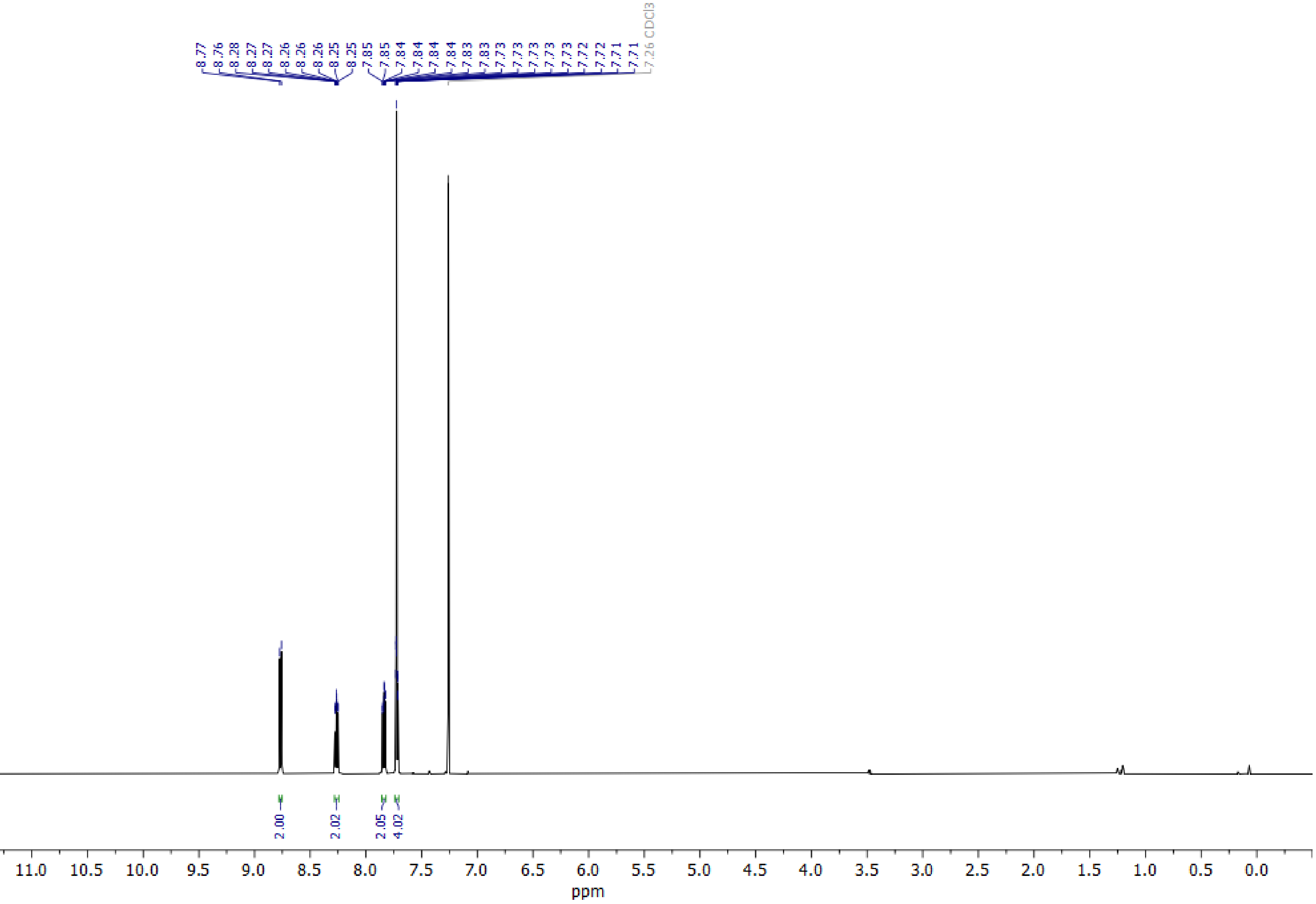

^{13}C NMR of acridinium 2H^{+}BF$_4^{-}$

CDCl$_3$, 150 MHz, 25 ºC

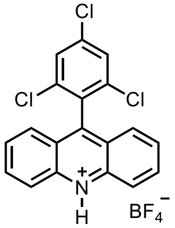

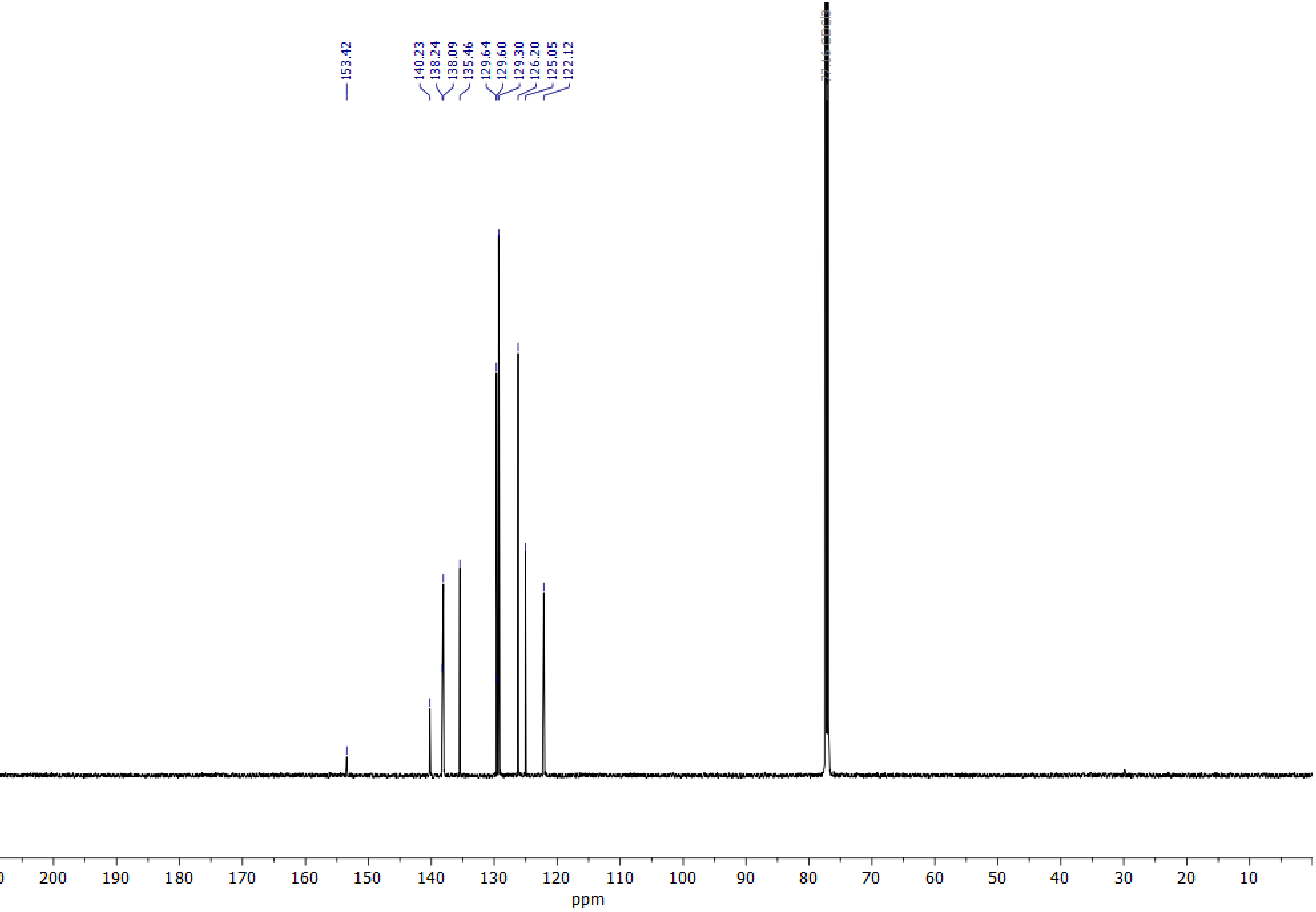

^{19}F NMR of acridinium $2H^{+}BF_4^{-}$

$CDCl_3$, 470 MHz, 25 ºC

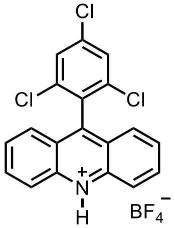

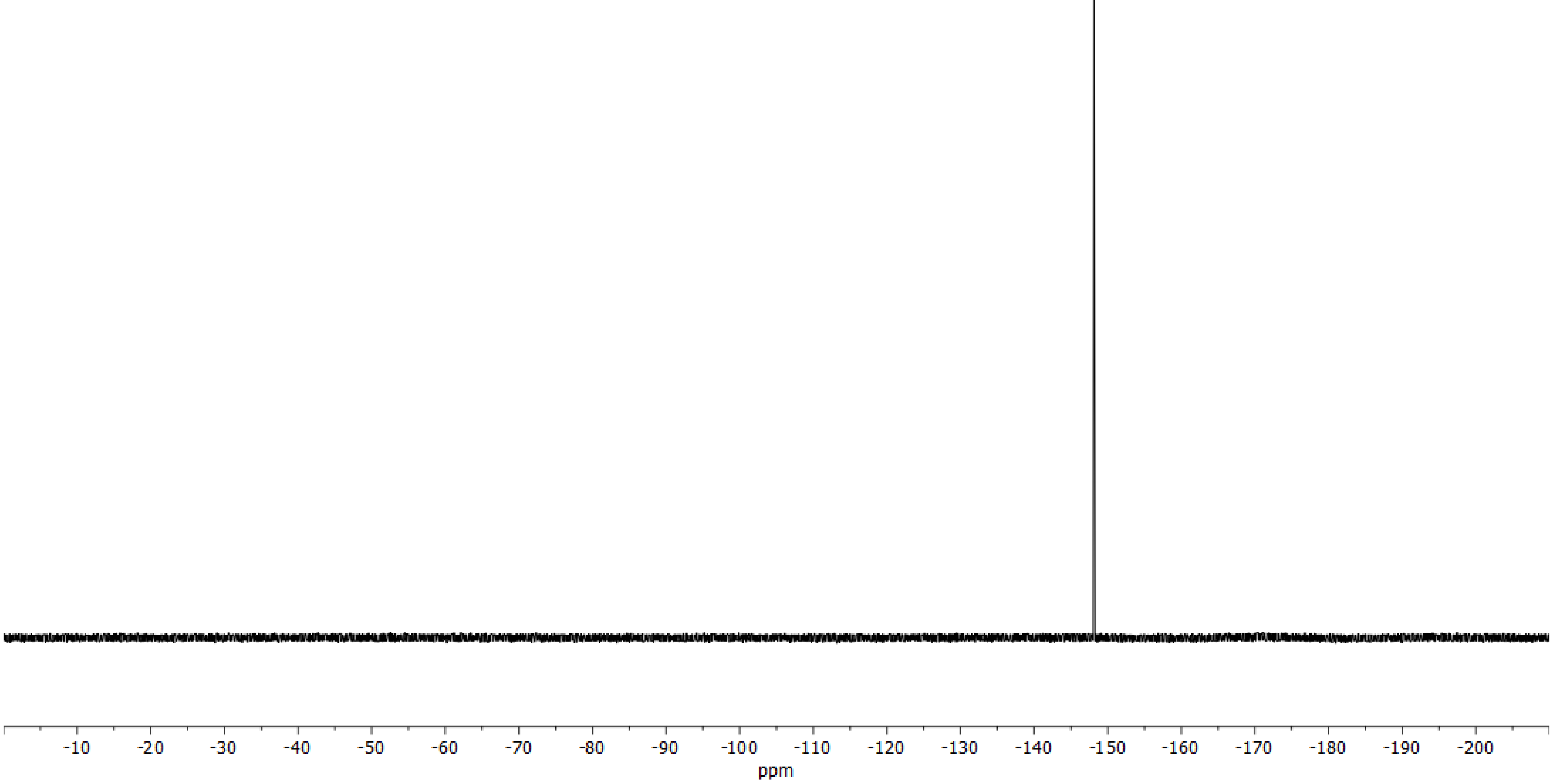

^{1}H NMR of acridinium $4H^{+}BF_4^{-}$

$CDCl_3$, 500 MHz, 25 ºC

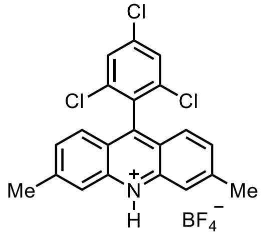

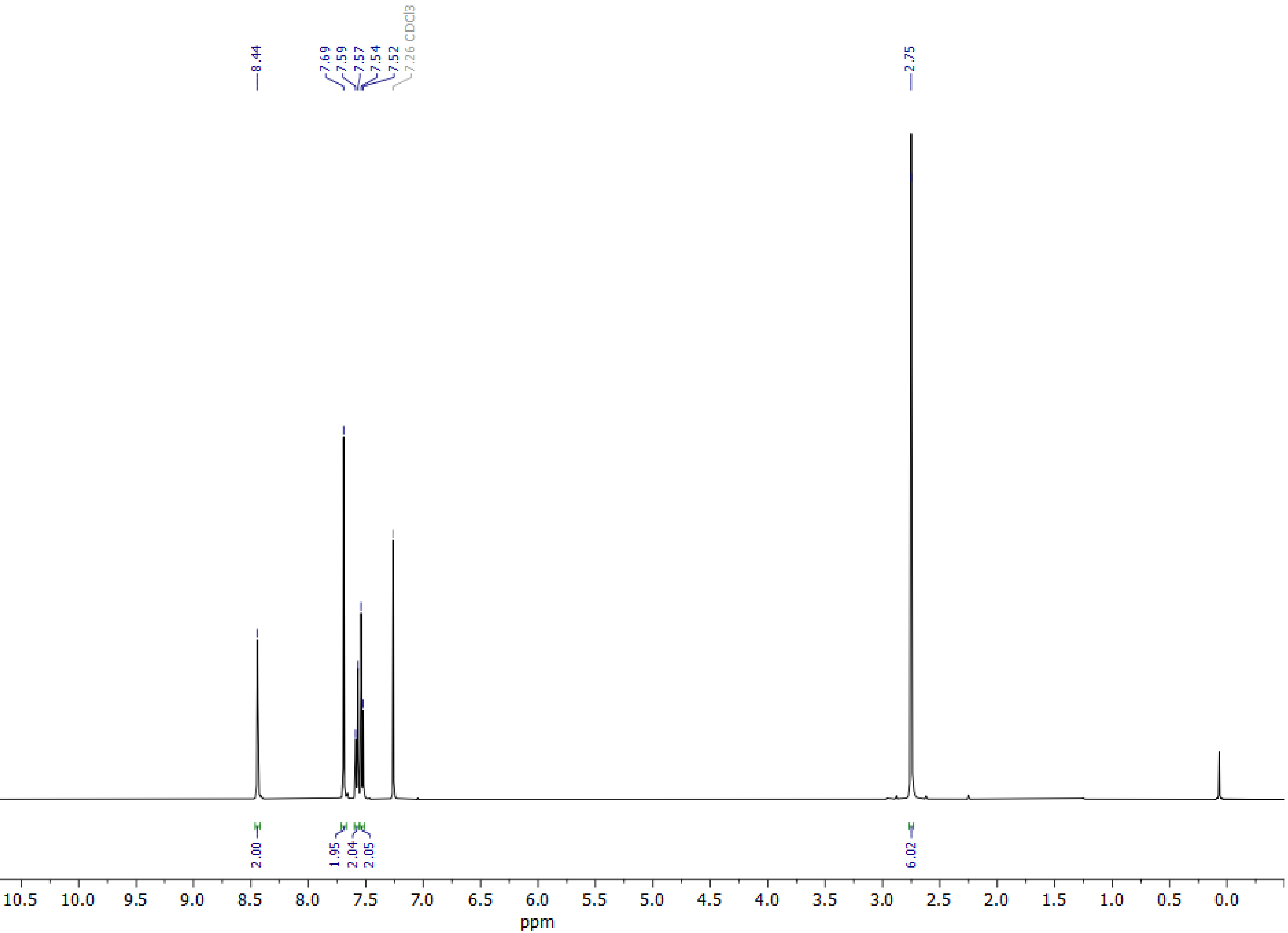

^{13}C NMR of acridinium $4H^{+}BF_4^{-}$

$CDCl_3$, 125 MHz, 25 ºC

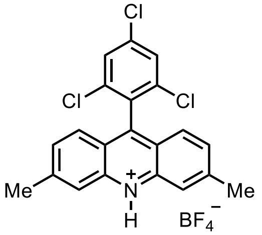

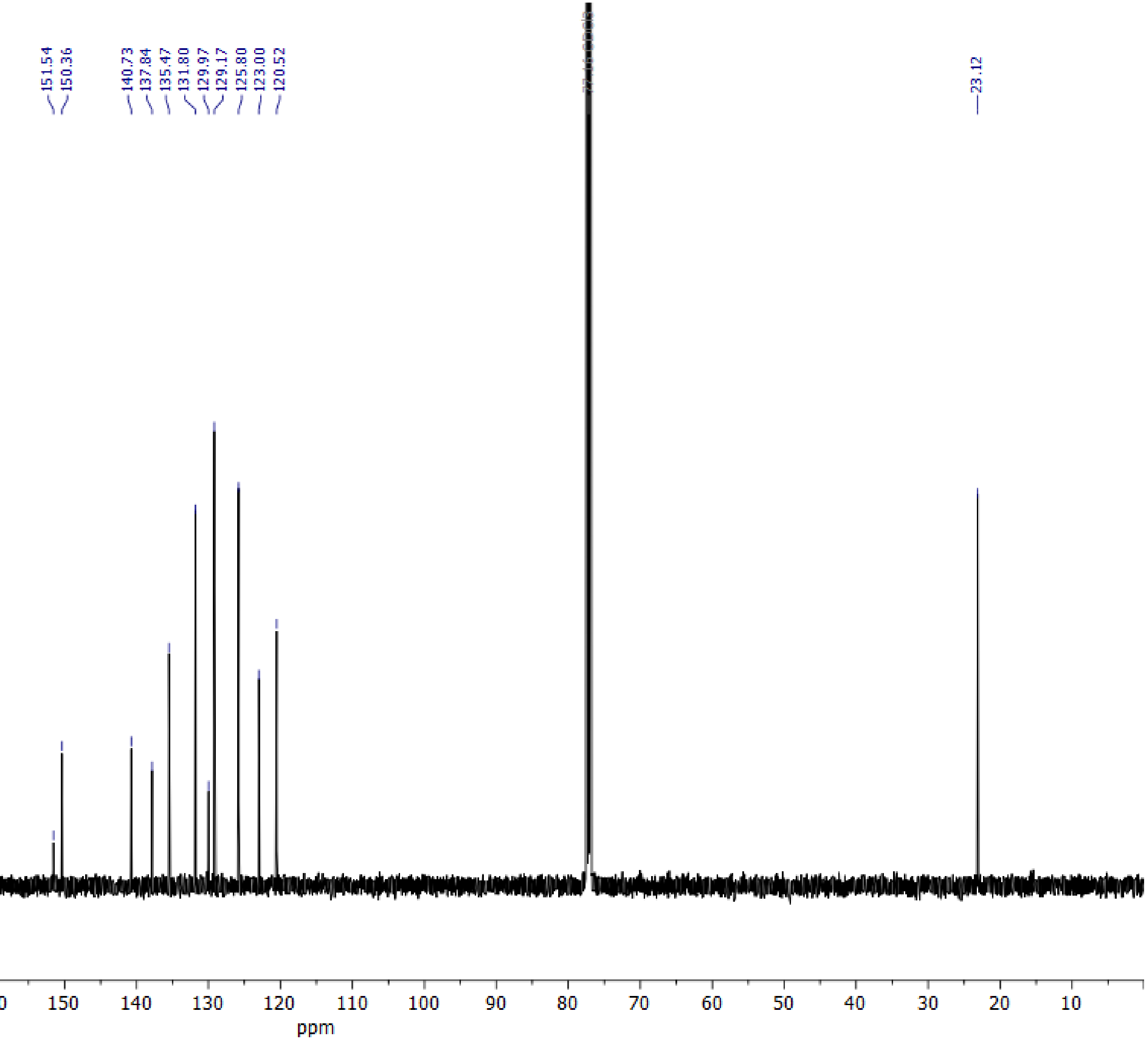

^{19}F NMR of acridinium $4H^{+}BF_4^{-}$

$CDCl_3$, 470 MHz, 25 ºC

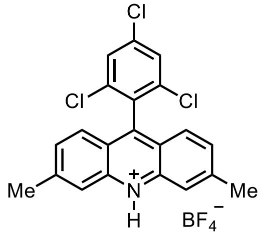

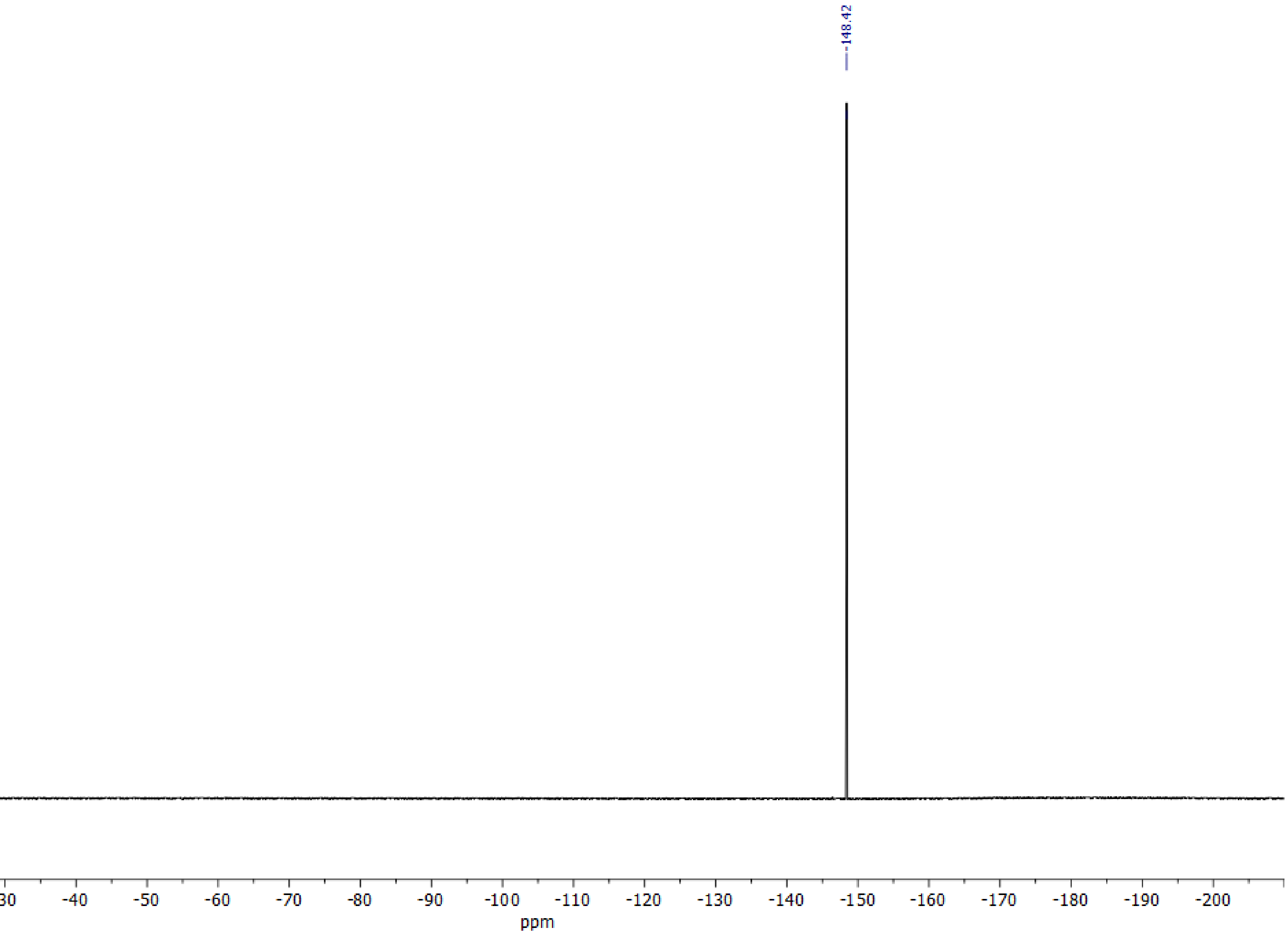

^{1}H NMR of olefin 5c

$CDCl_3$, 500 MHz, 25 ºC

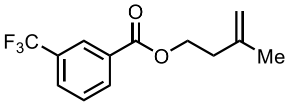

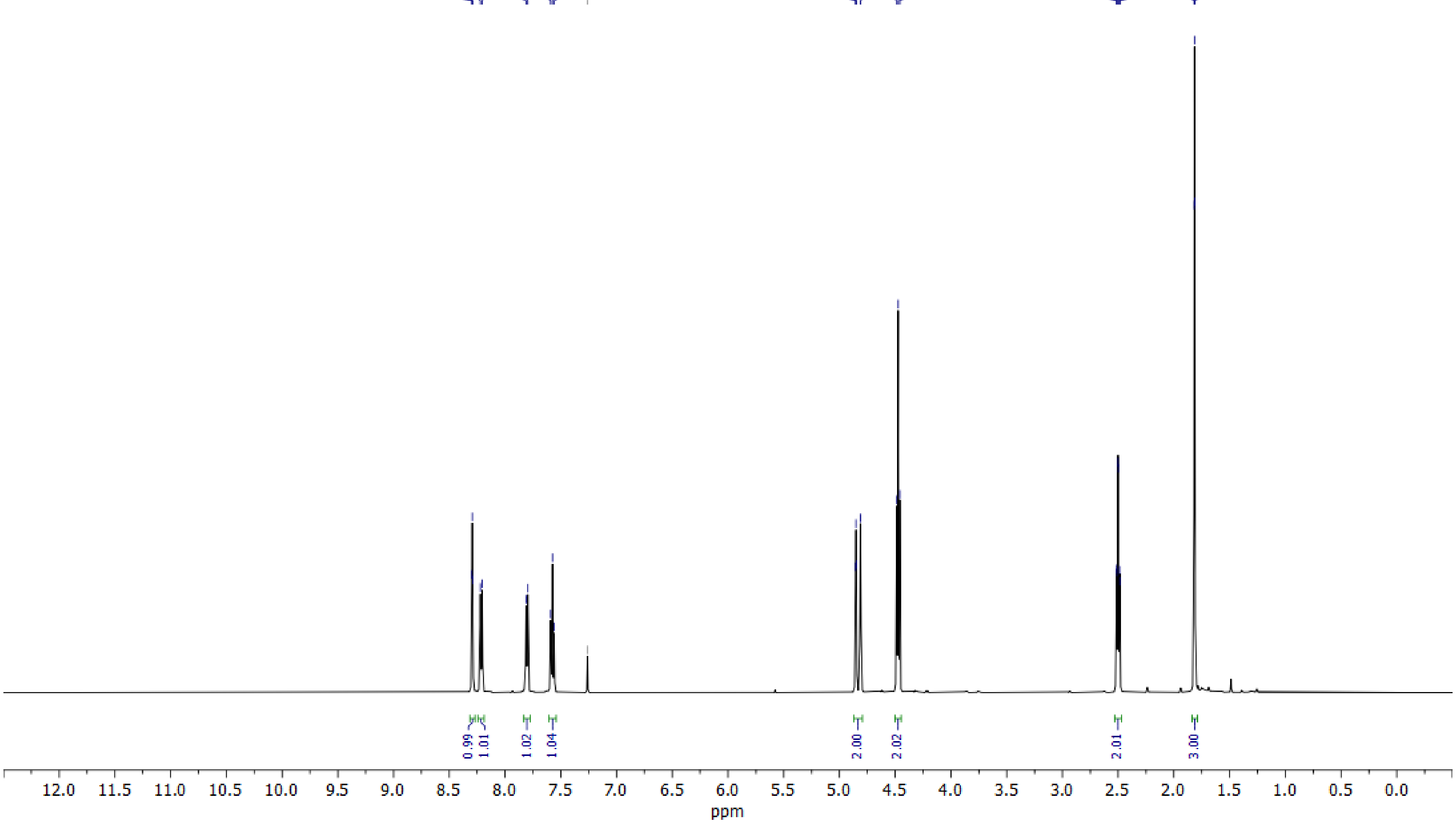

^{13}C NMR of olefin 5c

$CDCl_3$, 125 MHz, 25 ºC

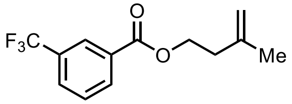

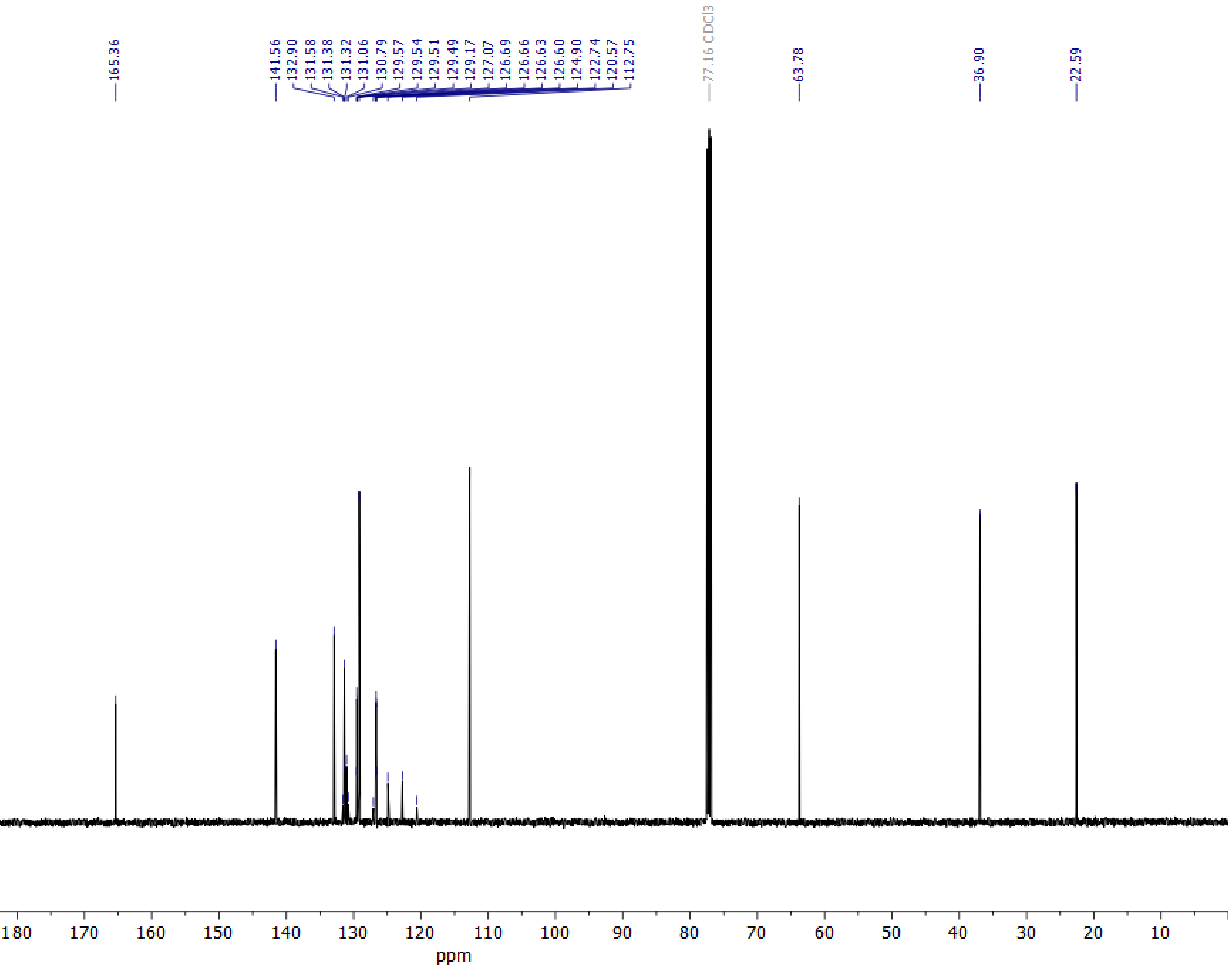

^{19}F NMR of olefin 5c

$CDCl_3$, 470 MHz, 25 ºC

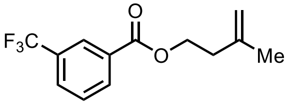

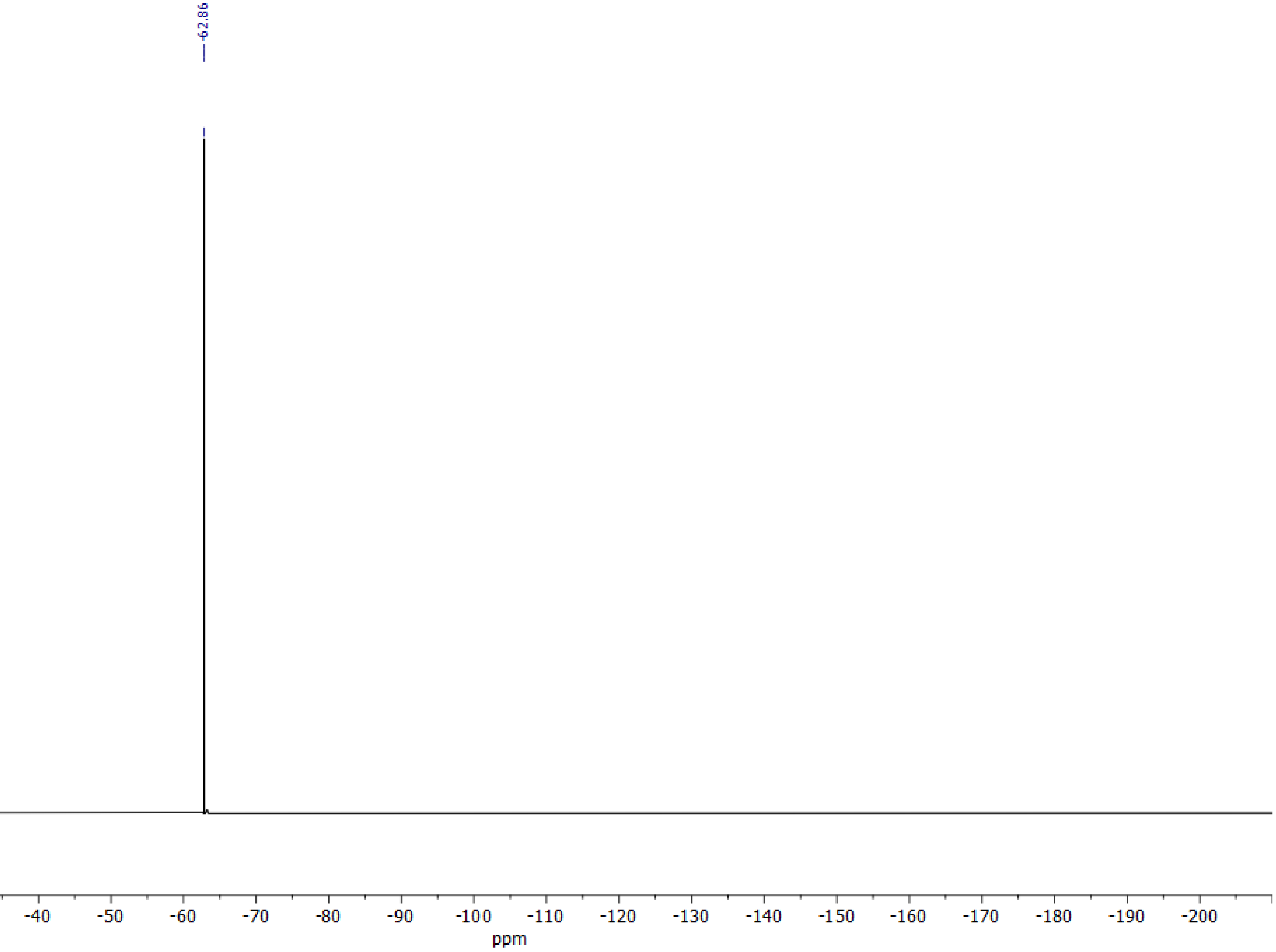

^{1}H NMR of olefin 5d

$CDCl_3$, 500 MHz, 25 ºC

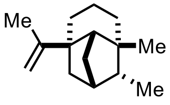

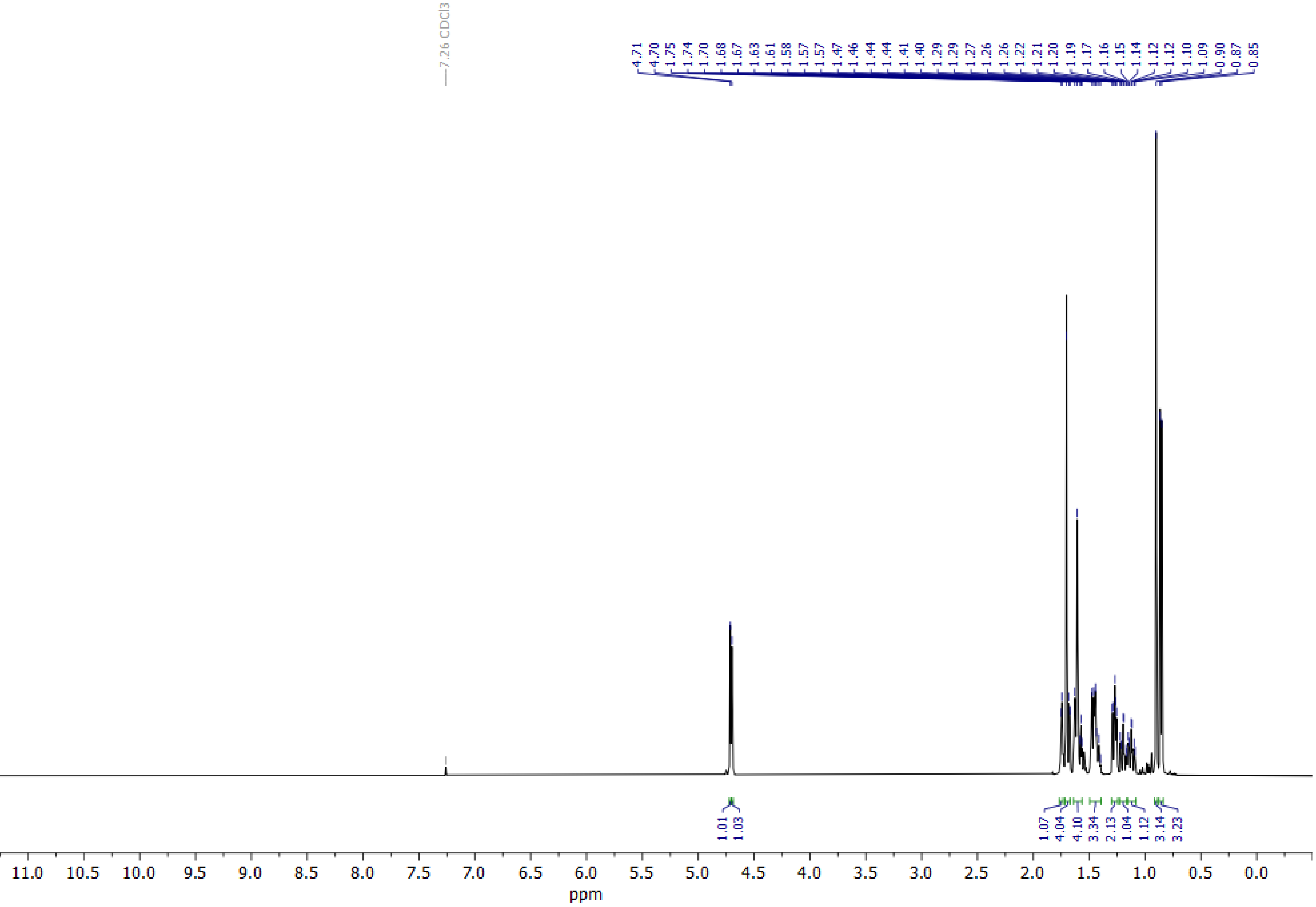

^{13}C NMR of olefin 5d

$CDCl_3$, 125 MHz, 25 ºC

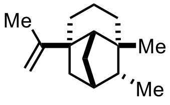

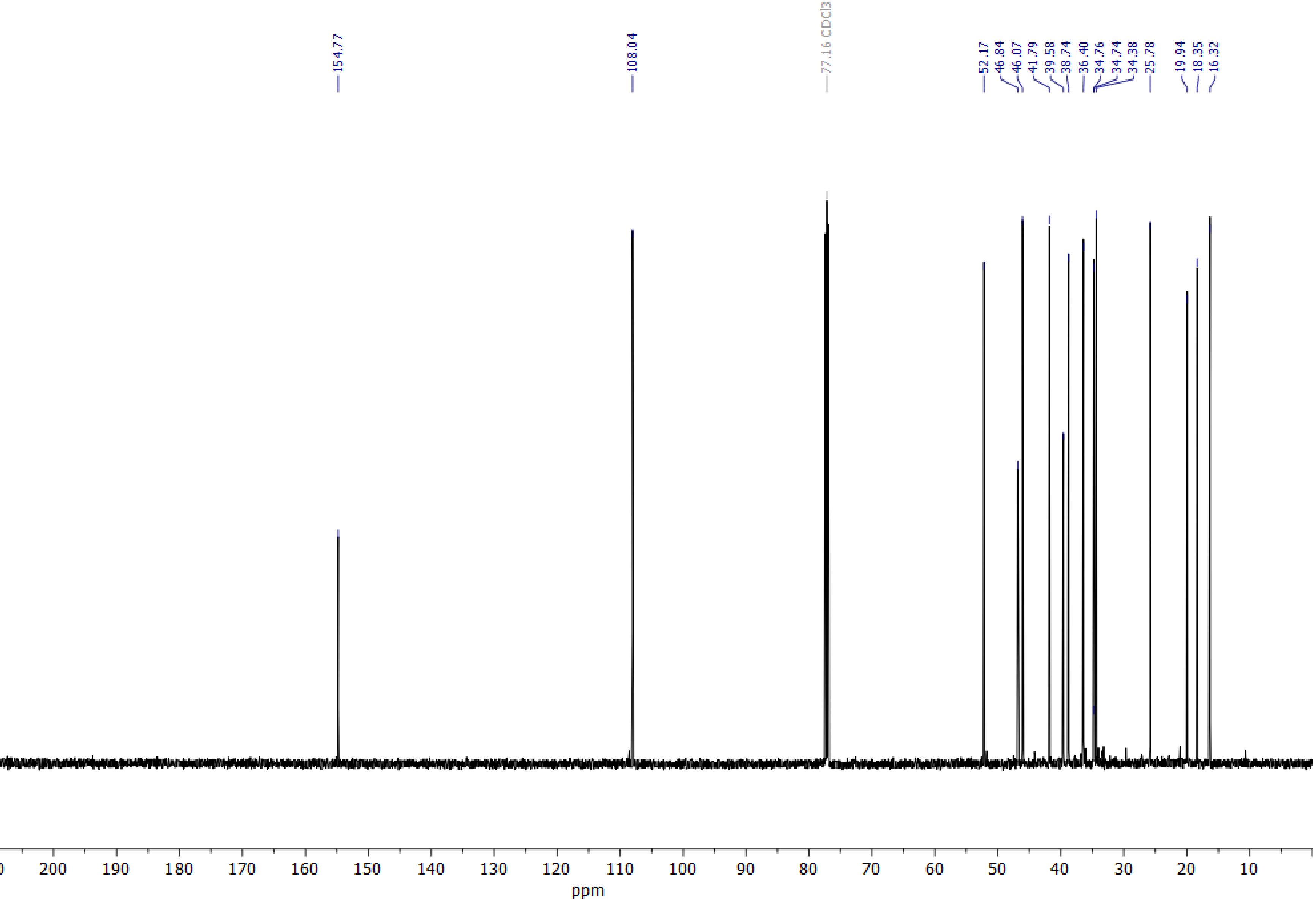

^{1}H NMR of olefin 5h

CDCl3, 500 MHz, 25 ºC

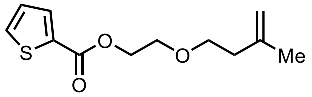

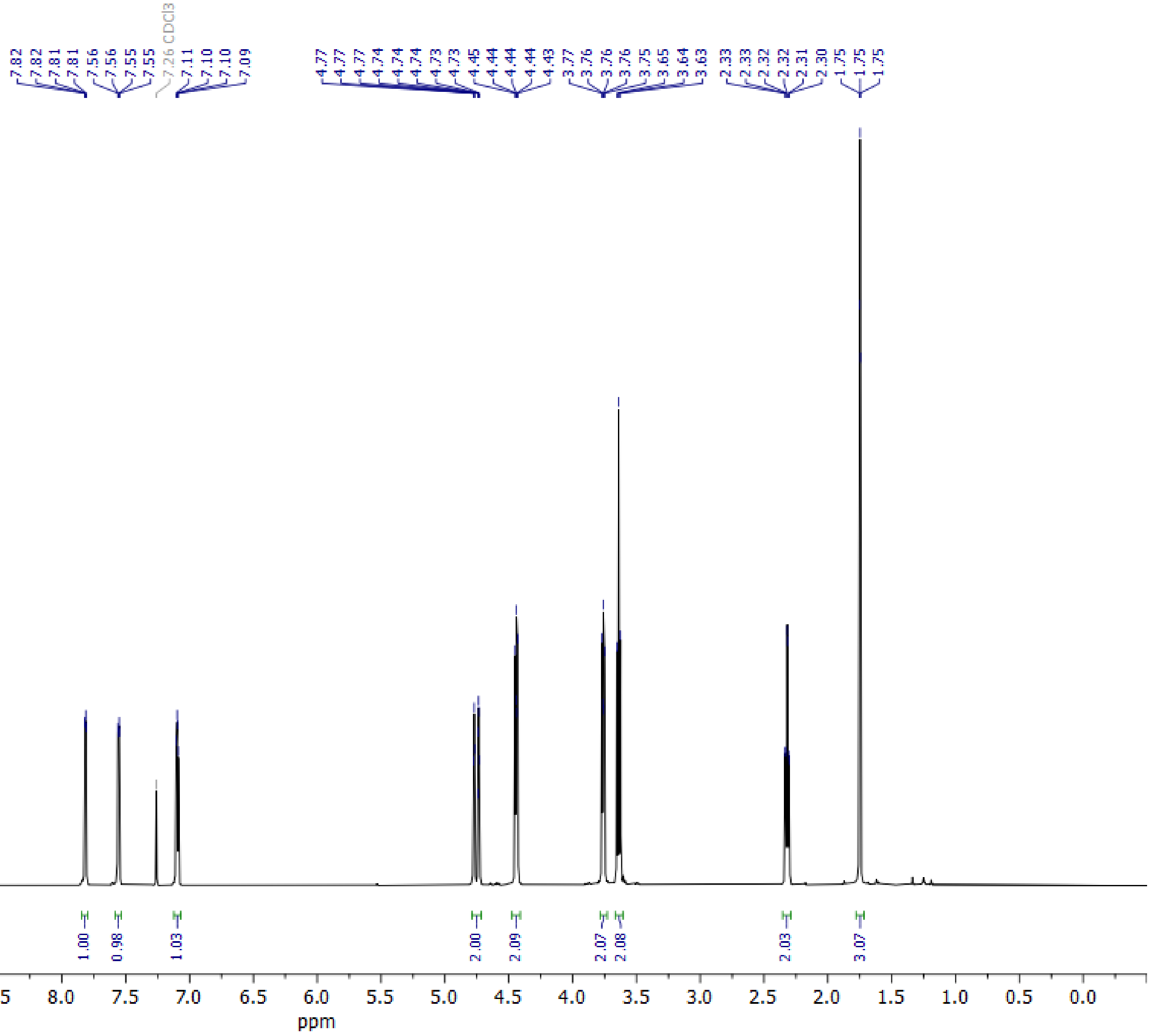

^{13}C NMR of olefin 5h

$CDCl_3$, 125 MHz, 25 ºC

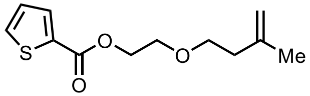

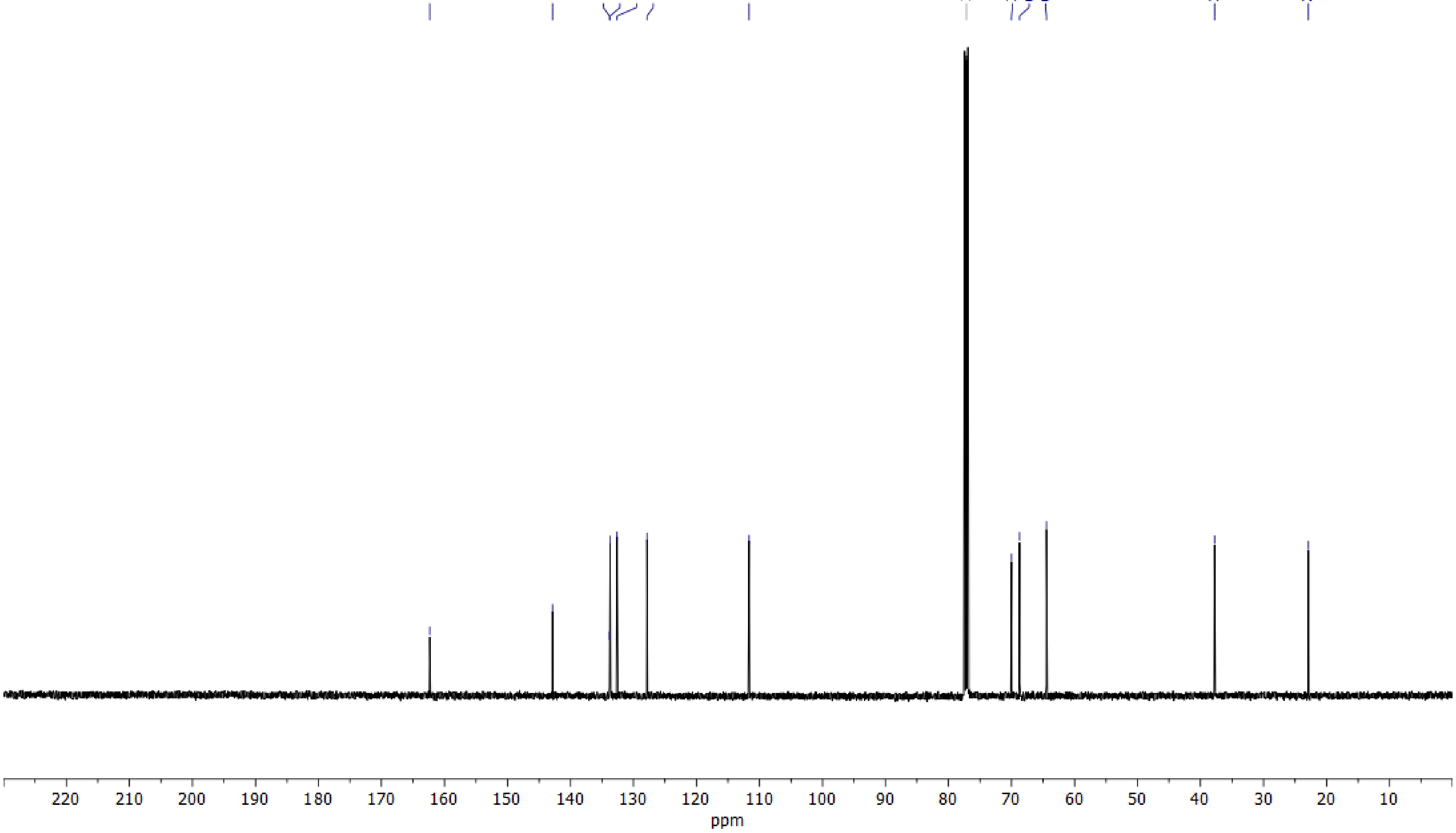

^{1}H NMR of olefin 5j

$CDCl_3$, 500 MHz, 25 ºC

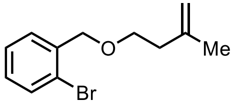

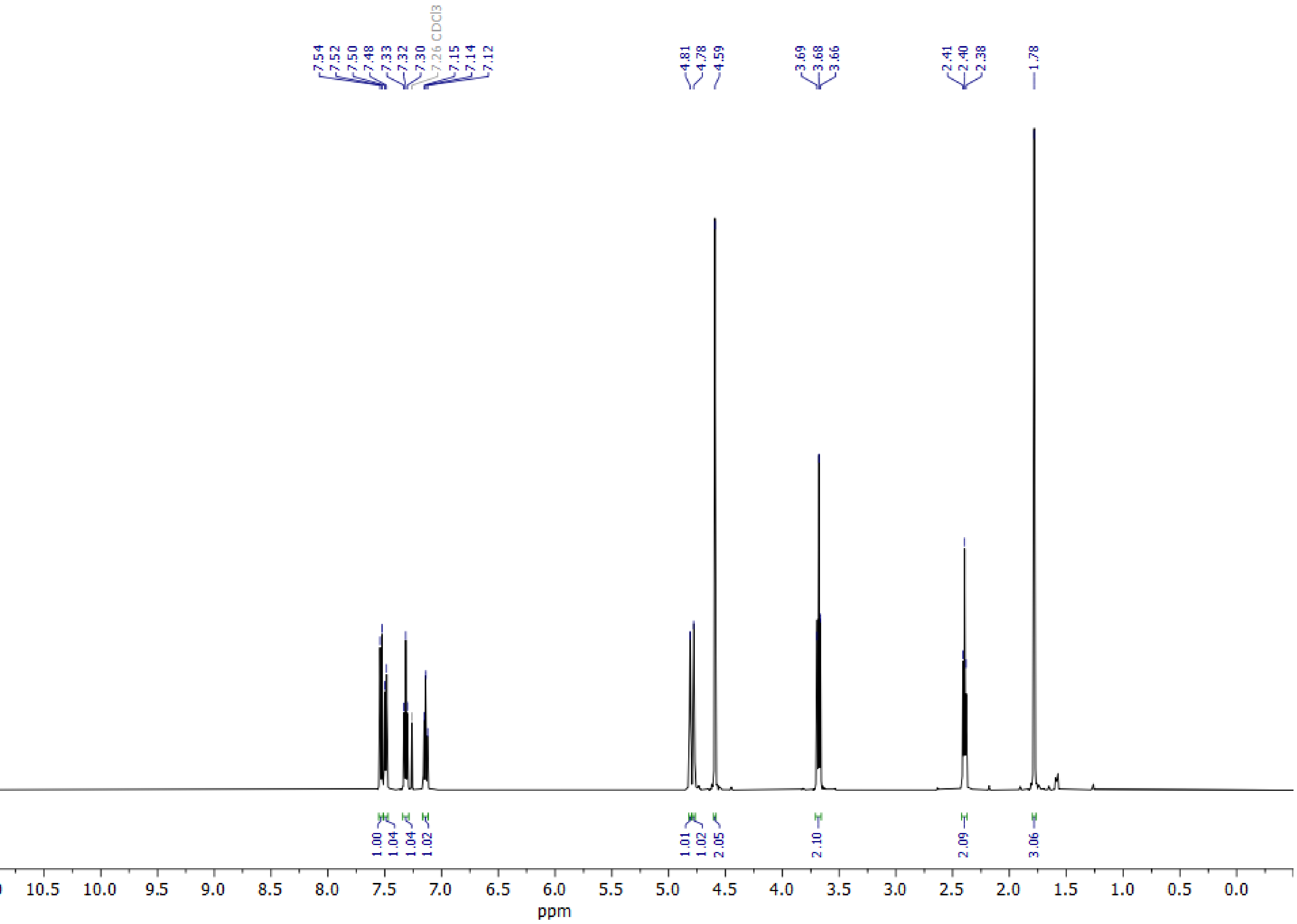

^{13}C NMR of olefin 5j

$CDCl_3$, 125 MHz, 25 ºC

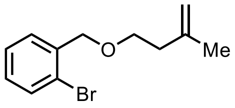

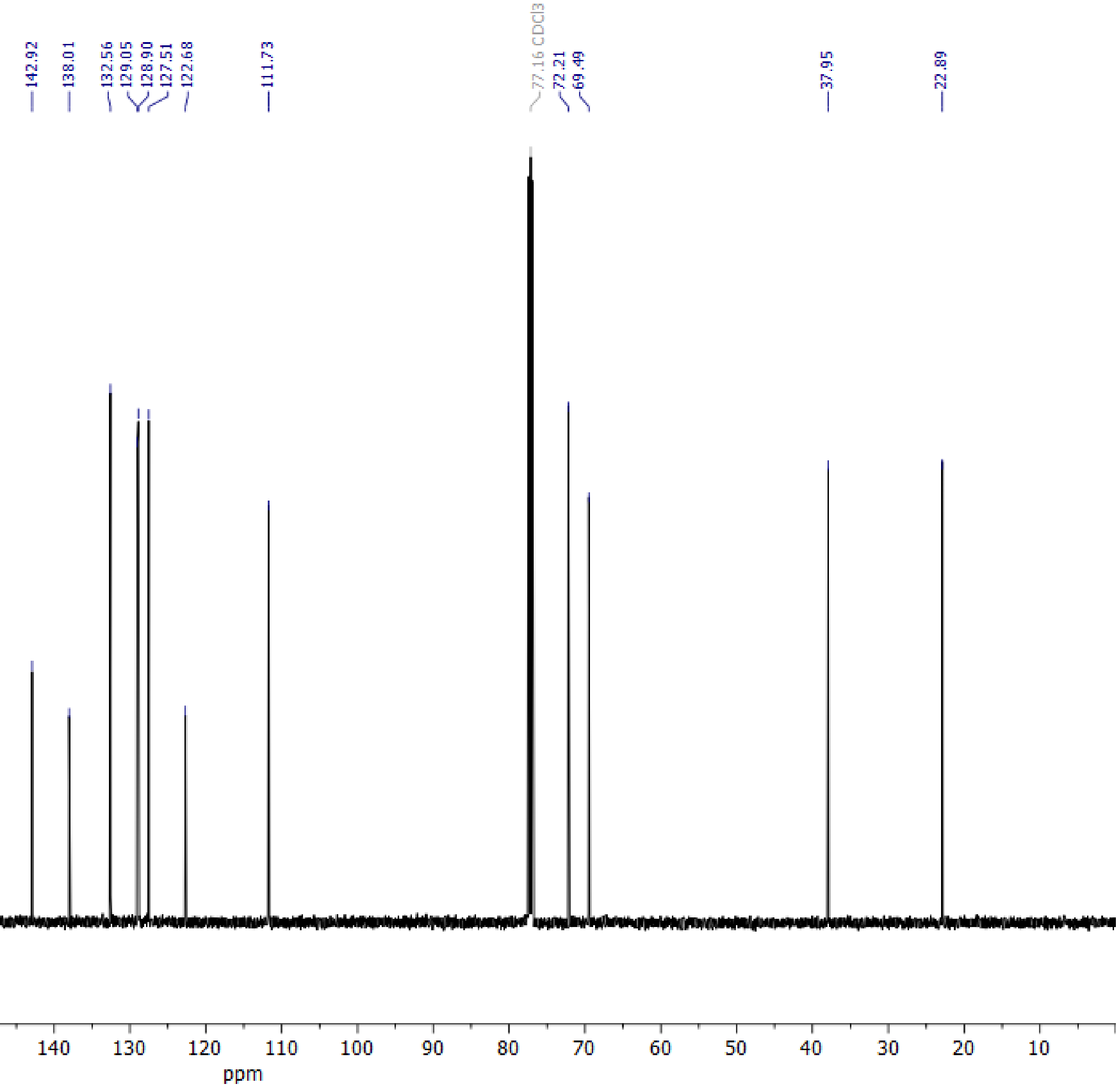

^{1}H NMR of olefin 5n

$CDCl_3$, 500 MHz, 25 ºC

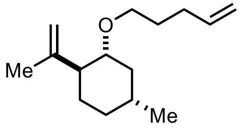

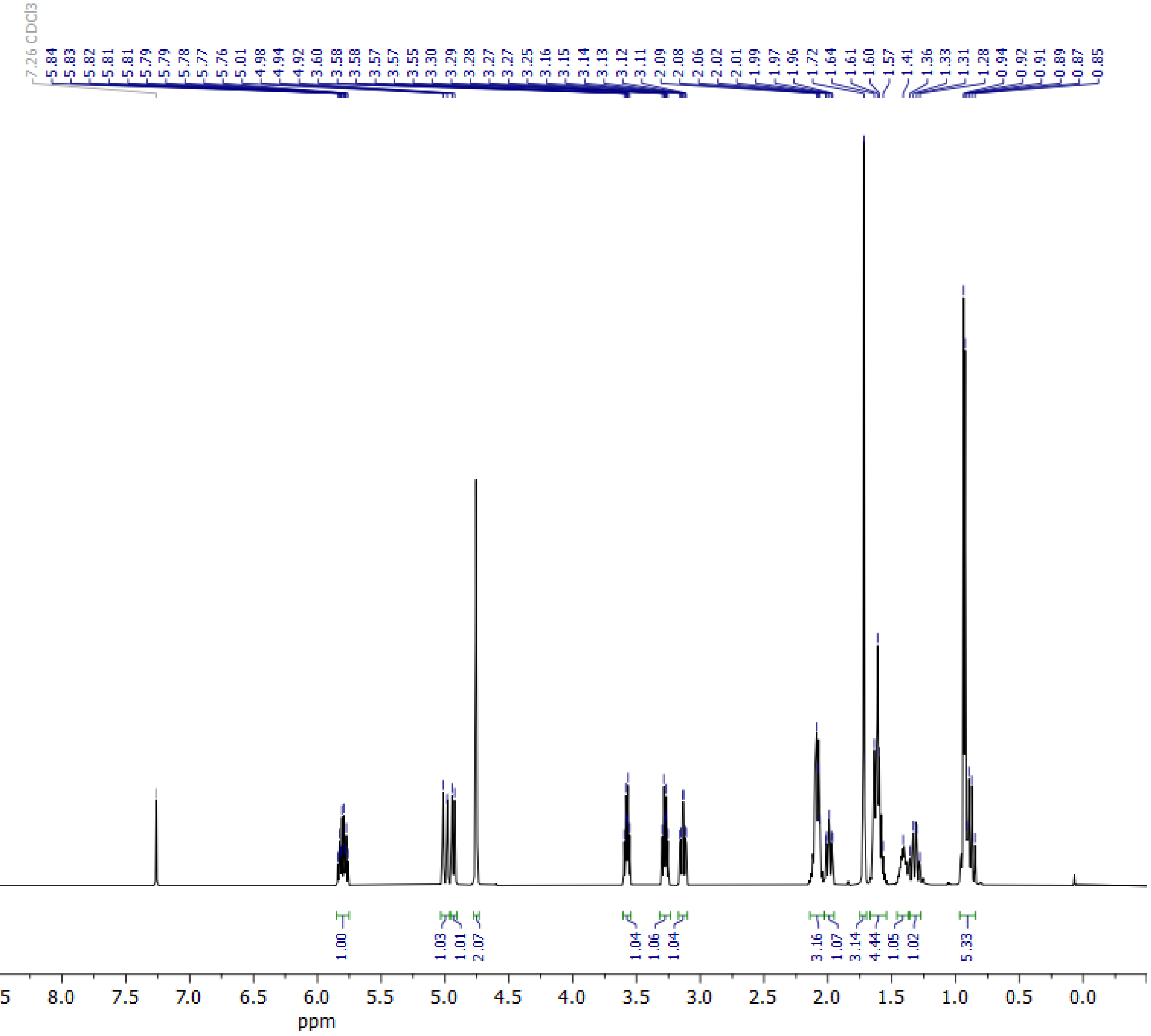

^{13}C NMR of olefin 5n

CDCl3, 125 MHz, 25 ºC

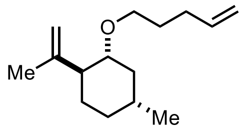

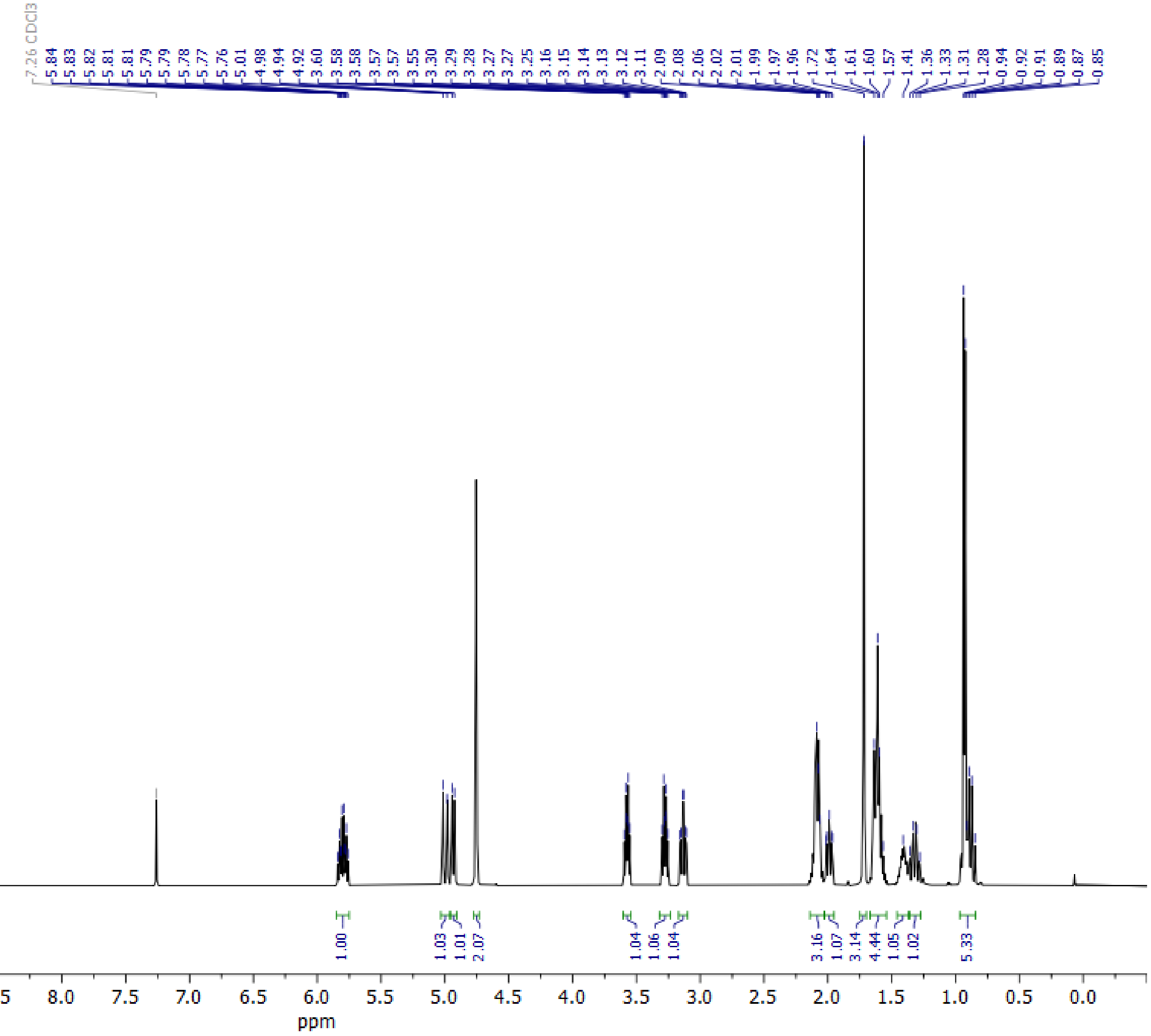

^{1}H NMR of 1-pentyl-1-cyclopentene

$CDCl_3$, 500 MHz, 25 ºC

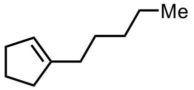

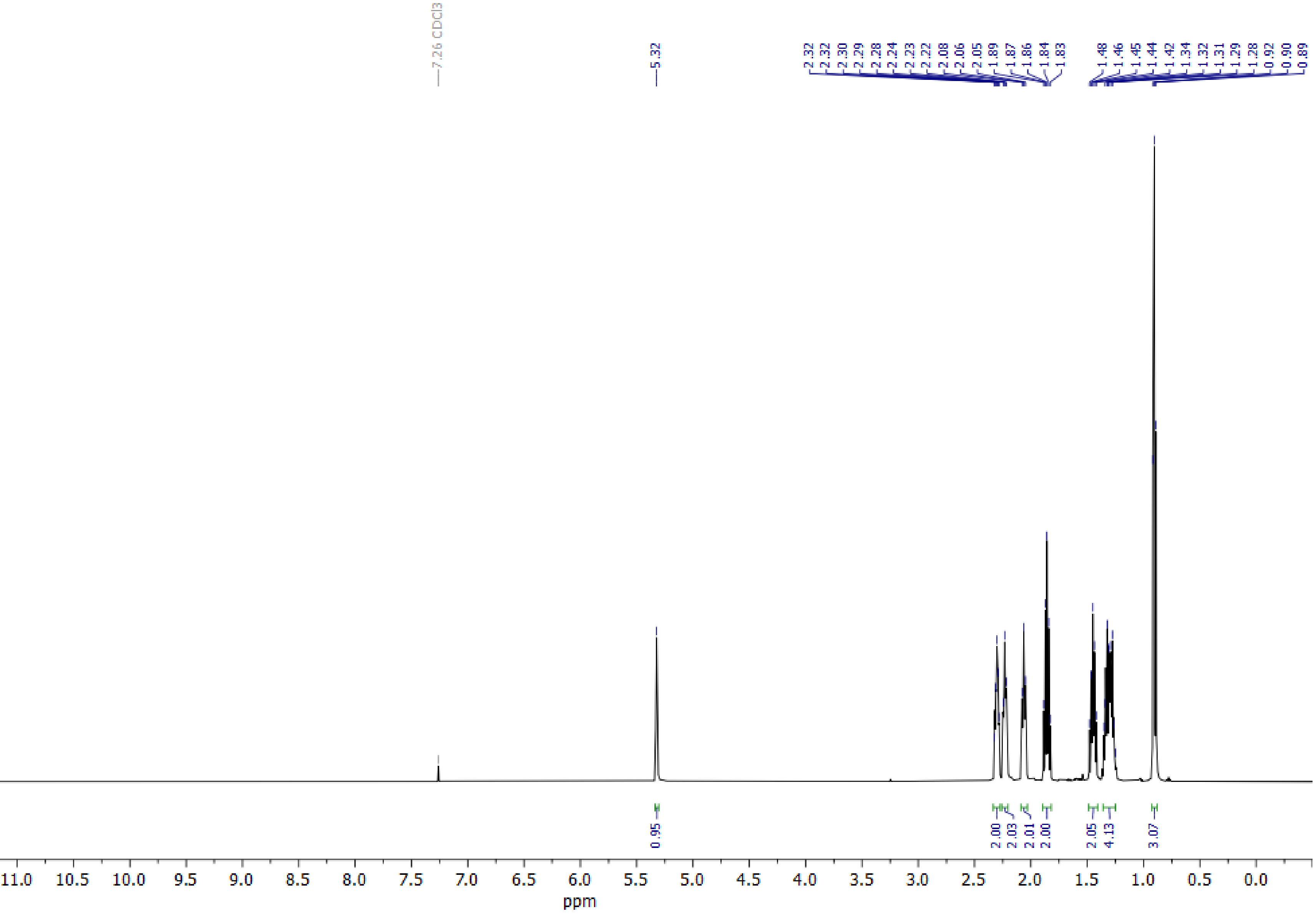

^{13}C NMR of 1-pentyl-1-cyclopentene

$CDCl_3$, 125 MHz, 25 ºC

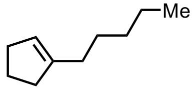

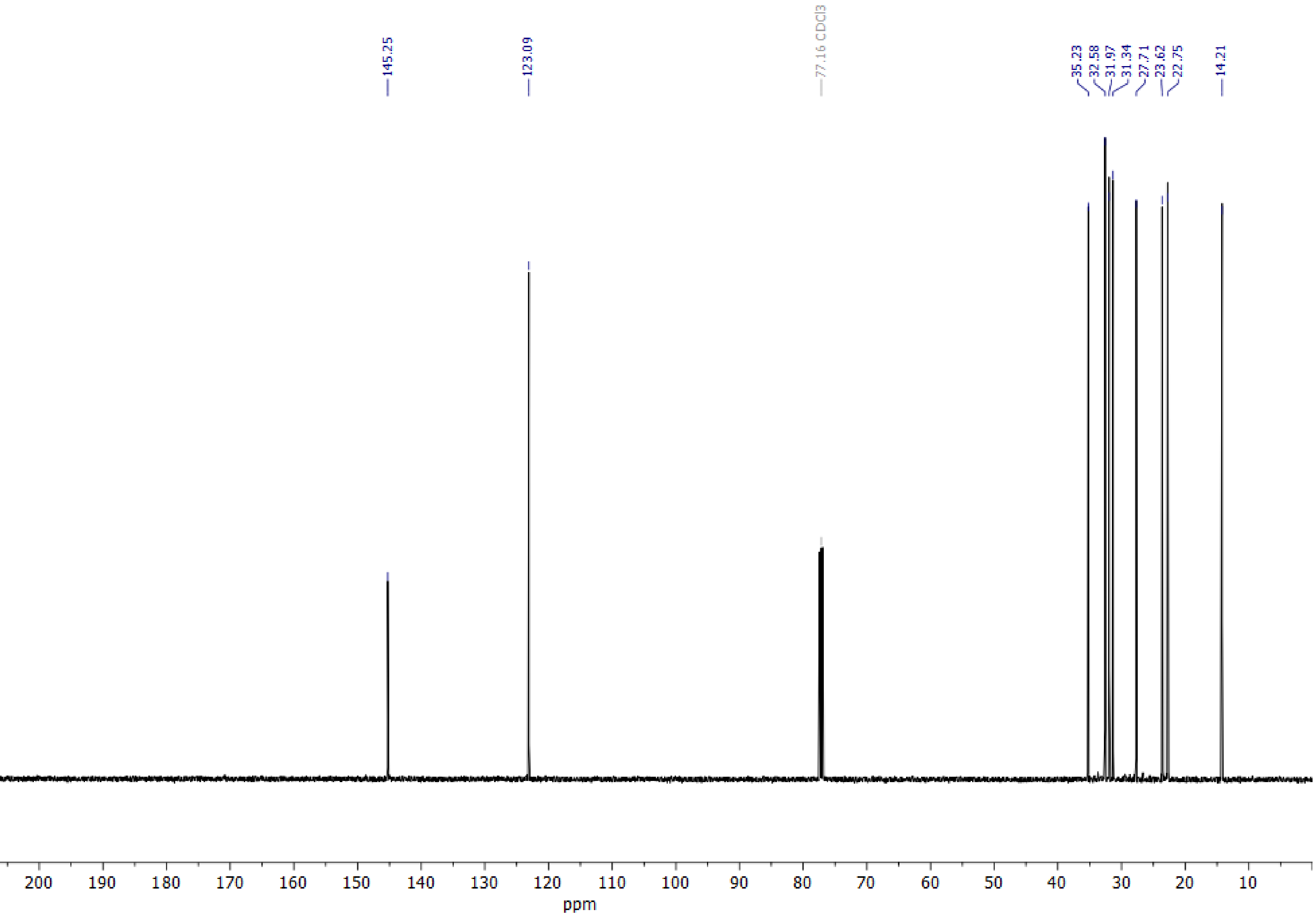

^{1}H NMR of 2-((3-methylbut-3-en-1-yl)oxy)ethan-1-ol

$CDCl_3$, 500 MHz, 25 ºC

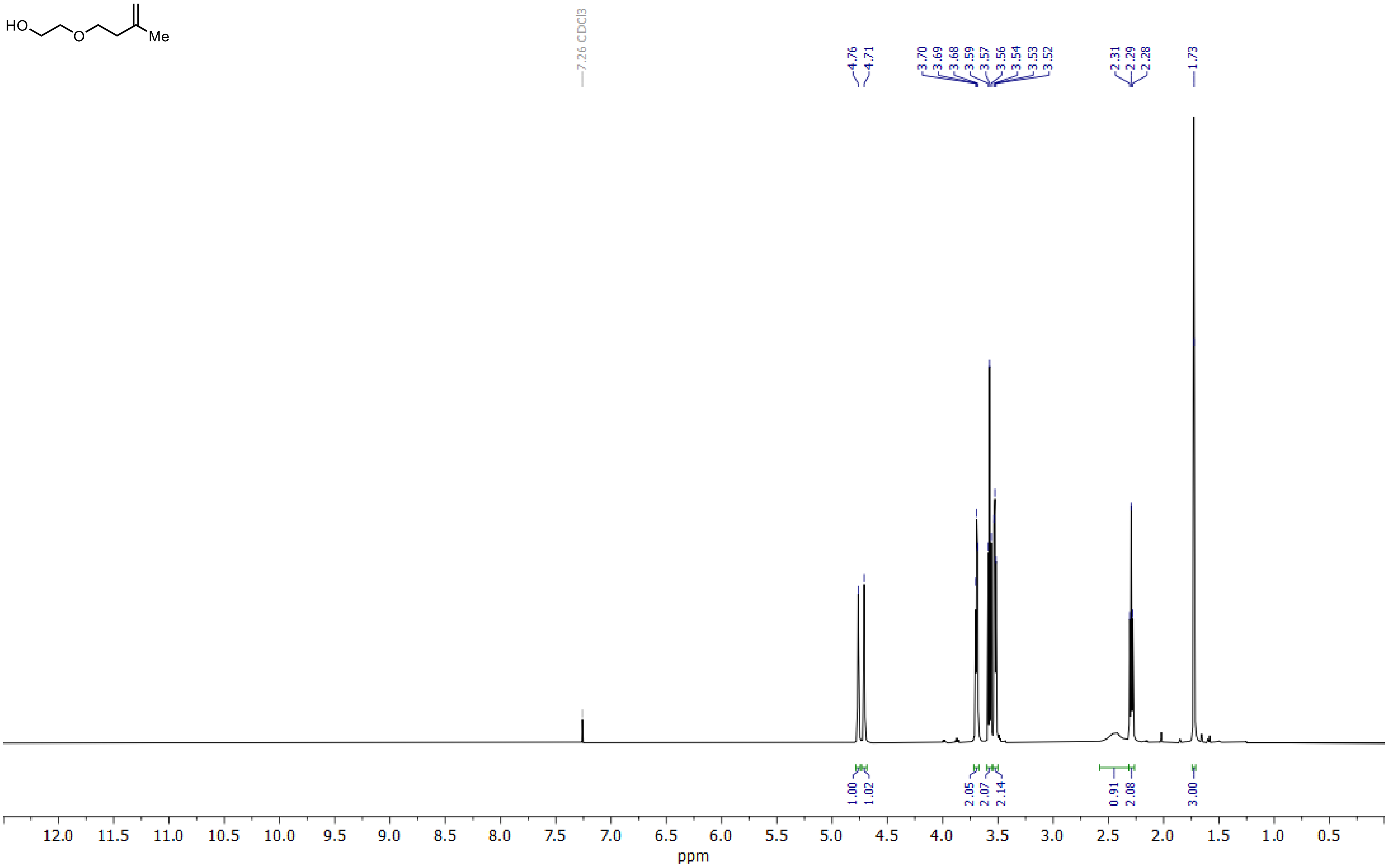

^{13}C NMR of 2-((3-methylbut-3-en-1-yl)oxy)ethan-1-ol

$CDCl_3$, 125 MHz, 25 ºC

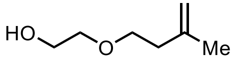

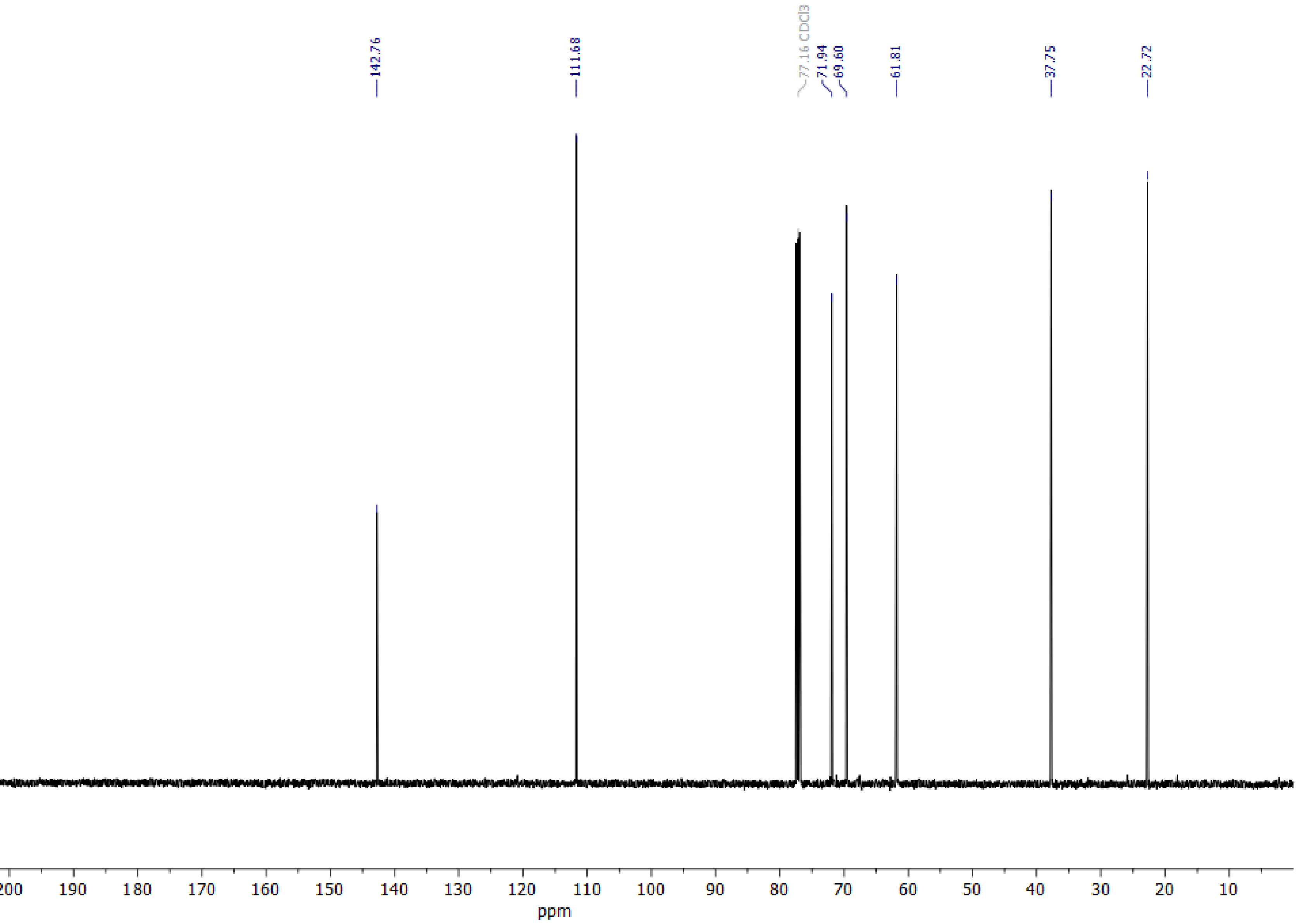

^{1}H NMR of primary alcohol 6a

$CDCl_3$, 500 MHz, 25 ºC

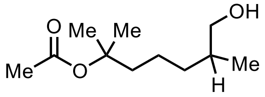

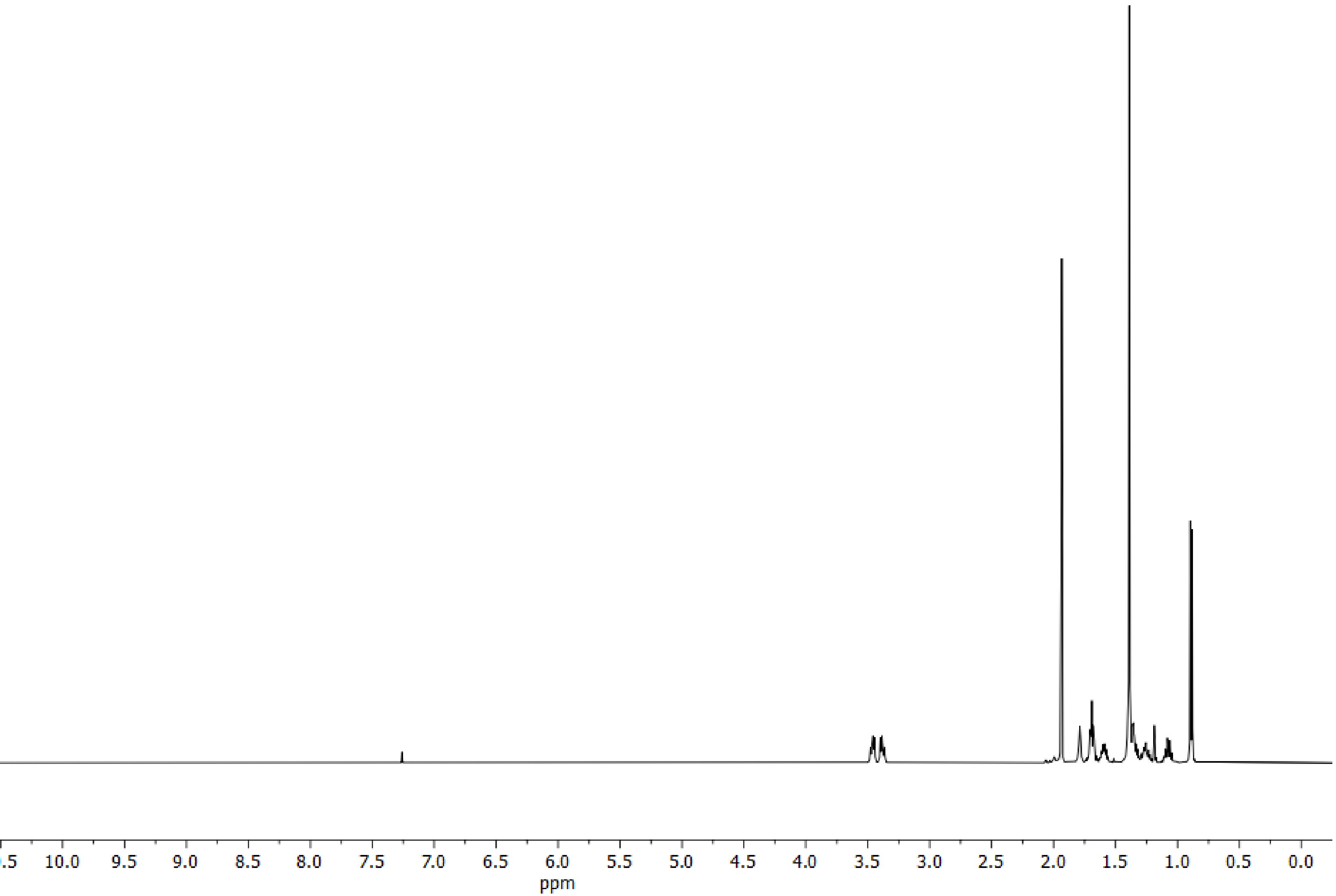

^{13}C NMR of primary alcohol 6a

$CDCl_3$, 125 MHz, 25 ºC

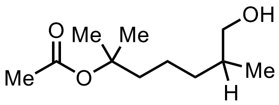

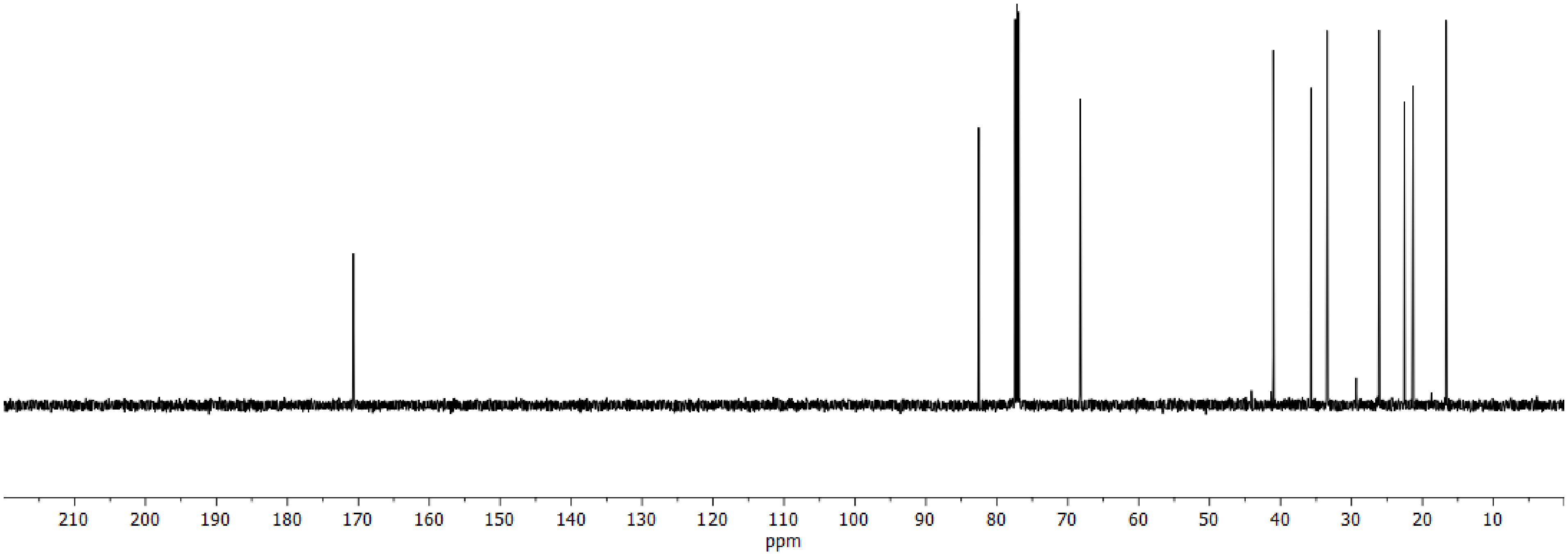

^{1}H NMR of primary alcohol 6b

CDCl3, 500 MHz, 25 ºC

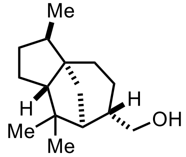

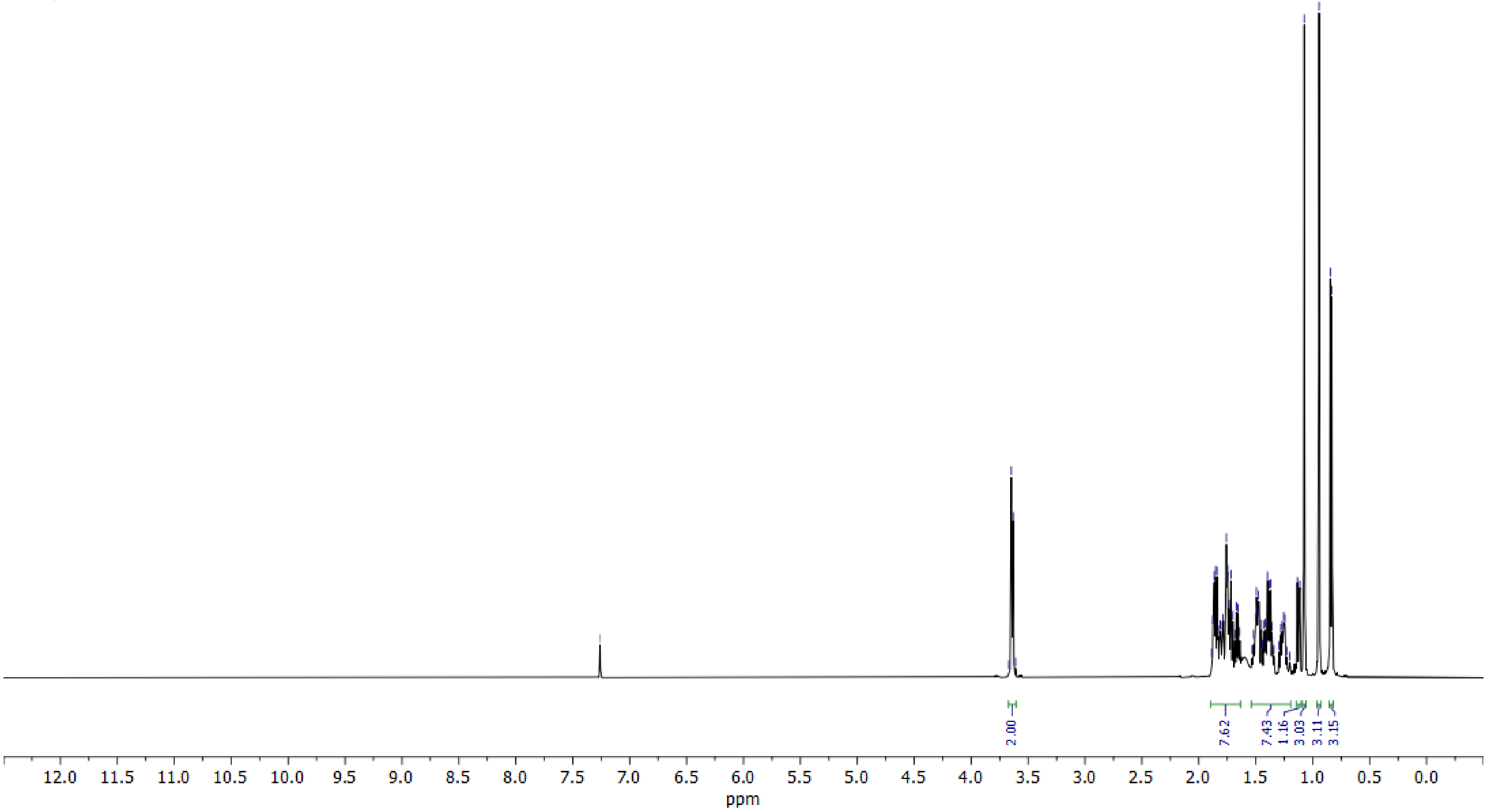

^{13}C NMR of primary alcohol 6b

$CDCl_3$, 125 MHz, 25 ºC

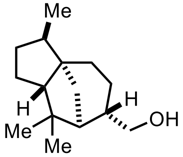

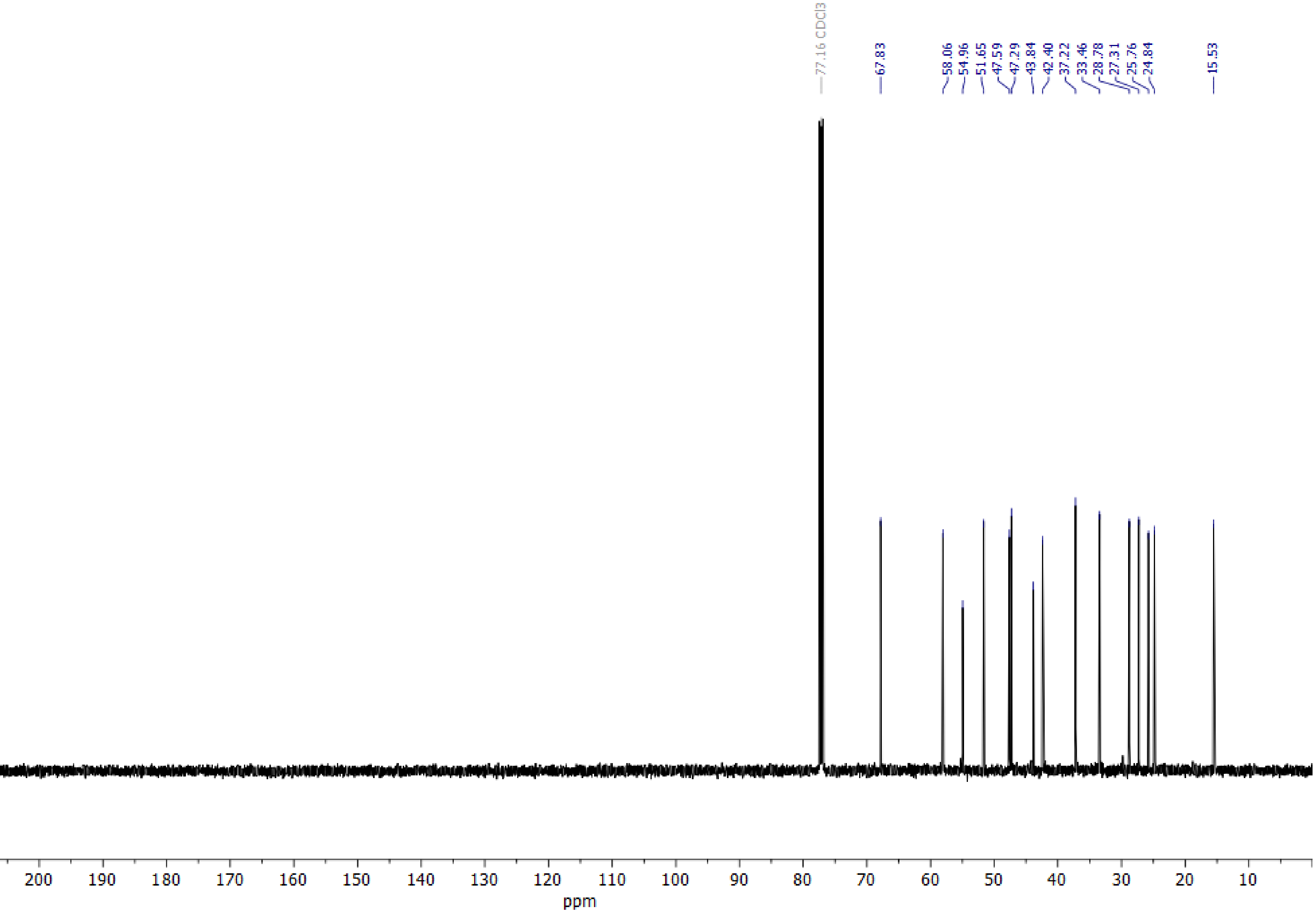

^{1}H NMR of primary alcohol 6c

$CDCl_3$, 500 MHz, 25 ºC

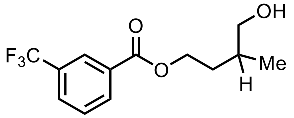

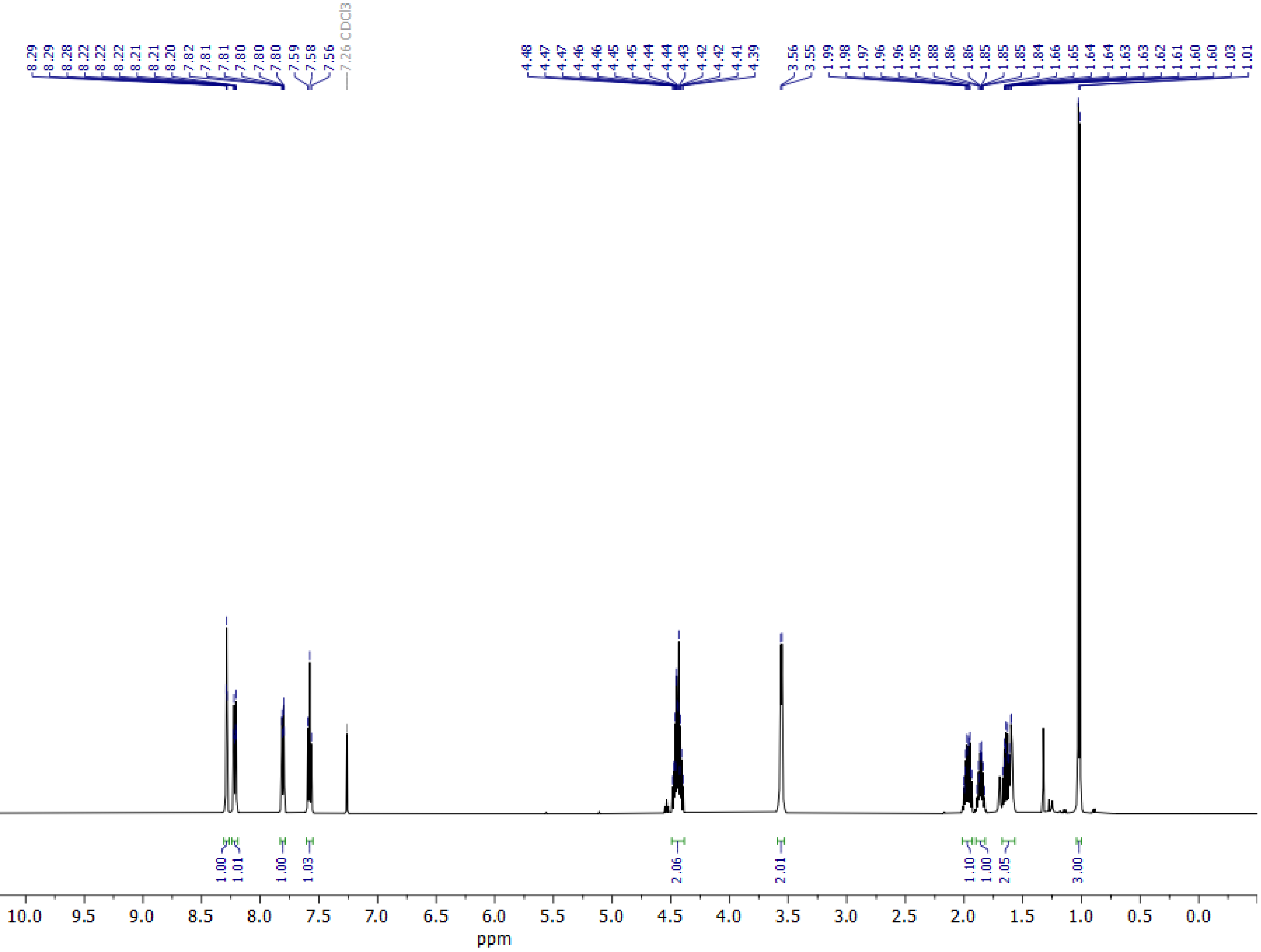

^{13}C NMR of primary alcohol 6c

CDCl₃, 125 MHz, 25 ºC

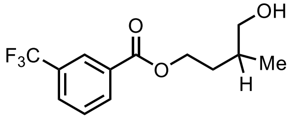

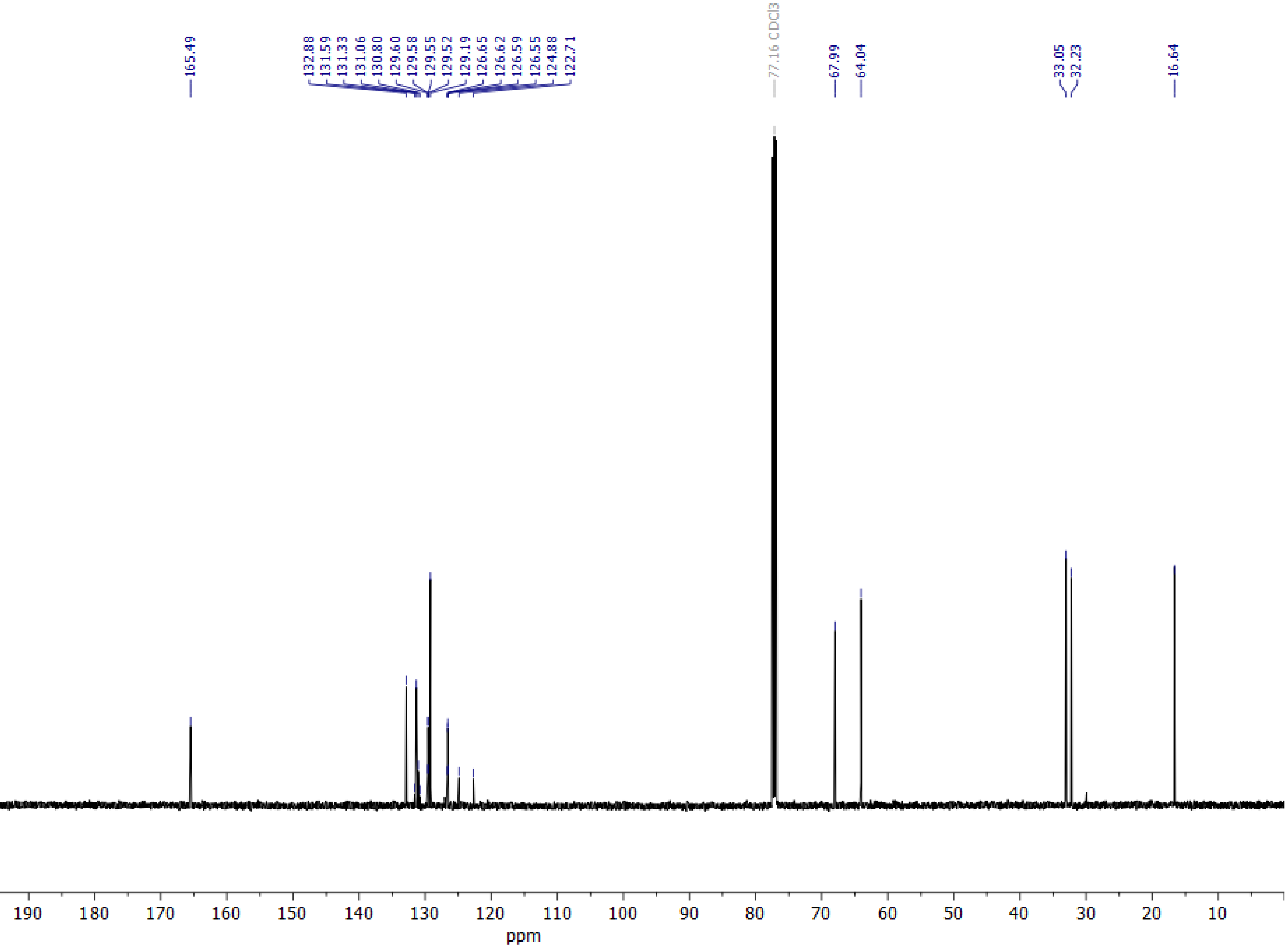

^{19}F NMR of primary alcohol 6c

$CDCl_3$, 470 MHz, 25 ºC

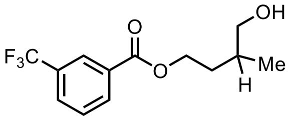

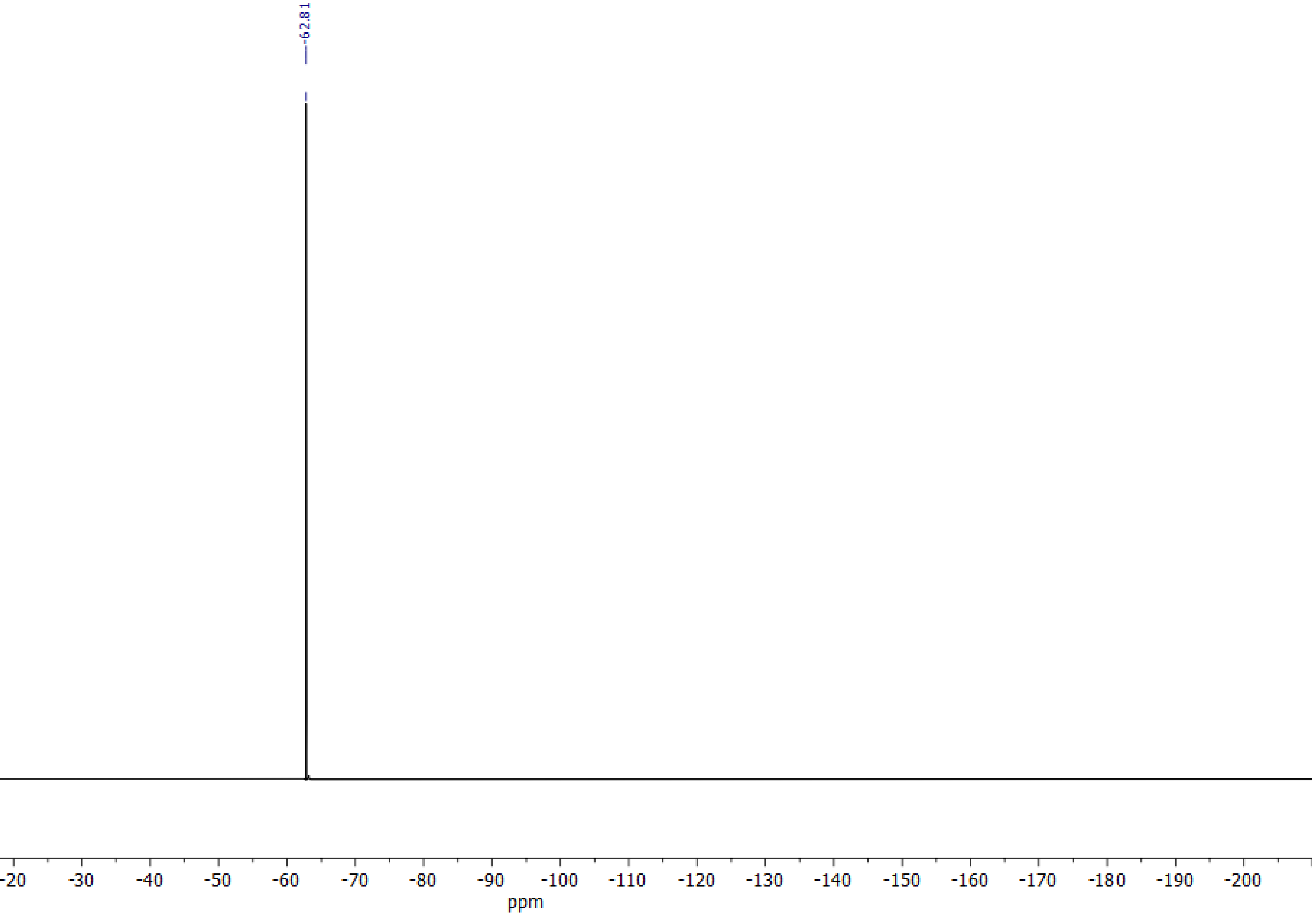

^{1}H NMR of primary alcohol 6d

$CDCl_3$, 500 MHz, 25 ºC

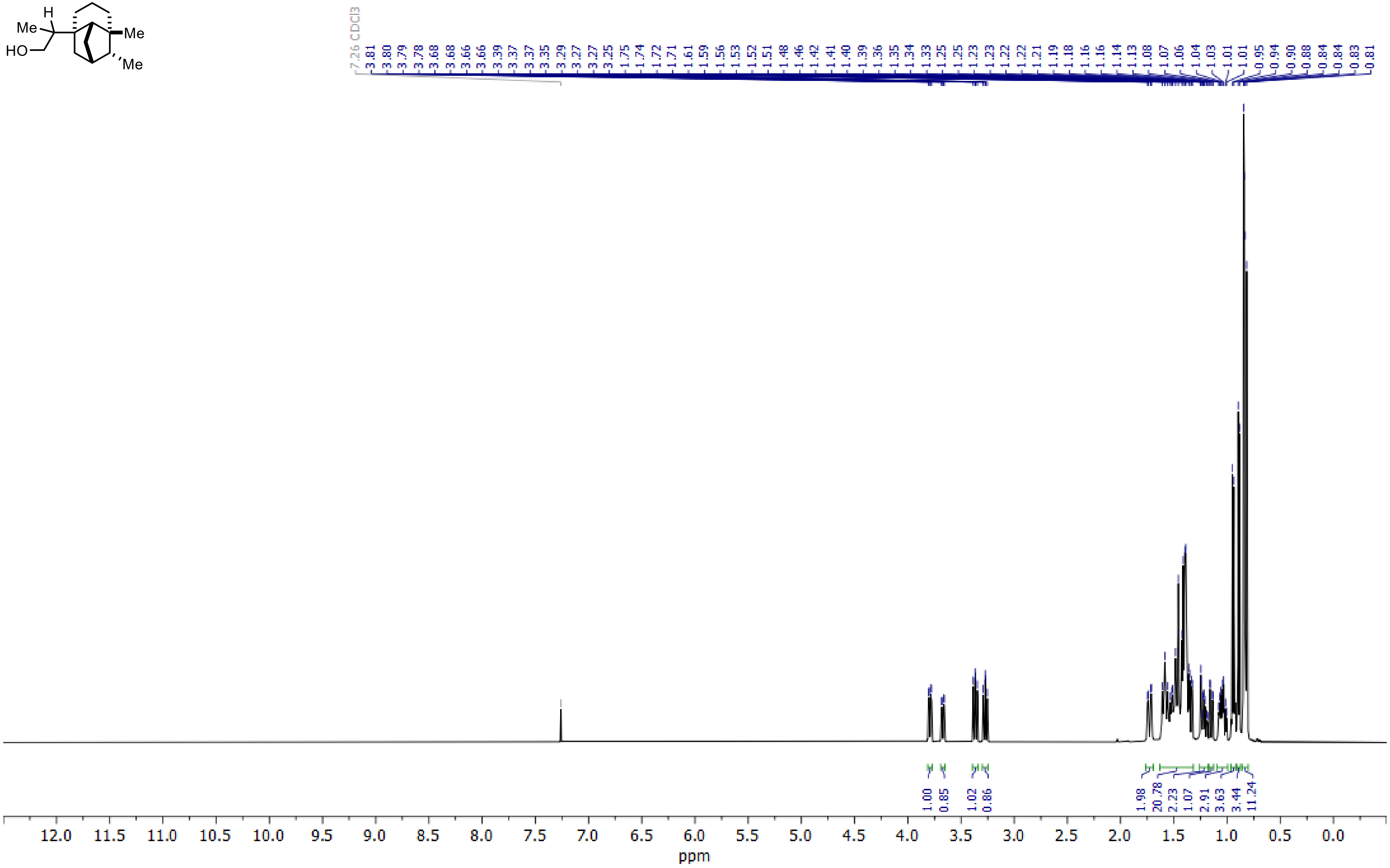

^{13}C NMR of primary alcohol 6d

CDCl$_3$, 125 MHz, 25 ºC

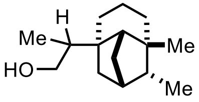

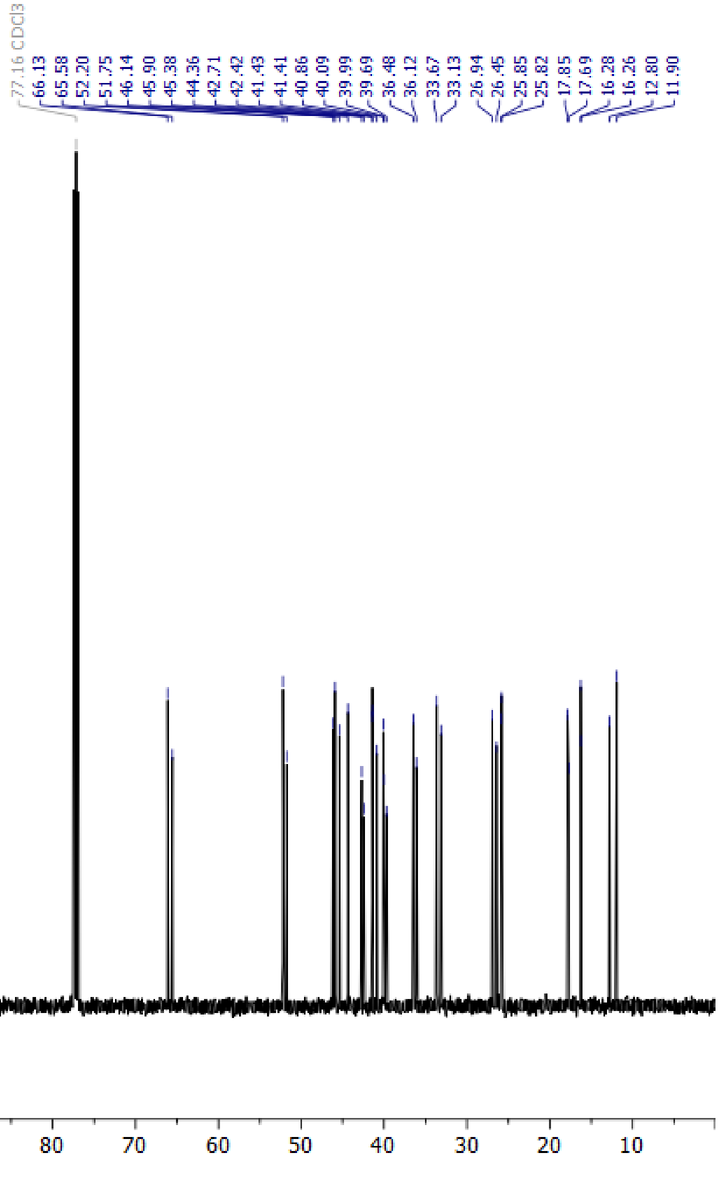

^{1}H NMR of primary alcohol 6e

CDCl3, 500 MHz, 25 ºC

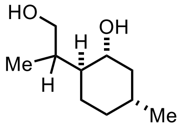

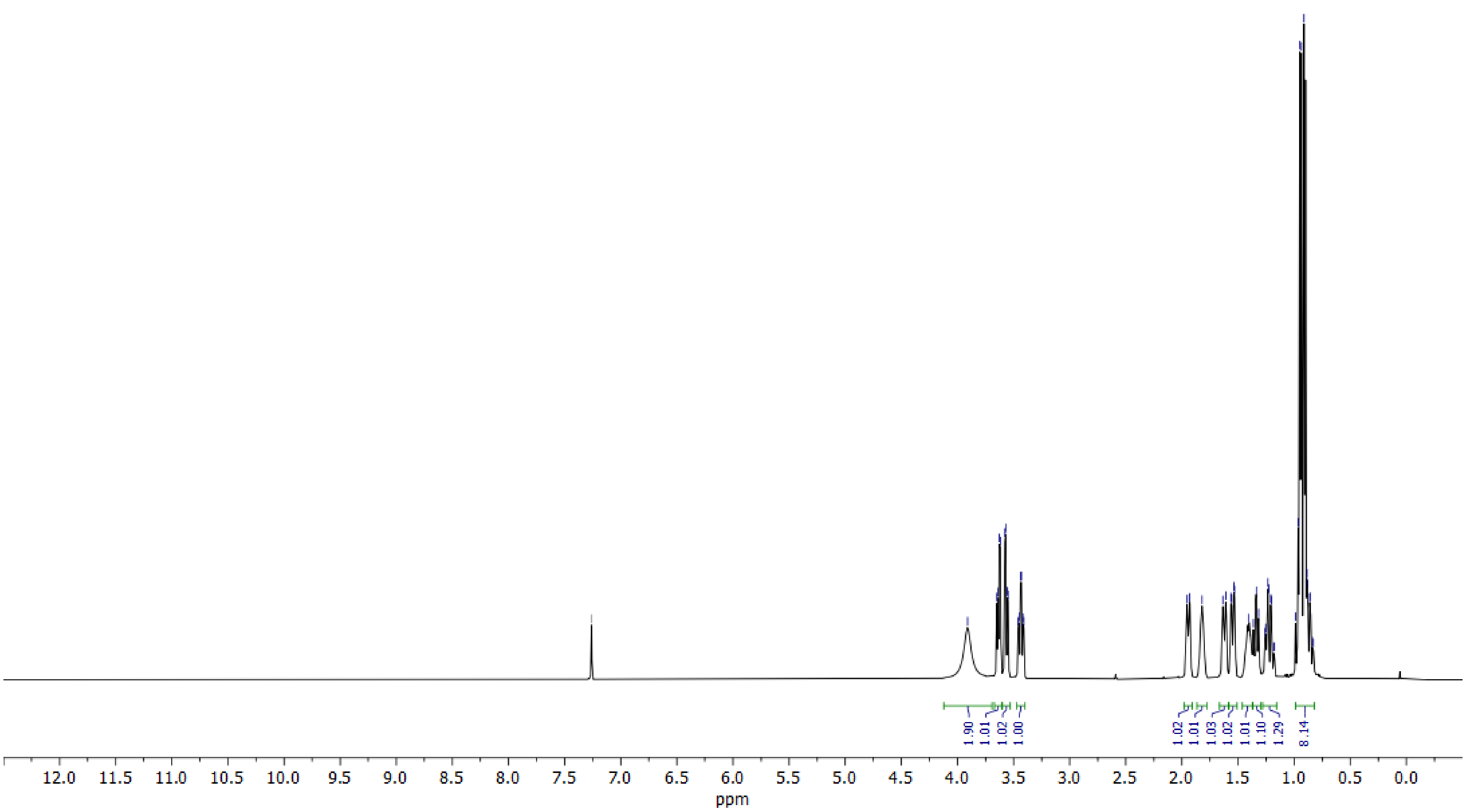

^{13}C NMR of primary alcohol 6e

$CDCl_3$, 125 MHz, 25 ºC

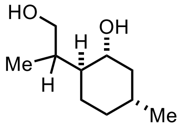

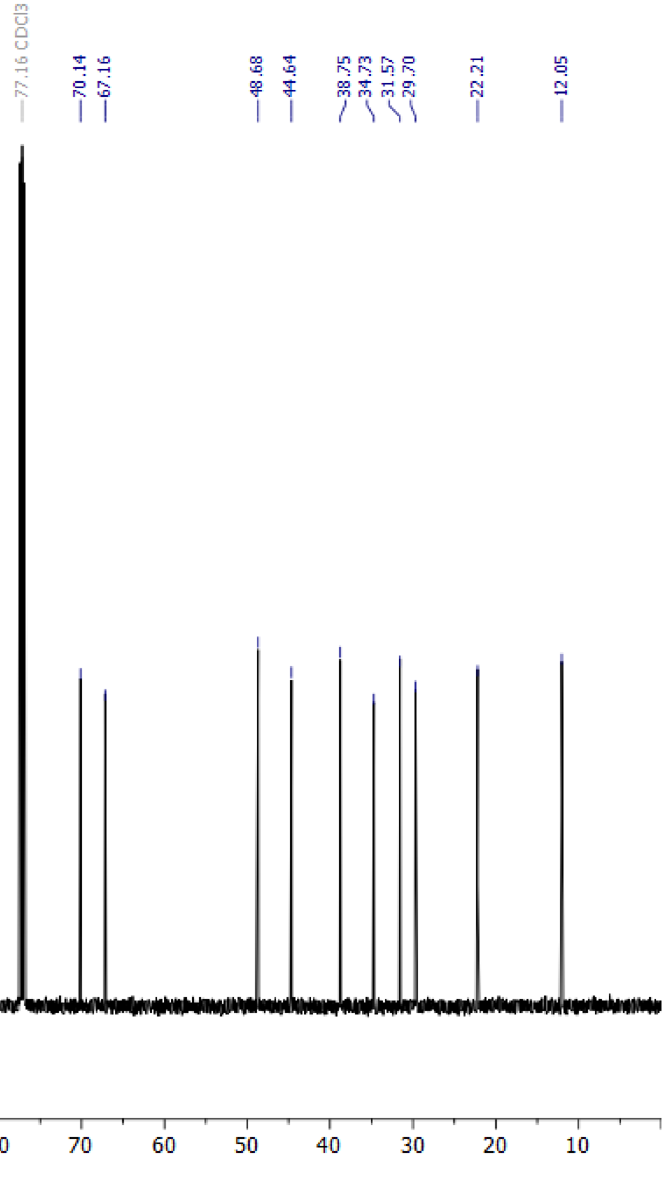

^{1}H NMR of primary alcohol 6f

$CDCl_3$, 500 MHz, 25 ºC

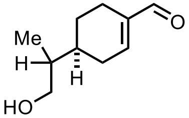

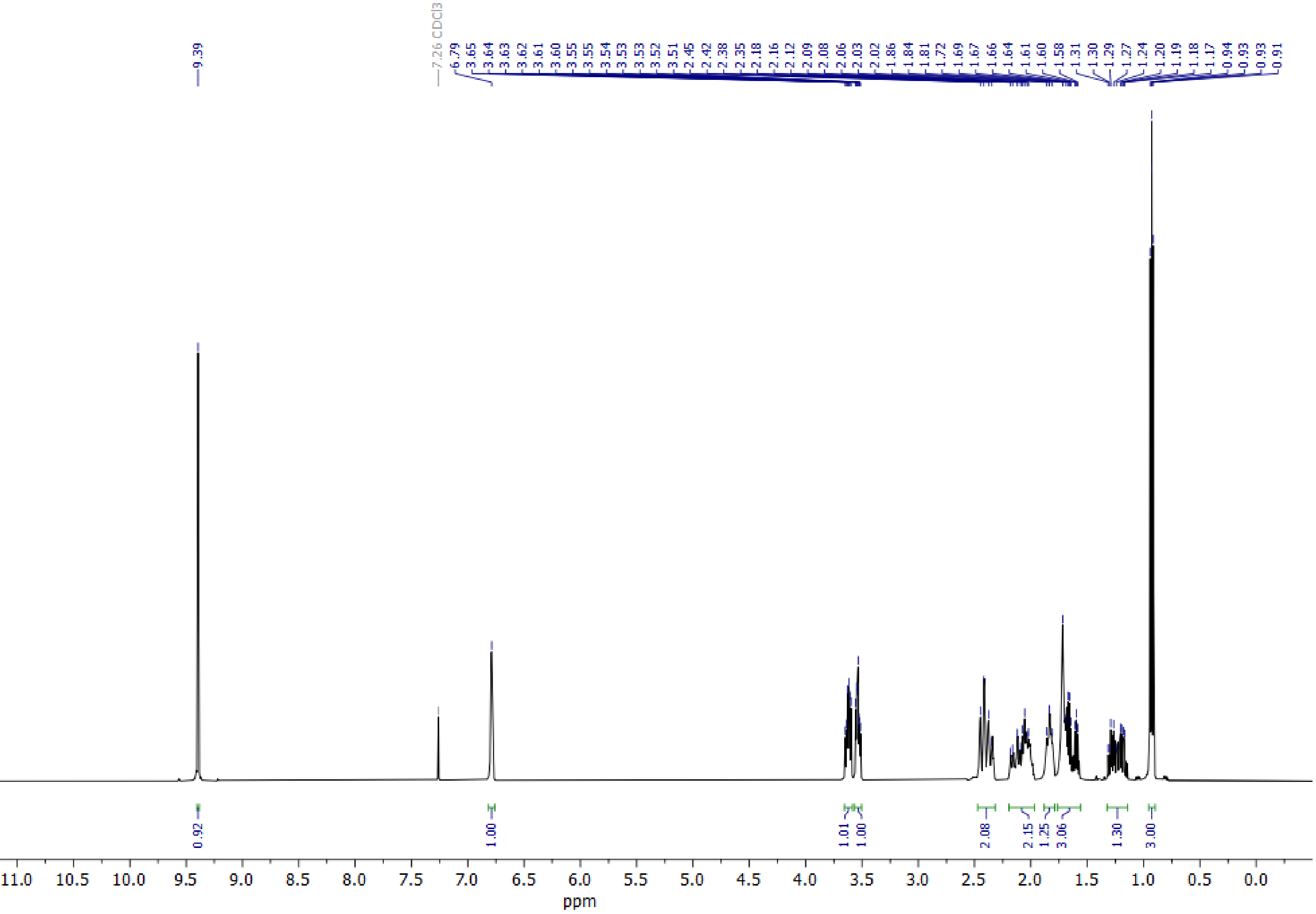

^{13}C NMR of primary alcohol 6f

$CDCl_3$, 125 MHz, 25 ºC

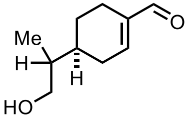

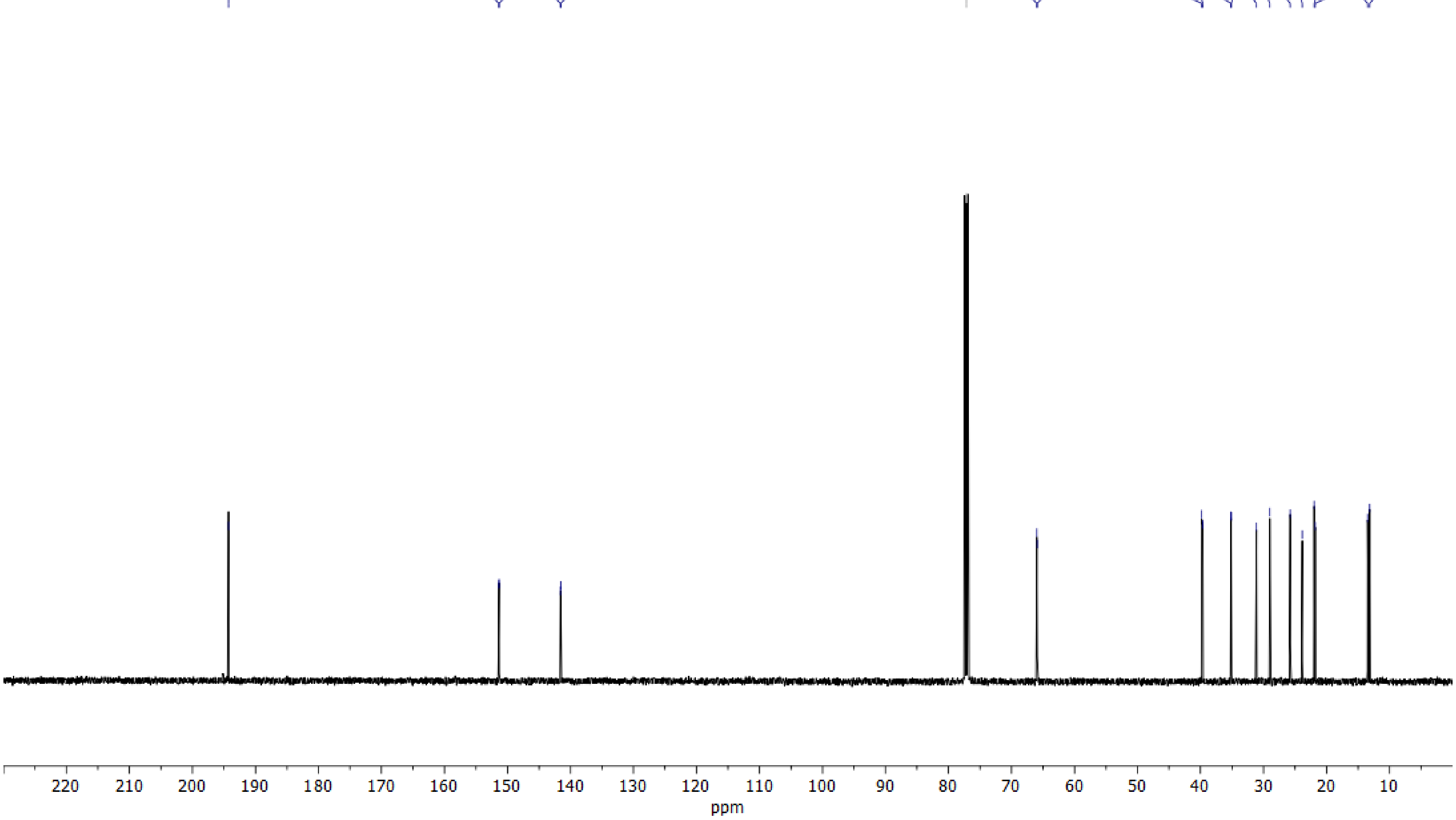

^{1}H NMR of primary alcohol 6g

$CDCl_3$, 500 MHz, 25 ºC

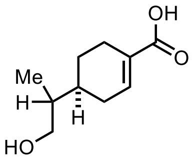

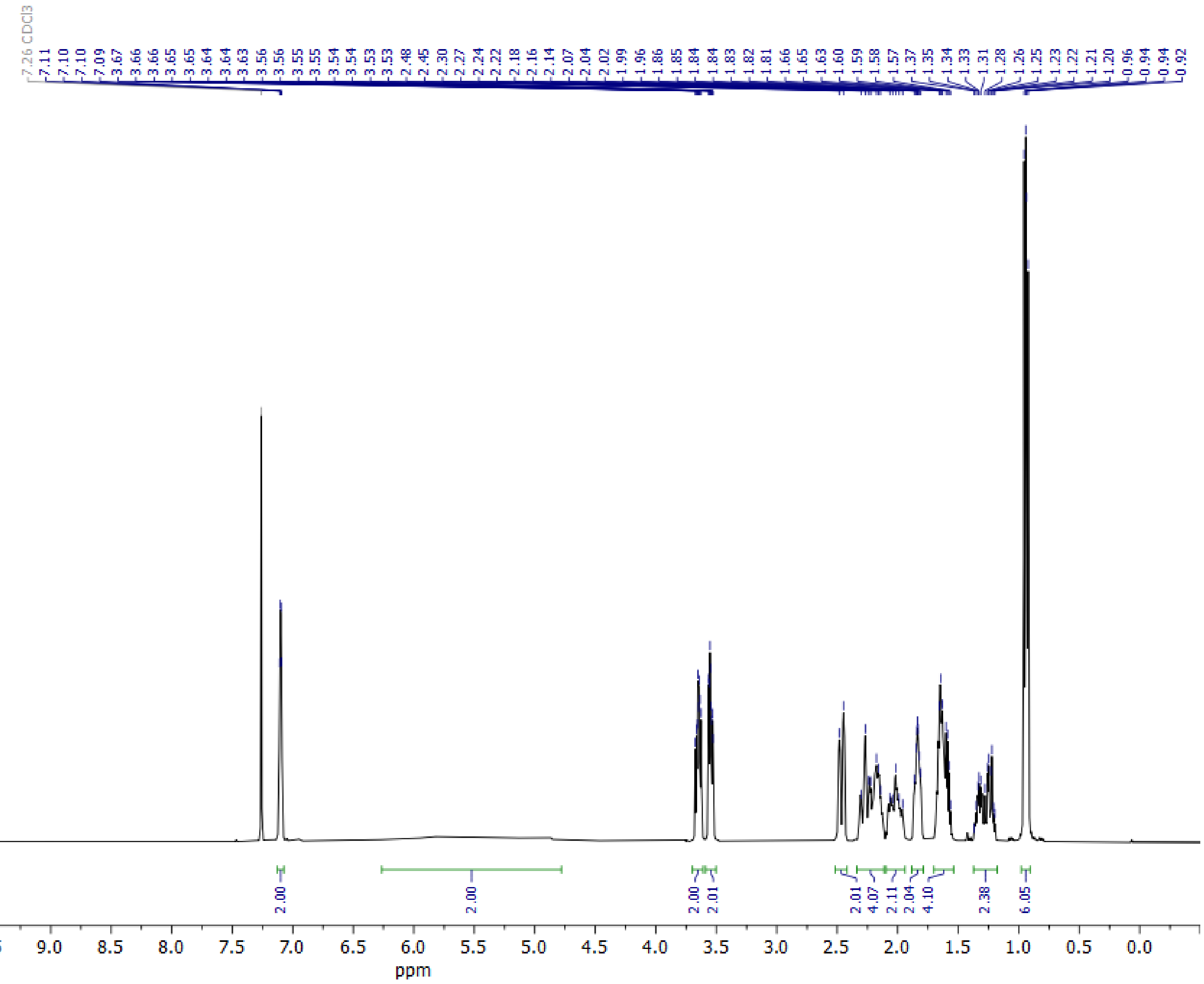

^{13}C NMR of primary alcohol 6g

$CDCl_3$, 125 MHz, 25 ºC

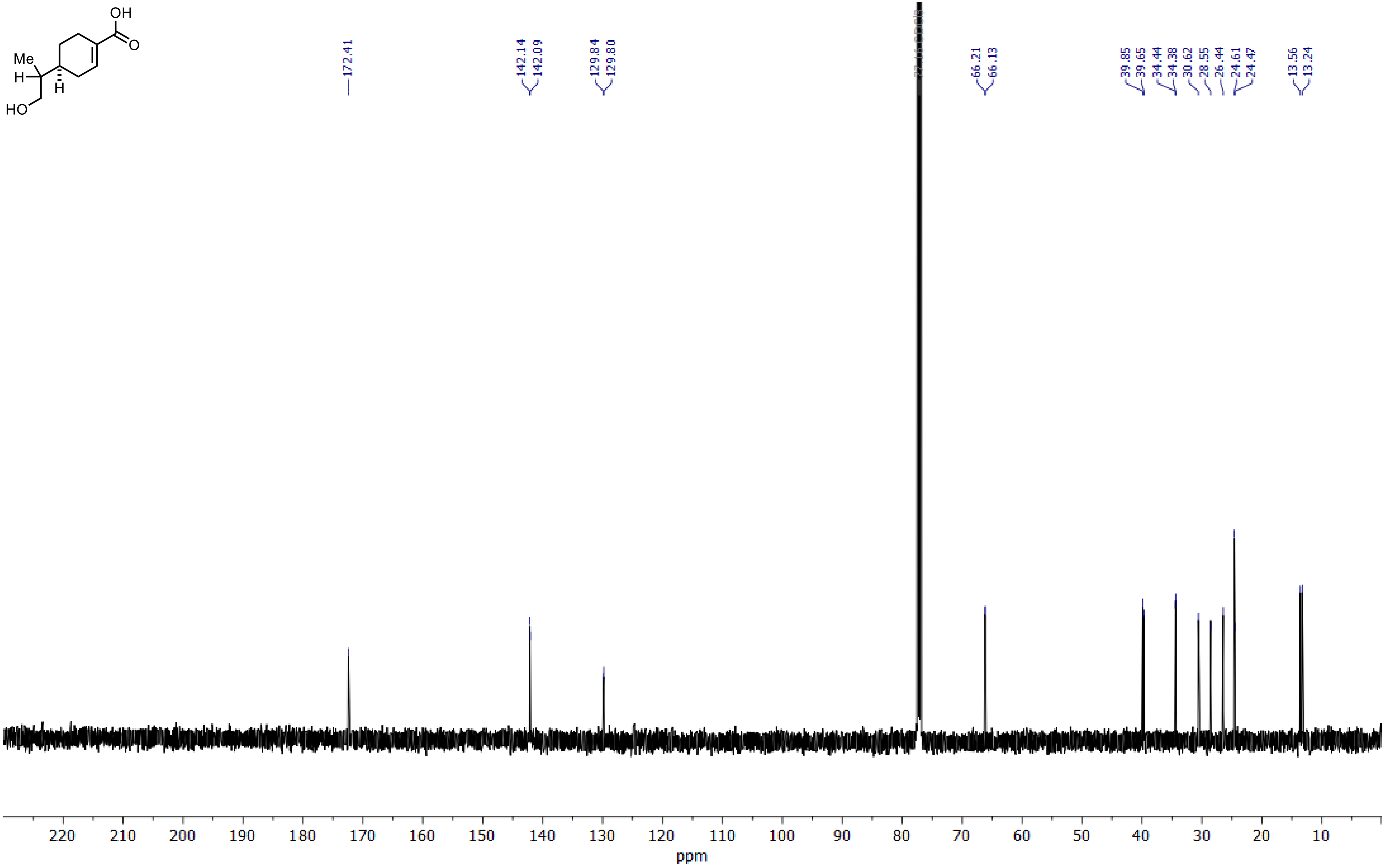

^{1}H NMR of primary alcohol 6h

$CDCl_3$, 500 MHz, 25 ºC

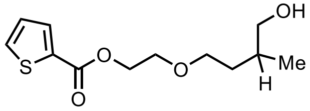

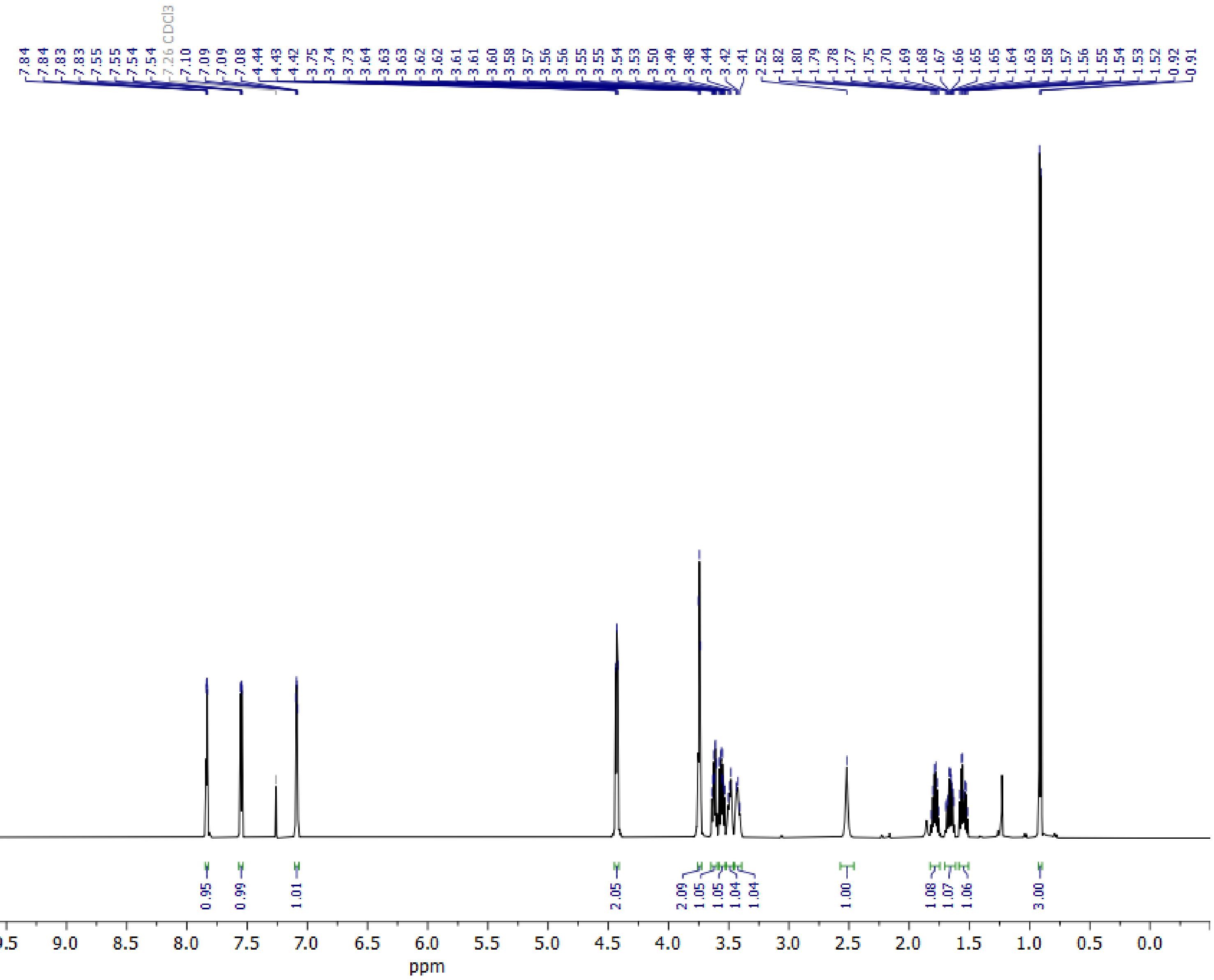

^{13}C NMR of primary alcohol 6h

$CDCl_3$, 125 MHz, 25 ºC

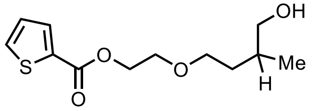

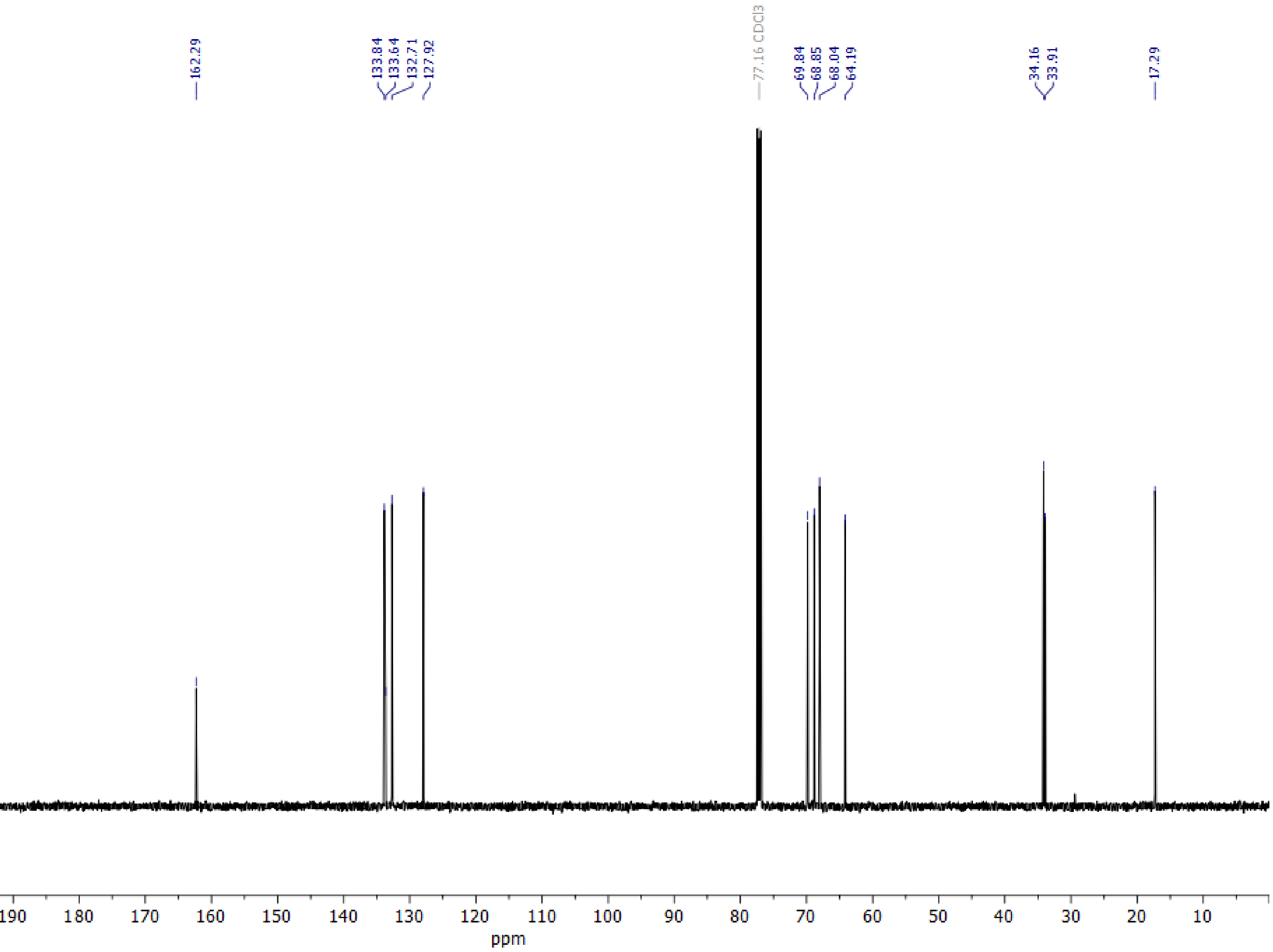

^{1}H NMR of primary alcohol 6i

$CDCl_3$, 500 MHz, 25 ºC

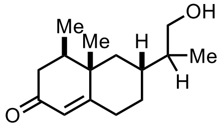

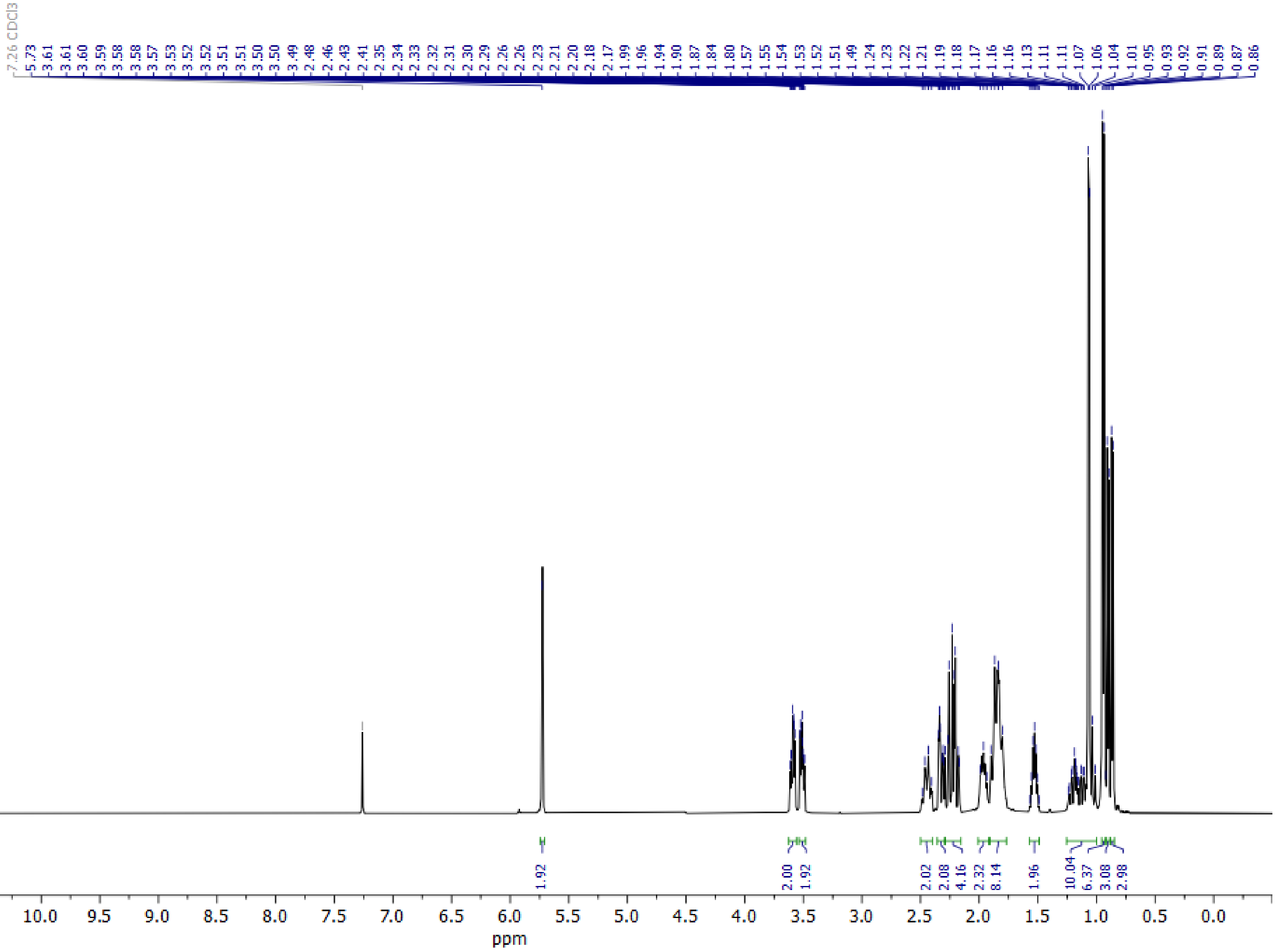

^{13}C NMR of primary alcohol 6i

$CDCl_3$, 125 MHz, 25 ºC

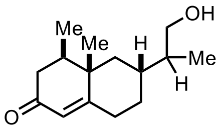

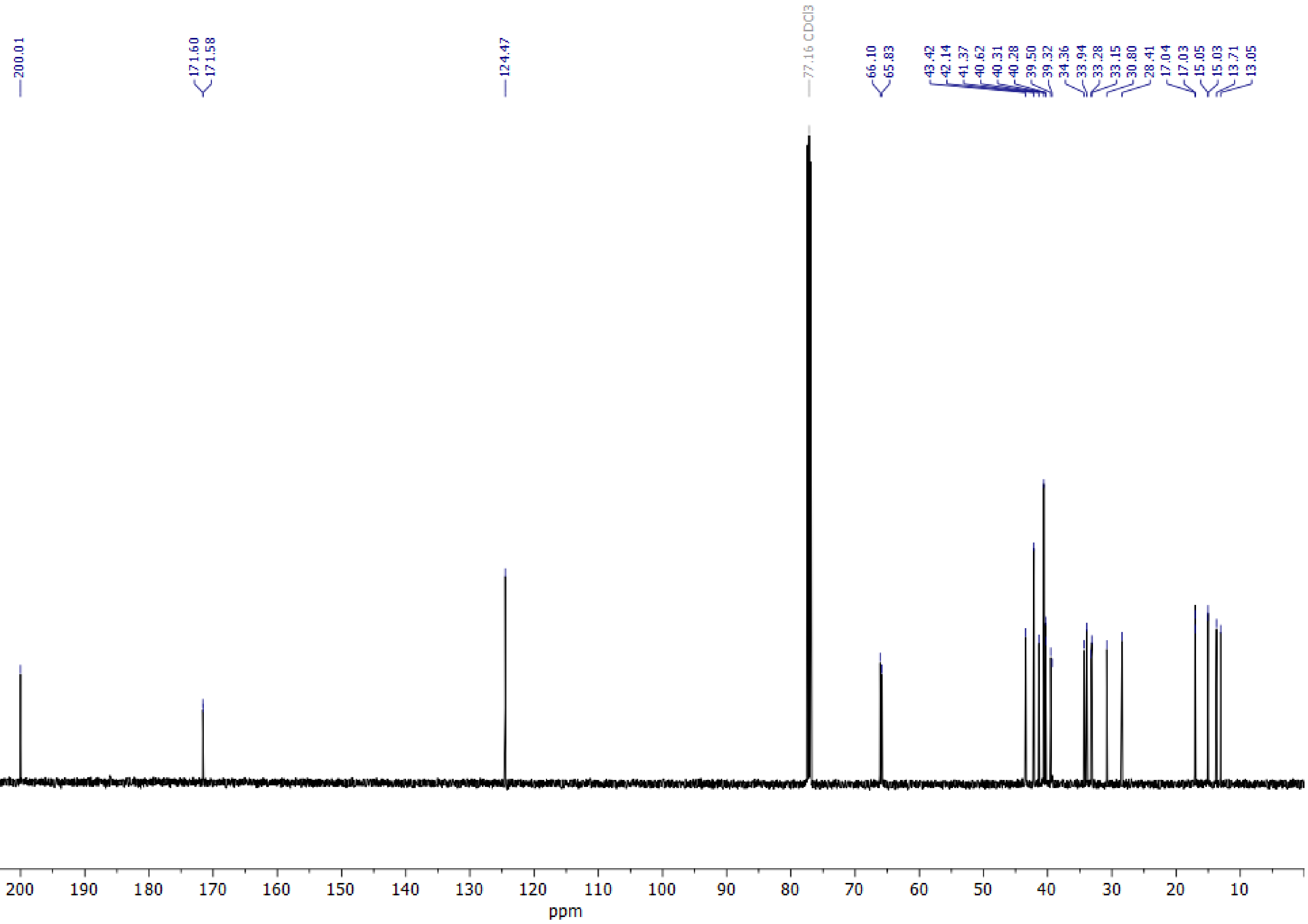

^{1}H NMR of primary alcohol 6j

CDCl$_3$, 500 MHz, 25 ºC

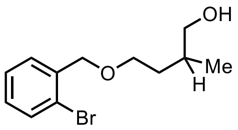

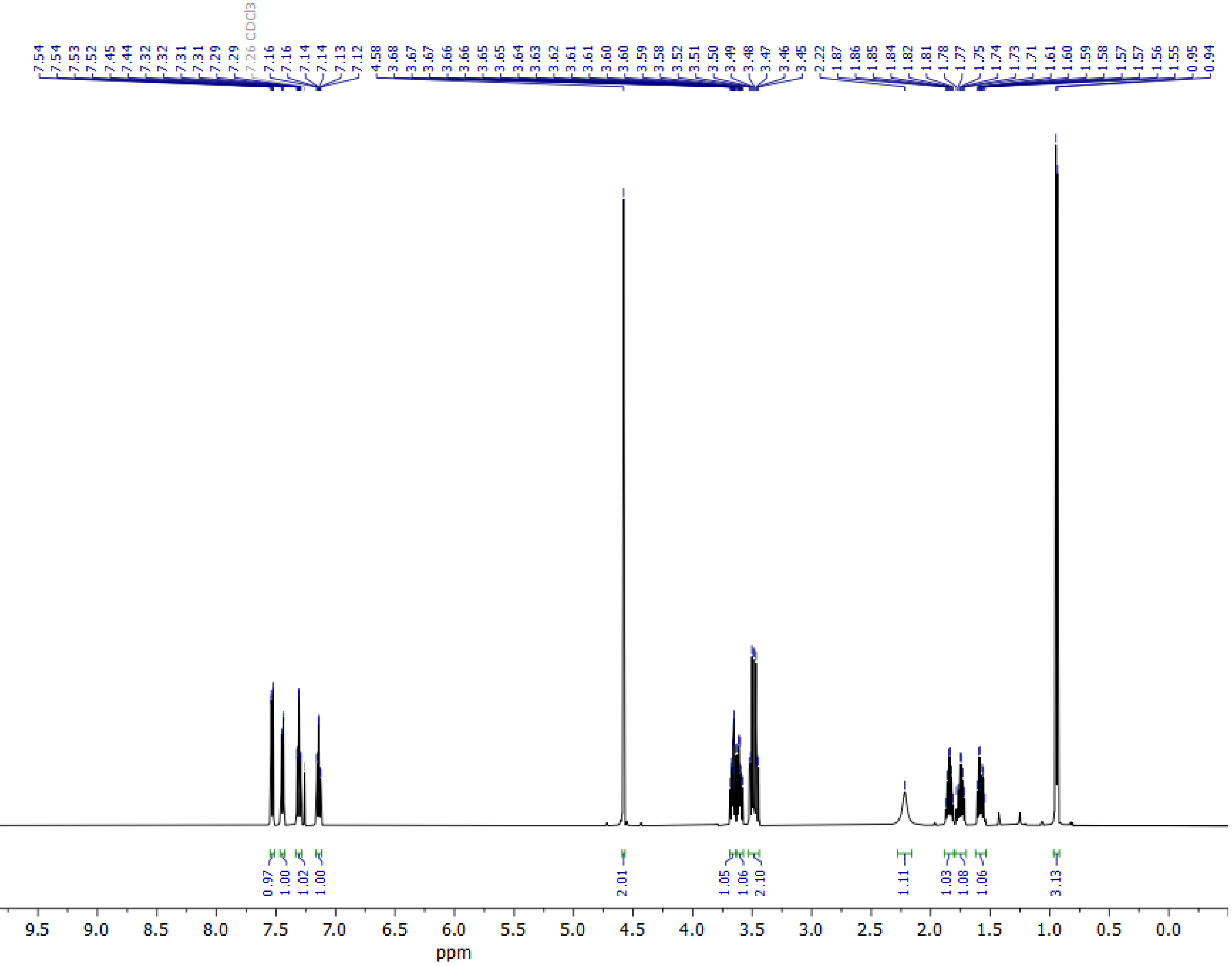

^{13}C NMR of primary alcohol 6j

$CDCl_3$, 125 MHz, 25 ºC

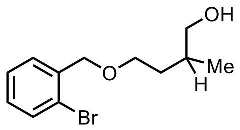

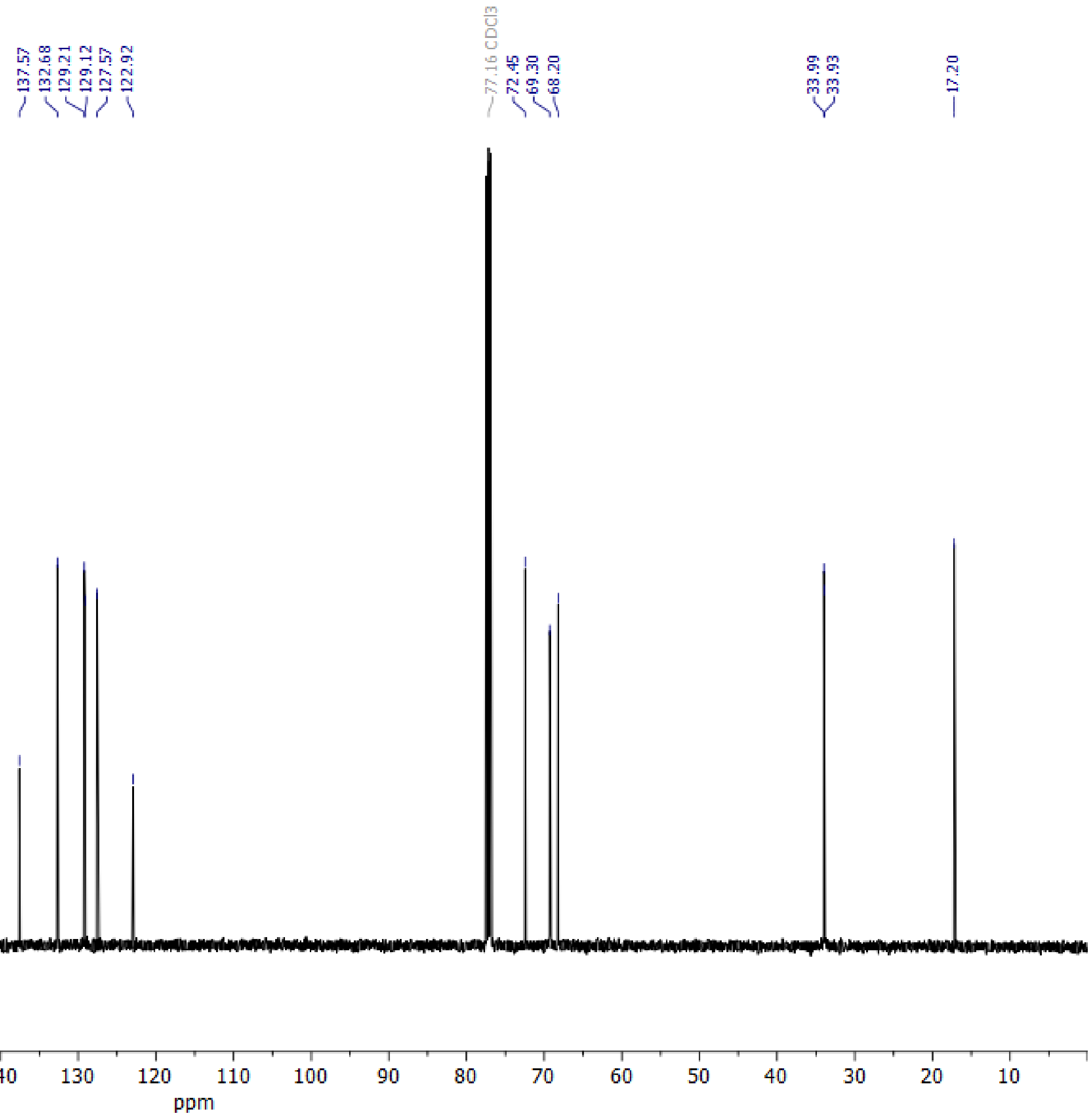

^{1}H NMR of primary alcohol 6k

$CDCl_3$, 500 MHz, 25 ºC

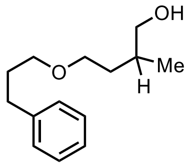

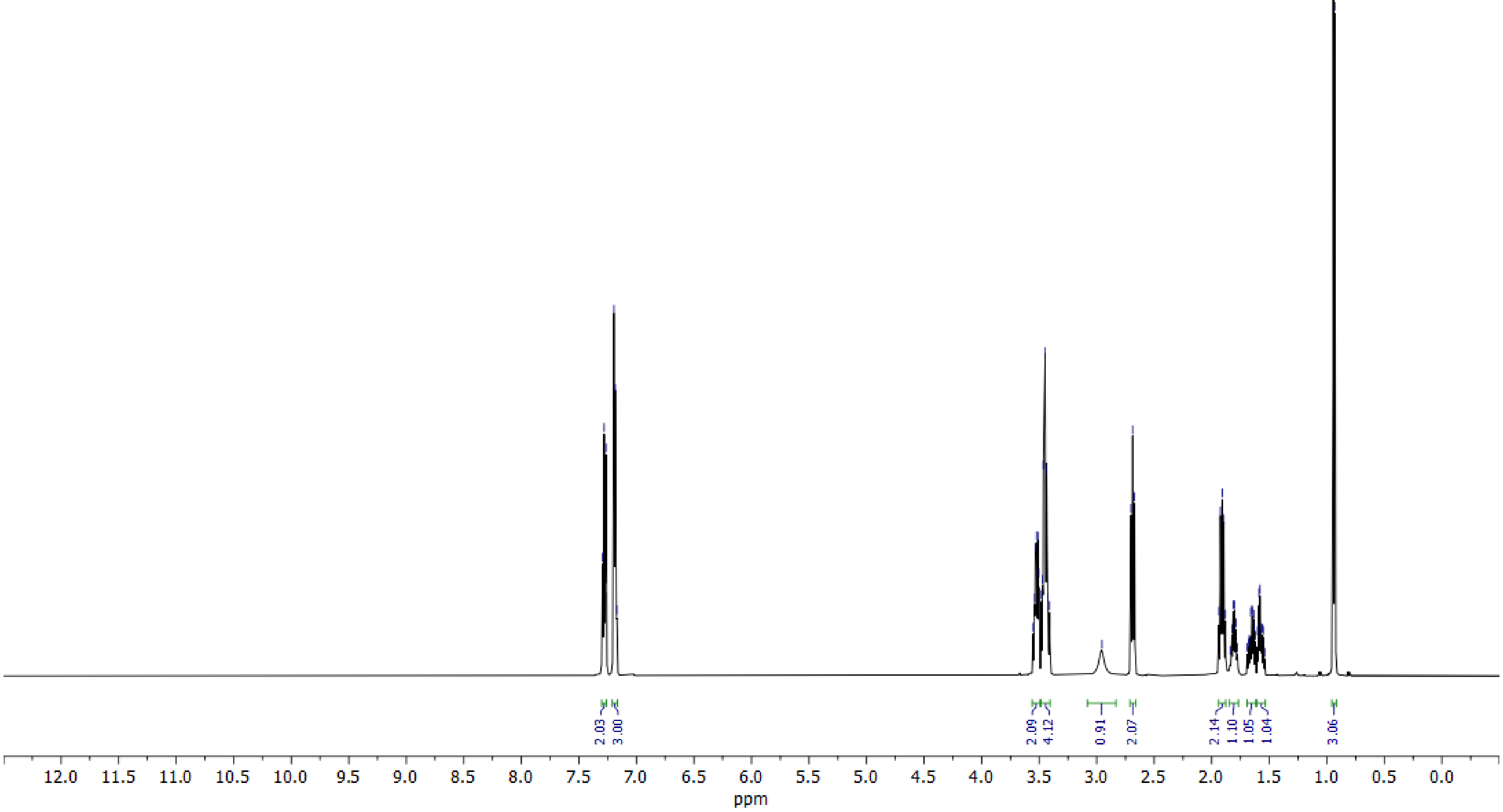

^{13}C NMR of primary alcohol 6k

$CDCl_3$, 125 MHz, 25 ºC

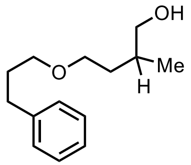

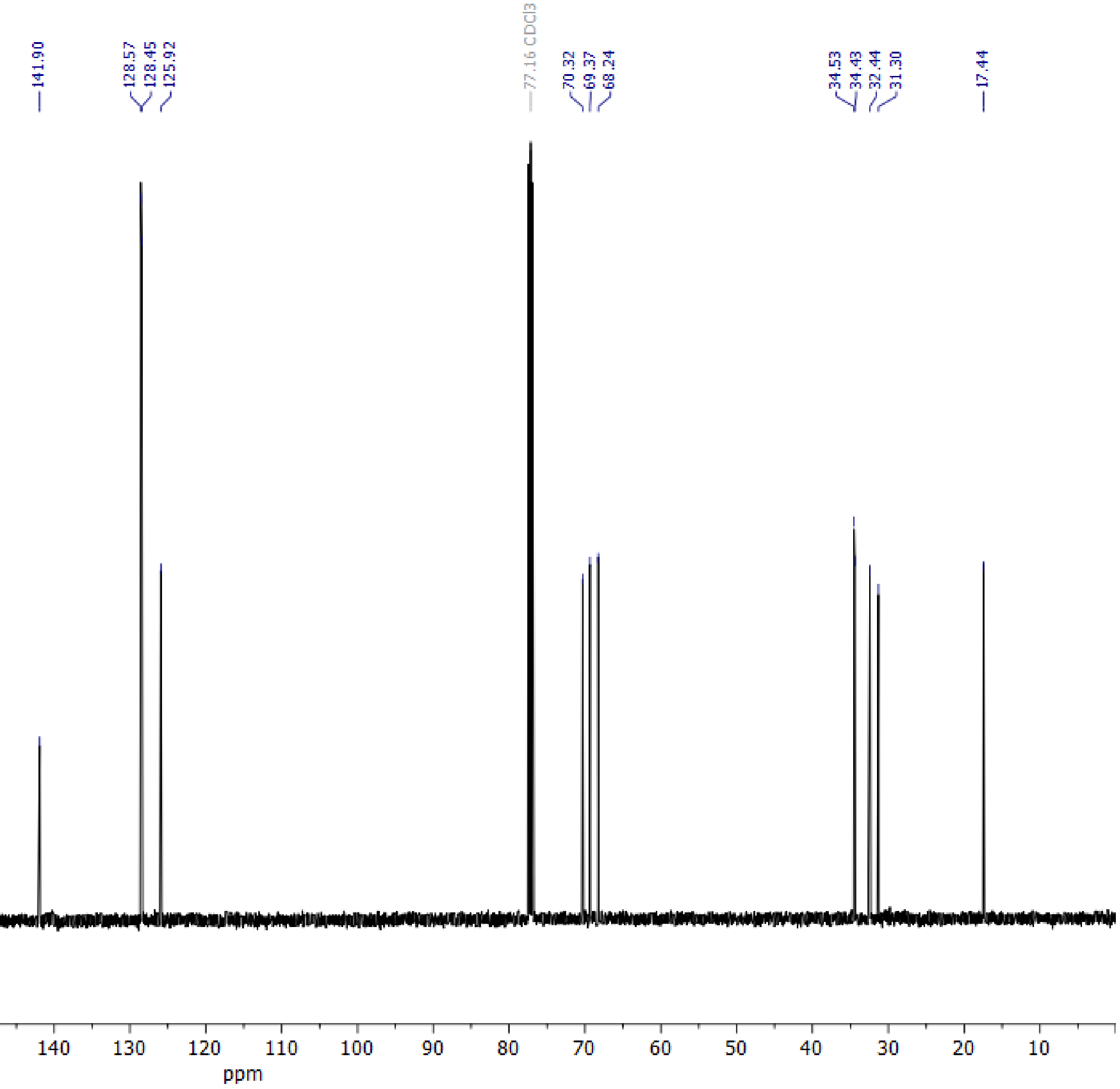

¹H NMR of secondary alcohol 6l

$CDCl_3$, 500 MHz, 25 ºC

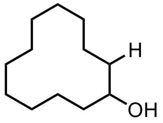

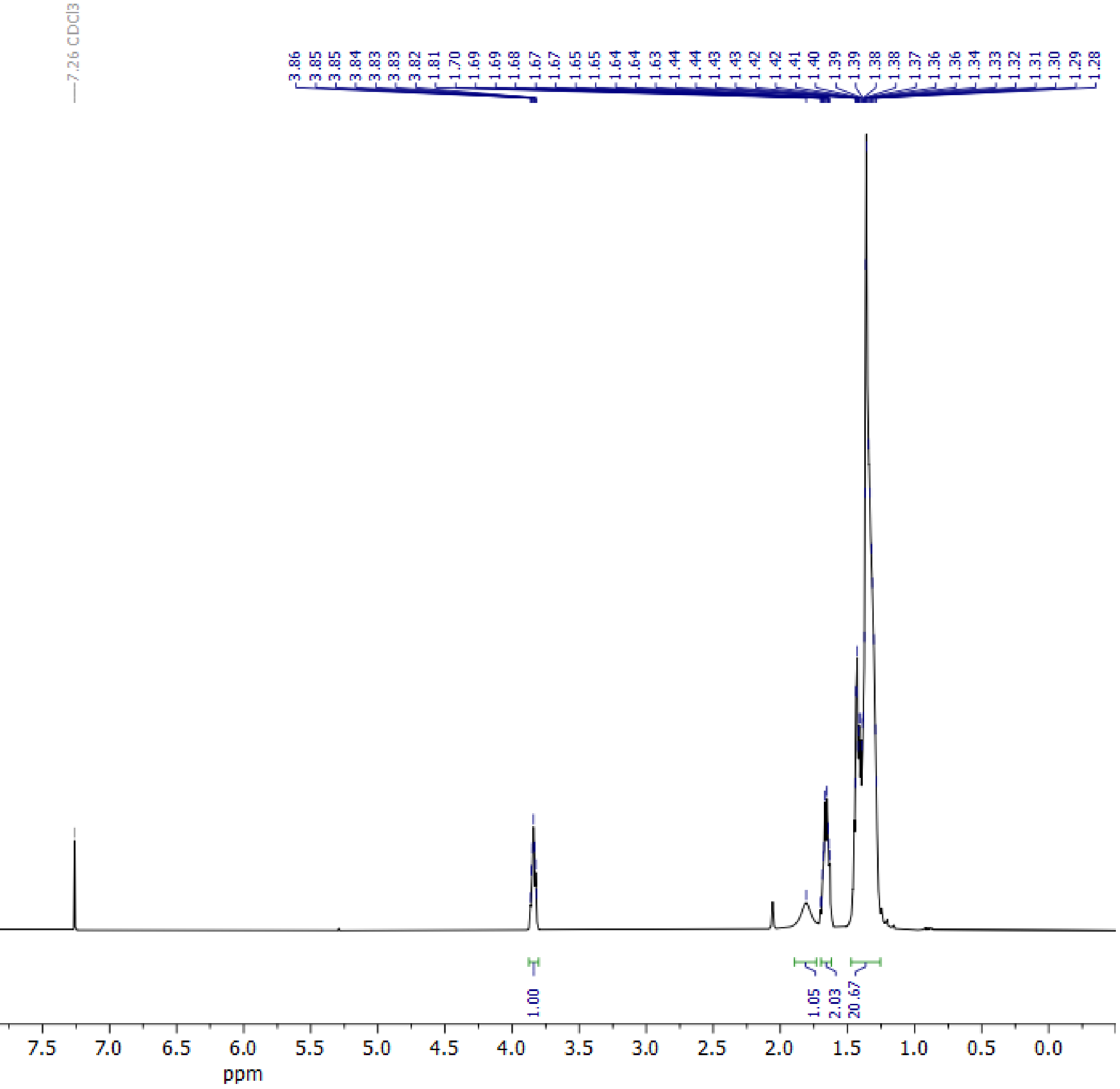

^{13}C NMR of secondary alcohol 6l

$CDCl_3$, 125 MHz, 25 ºC

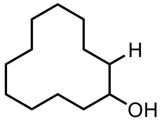

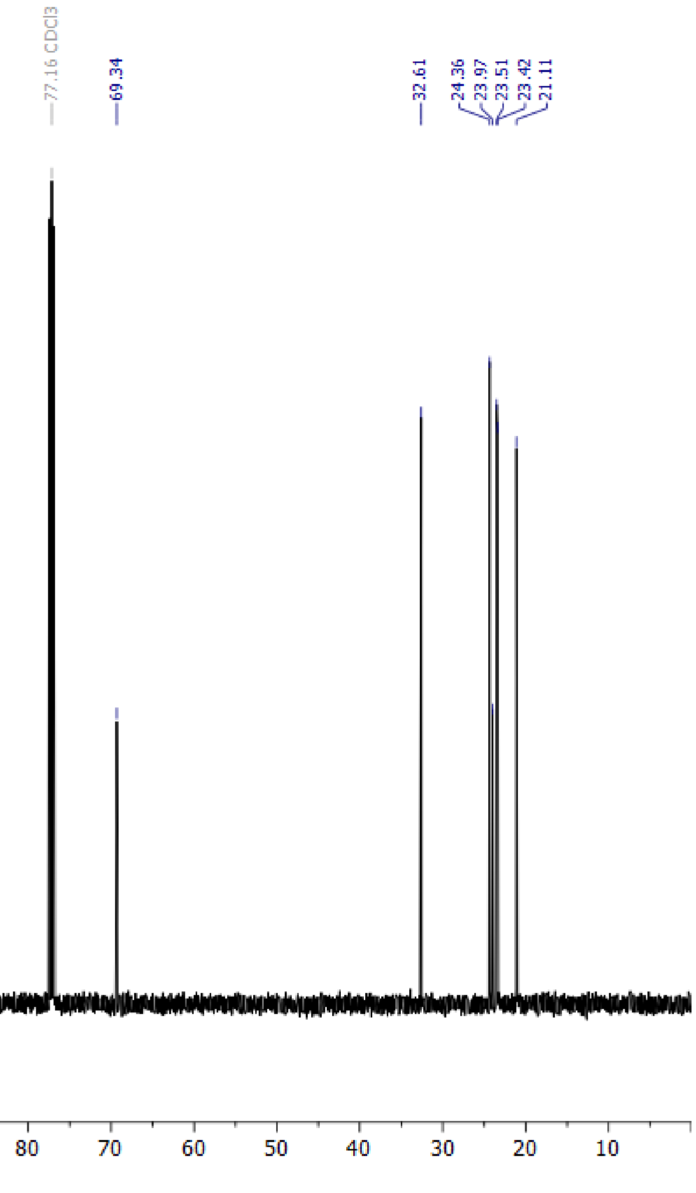

^{1}H NMR of primary alcohol 6m

CDCl$_3$, 500 MHz, 25 ºC

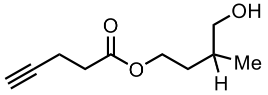

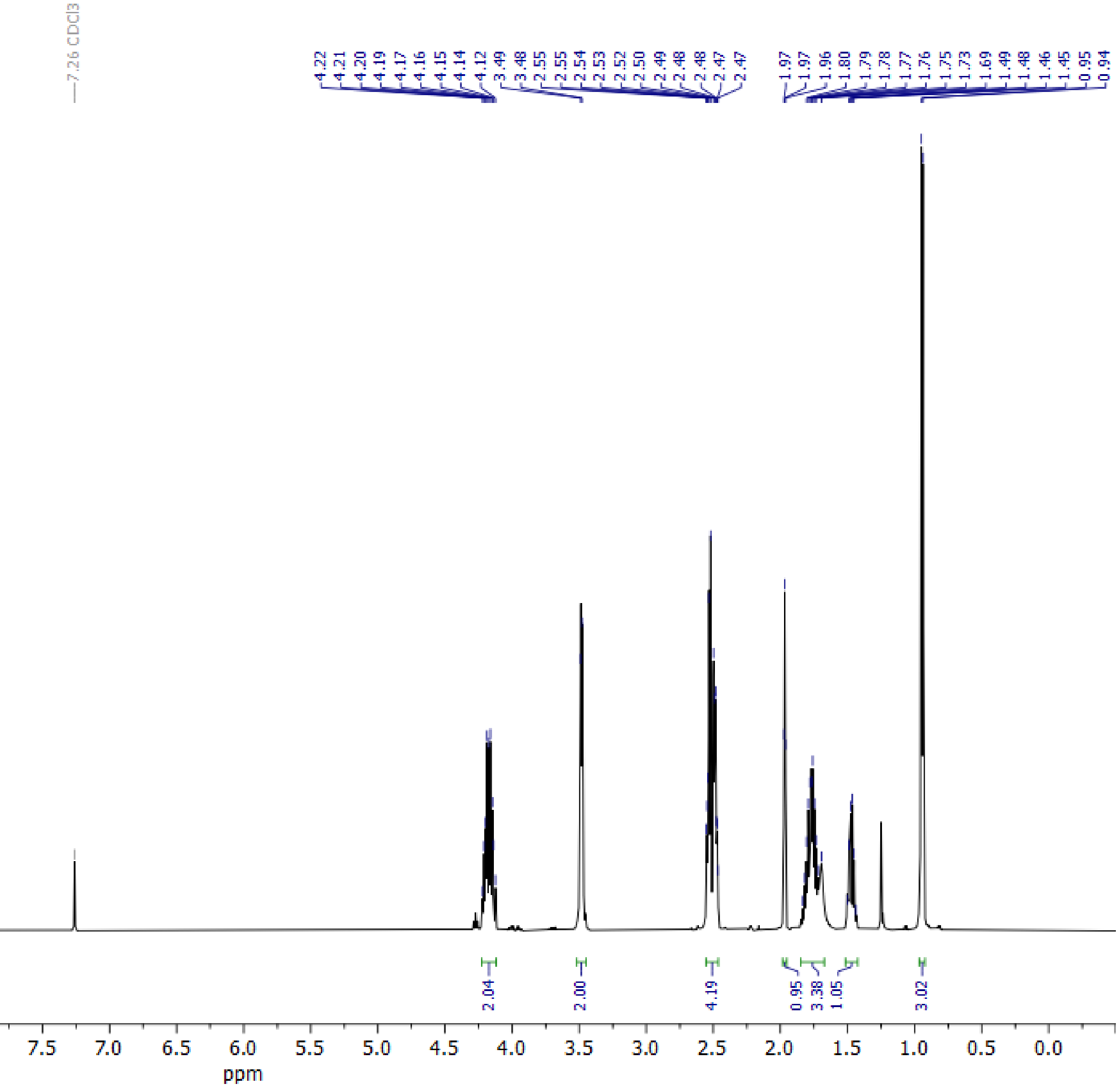

^{13}C NMR of primary alcohol 6m

$CDCl_3$, 125 MHz, 25 ºC

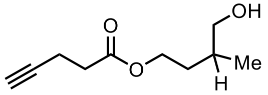

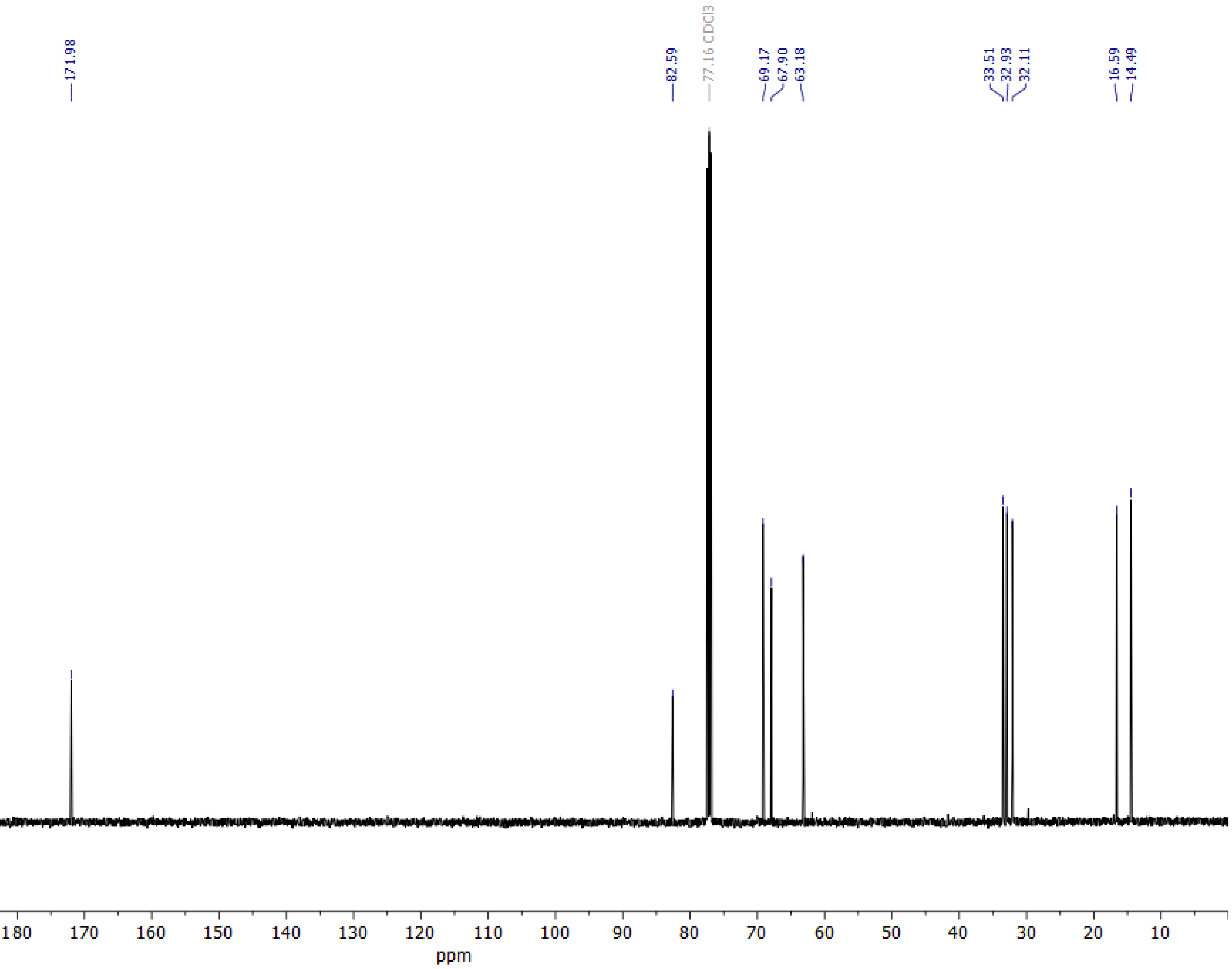

^{1}H NMR of primary alcohol 6n (diastereoisomer 1)

$CDCl_3$, 500 MHz, 25 ºC

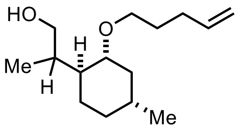

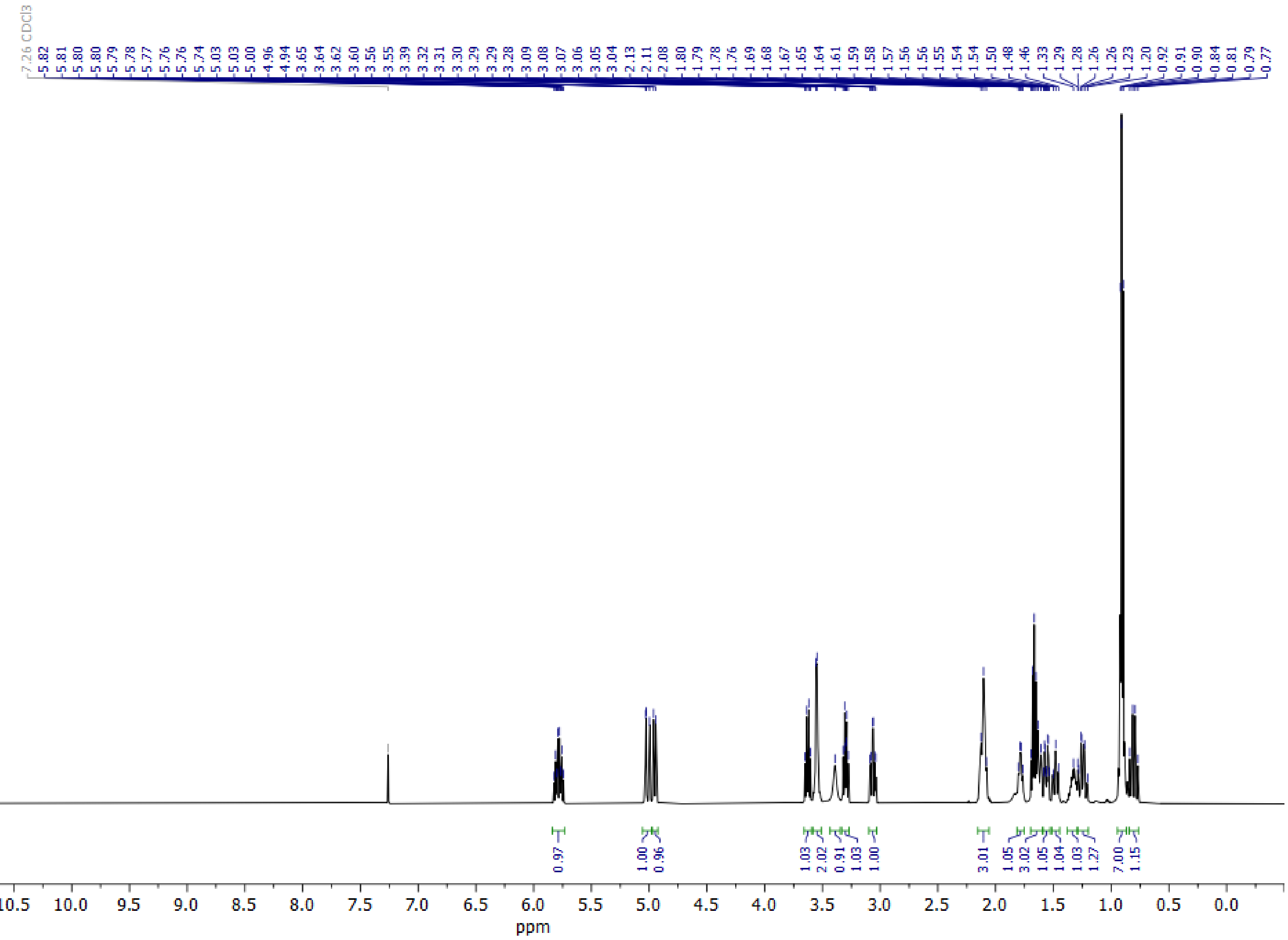

^{13}C NMR of primary alcohol 6n (diastereoisomer 1)

$CDCl_3$, 125 MHz, 25 ºC

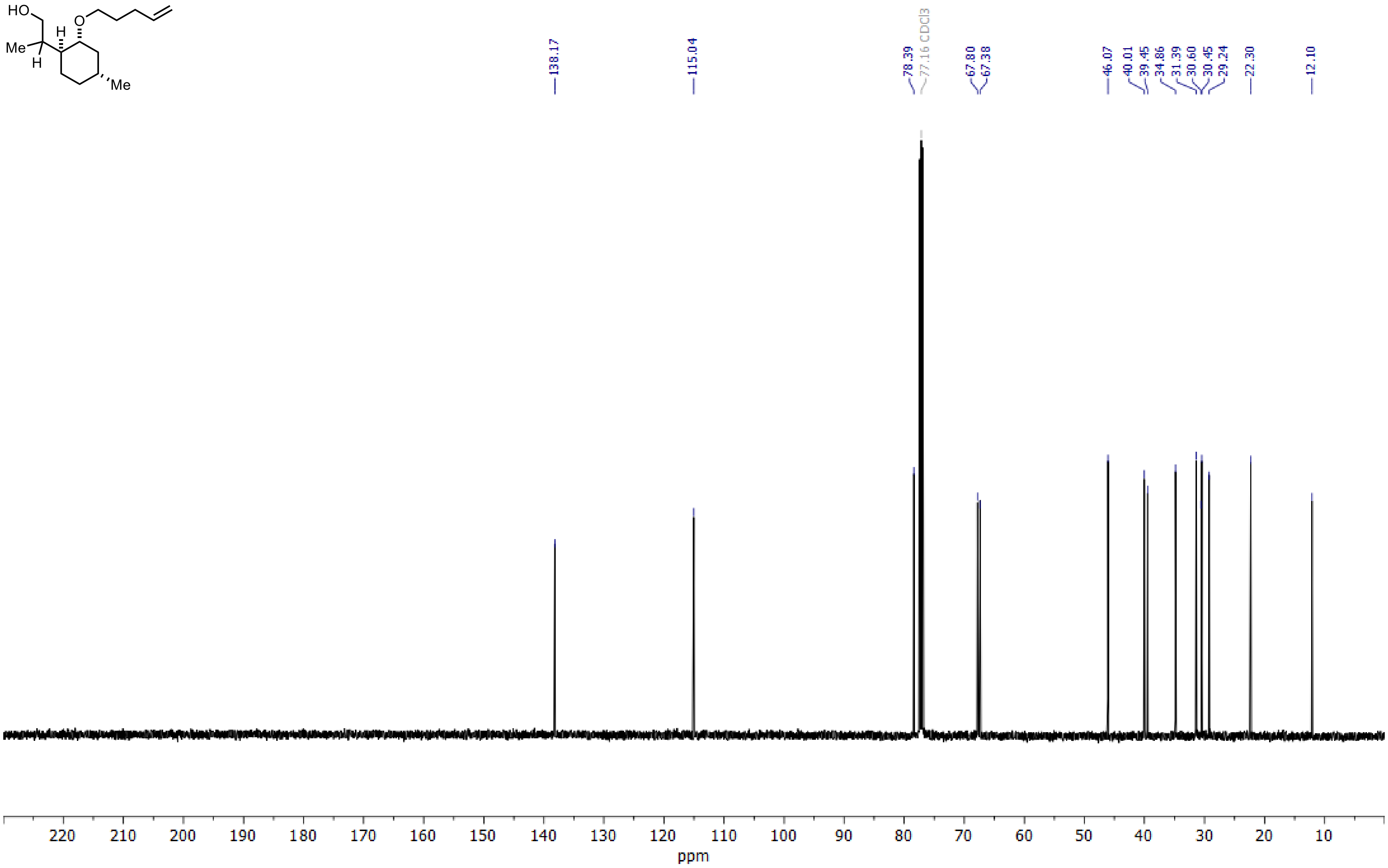

^{1}H NMR of primary alcohol 6n (diastereoisomer 2)

$CDCl_3$, 500 MHz, 25 ºC

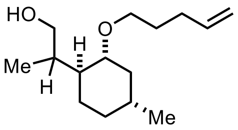

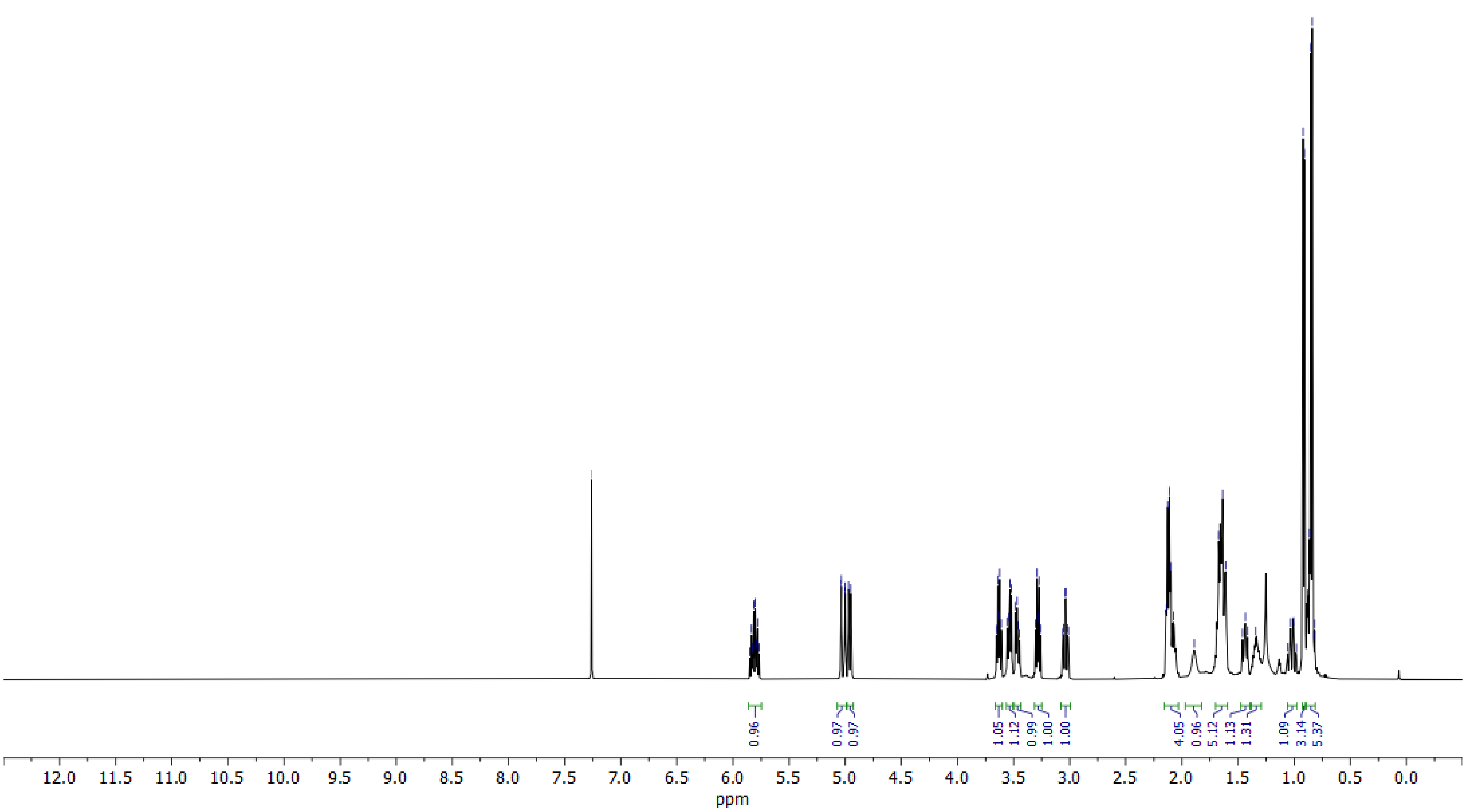

^{13}C NMR of primary alcohol 6n (diastereoisomer 2)

$CDCl_3$, 125 MHz, 25 ºC

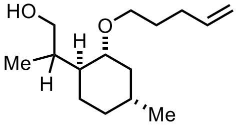

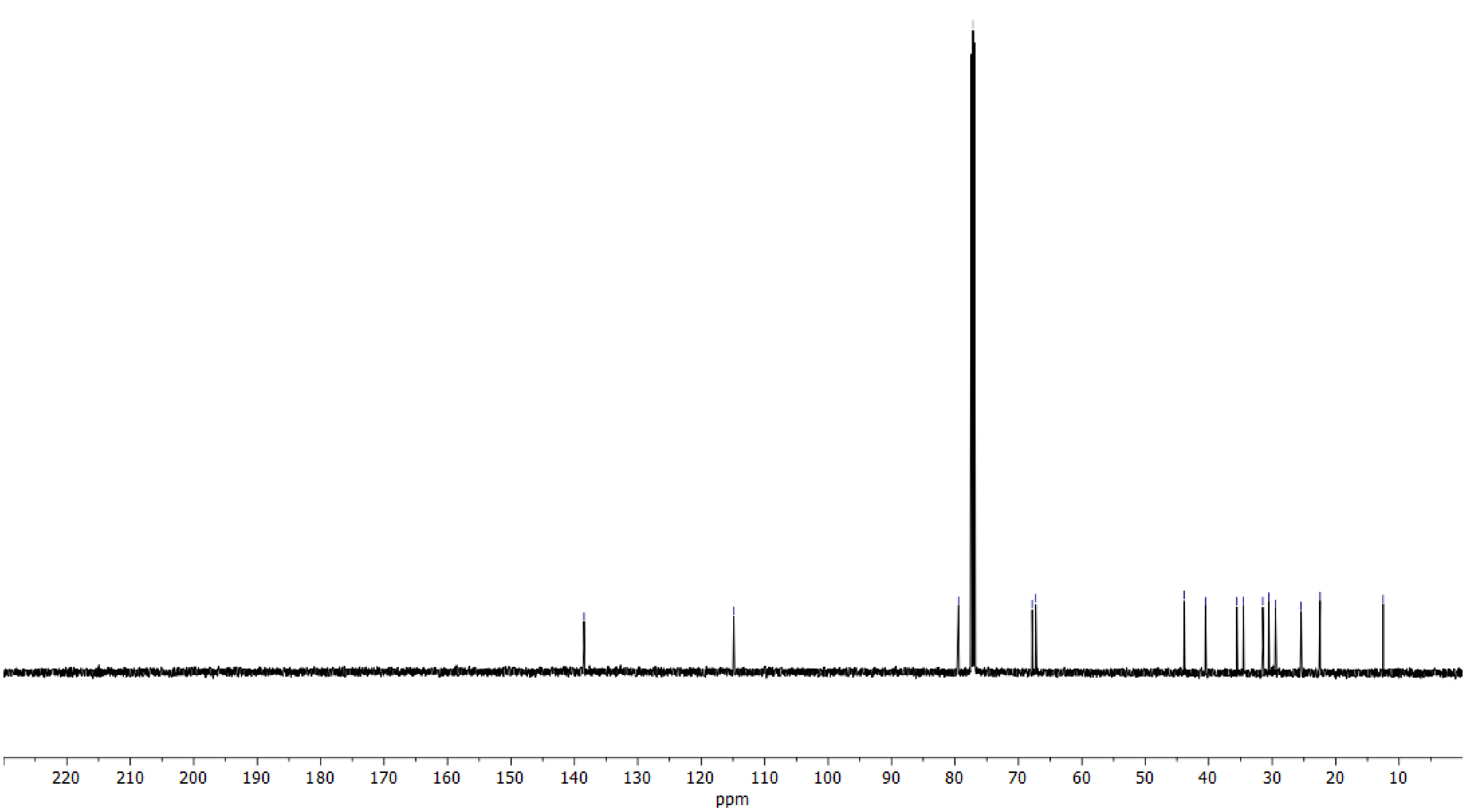

^{1}H NMR of primary alcohol 6o

$CDCl_3$, 500 MHz, 25 ºC

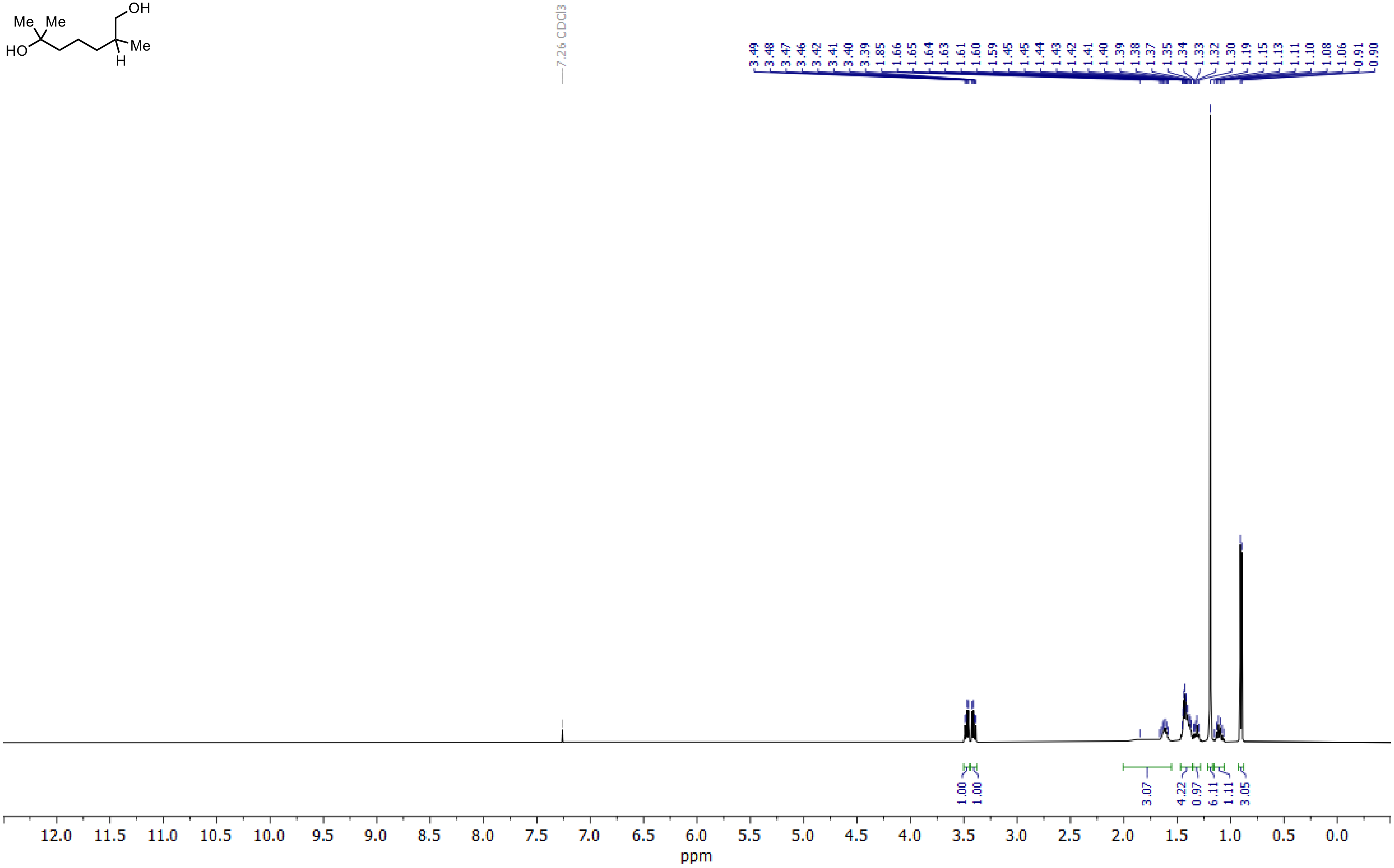

^{13}C NMR of primary alcohol 6o

$CDCl_3$, 125 MHz, 25 ºC

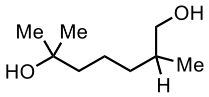

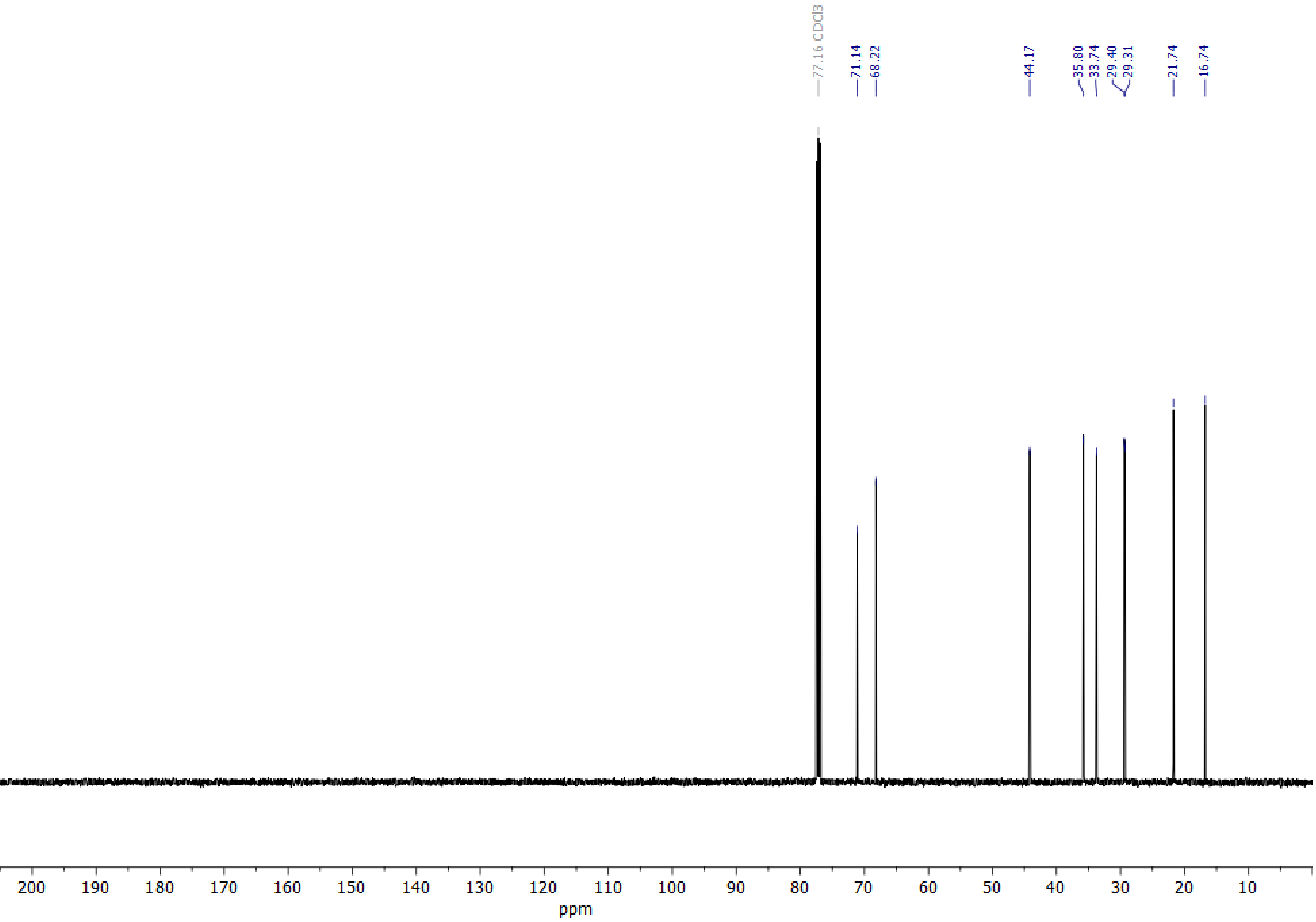

^{1}H NMR of primary alcohol 6p

$CDCl_3$, 500 MHz, 25 ºC

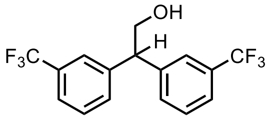

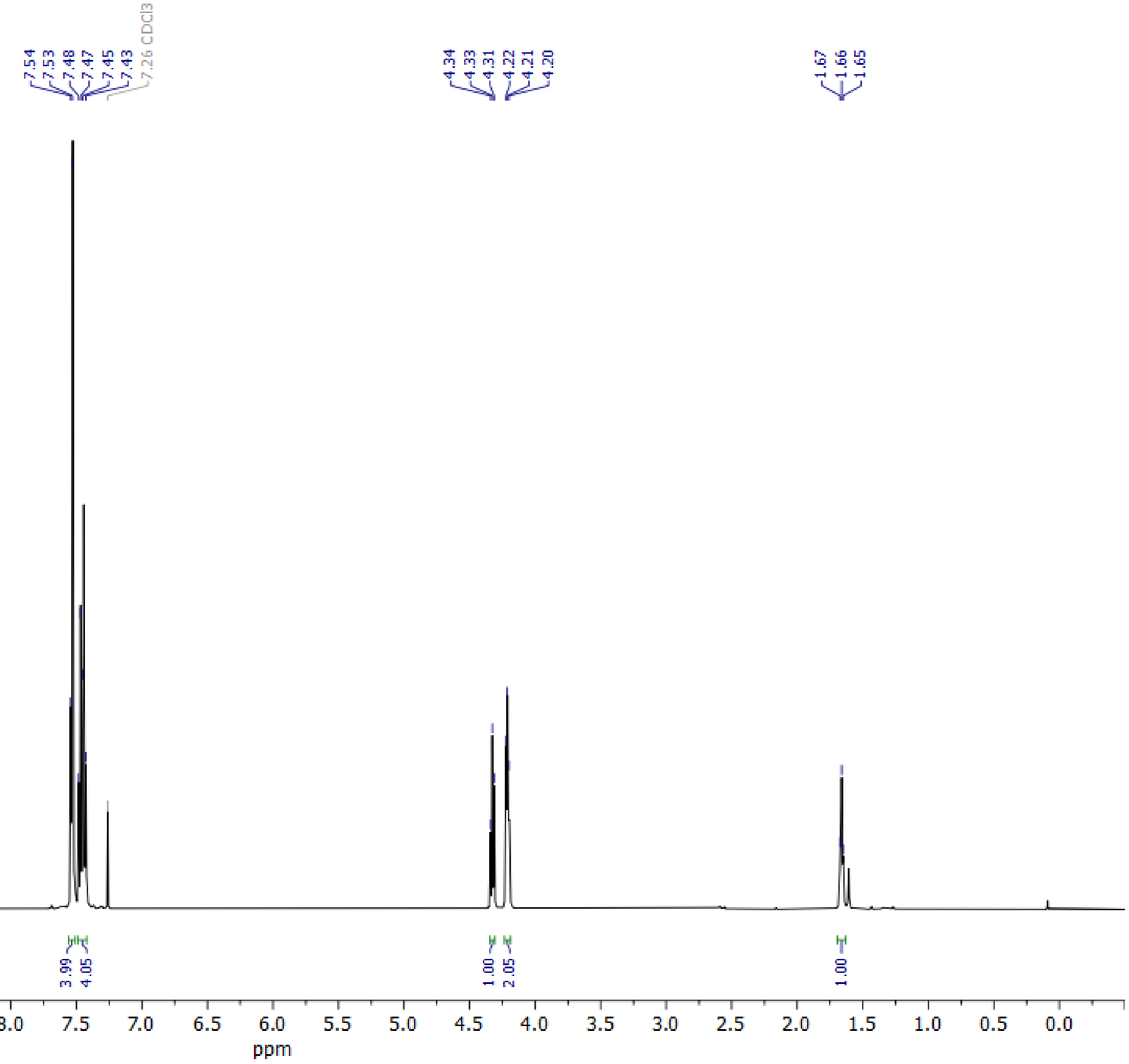

^{13}C NMR of primary alcohol 6p

CDCl$_3$, 125 MHz, 25 ºC

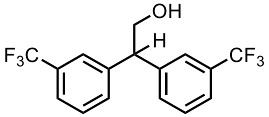

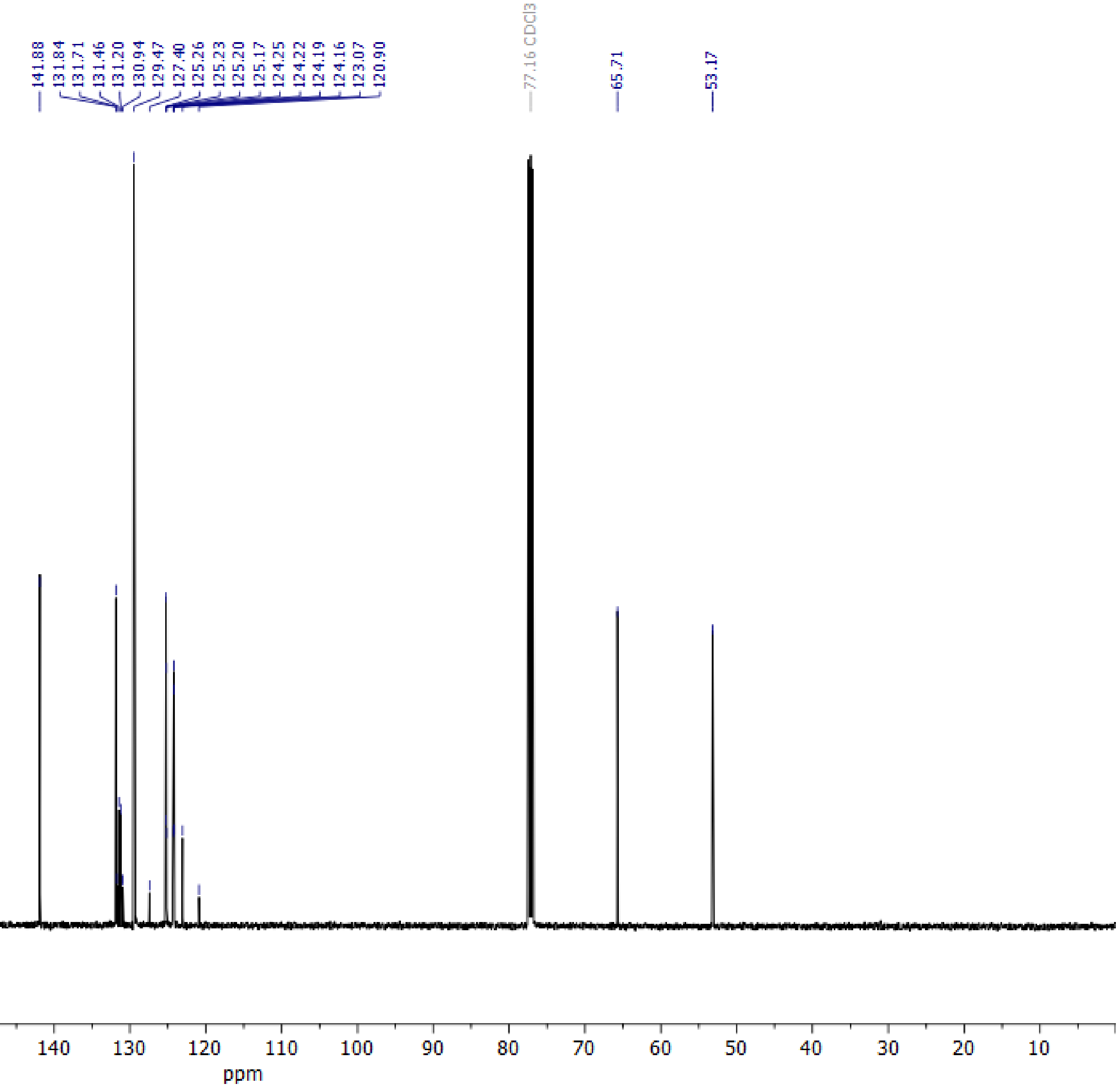

^{19}F NMR of primary alcohol 6p

$CDCl_3$, 470 MHz, 25 ºC

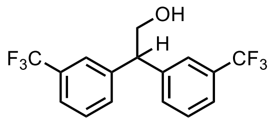

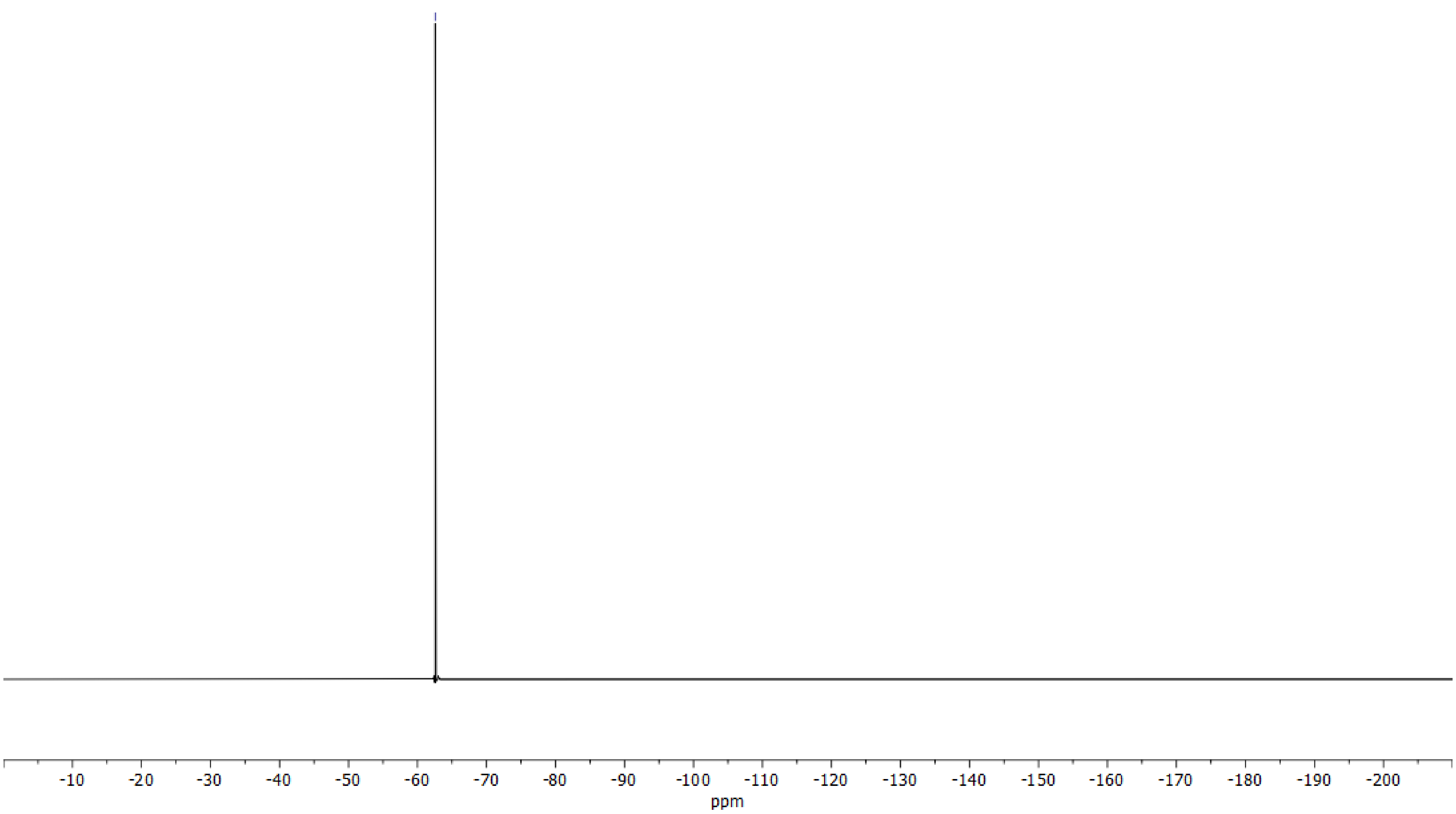

^{1}H NMR of secondary alcohol 6q

$CDCl_3$, 500 MHz, 25 ºC

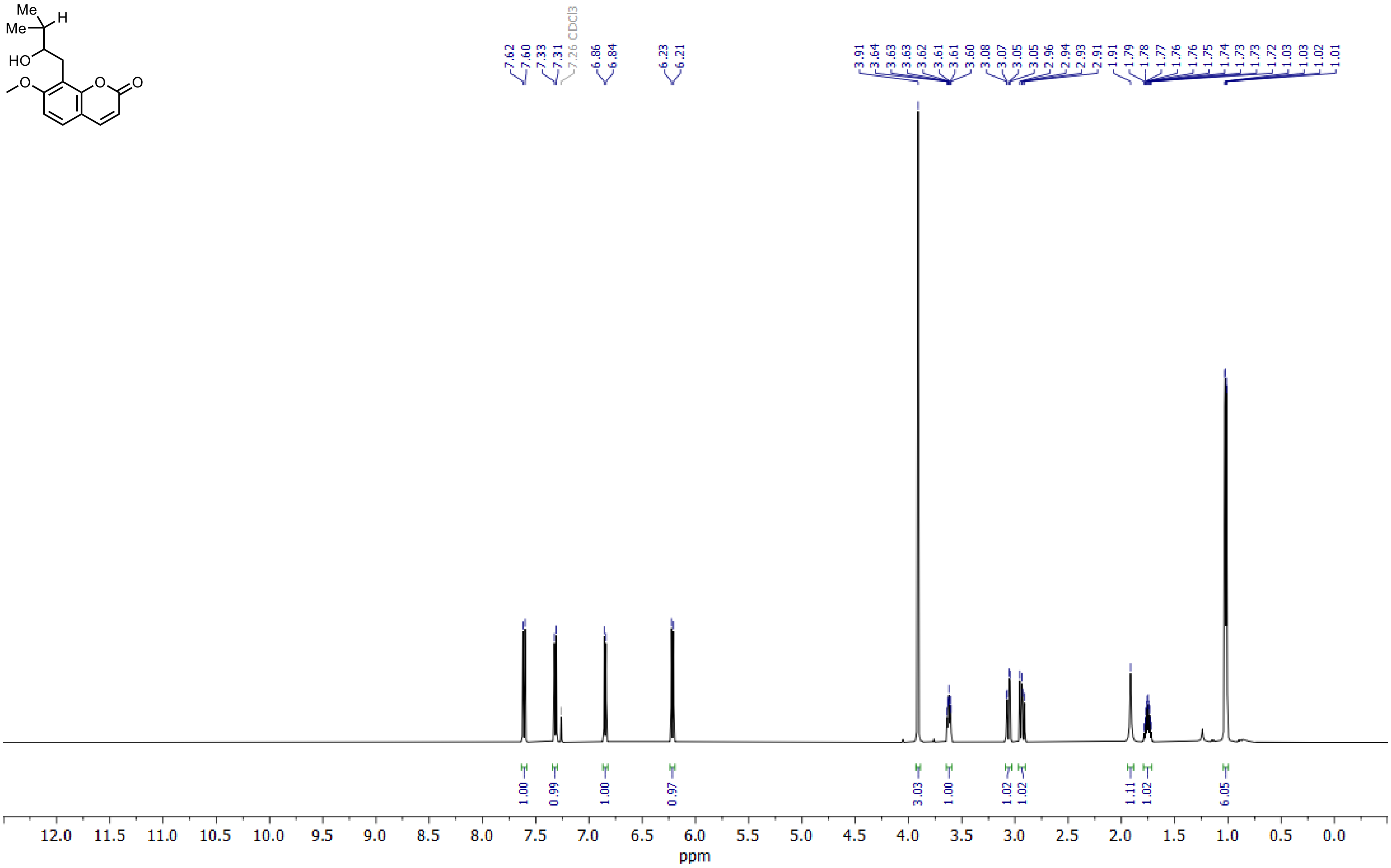

^{13}C NMR of secondary alcohol 6q

$CDCl_3$, 125 MHz, 25 ºC

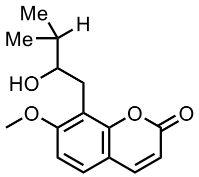

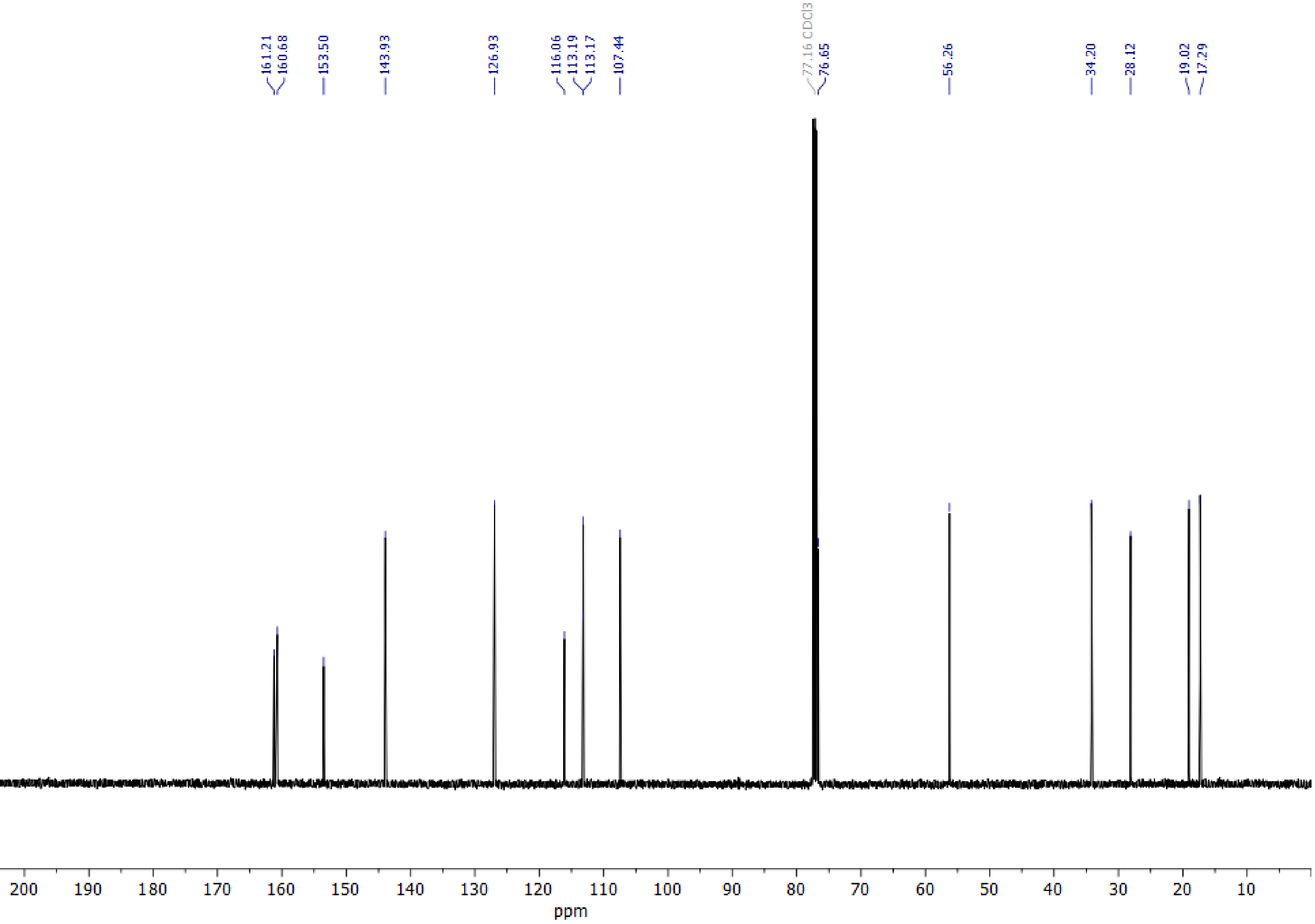

^{1}H NMR of tertiary alcohol 6q'

$CDCl_3$, 500 MHz, 25 ºC

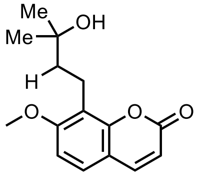

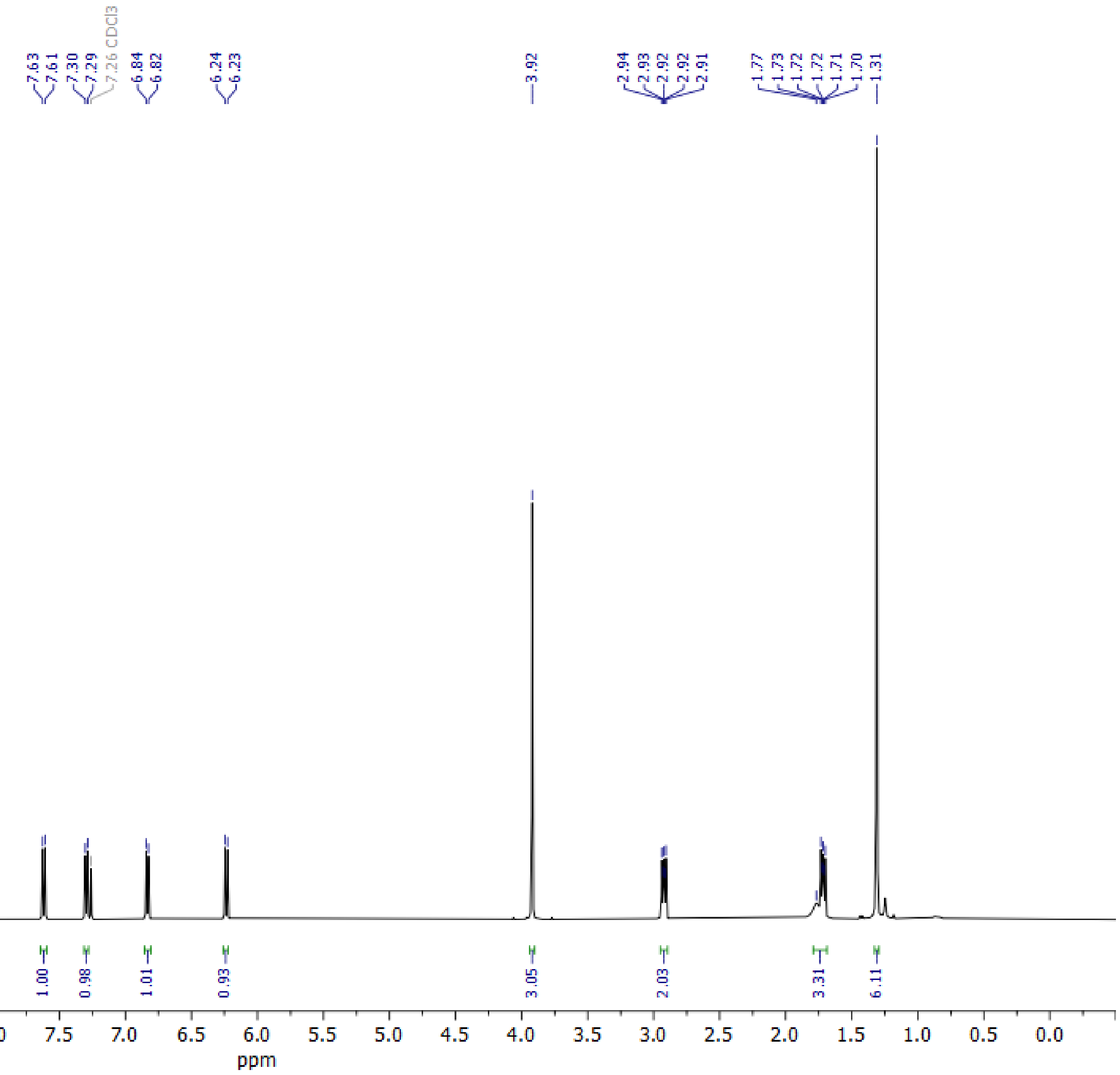

^{13}C NMR of tertiary alcohol 6q’

CDCl₃, 125 MHz, 25 ºC

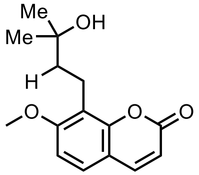

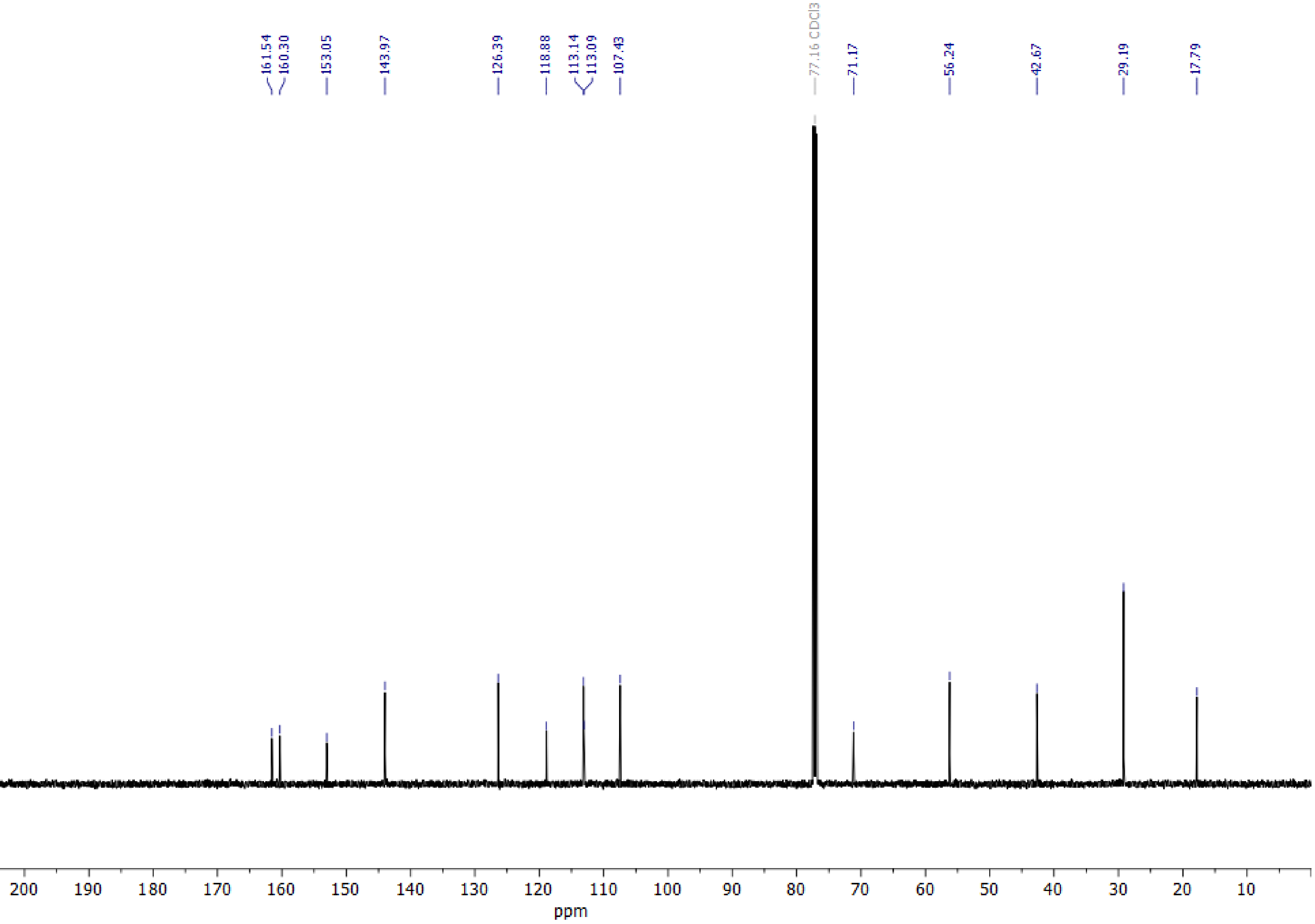

^{1}H NMR of secondary alcohol 6r

$CDCl_3$, 500 MHz, 25 ºC

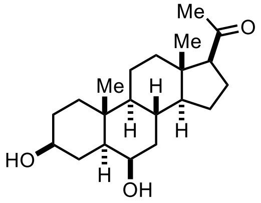

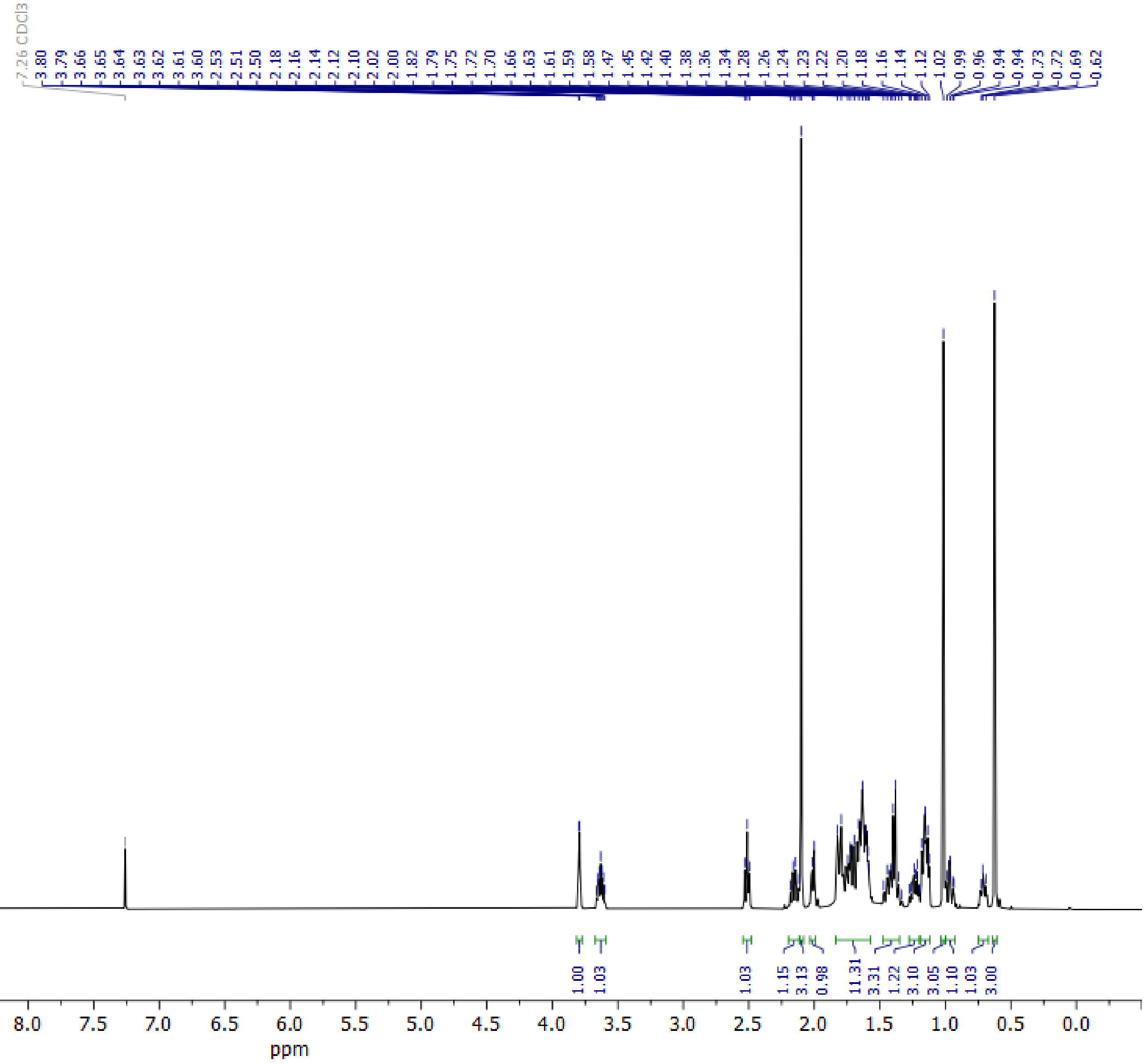

^{13}C NMR of secondary alcohol 6r

CDCl$_3$, 125 MHz, 25 ºC

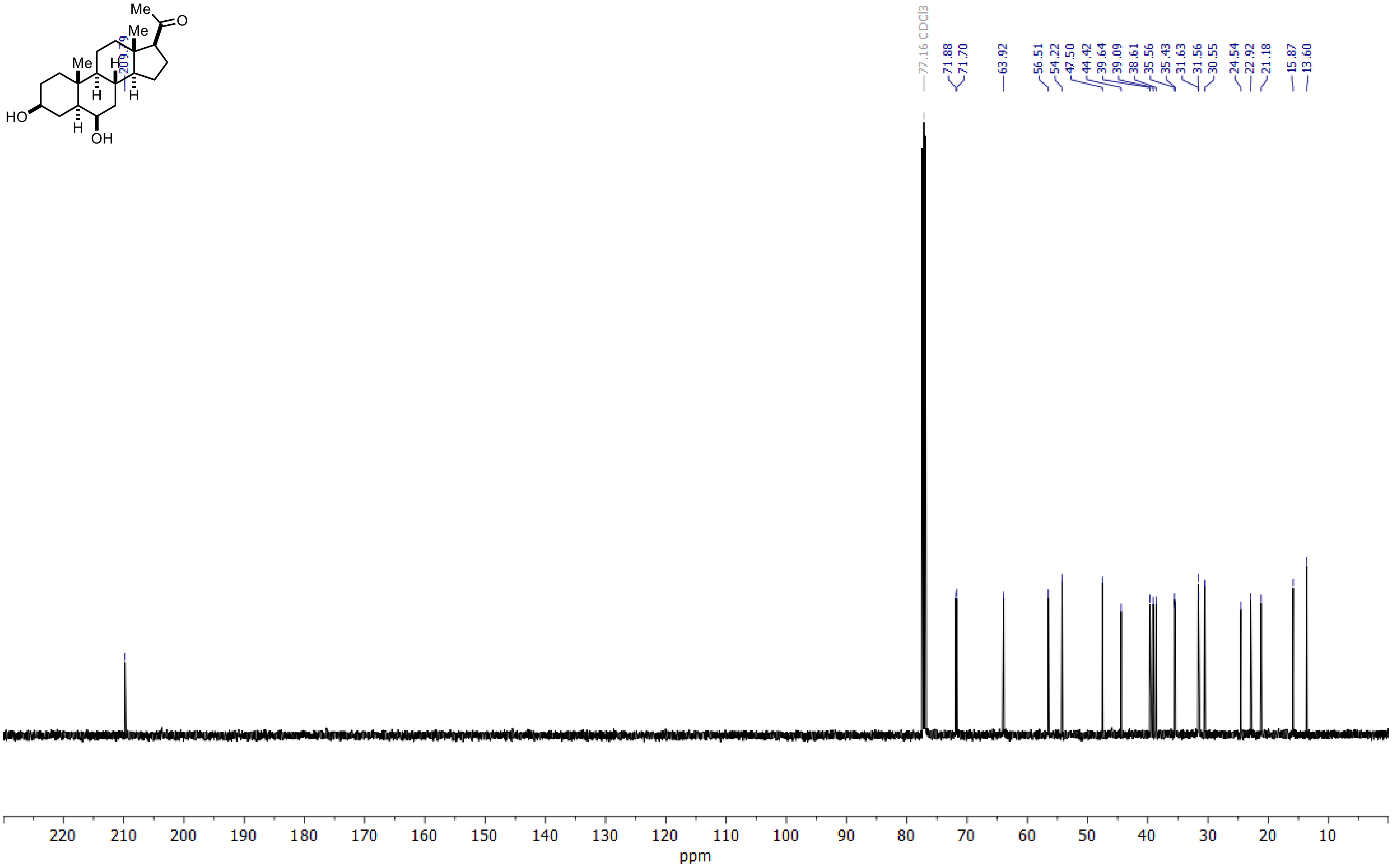

^{1}H NMR of secondary alcohol 6s

$CDCl_3$, 500 MHz, 25 ºC

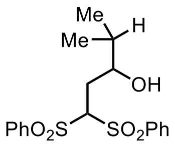

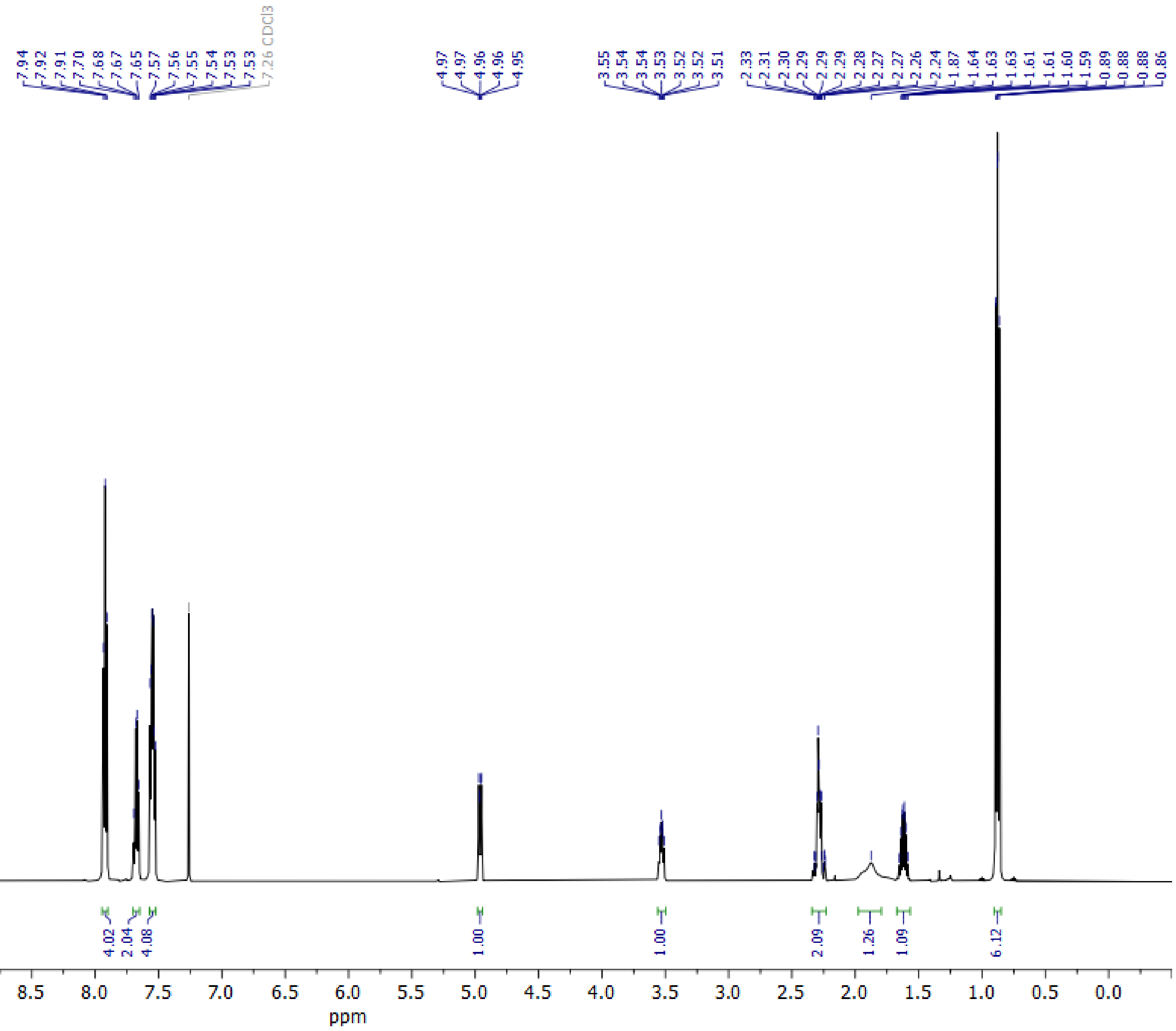

^{13}C NMR of secondary alcohol 6s

$CDCl_3$, 125 MHz, 25 ºC

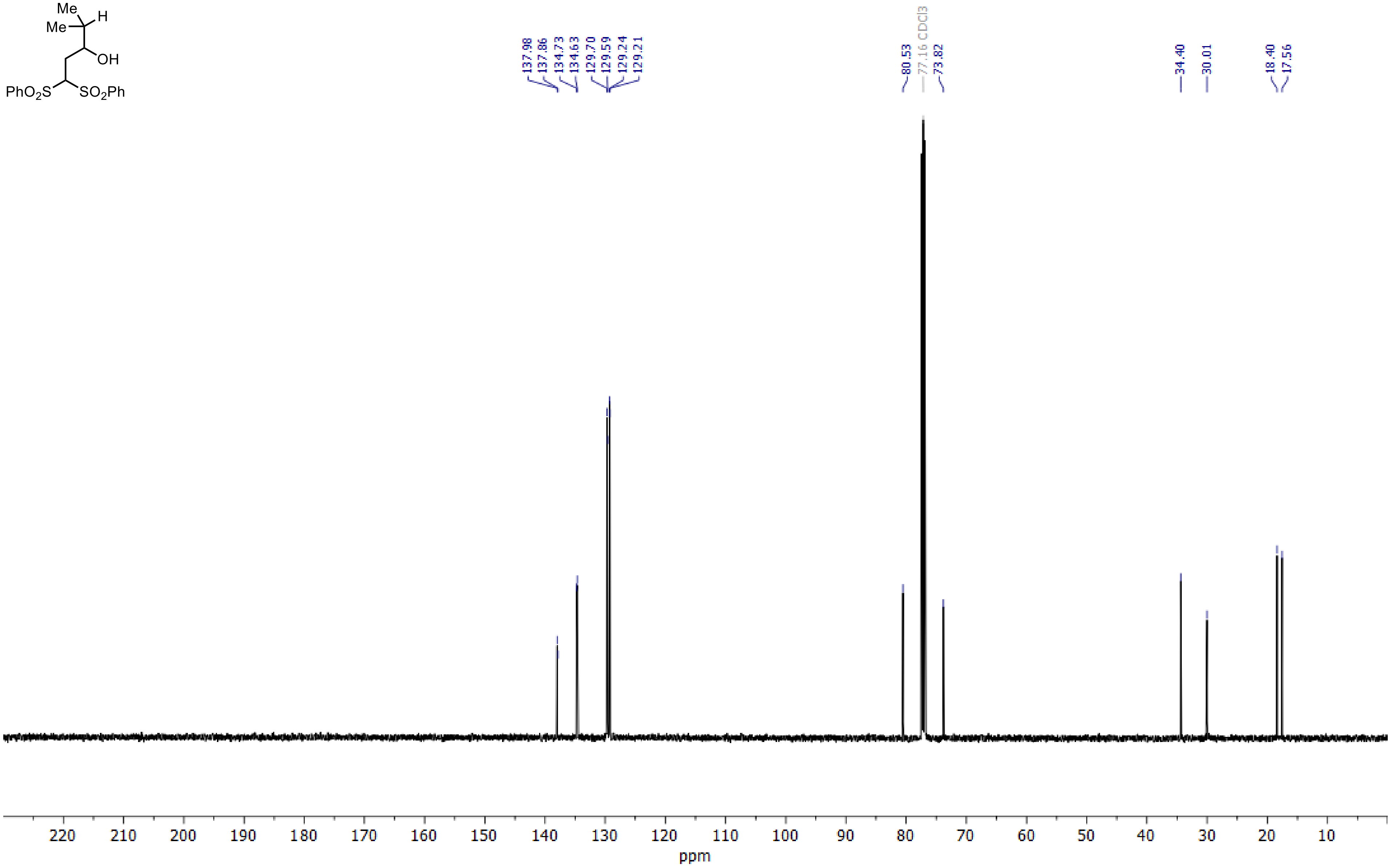

^{1}H NMR of tertiary alcohol 6s’

CDCl3, 500 MHz, 25 ºC

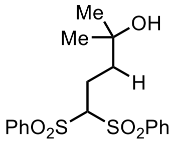

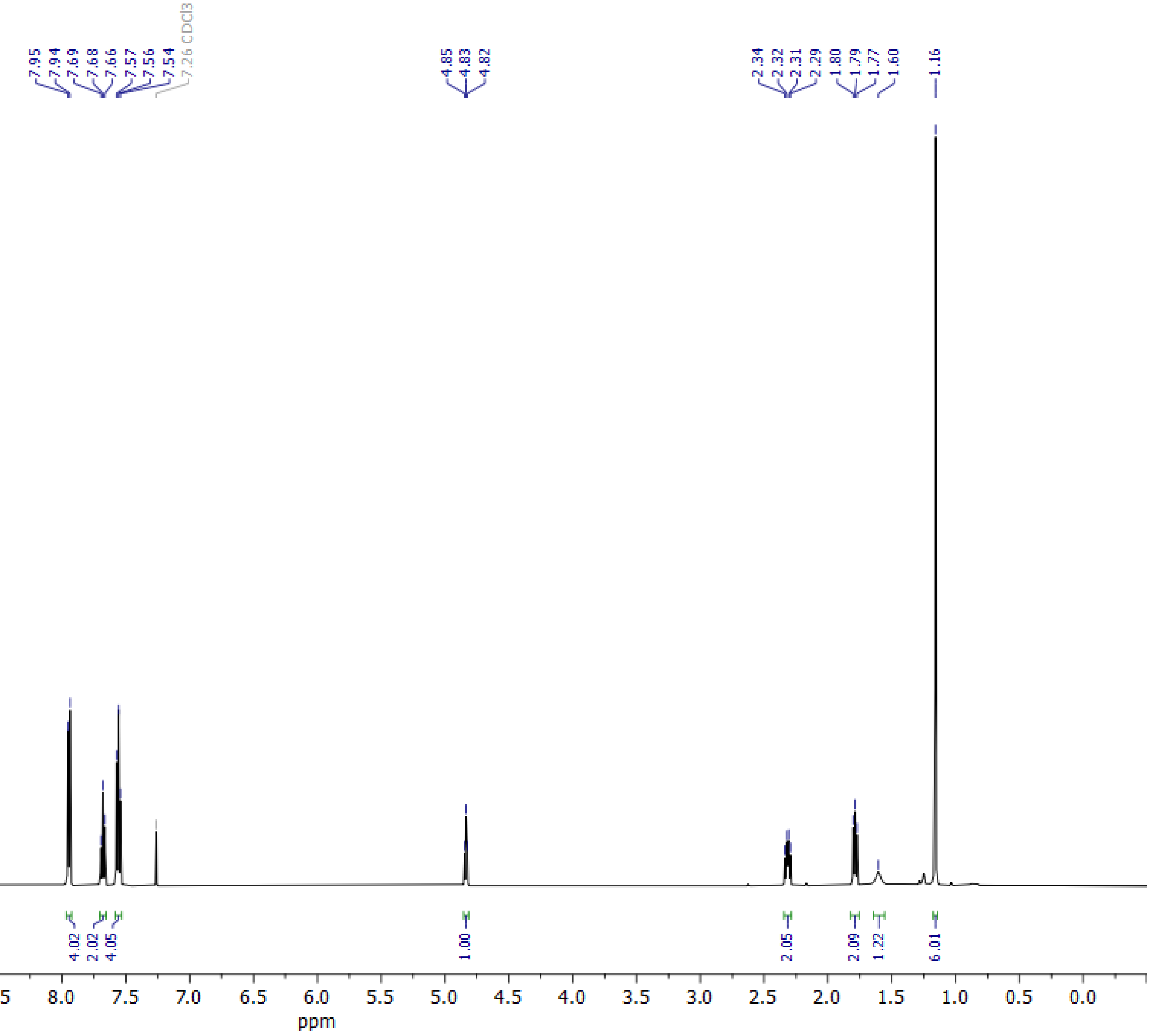

^{13}C NMR of tertiary alcohol 6s'

$CDCl_3$, 125 MHz, 25 ºC

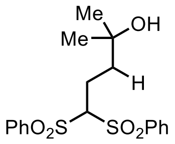

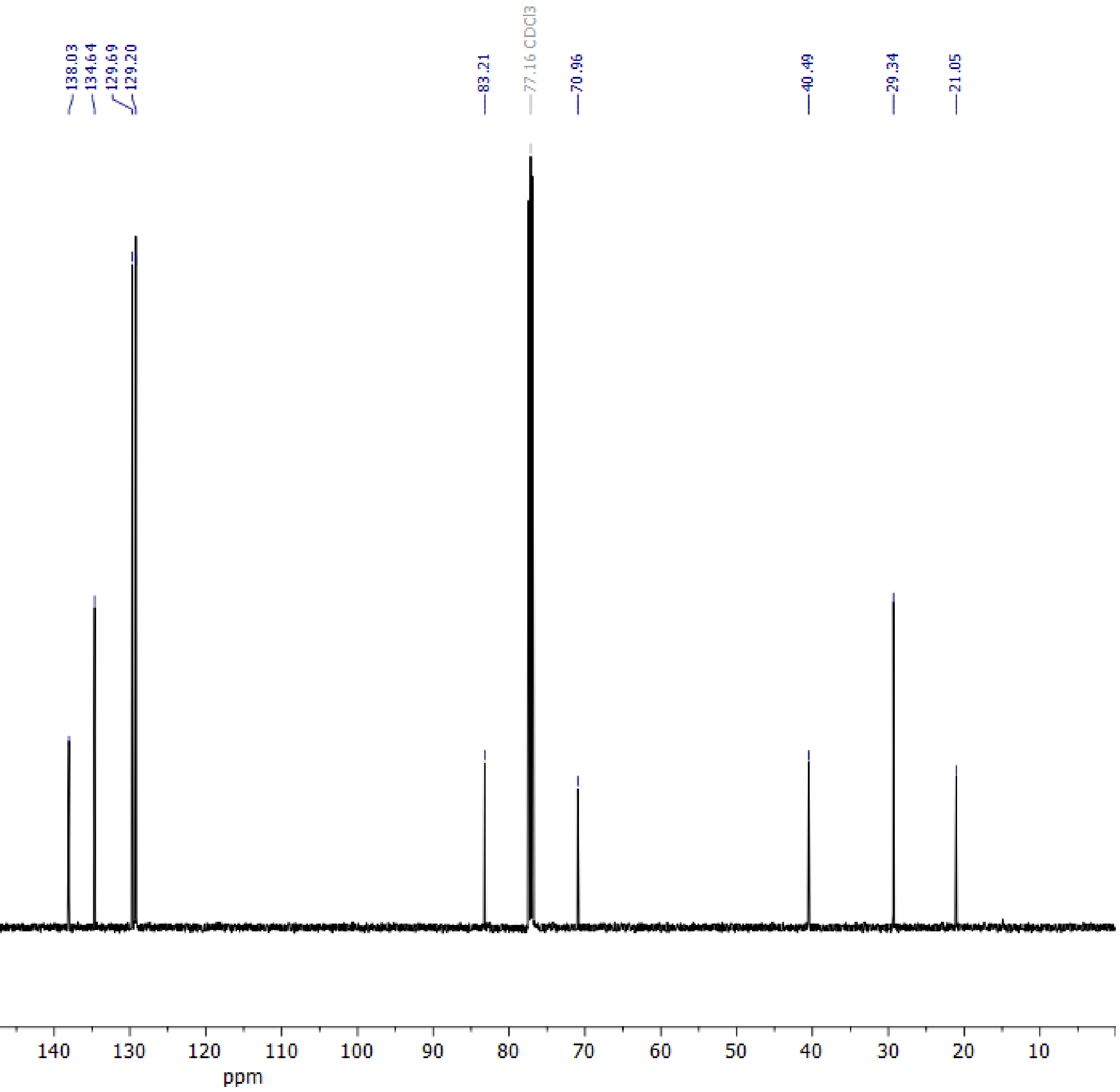

^{1}H NMR of primary alcohol 6t

$CDCl_3$, 500 MHz, 25 ºC

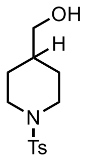

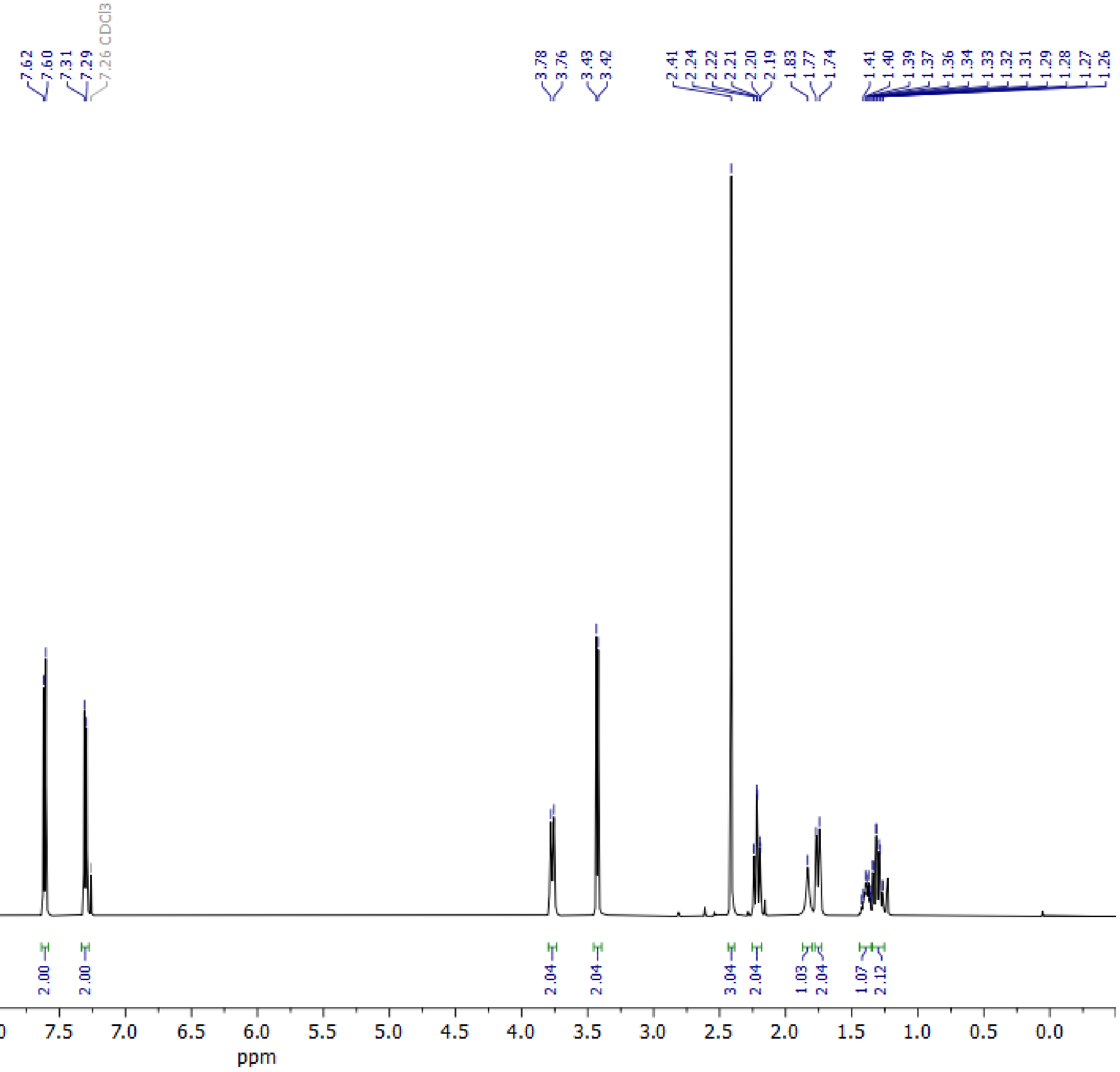

^{13}C NMR of primary alcohol 6t

$CDCl_3$, 125 MHz, 25 ºC

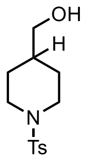

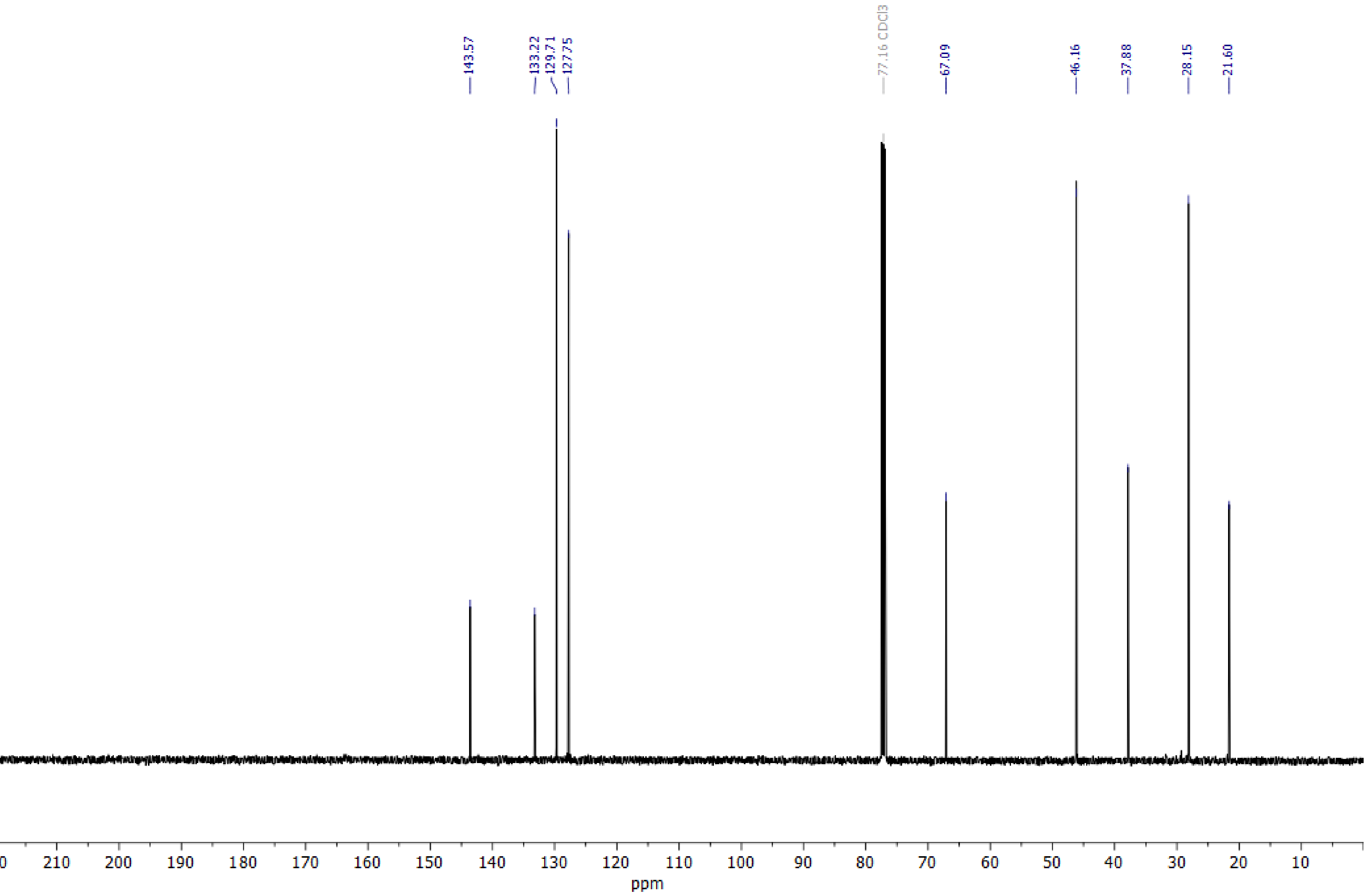

^{1}H NMR of methyl ether 7

$CDCl_3$, 500 MHz, 25 ºC

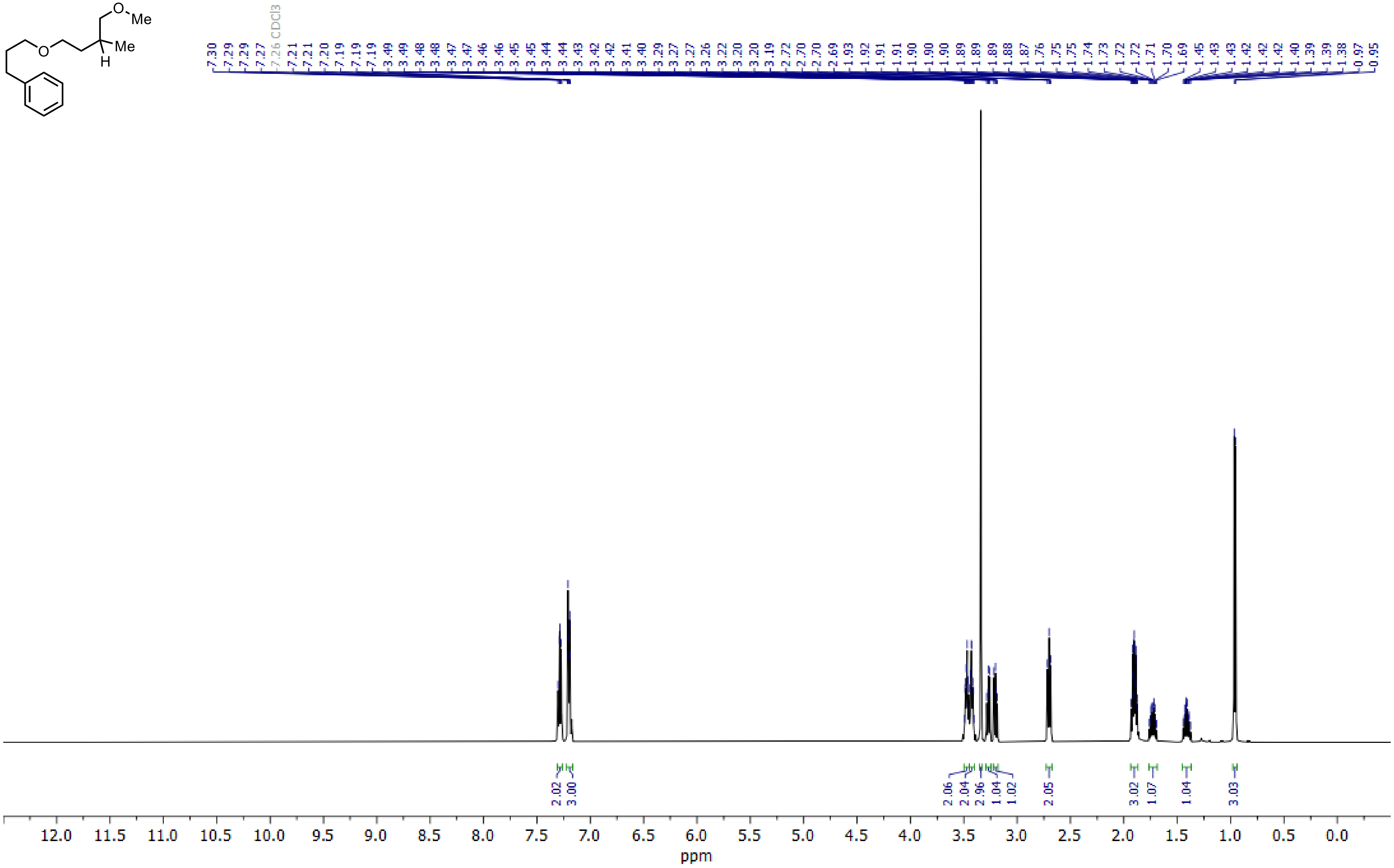

^{13}C NMR of methyl ether 7

$CDCl_3$, 125 MHz, 25 ºC

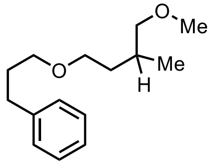

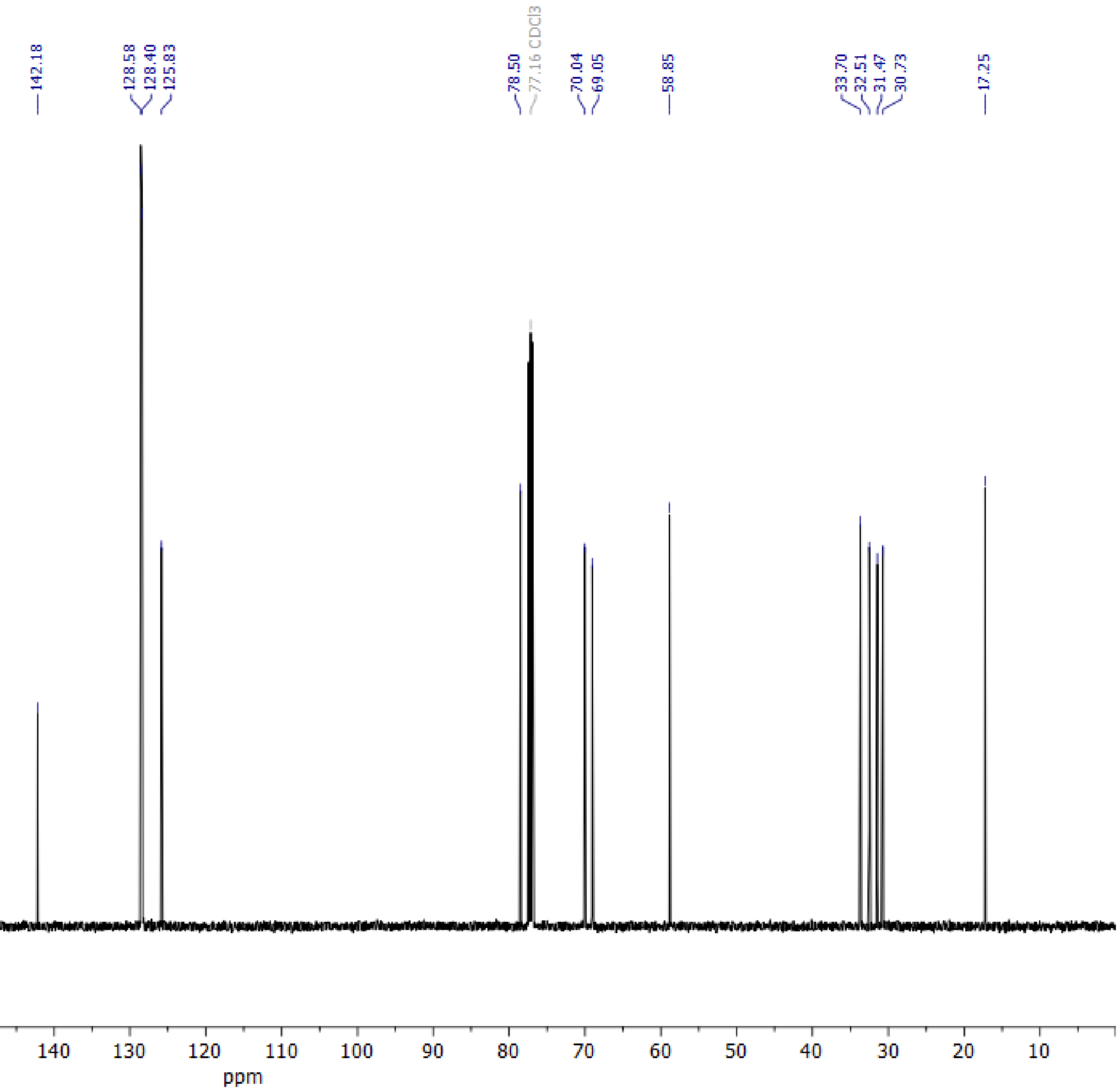

^{1}H NMR of ethyl ether 8

$CDCl_3$, 500 MHz, 25 ºC

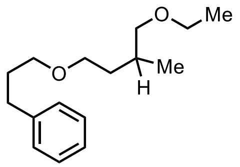

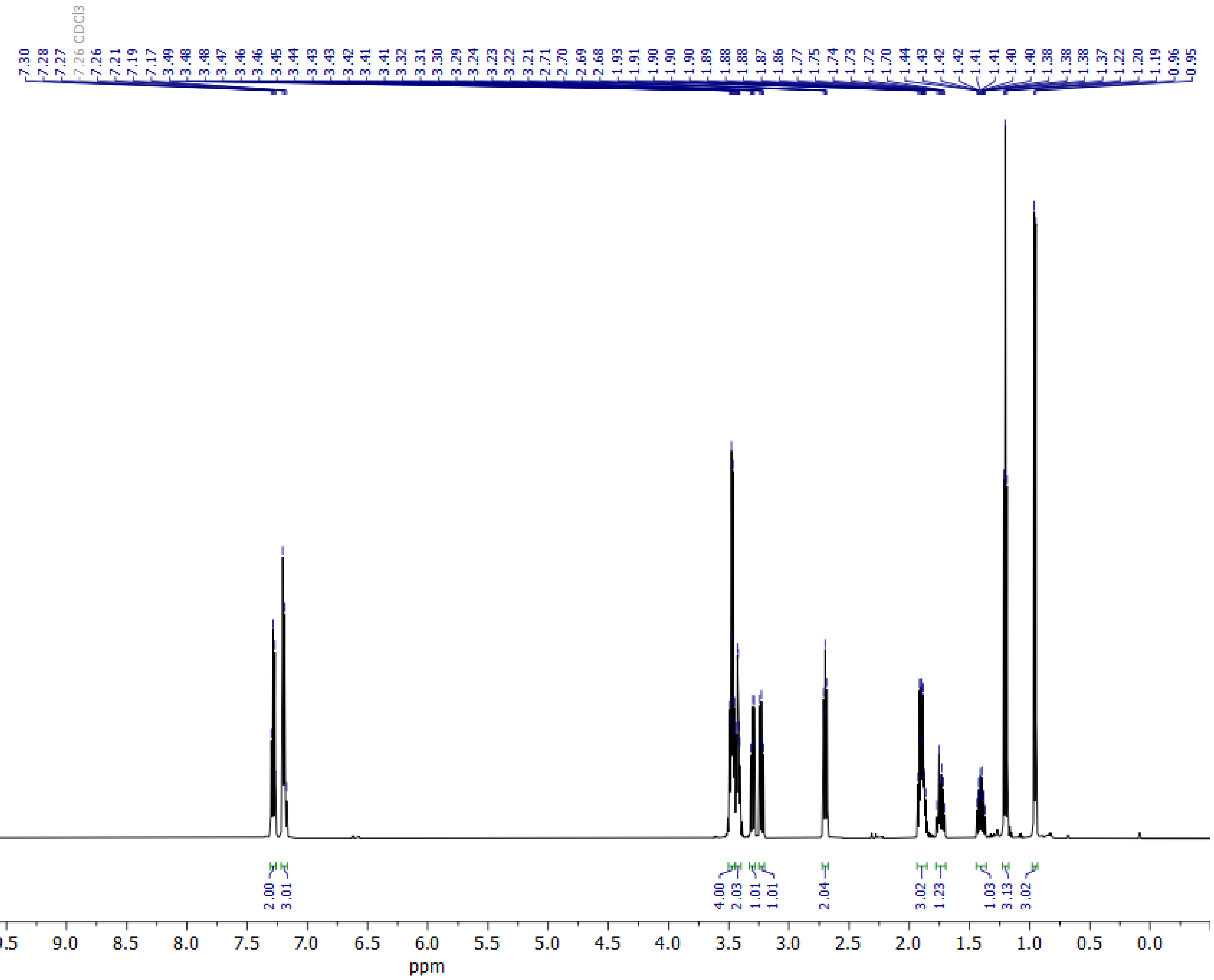

^{13}C NMR of ethyl ether 8

$CDCl_3$, 125 MHz, 25 ºC

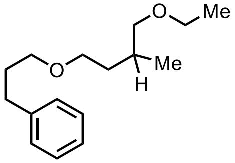

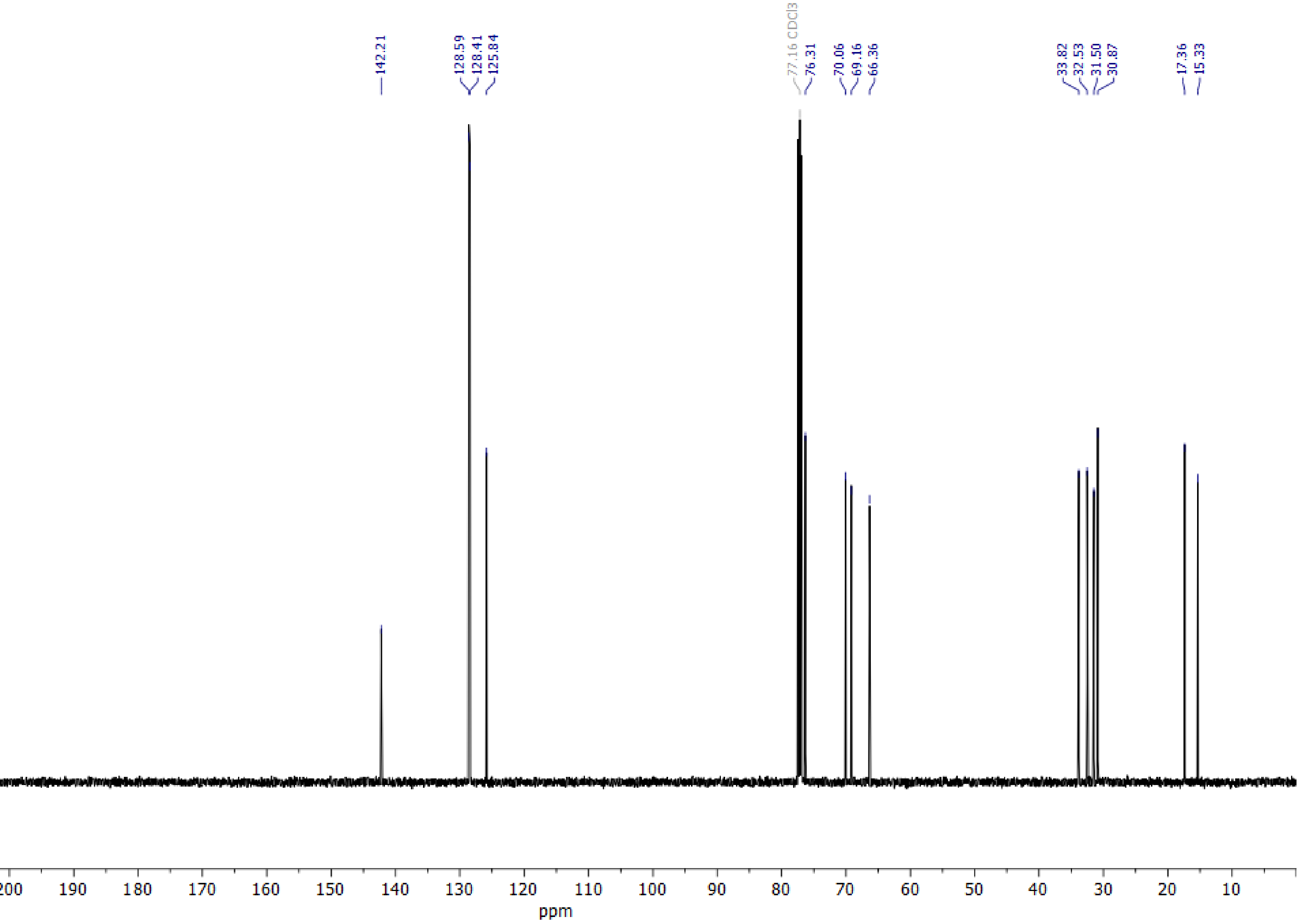

^{1}H NMR of isopropyl ether 9

$CDCl_3$, 500 MHz, 25 ºC

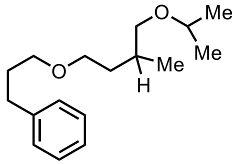

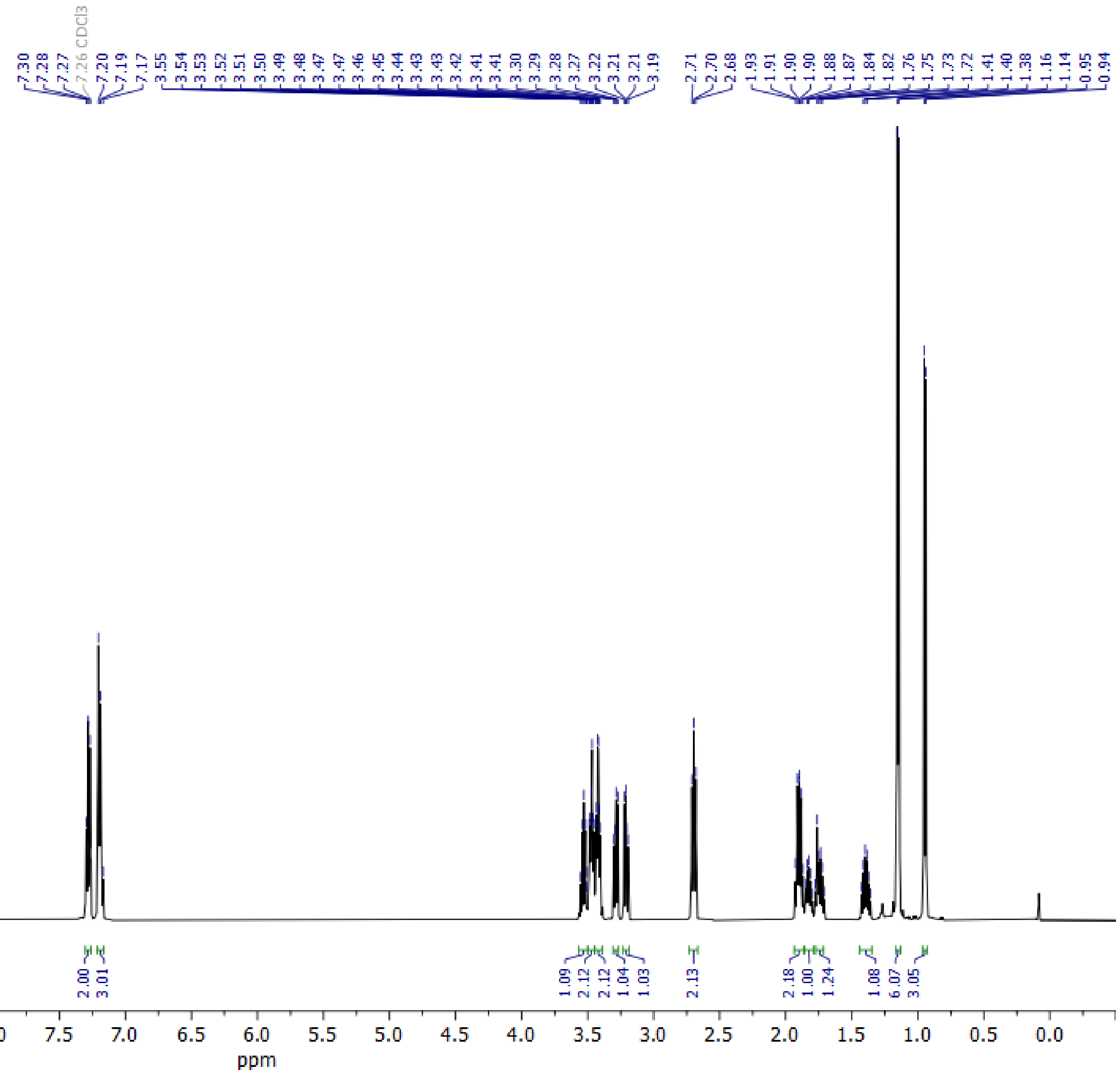

^{13}C NMR of isopropyl ether 9

$CDCl_3$, 125 MHz, 25 ºC

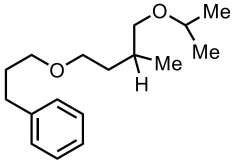

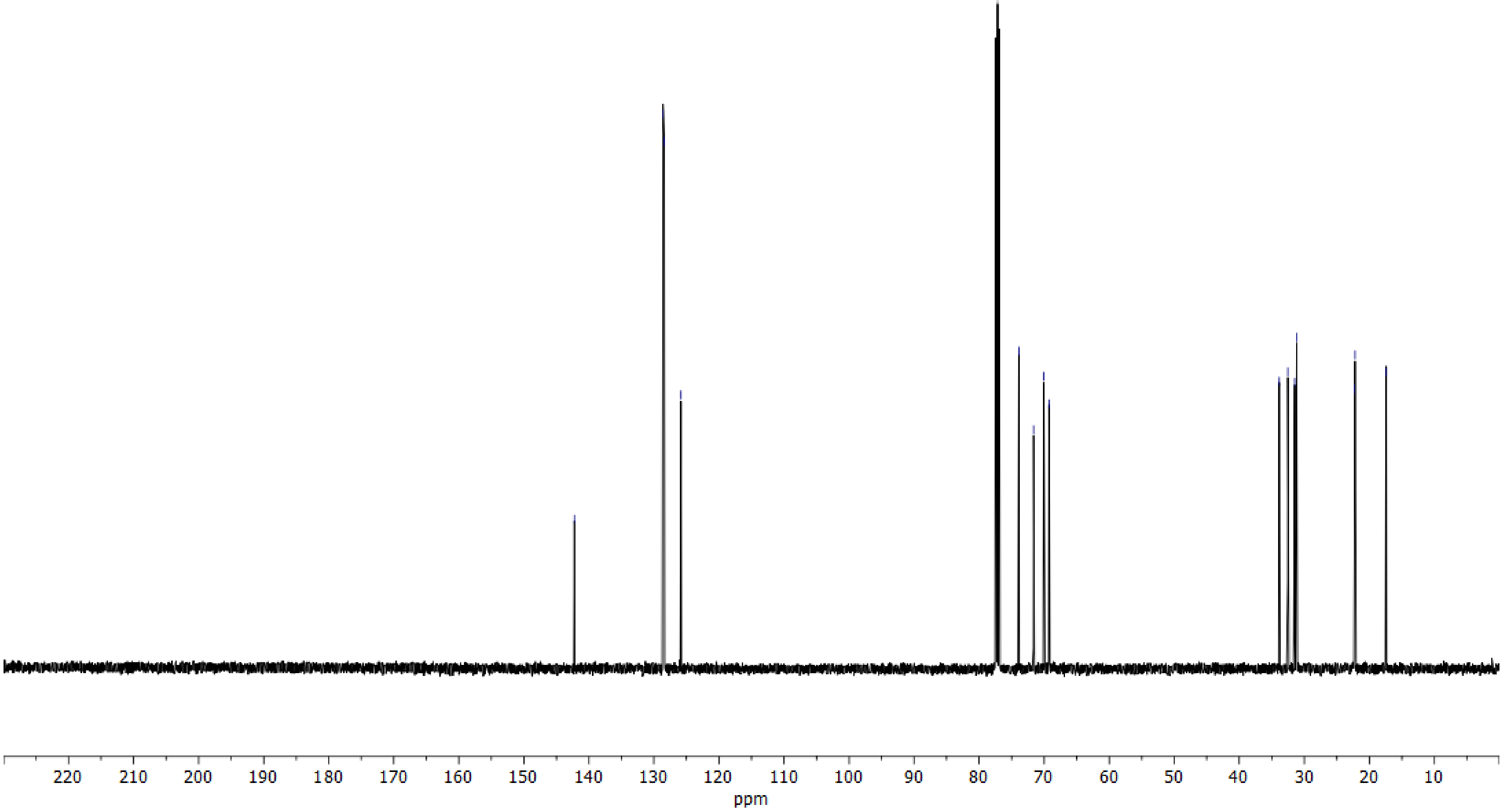

SUPPLEMENTARY REFERENCES

(1) Fulmer, G. R.; Miller, A. J. M.; Sherden, N. H.; Gottlieb, H. E.; Nudelman, A.; Stoltz, B. M.; Bercaw, J. E.; Goldberg, K. I. NMR Chemical Shifts of Trace Impurities: Common Laboratory Solvents, Organics, and Gases in Deuterated Solvents Relevant to the Organometallic Chemist. *Organometallics* **2010**, *29, 9,* 2176–2179.

(2) Ayotte, Y.; Woo, S.; LaPlante, S. R. Practical Considerations and Guidelines for Spectral Referencing for Fluorine NMR Ligand Screening. *ACS Omega* **2022**, *7, 15,* 13155–13163.

(3) Kim, J.; Sun, X.; van der Worp, B. A.; Ritter, T. Anti-Markovnikov Hydrochlorination and Hydronitrooxylation of α-Olefins via Visible-Light Photocatalysis. *Nat. Catal.* **2023**, *6*, 196–203.

(4) Cai, L.; Qian, X.; Song, W.; Liu, T.; Tao, X.; Li, W.; Xie, X. Effects of Solvent and Base on the Palladium-Catalyzed Amination: $PdCl_2(Ph_3P)_2/Ph_3P$-Catalyzed Selective Arylation of Primary Anilines with Aryl Bromides. *Tetrahedron* **2014**, *70, 32*, 4754–4759.

(5) Miller, D. C.; Ganley, J. M.; Musacchio, A. J.; Sherwood, T. C.; Ewing, W. R.; Knowles, R. R. Anti-Markovnikov Hydroamination of Unactivated Alkenes with Primary Alkyl Amines. *J. Am. Chem. Soc.* **2019**, *141*, *42*, 16590–16594.

(6) Lü, S.; Wang, Z.; Gao, X.; Chen, K.; Zhu, S. 1,2-Difunctionalization of Acetylene Enabled by Light. *Angew. Chem., Int. Ed.* **2023**, *62*, e202300268; *Angew. Chem.* **2023**, *135*, e202300268.

(7) Nambu, H.; Hata, K.; Matsugi, M.; Kita, Y. Efficient Synthesis of Thioesters and Amides from Aldehydes by Using an Intermolecular Radical Reaction in Water. *Chem. Eur. J.* **2005**, *11, 3,* 719–727.

(8) Mills, L. R.; Graham, J. M.; Patel, P.; Rousseaux, S. A. L. Ni-Catalyzed Reductive Cyanation of Aryl Halides and Phenol Derivatives via Transnitrilation. *J. Am. Chem. Soc.* **2019**, *141, 49,* 19257–19262.

(9) Soley, J.; Taylor, S. D. Mild, Rapid, and Chemoselective Procedure for the Introduction of the 9-Phenyl-9-fluorenyl Protecting Group into Amines, Acids, Alcohols, Sulfonamides, Amides, and Thiols. *J. Org. Chem.* **2020**, *85, 4,* 2068–2081.

(10) Muto, K.; Kumagai, T.; Kakiuchi, F.; Kochi, T. Remote Arylative Substitution of Alkenes Possessing an Acetoxy Group via β-Acetoxy Elimination. *Angew. Chem., Int. Ed.* **2021**, *60*, 24500.

(11) Brown, G. D.; Liang, G.-Y.; Sy, L.-K. Terpenoids from the Seeds of *Artemisia annua*. *Phytochemistry* **2003**, *64*, *1,* 303–323.

(12) Xie, Y.; Sun, P.-W.; Li, Y.; Wang, S.; Ye, M.; Li, Z. Ligand-Promoted Iron(III)-Catalyzed Hydrofluorination of Alkenes. *Angew. Chem., Int. Ed.* **2019**, *58*, 7097.

(13) Murphy, J. A.; Schoenebeck, F.; Findlay, N. J.; Thomson, D. W.; Zhou, S.-z.; Garnier, J. One-Carbon Extrusion from a Tetraazafulvalene: Isolation of Aldehydes and a Study of Their Origin. *J. Am. Chem. Soc.* **2009**, *131, 18,* 6475–6479.

(14) Schevenels, F. T.; Shen, M.; Snyder, S. A. Isolable and Readily Handled Halophosphonium Pre-reagents for Hydro- and Deuteriohalogenation. *J. Am. Chem. Soc.* **2017**, *139, 18,* 6329–6337.

(15) Trost, B. M.; Flygare, J. A. A Practical Synthesis of Rosefuran: Furans from Acetylenes and Allyl Alcohols. *J. Org. Chem.* **1994**, *59, 5,* 1078–1082.

(16) Wu, X.; Cruz, F. A.; Lu, A.; Dong, V. M. Tandem Catalysis: Transforming Alcohols to Alkenes by Oxidative Dehydroxymethylation. *J. Am. Chem. Soc.* **2018**, *140, 32,* 10126–10130.

(17) Nader, B. S.; Cordova, J. A.; Reese, K. E.; Powell, C. L. A Novel Fluoride Ion Mediated Olefination of Electron-Deficient Aryl Ketones by Alkanesulfonyl Halides. *J. Org. Chem.* **1994**, *59, 10,* 2898–2901.

(18) Muñoz, M. P.; Méndez, M.; Nevado, C.; Cárdenas, D. J.; Echavarren, A. M. Hydroxy- and Alkoxycyclizations of Enynes Catalyzed by Platinum(II) Chloride. *Synthesis* **2003**, *2003, 18,* 2898–2902.

(19) Nieto-Oberhuber, C.; Pérez-Galán, P.; Herrero-Gómez, E.; Lauterbach, T.; Rodríguez, C.; López, S.; Bour, C.; Rosellón, A.; Cárdenas, D. J.; Echavarren, A. M. Gold(I)-Catalyzed Intramolecular [4+2] Cycloadditions of Arylalkynes or 1,3-Enynes with Alkenes: Scope and Mechanism. *J. Am. Chem. Soc.* **2008**, *130*, *1,* 269–279.

(20) Oe, Y.; Yoshida, R.; Tanaka, A.; Adachi, A.; Ishibashi, Y.; Okazoe, T.; Aikawa, K.; Hashimoto, T. An N-Fluorinated Imide for Practical Catalytic Imidations. *J. Am. Chem. Soc.* **2022**, *144, 5,* 2107–2113.

(21) Furman, B.; Dziedzic, M. An Efficient Route to 4-(Substituted Benzyl)piperidines. *Tetrahedron Lett.* **2003**, *44*, *45,* 8249–8252.

(22) Shigehisa, H.; Ano, T.; Honma, H.; Ebisawa, K.; Hiroya, K. Co-Catalyzed Hydroarylation of Unactivated Olefins. *Org. Lett.* **2016**, *18, 15,* 3622–3625.

(23) Romero, N. A.; Nicewicz, D. A. Mechanistic Insight into the Photoredox Catalysis of Anti-Markovnikov Alkene Hydrofunctionalization Reactions. *J. Am. Chem. Soc.* **2014**, *136, 49,* 17024–17035.

(24) Albert, A.; Phillips, J. N. Ionization Constants of Heterocyclic Substances. Part II. Hydroxy-Derivatives of Nitrogenous Six-Membered Ring-Compounds. *J. Chem. Soc.* **1956**, 1294–1304.

(25) Zhang, K.; Chang, L.; An, Q.; Wang, X.; Zuo, Z. Dehydroxymethylation of Alcohols Enabled by Cerium Photocatalysis. *J. Am. Chem. Soc.* **2019**, *141, 26,* 10556–10564.

(26) Hui, S.-M.; Ngo, K.-S.; Brown, G. D. Epimerization in Acid Degradation Products of Artemisinin. *J. Chem. Soc., Perkin Trans. 1* **1997**, *1997, 0,* 3435–3442.

(27) Furusawa, M.; Hashimoto, T.; Noma, Y.; Asakawa, Y. Biotransformation of Citrus Aromatics Nootkatone and Valencene by Microorganisms. *Chem. Pharm. Bull.* **2005**, *53, 11,* 1423–1429.

(28) Li, G.; Norton, J. R. Ti(III)-Catalyzed Anti-Markovnikov Reduction of Epoxides with Borohydride. *Org. Lett.* **2024**, *26, 7,* 1382–1386.

(29) Hu, P.; Peters, B. K.; Malapit, C. A.; Vantourout, J. C.; Wang, P.; Li, J.; Mele, L.; Echeverria, P.-G.; Minteer, S. D.; Baran, P. S. Electroreductive Olefin–Ketone Coupling. *J. Am. Chem. Soc.* **2020**, *142, 50,* 20979–20986.

(30) Bhunia, A.; Bergander, K.; Daniliuc, C. G.; Studer, A. Fe-Catalyzed Anaerobic Mukaiyama-Type Hydration of Alkenes Using Nitroarenes. *Angew. Chem., Int. Ed.* **2021**, *60*, 8313.

(31) Sulzer-Mossé, S.; Alexakis, A.; Mareda, J.; Bollot, G.; Bernardinelli, G.; Filinchuk, Y. Enantioselective Organocatalytic Conjugate Addition of Aldehydes to Vinyl Sulfones and Vinyl Phosphonates as Challenging Michael Acceptors. *Chem. Eur. J.* **2009**, *15*, *13,* 3204–3220.

(32) Spieß, P.; Sirvent, A.; Tiefenbrunner, I.; Sargueil, J.; Fernandes, A. J.; Arroyo-Bondía, A.; Meyrelles, R.; Just, D.; Prado-Roller, A.; Shaaban, S.; Kaiser, D.; Maulide, N. Nms-Amides: An Amine Protecting Group with Unique Stability and Selectivity. *Chem. Eur. J.* **2023**, *29*, e202301312.

(33) Pavlishchuk, V. V.; Addison, A. W. Conversion Constants for Redox Potentials Measured versus Different Reference Electrodes in Acetonitrile Solutions at 25 °C. *Inorg. Chim. Acta* **2000**, *298, 1,* 97–102.

(34) Tshepelevitsh, S.; Kütt, A.; Lõkov, M.; Kaljurand, I.; Saame, J.; Heering, A.; Plieger, P. G.; Vianello, R.; Leito, I. On the Basicity of Organic Bases in Different Media. *Eur. J. Org. Chem.* **2019**, *2019,* 6735–6748.

(35) Joshi-Pangu, A.; Lévesque, F.; Roth, H. G.; Oliver, S. F.; Campeau, L.-C.; Nicewicz, D.; DiRocco, D. A. Acridinium-Based Photocatalysts: A Sustainable Option in Photoredox Catalysis. *J. Org. Chem.* **2016**, *81, 16,* 7244–7249.

(36) Neese, F. The ORCA Program System. *WIREs Comput. Mol. Sci.* **2012**, *2*, 73–78.

(37) Chai, J.-D.; Head-Gordon, M. Long-Range Corrected Hybrid Density Functionals with Damped Atom–Atom Dispersion Corrections. *Phys. Chem. Chem. Phys.* **2008**, *10*, 6615–6620.

(38) Chai, J.-D.; Head-Gordon, M. Systematic Optimization of Long-Range Corrected Hybrid Density Functionals. *J. Chem. Phys.* **2008**, *128*, 084106.

(39) Caldeweyher, E.; Ehlert, S.; Hansen, A.; Neugebauer, H.; Spicher, S.; Bannwarth, C.; Grimme, S. A Generally Applicable Atomic-Charge Dependent London Dispersion Correction. *J. Chem. Phys.* **2019**, *150, 15,* 154122.

(40) Caldeweyher, E.; Bannwarth, C.; Grimme, S. Extension of the D3 Dispersion Coefficient Model. *J. Chem. Phys.* **2017**, *147, 3,* 034112.

(41) Weigend, F.; Ahlrichs, R. Balanced Basis Sets of Split Valence, Triple Zeta Valence and Quadruple Zeta Valence Quality for H to Rn: Design and Assessment of Accuracy. *Phys. Chem. Chem. Phys.* **2005**, *7*, 3297–3305.

(42) Riplinger, C.; Neese, F. An Efficient and Near Linear Scaling Pair-Natural Orbital Based Local Coupled Cluster Method. *J. Chem. Phys.* **2013**, *138*, 034106.

(43) Riplinger, C.; Sandhoefer, B.; Hansen, A.; Neese, F. Natural Triple Excitations in Local Coupled Cluster Calculations with Pair Natural Orbitals. *J. Chem. Phys.* **2013**, *139*, 134101.

(44) Riplinger, C.; Pinski, P.; Becker, U.; Valeev, E. F.; Neese, F. Sparse Maps: A Systematic Infrastructure for Reduced-Scaling Electronic Structure Methods. II. Linear Scaling Domain-Based Pair Natural Orbital Coupled Cluster Theory. *J. Chem. Phys.* **2016**, *144*, 024109.

(45) Saitow, M.; Becker, U.; Riplinger, C.; Valeev, E. F.; Neese, F. A New Near-Linear Scaling, Efficient and Accurate, Open-Shell Domain-Based Local Pair Natural Orbital Coupled Cluster Singles and Doubles Theory. *J. Chem. Phys.* **2017**, *146*, 164105.

(46) Guo, Y.; Riplinger, C.; Becker, U.; Liakos, D. G.; Minenkov, Y.; Cavallo, L.; Neese, F. Communication: An Improved Linear Scaling Perturbative Triples Correction for the Domain-Based Local Pair-Natural Orbital Singles and Doubles Coupled Cluster Method [DLPNO-CCSD(T)]. *J. Chem. Phys.* **2018**, *148*, 011101.

(47) Marenich, A. V.; Cramer, C. J.; Truhlar, D. G. Universal Solvation Model Based on Solute Electron Density and on a Continuum Model of the Solvent Defined by the Bulk Dielectric Constant and Atomic Surface Tensions. *J. Phys. Chem. B* **2009**, *113, 18,* 6378–6396.

(48) Valeev, E. F. Libint: A Library for the Evaluation of Molecular Integrals of Many-Body Operators over Gaussian Functions. *http://libint.valeyev.net/*.

(49) Weigend, F. Accurate Coulomb-Fitting Basis Sets for H to Rn. *Phys. Chem. Chem. Phys.* **2006**, *8*, 1057–1065.

(50) Hellweg, A.; Hättig, C.; Höfener, S.; et al. Optimized Accurate Auxiliary Basis Sets for RI-MP2 and RI-CC2 Calculations for the Atoms Rb to Rn. *Theor. Chem. Acc.* **2007**, *117*, 587–597.

(51) Zhurko, G. A. Chemcraft: Graphical Program for Visualization of Quantum Chemistry Computations. *https://chemcraftprog.com/*.

(52) Roth, H. G.; Romero, N. A.; Nicewicz, D. A. Experimental and Calculated Electrochemical Potentials of Common Organic Molecules for Applications to Single-Electron Redox Chemistry. *Synlett* **2016**, *27*, 714–723.

(53) Isse, A. A.; Gennaro, A. Absolute Potential of the Standard Hydrogen Electrode and the Problem of Interconversion of Potentials in Different Solvents. *J. Phys. Chem. B* **2010**, *114*, *23,* 7894–7899.
